# Central Pressure Waveform-Derived Indexes Obtained From Carotid and Radial Tonometry and Brachial Oscillometry in Healthy Subjects (2–84 Y): Age-, Height-, and Sex-Related Profiles and Analysis of Indexes Agreement

**DOI:** 10.3389/fphys.2021.774390

**Published:** 2022-01-20

**Authors:** Yanina Zócalo, Daniel Bia

**Affiliations:** Departamento de Fisiología, Facultad de Medicina, Centro Universitario de Investigación, Innovación y Diagnóstico Arterial (CUiiDARTE), Universidad de la República, Montevideo, Uruguay

**Keywords:** adolescents, adults, applanation tonometry, children, oscillometry, pulse wave analysis, wave separation analysis, normative data

## Abstract

Aortic blood pressure (aoBP) waveform-derived indexes could provide valuable (prognostic) information over and above cardiovascular risk factors (CRFs). To obtain aoBP waveform-characteristics, several (i) techniques, (ii) recording sites, (iii) pressure-only waveform analysis mathematical approaches [e.g., pulse wave analysis (PWA), wave separation analysis (WSA)], and (iv) indexes [augmentation pressure and index (AP and AIx), forward (Pf) and backward (Pb) components of aoBP, reflection magnitude (RM), and reflection index (Rix)], were proposed. An accurate clinical use of these indexes requires knowing their physiological age-related profiles and the expected values for a specific subject. There are no works that have characterized waveform-derived indexes profiles in large populations considering: (i) as a continuous, data from different age stages (childhood, adolescence, and adulthood), (ii) complementary indexes, (iii) data obtained from different techniques and approaches, and (iv) analyzing potential sex- and body height (BH)-related differences. In addition, (v) there is a lack of normative data (reference intervals, RIs) for waveform-derived indexes.

**Aims:**

(1) to evaluate the association and agreement between PWA- and/or WSA-derived indexes obtained with different techniques and approaches; (2) to determine the need for sex-, BH-, and/or age-specific RIs; (3) to define RIs for PWA- and WSA-derived indexes in a large cohort of healthy children, adolescents, and adults.

**Methods:**

3619 subjects (3–90 y) were included; 1688 healthy (2–84 y). AP, AIx, AIx@75, Pf, Pb, RM, and RIx were obtained (carotid and radial tonometry, brachial oscillometry/plethysmography). The association and agreement between indexes were analyzed (Concordance correlation coefficients, Bland–Altman analysis). Mean and SD equations and sex-specific BH- and age-related profiles were obtained (regression methods; fractional polynomials).

**Results:**

Waveform-derived indexes were not equivalent; for a specific index, there were systematic and proportional differences associated with the recording site (e.g., carotid *vs.* radial) and technique (e.g., tonometry *vs.* oscillometry). The need for sex-, BH-, or age-specific RIs was dependent on the index and/or age considered. RIs were defined for each index considering differences between recording sites and techniques. Equations for waveform-derived indexes age-related profiles were included, enabling to determine for a specific subject, the expected values and potential data deviations.

## Introduction

Central aortic blood pressure (aoBP) waveform contains valuable (e.g., prognostic) information beyond and in addition to the obtained from its corresponding systolic, diastolic, and pulse pressure levels (aoSBP, aoDBP, aoPP) (Mynard et al., 2020). Several techniques (e.g., applanation tonometry, oscillometry/plethysmography) and mathematical methods (e.g., direct carotid or distal-arteries recordings associated to a general transfer function) have been proposed to perform waveform analyses (Hametner and Wassertheurer, 2017). In addition, different pressure-only approaches for waveform analysis [e.g., pulse wave analysis (PWA), wave separation analysis (WSA)] are available (Westerhof et al., 2006; Wang et al., 2010; Chirinos et al., 2012; Weber et al., 2012; Zamani et al., 2014; Hametner et al., 2015; Sluyter et al., 2017; Mynard et al., 2020). Augmentation pressure (AP), augmentation index (AIx), and AIx corrected for heart rate (AIx@75) are the PWA-derived indexes most commonly used. The concept or basic idea underlying PWA is that forward waves traveling from the ventricle toward the periphery are distally reflected. Reflected waves augment (central) pressure. AP represents the augmentation level (a positive AP indicates “additional” pressure arising from reflections) (Baksi et al., 2009; Sugawara et al., 2010). It is calculated from the inflection point in the pressure waveform (systolic phase) that “signalizes or identifies” the reflected component’s arrival to the aortic root (Kelly et al., 1989). AIx, calculated as AP/aoPP, is considered as a surrogate index of wave reflection (although it is known that it also depends on factors like heart rate or ventricle function) (Hametner and Wassertheurer, 2017). In WSA, the aoBP waveform is decomposed into single forward (Pf) and backward (Pb) components, which actually integrate different forward and backward propagating waves. Furthermore, it is to note that the Pf represents the integration of forward wave arising from the ventricle and re-reflections of backward propagating waves at the ventricular-aorta interface. From Pf and Pb, the reflection magnitude (RM; RM = Pb/Pf) and index {RIx; RIx = [Pb/(Pf + Pb)]} were determined. However, taking into account the above stated (and despite the names), they cannot be considered as simple measures or indicators of the reflections (Westerhof and Westerhof, 2013; Zamani et al., 2014).

In the last decade, several clinical studies have shown that waveform analysis could provide valuable information (even exceeding the information obtained from the analysis of the exposure to cardiovascular risk factors [CRFs]) (Zamani et al., 2014; Hametner and Wassertheurer, 2017; Mynard et al., 2020). However, to optimize their value and to ensure a proper use of waveform-derived indexes, some issues should be assessed and clarified. First, it is unknown whether the different techniques and methods used to quantify waveform-derived indexes provide equivalent information that would allow the use of similar normative data (reference intervals, [RIs]) regardless of the approach considered. Second, there is limited information concerning age and/or sex-related RIs for PWA- and WSA-derived indexes obtained at the same time in large healthy populations (including children, adolescents, and adults). This is true, even more so if data from South American populations are considered. In this regard, it is to note that ethnicity may be an independent determinant of wave reflections (both in adults and children) (Chirinos et al., 2011; Heffernan et al., 2020). In addition, an accurate use of waveform-derived indexes requires knowing the expected physiological age-related profiles and the predicted value for a specific subject. However, in our knowledge, there are no works assessing waveform-derived indexes’ variations (as a continuous) considering data from different age-stages and their transitions (childhood-adolescence-adulthood). It is to note that studies that aimed at analyzing age-related differences do not allow for their adequate and comprehensive characterization, as they (i) considered small numbers of subjects [e.g., *n* = 65 (Hughes et al., 2013), *n* = 267 (Yu et al., 2020)], (ii) did not exclude subjects with cardiovascular disease or exposed to CRFs [e.g., cardiology outpatients (Namasivayam et al., 2016), unhealthy or diseased subjects (Torjesen et al., 2014; Hodson et al., 2016; Li et al., 2019), and subjects with CRFs (Mitchell et al., 2004; Hughes et al., 2013; Hickson et al., 2016; Wilenius et al., 2016; Gómez-Sánchez et al., 2020; Yu et al., 2020)]; (iii) compared “mean values” of groups comprising subjects of wide age ranges (e.g., 5–7 years (y) (Segers et al., 2007; Janner et al., 2010; Hodson et al., 2016) or 10 y (Kelly et al., 1989; Mitchell et al., 2004; McEniery et al., 2005; Torjesen et al., 2014; Hickson et al., 2016; Namasivayam et al., 2016; Solanki et al., 2018; Li et al., 2019; Yu et al., 2020) of difference in the age of subjects belonging to the same group); (iv) considered only adults within a limited age range (e.g., 40–70 y, grouped by decades) (Li et al., 2019), and (v) in general, did not consider subjects under 18–20 years of age. In this context, it should be noted that the need for RIs for vascular parameters in children and adolescents is now well-recognized and is considered necessary to extend their use in clinical practice (Climie et al., 2021).

In this context, this work’s aims were: (1) to evaluate the association and/or agreement between PWA- and/or WSA-derived indexes obtained with different techniques and approaches, (2) to determine the need for sex-, body height (BH)-, and/or age-specific RIs, (3) to define RIs for PWA- and WSA-derived indexes in a large cohort of healthy children, adolescents, and adults from South America. As a secondary aim, non-linear equations for sex-, BH-, and/or age-specific percentiles were determined and included (text and spreadsheet formats); the information given enables to determine the expected value for a particular subject (and to assess any possible deviation from the anticipated).

## Materials and Methods

### Study Population and Clinical and Anthropometric Evaluation

The study was carried out in the context of the CUiiDARTE project (Bia et al., 2011; Santana et al., 2012a,b; Zócalo et al., 2020; Bia and Zócalo, 2021), a population-based study developed in Uruguay. In this work, we considered data from 3619 subjects included in CUiiDARTE database. This contains data on demographic and anthropometric variables, exposure to CRFs, personal and family history of cardiovascular disease and data on hemodynamic, and on structural and functional vascular parameters (Bia et al., 2011; Santana et al., 2012a,b; Zócalo et al., 2020; Bia and Zócalo, 2021; Zócalo and Bia, 2021a,b). In this work, the analysis was focused on PWA- and WSA-derived indexes.

All procedures agree with the Declaration of Helsinki (1975 and reviewed in 1983). The study protocol was reviewed and approved by the Ethics Committee of Centro Hospitalario Pereira Rossell, Universidad de la República. Prior to the evaluation, the participants provided their written informed consent to participate in the study. In subjects under 18 y, parents’ written consent and children’s assent were obtained before the evaluations. Subjects or parents (in case of subjects aged < 18 y) provided informed written consent to have data from their medical records used in research.

Before cardiovascular evaluation, a brief clinical interview together with the anthropometric and blood test results evaluation enabled to assess exposure to CRFs. Body weight and BH were measured with the participant wearing light clothing and no shoes. Standing BH was measured using a portable stadiometer and recorded to the nearest 0.1 cm. Body weight was measured with an electronic scale (841/843, Seca Inc., Hamburg, Germany; model HBF-514C, Omron Inc., Chicago, IL, United States) and recorded to the nearest 0.1 kg. Body mass index (BMI) was calculated as body weight-to-squared BH ratio. In children and adolescents, z-scores for BMI were calculated using the WHO software (Anthro-v.3.2.2; Anthro-Plus-v.1.0.4) (Castro et al., 2019).

Obesity was defined as z-score for BMI ≥ 2.0 (for subjects < 18 y) or BMI > 30 Kg/m^2^ (for subjects ≥ 18 y). Arterial hypertension was considered to be present, if it had been previously diagnosed in agreement with reference guidelines and/or use of blood pressure-lowering drugs was reported. Cut-off values were: brachial systolic blood pressure (baSBP) ≥ 140 mmHg and/or diastolic blood pressure (baDBP) ≥ 90 mmHg (for subjects ≥ 18 y) and baSBP and baDBP > 95th percentile for sex, age, and BH (for subjects < 18 y). Personal and family histories of cardiovascular disease (i.e., presence of cerebral, coronary, aortic, or peripheral arterial disease) were assessed. A family history of cardiovascular disease was defined by the presence of first-degree (for all the subjects) and/or second-degree (for subjects ≤ 18 y) relatives with early (< 55 y in males, < 65 y in females) cardiovascular disease. History of dyslipidemia and diabetes were considered to be present if they had been previously diagnosed in agreement with reference guidelines and/or the use of lipid- or glucose-lowering drugs (respectively) was reported. Dyslipidemia was defined as total cholesterol > 240 mg/dL or high-density lipoprotein cholesterol for men < 40 mg/dL and for women < 46 mg/dL. In turn, diabetes diagnosis was based on plasma glucose levels (fasting plasma glucose ≥ 126 mg/dl). Regular (current) smokers, defined as usually smoking at least one cigarette/week, were identified.

### Cardiovascular Evaluation

The participants were asked to avoid exercise, tobacco, alcohol, caffeine, and food-intake 4 h before the evaluation. All hemodynamic measurements were performed in a temperature-controlled environment (21–23°C), with the subject in supine position and after resting for at least 10–15 min, which enabled reaching steady hemodynamic conditions. Using a validated oscillometric device (HEM-433INT; Omron Healthcare Inc., IL, United States), heart rate, baSBP, and baDBP were recorded in supine position simultaneously and/or immediately before or after each non-invasive arterial recording. Then, brachial artery pulse pressure (baPP; baPP = baSBP-baDBP) and mean BP (baMBP, baMBP = baDBP + baPP/3) were calculated.

### Structural and Functional Markers of Subclinical Atherosclerosis

Left and right common, internal, and external carotid arteries, vertebral artery, common femoral artery, and left brachial artery were examined (B-Mode and Doppler ultrasound, 7–13 MHz, linear transducer, M-Turbo, SonoSite Inc., Bothell, WA, United States). Transverse and longitudinal arterial views were obtained to assess the presence of atherosclerotic plaques (defined as focal wall thickening at least 50% greater than the adjacent segment, focal thickening protruding ≥0.5 mm into the lumen, or an intima-media thickness ≥1.5 mm) (Zócalo and Bia, 2016; Marin et al., 2020).

Left and right brachial and tibial systolic and diastolic blood pressure levels were obtained (no fixed order) at 5 min intervals (Hem-4030, Omron Inc., IL, United States). At least five measurements were obtained from each recording site. The Ankle Brachial Index, an index of arterial permeability and central-peripheral blood pressure amplification, was calculated as tibial systolic blood pressure/baSBP (Zócalo and Bia, 2016). Right and left Ankle-Brachial Index values < 0.9 were used to define and rule out stenosis of at least 50% distal to common femoral artery (Zócalo and Bia, 2016). After applying the exclusion criteria related to exposure to CRFs, there were no subjects with Ankle-Brachial Index < 0.9 in the group of RIs.

### Central Blood Pressure Levels and Waveform Analysis-Derived Indexes

Central aoBP levels and waveforms were obtained (random order) using commercially available devices: SphygmoCor-CvMS ([SCOR]; v.9, AtCor-Medical, Sydney, NSW, Australia) and Mobil-O-Graph PWA-monitor system ([MOG]; I.E.M.-GmbH, Stolberg, Germany).

**FIGURE 1:**
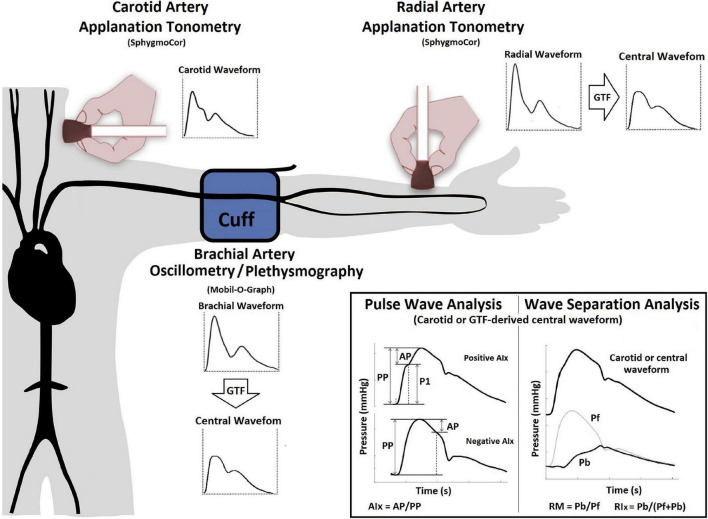
Instrumental approach employed to obtain central blood pressure waves and waveform-derived indexes. Examples of positive and negative AIx. Aix, Augmentation index; AP, augmentation pressure; GTF, general transfer function; Pf and Pb, forward and backward central blood pressure components; PP, pulse pressure; SphygmoCor, SphygmoCor device; v.9, AtCor-Medical, Sydney, NSW, Australia. Mobil-O-Graph, Mobil-O-Graph PWA-monitor system, I.E.M.-GmbH, Stolberg, Germany.

Using SCOR, aoBP waveforms were derived from, (i) radial artery (applying a general transfer function) and (ii) carotid artery (directly) manual tonometric recordings (Figure 1). Carotid pulse waveforms were assumed to be identical to the aortic ones (due to the proximity of the arterial sites) (Karamanoglu and Feneley, 1996). Thus, a transfer function was not applied to obtain central waveforms from carotid recordings. Only accurate waveforms on visual inspection and high-quality recordings (in-device quality control (operator) index > 75%) were considered. The operator index is an indicator of recorded signals’ overall reproducibility. It is calculated by assesing weighted quality control parameters and adding them to give a number as a percentage. Quality index assessment includes: (i) average height of individual records above 100 units, (ii) pulse height variation (accepted values: <5%), (iii) diastolic variation (indicates constancy of the basal level of the wave, accepted value: <5%), (iv) shape variation (difference in the shape of the recorded waves, accepted values <5%), and (v) maximum dP/dt (maximum value of the first derivative or maximal rate of wave rise). Based on the described variables, the operator index (recordings quality/reproducibility) was automatically assessed by the recording device. Brachial artery blood pressure levels and waveforms were automatically obtained using the MOG system (brachial cuff-based oscillometric device) (Zinoveev et al., 2019). The device determined aoBP levels and waveforms from peripheral recordings using a validated general transfer function. Only high-quality records (index equal to 1 or 2) and satisfactory waveforms (visual inspection) were considered.

Both devices (SCOR and MOG) enable PWA and WSA. A detailed (step-by-step) explanation of the method used for WSA based on recorded (carotid waveforms, SCOR) and mathematically derived aortic waveforms (SCOR and MOG) was included as Supplementary Material in a previous work (Zinoveev et al., 2019). As was previously published, absolute and relative intra- (repeatability) and interobserver (reproducibility) variability of aoBP levels and waveform-derived indices were analyzed (considering different methodological approaches: radial tonometry, carotid tonometry, and brachial oscillometric records) (Zinoveev et al., 2019). No significant differences were observed in aoSBP, aoPP, and waveform derived-indexes levels either within each visit, between two records [obtained by a single investigator (Y.Z.)], or between records obtained by two investigators (Y.Z., D.B.); indicating adequate repeatability, as well as reproducibility. In all cases, relative inter- and intraobserver variability was <6%.

Using both devices (SCOR and MOG) and the three recording sites (carotid and radial with SCOR and brachial with MOG), we quantified: (i) AP, AIx, AIx@75 and (ii) Pf, Pb, RM, and RIx (Figure 1). Recorded waveforms were calibrated using baDBP and baMBP (Zinoveev et al., 2019). AIx, AIx@75, RM, and RIx are “attractive” biomarkers because they are dimensionless and, therefore, do not depend on waveform calibration.

The variables were named based on the (i) waveform-derived index, (ii) recording site, and (iii) device (e.g., Pf_Radial_SCOR *vs.* Pf_Carotid_SCOR *vs.* Pf_Brachial_MOG).

### Data Analysis

**FIGURE 2:**
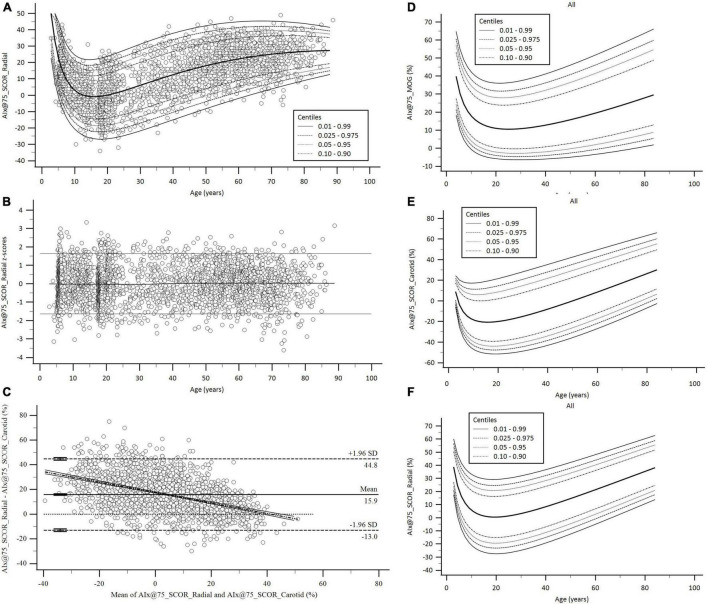
Age-related profiles and Bland–Altman analysis for AIx@75. **(A,B)** Age-related profiles (1th, 2.5th, 5th, 10th, 50th, 90th, 95th, 97.5th, and 99th percentiles) for AIx@75_SCOR_Radial and z-score diagram used to verify model fit. **(C)** Bland–Altman diagram for AIx@75_SCOR_Radial and AIx@75_SCOR_Carotid comparison. There were significant mean (15.9, 95% CI.; 15.28 to 16.53%, *p* < 0.001) and proportional errors (slope coefficient: –0.4234, 95% CI.: –0.4571 to –0.3897, *p* < 0.001). **(D–F)** Age-related percentile curves for AIx@75 obtained with three different methods. Age-related percentile curves (1th, 2.5th, 5th, 10th, 50th, 90th, 95th, 97.5th, and 99th percentiles) for AIx@75_MOG, AIx@75_SCOR_Radial, and AIx@75_SCOR_Carotid.

A step-wise analysis was performed. First, after descriptive statistics were computed and checked (Tables 1, 2 and Supplementary Tables 1, 2), it was analyzed whether the studied variables showed the expected trend in terms of age-related variations. Figure 2 exemplifies the results obtained for AIx@75_SCOR_Radial.

**TABLE 1:** Subjects demographic, anthropometric, and clinical characteristics.

	All (*n* = 3619)							Reference Intervals (*n* = 1688)						
Variable	MV	SD	Min	p25th	p50th	p75th	Max	MV	SD	Min	p25th	p50th	p75th	Max
Age [years]	33.87	24.20	2.80	11.50	23.65	56.40	89.00	20.12	16.93	2.80	6.29	17.60	21.80	84.20
Body weight [Kg.]	61.13	25.28	12.30	45.60	63.20	78.10	150.60	47.91	22.83	12.30	22.60	52.80	65.20	105.00
Body height [m]	1.55	0.23	0.90	1.46	1.62	1.71	1.97	1.47	0.26	0.90	1.17	1.58	1.69	1.94
BMI [Kg./m^2^]	24.06	6.02	11.53	19.70	23.63	27.84	71.34	20.36	4.22	11.53	16.59	20.00	23.56	29.95
z-BMI [SD]	0.94	1.45	−4.63	−0.05	0.74	1.77	8.03	0.34	0.92	−4.63	−0.27	0.41	1.00	1.98
TC [mg/dl]	200.26	44.27	94.30	170.00	196.00	227.00	379.00	194.87	25.85	99.00	179.00	198.00	214.00	238.00
HDL [mg/dl]	51.18	15.09	17.00	41.00	49.00	60.00	122.00	57.73	12.03	41.00	49.00	55.00	64.00	100.00
LDL [mg/dl]	123.39	39.83	28.00	95.00	119.00	146.00	323.00	117.96	25.51	31.00	101.00	120.60	134.00	180.00
Triglycerides [mg/dl]	133.21	85.94	24.00	80.00	111.00	158.00	783.00	93.30	38.85	24.00	65.00	86.00	113.00	272.00
Glicaemia [mg/dl]	94.35	18.68	40.00	85.00	92.00	100.00	307.00	88.23	9.48	40.00	83.00	88.00	93.00	121.00
baSBP [mmHg]	119.02	16.81	64.25	107.00	118.44	128.76	235.00	112.02	13.35	80.00	101.35	111.63	121.25	171.00
baDBP [mmHg]	68.84	10.36	41.25	60.78	67.73	75.80	129.18	64.79	8.36	46.70	58.92	63.29	70.00	97.44
TC ≥ 240 mg/dl [%]	7.2							0						
HDL < 40 mg/dl [%]	8.9							0						
Glicamia ≥ 126 mg/dl [%]	0.9							0						
Current Smoke [%]	11.4							0						
Hypertension [%]	26.4							0						
Diabetes [%]	5.7							0						
History of CVD [%]	8.8							0						
Obesity [%]	21.9							0						
Familiar CVD [%]	13.5							7.6						
Antihypertensive drugs	21.7							0						
Antihyperlipidemic agent [%]	13.5							0						
Antidiabetic agents [%]	4.1							0						
Atherosclerotic plaques (%)	22.2							6.6						

*MV, mean value; Min. and Max., minimal and maximal value. p25th, p50th (median), and p75th: 25, 50 and 75 percentiles; BMI, body mass index; baSBP, baDBP, brachial artery systolic and diastolic blood pressure; CVD, cardiovascular disease; TC, total cholesterol; HDL, HDL Cholesterol; LDL, LDL Cholesterol; Familiar CVD, Familiar history of premature CVD [%].*

**TABLE 2:** Central and peripheral blood pressure and central waveform-derived indexes.

	All (*n* = 3619)							Reference Intervals (*n* = 1688)						
Variable	MV	SD	Min	p25th	p50th	p75th	Max	MV	SD	Min	p25th	p50th	p75th	Max
**Brachial Artery Oscillometry/Plethysmography (MOG device)**														
baSBP [mmHg]	116.66	14.20	81.00	107.00	114.55	125.00	199.27	111.85	11.68	81.00	103.75	110.50	120.07	157.67
baDBP [mmHg]	68.31	11.25	36.00	60.40	66.87	74.75	131.43	64.76	8.89	38.67	58.40	63.71	70.25	106.00
aoSBP [mmHg]	104.89	16.99	71.00	92.00	103.78	115.63	185.09	99.13	14.93	71.00	86.73	97.50	108.53	168.50
aoDBP [mmHg]	69.63	11.25	38.00	61.83	68.47	76.00	133.14	66.08	8.93	39.33	59.50	65.00	71.61	109.00
HR [b.p.m]	75.68	15.47	33.40	64.00	73.47	85.67	135.25	78.60	16.29	41.00	66.75	76.75	88.83	135.25
AP [mmHg]	7.22	5.38	1.00	3.76	5.39	8.63	37.60	5.93	3.77	1.00	3.62	5.00	7.00	32.00
AIx [%]	19.33	10.49	−7.00	11.57	16.74	24.39	60.82	17.55	8.41	−7.00	11.24	16.17	22.36	60.00
AIx@75 [%]	19.67	11.95	−7.33	10.42	19.00	28.33	65.00	19.57	12.31	−7.00	10.00	19.15	28.42	65.00
Pf [mmHg]	23.37	7.54	10.30	18.13	21.94	26.80	65.55	22.27	7.68	10.30	17.06	20.60	25.25	65.55
Pb [mmHg]	13.91	5.17	4.20	10.13	13.10	16.58	38.15	12.79	5.10	4.20	9.15	11.65	15.23	38.15
RM	0.59	0.1	0.18	0.53	0.6	0.66	0.81	0.57	0.09	0.18	0.52	0.58	0.63	0.8
RIx	0.37	0.04	0.15	0.35	0.37	0.4	0.45	0.36	0.04	0.15	0.34	0.37	0.39	0.44

**Radial Artery Applanation Tonometry (SCOR device)**														
baSBP [mmHg]	120	16.48	77	109	120	130	235	114.24	13.55	78	105	114	124	160
baDBP [mmHg]	69	10.86	37	61	69	76	130	65.17	9.29	42	59	64	71	95
aoSBP [mmHg]	105	16.3	64	94	105	115	208	98.57	13.35	64	88	99	108	140
aoDBP [mmHg]	70	10.96	17	62	70	77	131	66.47	9.27	43	60	65	73	97
HR [b.p.m]	72	14.05	35	63	71	82	151	75.56	15.32	38	65	74	85	151
AP [mmHg]	4.93	6.76	−21	1	4	9	50	2.2	4.87	−21	−1	2	5	26
AIx [%]	13.2	15.3	−36	2	13	25	54	7.71	13.57	−36	−2	7	17	46
AIx@75 [%]	12.08	14.56	−37	1	13	23	49	7.78	14.27	−37	−3	8	18	49
Pf [mmHg]	29.13	9.13	7	23	28	34	89	28.57	9.66	7	21	27	34	71
Pb [mmHg]	15.47	5.75	3	12	14	18	78	13.45	4.31	3	11	13	16	78
RM	0.55	0.17	0.17	0.42	0.52	0.65	2.05	0.49	0.15	0.17	0.4	0.47	0.56	2.05
RIx	0.35	0.07	0.14	0.3	0.34	0.4	0.67	0.33	0.06	0.14	0.28	0.32	0.36	0.67

**Carotid Artery Applanation Tonometry (SCOR device)**														
baSBP [mmHg]	120.94	17.55	78	109	120	131	239	114.35	15.29	78	104	114	124	217
baDBP [mmHg]	68.84	10.98	38	60	68	76	127	64.47	9.31	38	58	63	70	100
aoSBP [mmHg]	112.6	18.17	69	100	111	124	216	105.48	16	69	94	105	116	216
aoDBP [mmHg]	68.84	10.98	38	60	68	76	127	64.47	9.31	38	58	63	70	100
HR [b.p.m]	71.75	14.15	32	61	70	80	145	75.11	15.44	40	64	74	84	145
AP [mmHg]	−1	11.69	−64	−8	−2	7	49	−5.38	9.6	−64	−10	−5	0	25
AIx [%]	−2.51	22.95	−63	−21	−5	17	56	−11.82	19.02	−55	−25	−14	0	44
AIx@75 [%]	−4.02	20.91	−60	−20	−5	12	53	−11.81	18.04	−56	−24	−12	−1	43
Pf [mmHg]	39.78	13.26	8	31	37	47	114	40.06	13.83	8	30	38	47	114
Pb [mmHg]	18.75	6.89	5	14	18	23	53	16.03	5.42	5	12	15	19	39
RM	0.5	0.18	0.11	0.36	0.47	0.63	1	0.43	0.15	0.11	0.33	0.41	0.5	0.97
RIx	0.32	0.08	0.1	0.27	0.32	0.38	0.5	0.29	0.07	0.1	0.25	0.29	0.33	0.49

*MV, mean value; Min. and Max., minimal and maximal value; p25th, p50th (median) and p75th, 25, 50 and 75 percentiles; BMI, body mass index; HR, heart rate; baSBP, baDBP, brachial systolic and diastolic pressure; aoSBP, aoDBP, aortic systolic and diastolic pressure; CVD, cardiovascular disease; AP, Augmentation Pressure; AIx, Augmentation Index. AIx@75, AIx adjusted by HR equal 75 beats/minute (b.p.m.); Pf, forward pressure; Pb, backward pressure; RM and RIx, reflection magnitude and index, respectively.*

Second, it was defined as a reference subgroup to determine RIs. This subgroup included subjects (*n* = 1688, 864 females) without any of the following (Engelen et al., 2013, 2015; Bossuyt et al., 2015): (i) cardiovascular disease; (ii) use of blood pressure-, lipid-, and/or glucose-lowering drugs; (iii) arterial hypertension; (iv) tobacco use; (v) diabetes; (vi) dyslipidemia; and (vii) obesity. In this subgroup, the atherosclerotic plaques presence was not associated with the waveform-derived indexes. Then, the subjects with plaques were not excluded from the RIs subgroup (Supplementary Table 3).

Third, in order to determine whether specific RIs were necessary for the same waveform-derived parameter obtained with different approaches (e.g., Pf_Radial_SCOR *vs.* Pf_Carotid_SCOR *vs.* Pf_Brachial_MOG), we analyzed the degree of association and agreement between data by assessing: (i) concordance correlation coefficients and (ii) mean and proportional differences (Bland–Altman analysis) (Table 3 and Supplementary Table 4). Figure 2 exemplifies the results obtained when comparing AIx@75_SCOR_Radial and AIx@75_SCOR_Carotid. Specific RIs for all waveform-derived indexes obtained with the different approaches were defined as necessary.

**TABLE 3:** Agreement between central waveform-derived indexes obtained with carotid and radial applanation tonometry and brachial oscillometry: Concordance Correlation and Bland–Altman analysis.

Method A	MOG_Brachial	MOG_Brachial	SCOR_Radial	MOG_Brachial	MOG_Brachial	SCOR_Radial

Method B	SCOR_Radial	SCOR_Carotid	SCOR_Carotid	SCOR_Radial	SCOR_Carotid	SCOR_Carotid
		
	Augmentation Pressure (AP; mmHg)			AIx@75 (%)		
**Concordance Correlation Coefficient**						
CCC	0.4246	0.2139	0.5521	0.3316	0.1163	0.4771
95% C.I. CCC	0.38 to 0.46	0.18 to 0.24	0.53 to 0.57	0.29 to 0.37	0.09 to 0.13	0.45 to 0.49
Pearson ρ (precision)	0.5198	0.4587	0.7675	0.4218	0.3184	0.709
Bias correction factor (accuracy)	0.8169	0.4662	0.7193	0.7862	0.3653	0.6729
**Bland–Altman**						
Mean Error (Method A–B)	3.8533	10.5921	5.9383	8.8828	26.7975	15.9068
Mean Error, 95% C.I.	3.53 to 4.17	9.99 to 11.18	5.60 to 6.26	8.07 to 9.68	25.63 to 27.96	15.28 to 16.53
P (H_0_: Mean Error = 0)	<1.0E-14	<1.0E-14	<1.0E-14	<1.0E-14	<1.0E-14	<1.0E-14
Lower limit, Mean Error	−7.3202	−9.352	−9.4158	−18.8699	−12.0174	−13.0218
Upper limit, Mean Error	15.0267	30.5362	21.2924	36.6355	65.6124	44.8354
Regression Equation	y = 4.42-0.10×	y = 12.55-0.90×	y = 7.15-0.61×	y = 13.82-0.34×	y = 31.07-0.82×	y = 17.58-0.42×
Intercept, *p* value	<1.0E-14	<1.0E-14	<1.0E-14	<1.0E-14	<1.0E-14	<1.0E-14
Slope, *p* value	0.0014	<1.0E-14	<1.0E-14	<1.0E-14	<1.0E-14	<1.0E-14

	**Forward pressure (Pf; mmHg)**			**Backward pressure (Pb; mmHg)**		
**Concordance Correlation Coefficient**						
CCC	0.2859	0.08184	0.367	0.4306	0.3032	0.6187
95% C.I. CCC	0.24 to 0.32	0.05 to 0.10	0.34 to 0.39	0.38 to 0.47	0.25 to 0.35	0.59 to 0.64
Pearson ρ (precision)	0.3501	0.2197	0.572	0.4329	0.3688	0.7155
Bias correction factor (accuracy)	0.8166	0.3725	0.6417	0.9947	0.8222	0.8647
**Bland–Altman**						
Mean Error (Method A–B)	−5.3221	−17.7235	−10.5389	−0.113	−3.5821	−2.9946
Mean Error, 95% C.I.	−5.88 to −4.76	−18.62 to −16.81	−11.03 to −10.04	−0.41 to 0.18	−4.01 to −3.14	−3.21 to −2.77
P (H_0_: Mean Error = 0)	<1.0E-14	<1.0E-14	<1.0E-14	0.4614	<1.0E-14	<1.0E-14
Lower limit, Mean Error	−24.5978	−45.6596	−31.9075	−10.4926	−17.0728	−12.4717
Upper limit, Mean Error	13.9536	10.2126	10.8296	10.2665	9.9087	6.4826
Regression Equation	y = 3.52-0.33×	y = 12.00-0.90×	y = 6.64-0.49×	y = −2.11 + 0.14×	y = 2.66-0.38×	y = 2.21-0.30×
Intercept, *p* value	0.0011	<1.0E-14	<1.0E-14	0.0001	0.000291	7.39E-11
Slope, *p* value	<1.0E-14	<1.0E-14	<1.0E-14	0.000124	<1.0E-14	<1.0E-14

	**Reflection Magnitude (RM; Pb/Pf)**			**Reflection Index {RIx; [Pb/(Pf + Pb)]}**		
**Concordance Correlation Coefficient**						
CCC	0.307	0.2321	0.6592	0.2759	0.2016	0.6286
95% C.I. CCC	0.26 to 0.34	0.19 to 0.26	0.63 to 0.68	0.23 to 0.31	0.17 to 0.23	0.60 to 0.65
Pearson ρ (precision)	0.413	0.4322	0.7014	0.3872	0.4164	0.6852
Bias correction factor (accuracy)	0.7432	0.537	0.9397	0.7125	0.484	0.9175
**Bland–Altman**						
Mean Error (Method A–B)	0.08005	0.148	0.06211	0.03757	0.06985	0.02883
Mean Error, 95% C.I.	0.071 to 0.088	0.13 to 0.15	0.055 to 0.068	0.034 to 0.041	0.065 to 0.074	0.026to 0.031
P (H_0_: Mean Error = 0)	<1.0E-14	<1.0E-14	<1.0E-14	<1.0E-14	<1.0E-14	<1.0E-14
Lower limit, Mean Error	−0.2138	−0.1578	−0.2067	−0.08566	−0.06858	−0.08882
Upper limit, Mean Error	0.3739	0.4539	0.331	0.1608	0.2083	0.1465
Regression Equation (y=)	0.472-0.701×	0.566-0.785×	0.105-0.0811×	0.287-0.707×	0.376-0.899×	0.095-0.198×
Intercept, *p* value	<1.0E-14	<1.0E-14	<1.0E-14	<1.0E-14	<1.0E-14	<1.0E-14
Slope, *p* value	<1.0E-14	<1.0E-14	0.0000321	<1.0E-14	<1.0E-14	<1.0E-14

*CCC, Concordance correlation coefficient; SCOR, SphygmoCor device; MOG, Mobil-O-Graph device; Bland–Altman test: (y: Method A–Method B; x: Mean of both Methods). AIx@75, Augmentation Index adjusted by HR equal 75 beats/minute.*

Fourth, we evaluated whether age-, BH-, and/or sex-specific RIs were necessary using multiple linear regression models that included interaction analysis (Sex*Age; Sex*BH) with Johnson-Neyman significance regions definition (Supplementary Tables 5–10). Variables “y,” “x,” and “w” (moderating variable) were assigned to the waveform-derived indexes (y), sex (x), and age or BH (w). We identified indexes that: (i) required sex-specific RIs only from a certain age or BH, (ii) required sex-specific RIs regardless of age or BH, (iii) did not require sex-specific RIs, (iv) did not require age-, BH-, and/or sex-specific RIs (Supplementary Table 11). To enable comparisons with other authors and, at the same time, to minimize type 1 error associated with the development of several multiple regression models (*n* = 42), even in the case that sex-variable was significant, we defined RIs for the whole group (males and females).

Fifth, age- and BH-related percentile curves and RIs were obtained. To obtain age- and BH-related equations for mean values and SD, we used parametric regression methods based on fractional polynomials (Royston and Wright, 1998; Díaz et al., 2018; Zócalo et al., 2020; Bia and Zócalo, 2021; Zócalo and Bia, 2021a,b). Briefly, fitting fractional polynomials, age-specific (and BH-specific) mean value and SD regression curves were defined for the different variables (e.g., AIx@75_SCOR_Radial) using an iterative procedure (generalized least squares). Then, age-specific (and BH-specific) equations were obtained for the different indexes. For instance, AIx@75_SCOR_Radial equation would be: “AIx@75_SCOR_Radial mean value = a + b*Age^p^ + c*Age^q^ + .,” where a, b, c, are the coefficients, and p and q are the powers, with numbers selected from the set [−2, −1, −0.5, 0, 0.5, 1, 2, 3] estimated from the regression for mean AIx@75_SCOR_Radial curve, and likewise from the regression for SD curve. Fractional polynomials with powers [1,2], that is, with p = 1 and q = 2, illustrate an equation with the form a + b*Age + c*Age^2^ (Royston and Wright, 1998). Residuals were used to assess the model fit, which was deemed appropriate if the scores were normally distributed, with a mean of 0 and a SD of 1, randomly scattered above and below 0 when plotted against age (or BH). Best-fit curves, according to visual and mathematical criteria (Kurtosis and Skewness coefficients), were selected. From the equations obtained, age- and BH-specific percentiles were defined using the standard normal distribution (Z) (Supplementary Tables 12, 13).

Detailed age- and BH-related RIs and percentile curves data can be found in Supplementary Tables 14–76 (for age) and 77–139 (for BH) and in Supplementary Material 2 (Supplementary Figures 1–21; for age) and 3 (Supplementary Figures 22–42; for BH). Figure 2 exemplifies age-related percentile curves for AIx@75_MOG, AIx@75_SCOR_Radial and AIx@75_SCOR_Carotid. Table 4 shows a summary (5 y intervals) of the age-related RIs (p50th, p75th, p90th, p95th, p97.5th, p99th) for each waveform-derived index. Table 5 shows a summary (10 cm intervals) of BH-related RIs (p50th, p75th, p90th, p95th, p97.5th, p99th) for each waveform-derived index.

**TABLE 4:** Age-related reference intervals for central waveform-derived indexes (All: Females and Males).

	Brachial Oscillometry (MOG)						Radial Artery Tonometry (SCOR)						Carotid Artery Tonometry (SCOR)					
Age [y]	50th	75th	90th	95th	97.5th	99th	50th	75th	90th	95th	97.5th	99th	50th	75th	90th	95th	97.5th	99th
**Augmentation Pressure (AP; mmHg)**																		
3	4.51	5.83	7.42	8.62	9.85	11.56	5.93	6.96	7.89	8.45	8.93	9.50	0.18	1.86	3.35	4.23	5.00	5.88
5	4.41	5.90	7.78	9.23	10.77	12.94	2.79	4.40	5.86	6.75	7.52	8.42	−4.73	−1.72	0.94	2.51	3.86	5.41
10	4.55	6.36	8.76	10.71	12.84	15.97	0.29	2.54	4.60	5.84	6.93	8.21	−8.72	−4.16	−0.19	2.14	4.13	6.42
15	4.82	6.92	9.78	12.17	14.83	18.82	0.01	2.55	4.87	6.28	7.51	8.95	−9.05	−3.85	0.66	3.30	5.56	8.14
20	5.14	7.52	10.83	13.65	16.83	21.68	0.46	3.16	5.63	7.13	8.44	9.98	−8.14	−2.65	2.11	4.89	7.26	9.98
25	5.49	8.15	11.92	15.18	18.88	24.62	1.25	4.04	6.60	8.15	9.51	11.11	−6.68	−1.08	3.78	6.61	9.04	11.82
30	5.87	8.81	13.05	16.76	21.02	27.69	2.22	5.07	7.68	9.26	10.65	12.28	−4.95	0.66	5.53	8.38	10.81	13.60
35	6.27	9.51	14.24	18.41	23.25	30.90	3.29	6.18	8.81	10.42	11.82	13.46	−3.10	2.46	7.30	10.13	12.55	15.32
40	6.69	10.24	15.48	20.15	25.59	34.28	4.43	7.33	9.98	11.59	12.99	14.64	−1.19	4.29	9.06	11.85	14.24	16.98
45	7.13	11.01	16.78	21.97	28.07	37.86	5.61	8.51	11.16	12.76	14.16	15.81	0.74	6.11	10.79	13.54	15.89	18.58
50	7.59	11.81	18.15	23.89	30.68	41.66	6.82	9.71	12.34	13.94	15.33	16.97	2.66	7.91	12.49	15.18	17.48	20.13
55	8.08	12.66	19.59	25.92	33.44	45.69	8.04	10.91	13.52	15.11	16.49	18.11	4.57	9.69	14.16	16.78	19.03	21.62
60	8.59	13.55	21.11	28.05	36.36	49.97	9.27	12.11	14.70	16.27	17.64	19.24	6.45	11.43	15.78	18.35	20.54	23.06
65	9.13	14.48	22.71	30.32	39.46	54.53	10.50	13.31	15.87	17.42	18.78	20.36	8.31	13.14	17.37	19.87	22.00	24.46
70	9.69	15.46	24.40	32.71	42.75	59.38	11.74	14.51	17.04	18.57	19.90	21.46	10.13	14.82	18.93	21.35	23.43	25.82
75	10.28	16.50	26.18	35.24	46.24	64.56	12.97	15.71	18.20	19.70	21.01	22.55	11.92	16.46	20.45	22.80	24.82	27.14
80	10.90	17.58	28.07	37.92	49.94	70.07	14.21	16.90	19.34	20.82	22.11	23.62	13.67	18.07	21.93	24.21	26.17	28.42
84	11.42	18.49	29.65	40.18	53.07	74.74	15.19	17.85	20.26	21.71	22.98	24.47	15.05	19.33	23.09	25.32	27.23	29.43

**Augmentation Index (AIx; %)**																		
3	27.09	33.61	39.96	44.01	47.66	52.07	22.90	29.39	35.33	38.95	42.11	45.82	−8.86	−2.81	2.88	6.41	9.53	13.25
5	20.53	25.82	30.99	34.27	37.24	40.82	12.03	19.07	25.56	29.52	32.99	37.07	−16.25	−8.24	−0.53	4.32	8.67	13.89
10	15.21	19.73	24.16	26.99	29.55	32.65	2.95	10.49	17.48	21.76	25.53	29.97	−20.98	−11.00	−1.17	5.12	10.81	17.68
15	14.02	18.60	23.11	26.00	28.61	31.79	1.21	8.94	16.11	20.52	24.39	28.96	−20.49	−9.60	1.21	8.14	14.42	22.03
20	14.13	19.02	23.87	26.98	29.81	33.25	1.91	9.73	16.99	21.45	25.37	29.99	−18.32	−6.85	4.51	11.80	18.40	26.39
25	14.85	20.19	25.50	28.92	32.03	35.82	3.75	11.62	18.91	23.38	27.32	31.96	−15.33	−3.49	8.21	15.70	22.47	30.66
30	15.93	21.78	27.63	31.42	34.86	39.07	6.22	14.10	21.39	25.86	29.79	34.43	−11.87	0.23	12.12	19.70	26.55	34.82
35	17.23	23.67	30.12	34.30	38.11	42.77	9.07	16.94	24.22	28.67	32.58	37.19	−8.10	4.15	16.13	23.75	30.61	38.89
40	18.71	25.77	32.86	37.47	41.68	46.83	12.17	20.02	27.26	31.68	35.57	40.14	−4.09	8.23	20.21	27.81	34.63	42.86
45	20.32	28.03	35.81	40.87	45.50	51.16	15.45	23.25	30.44	34.83	38.67	43.20	0.09	12.41	24.33	31.86	38.62	46.74
50	22.03	30.43	38.93	44.46	49.53	55.74	18.85	26.60	33.72	38.06	41.87	46.34	4.41	16.67	28.48	35.91	42.56	50.55
55	23.83	32.95	42.19	48.22	53.75	60.52	22.34	30.01	37.06	41.35	45.11	49.53	8.85	21.00	32.63	39.94	46.47	54.28
60	25.70	35.57	45.59	52.13	58.13	65.48	25.89	33.49	40.45	44.68	48.40	52.75	13.38	25.37	36.80	43.95	50.33	57.96
65	27.65	38.28	49.10	56.16	62.65	70.62	29.49	37.00	43.86	48.04	51.70	55.99	18.00	29.78	40.96	47.94	54.16	61.57
70	29.66	41.08	52.71	60.32	67.32	75.90	33.13	40.53	47.30	51.42	55.02	59.24	22.70	34.23	45.13	51.91	57.95	65.13
75	31.72	43.95	56.43	64.60	72.11	81.33	36.79	44.08	50.75	54.80	58.34	62.49	27.45	38.70	49.29	55.87	61.70	68.64
80	33.83	46.89	60.23	68.97	77.01	86.89	40.46	47.65	54.20	58.18	61.66	65.74	32.27	43.20	53.45	59.80	65.43	72.11
84	35.56	49.29	63.34	72.55	81.02	91.43	43.41	50.50	56.97	60.89	64.31	68.33	36.16	46.81	56.77	62.93	68.39	74.85

**Augmentation Index adjusted by heart rate 75 beats/minute (AIx@75; %)**																		
3	48.03	55.03	61.52	65.50	69.00	73.14	35.65	41.99	47.68	51.11	54.08	57.54	6.08	11.03	15.54	18.27	20.67	23.47
5	32.39	39.21	45.58	49.52	53.00	57.12	20.36	27.48	33.88	37.73	41.08	44.97	−7.39	0.03	6.88	11.08	14.77	19.12
10	17.82	24.39	30.66	34.57	38.05	42.19	5.70	13.61	20.73	25.02	28.74	33.08	−18.53	−8.74	0.49	6.20	11.25	17.24
15	12.87	19.41	25.71	29.67	33.21	37.45	1.30	9.48	16.87	21.31	25.17	29.67	−20.73	−9.95	0.25	6.60	12.23	18.91
20	10.99	17.61	24.04	28.10	31.73	36.08	0.48	8.77	16.25	20.76	24.67	29.23	−20.00	−8.69	2.04	8.71	14.63	21.67
25	10.52	17.30	23.91	28.09	31.83	36.32	1.37	9.69	17.19	21.71	25.63	30.20	−17.90	−6.27	4.74	11.59	17.66	24.88
30	10.83	17.82	24.64	28.95	32.81	37.45	3.22	11.51	18.99	23.49	27.40	31.96	−15.03	−3.22	7.94	14.87	21.01	28.30
35	11.64	18.87	25.91	30.36	34.35	39.14	5.65	13.88	21.31	25.78	29.66	34.18	−11.67	0.22	11.43	18.37	24.52	31.82
40	12.79	20.28	27.56	32.16	36.28	41.21	8.44	16.60	23.96	28.38	32.22	36.70	−7.99	3.92	15.10	22.02	28.13	35.38
45	14.19	21.95	29.48	34.22	38.47	43.56	11.48	19.55	26.82	31.19	34.99	39.41	−4.07	7.78	18.88	25.74	31.79	38.96
50	15.79	23.82	31.60	36.49	40.87	46.12	14.69	22.65	29.83	34.14	37.89	42.26	0.02	11.77	22.75	29.51	35.47	42.53
55	17.55	25.86	33.88	38.92	43.43	48.83	18.01	25.87	32.94	37.19	40.88	45.18	4.24	15.85	26.66	33.30	39.16	46.09
60	19.44	28.02	36.29	41.48	46.12	51.67	21.41	29.15	36.11	40.30	43.93	48.17	8.57	19.99	30.60	37.11	42.85	49.62
65	21.44	30.29	38.81	44.14	48.91	54.60	24.86	32.47	39.33	43.45	47.02	51.19	12.97	24.17	34.56	40.92	46.52	53.13
70	23.53	32.66	41.41	46.89	51.78	57.62	28.35	35.83	42.57	46.62	50.13	54.23	17.43	28.39	38.52	44.73	50.18	56.61
75	25.70	35.10	44.09	49.71	54.73	60.71	31.85	39.21	45.83	49.81	53.26	57.28	21.94	32.63	42.50	48.52	53.82	60.06
80	27.94	37.60	46.83	52.59	57.73	63.86	35.37	42.59	49.09	53.00	56.39	60.33	26.49	36.89	46.47	52.31	57.44	63.48
84	29.78	39.65	49.06	54.94	60.17	66.41	38.18	45.30	51.70	55.55	58.89	62.78	30.15	40.30	49.64	55.33	60.32	66.19

**Forward pressure (Pf; mmHg)**																		
3	12.39	13.66	14.90	15.69	16.42	17.31	12.94	15.40	17.98	19.73	21.37	23.44	17.45	21.43	25.77	28.78	31.68	35.41
5	14.98	16.90	18.82	20.08	21.23	22.66	18.40	22.05	25.92	28.54	31.02	34.16	22.91	28.00	33.53	37.35	41.01	45.73
10	22.33	26.47	30.84	33.80	36.59	40.13	26.39	31.87	37.71	41.69	45.47	50.27	39.72	48.62	58.29	64.98	71.41	79.68
15	24.60	29.55	34.83	38.44	41.87	46.24	30.14	36.51	43.32	47.97	52.38	58.01	43.00	52.98	63.89	71.48	78.79	88.23
20	24.94	30.02	35.44	39.15	42.68	47.18	31.73	38.49	45.72	50.66	55.36	61.35	42.92	53.14	64.36	72.20	79.76	89.55
25	24.59	29.52	34.77	38.36	41.78	46.13	32.10	38.96	46.30	51.32	56.09	62.18	41.65	51.71	62.78	70.52	78.01	87.71
30	24.04	28.74	33.73	37.13	40.36	44.46	31.74	38.53	45.79	50.76	55.48	61.51	40.07	49.78	60.46	67.94	75.17	84.55
35	23.50	27.97	32.69	35.89	38.93	42.78	30.94	37.55	44.63	49.46	54.06	59.93	38.54	47.80	58.00	65.12	72.01	80.93
40	23.08	27.33	31.81	34.84	37.70	41.32	29.89	36.26	43.07	47.73	52.16	57.80	37.19	45.99	55.64	62.38	68.88	77.29
45	22.79	26.88	31.16	34.04	36.76	40.19	28.68	34.78	41.29	45.74	49.98	55.37	36.07	44.41	53.51	59.85	65.94	73.81
50	22.67	26.62	30.75	33.52	36.13	39.42	27.40	33.20	39.40	43.63	47.65	52.78	35.20	43.09	51.65	57.58	63.27	70.60
55	22.70	26.56	30.59	33.28	35.81	39.00	26.09	31.59	37.46	41.47	45.28	50.13	34.58	42.02	50.06	55.59	60.89	67.68
60	22.89	26.71	30.67	33.33	35.81	38.93	24.78	29.98	35.53	39.31	42.91	47.49	34.19	41.21	48.73	53.88	58.79	65.06
65	23.25	27.06	31.01	33.65	36.11	39.21	23.49	28.40	33.63	37.20	40.58	44.90	34.01	40.63	47.65	52.44	56.97	62.74
70	23.77	27.62	31.59	34.24	36.73	39.84	22.24	26.86	31.79	35.14	38.33	42.38	34.05	40.27	46.81	51.23	55.40	60.68
75	24.46	28.38	32.43	35.13	37.65	40.81	21.03	25.38	30.01	33.17	36.16	39.96	34.29	40.12	46.19	50.25	54.07	58.87
80	25.33	29.36	33.53	36.30	38.90	42.14	19.87	23.96	28.31	31.27	34.08	37.65	34.74	40.17	45.76	49.48	52.96	57.30
84	26.15	30.31	34.61	37.47	40.14	43.48	18.99	22.88	27.01	29.83	32.49	35.88	35.23	40.35	45.57	49.01	52.21	56.20

**Backward pressure (Pb; mmHg)**																		
3	6.67	7.46	8.24	8.75	9.22	9.80	6.97	8.03	9.12	9.84	10.52	11.36	8.17	9.91	11.78	13.06	14.29	15.87
5	7.99	9.20	10.44	11.26	12.03	12.99	8.25	9.56	10.92	11.82	12.66	13.72	9.47	11.42	13.52	14.95	16.32	18.07
10	12.05	14.75	17.70	19.74	21.69	24.22	11.96	14.03	16.20	17.65	19.02	20.74	13.53	16.25	19.16	21.14	23.02	25.43
15	13.76	17.08	20.73	23.29	25.76	28.97	13.02	15.31	17.71	19.32	20.84	22.76	15.01	18.08	21.37	23.62	25.76	28.49
20	14.47	17.94	21.77	24.44	27.02	30.37	13.53	15.91	18.40	20.08	21.66	23.65	15.89	19.20	22.75	25.19	27.51	30.49
25	14.74	18.17	21.91	24.52	27.02	30.27	13.84	16.26	18.80	20.51	22.11	24.13	16.50	19.99	23.74	26.32	28.78	31.93
30	14.84	18.13	21.69	24.16	26.52	29.56	14.09	16.54	19.10	20.82	22.44	24.48	17.00	20.62	24.52	27.20	29.77	33.05
35	14.88	18.00	21.36	23.66	25.87	28.68	14.36	16.83	19.41	21.15	22.78	24.83	17.45	21.17	25.19	27.96	30.60	33.99
40	14.91	17.88	21.03	23.18	25.23	27.84	14.67	17.17	19.78	21.53	23.17	25.23	17.88	21.70	25.81	28.64	31.35	34.81
45	14.99	17.80	20.77	22.79	24.69	27.11	15.04	17.58	20.22	21.99	23.65	25.74	18.34	22.23	26.42	29.29	32.05	35.57
50	15.11	17.80	20.61	22.50	24.29	26.54	15.48	18.07	20.75	22.55	24.24	26.36	18.82	22.77	27.02	29.94	32.72	36.29
55	15.30	17.88	20.55	22.35	24.03	26.14	16.00	18.65	21.39	23.23	24.95	27.11	19.34	23.35	27.64	30.59	33.40	36.99
60	15.56	18.04	20.61	22.33	23.93	25.93	16.61	19.33	22.15	24.03	25.79	28.00	19.90	23.96	28.29	31.26	34.08	37.69
65	15.89	18.31	20.79	22.44	23.98	25.90	17.31	20.12	23.02	24.96	26.77	29.04	20.52	24.62	28.98	31.96	34.79	38.40
70	16.30	18.67	21.10	22.70	24.19	26.04	18.12	21.02	24.03	26.03	27.90	30.25	21.19	25.32	29.71	32.69	35.52	39.12
75	16.79	19.14	21.52	23.10	24.55	26.36	19.03	22.06	25.18	27.26	29.20	31.63	21.93	26.08	30.48	33.46	36.28	39.86
80	17.36	19.71	22.08	23.64	25.08	26.86	20.06	23.22	26.48	28.65	30.67	33.20	22.73	26.90	31.30	34.27	37.07	40.62
84	17.89	20.25	22.63	24.18	25.62	27.40	20.98	24.26	27.64	29.89	31.98	34.61	23.42	27.60	31.99	34.95	37.74	41.26

**Reflection Magnitude (RM)**																		
3	0.54	0.59	0.63	0.66	0.68	0.70	0.65	0.73	0.80	0.85	0.89	0.95	0.42	0.49	0.56	0.61	0.65	0.70
5	0.54	0.59	0.64	0.66	0.68	0.70	0.53	0.59	0.67	0.71	0.76	0.81	0.38	0.45	0.52	0.56	0.60	0.66
10	0.56	0.61	0.65	0.67	0.69	0.72	0.44	0.50	0.57	0.62	0.67	0.72	0.35	0.42	0.50	0.54	0.59	0.64
15	0.58	0.62	0.67	0.69	0.71	0.73	0.42	0.49	0.56	0.61	0.66	0.72	0.36	0.43	0.51	0.56	0.61	0.66
20	0.59	0.64	0.68	0.70	0.72	0.74	0.42	0.50	0.58	0.63	0.68	0.74	0.37	0.45	0.53	0.59	0.63	0.70
25	0.61	0.65	0.69	0.72	0.73	0.76	0.44	0.52	0.60	0.66	0.71	0.78	0.39	0.48	0.56	0.62	0.67	0.73
30	0.62	0.67	0.71	0.73	0.75	0.77	0.46	0.54	0.63	0.69	0.75	0.83	0.42	0.51	0.59	0.65	0.71	0.78
35	0.63	0.68	0.72	0.74	0.76	0.78	0.49	0.57	0.67	0.74	0.80	0.88	0.44	0.54	0.63	0.69	0.75	0.82
40	0.64	0.69	0.73	0.75	0.77	0.79	0.51	0.61	0.71	0.78	0.85	0.94	0.47	0.57	0.67	0.73	0.79	0.86
45	0.66	0.70	0.74	0.76	0.78	0.80	0.55	0.65	0.76	0.84	0.91	1.01	0.50	0.60	0.70	0.77	0.83	0.91
50	0.67	0.71	0.75	0.77	0.79	0.81	0.58	0.69	0.81	0.90	0.98	1.08	0.53	0.64	0.74	0.81	0.88	0.96
55	0.68	0.72	0.76	0.78	0.80	0.82	0.62	0.74	0.87	0.96	1.05	1.16	0.56	0.67	0.78	0.86	0.93	1.01
60	0.69	0.73	0.77	0.79	0.80	0.82	0.67	0.79	0.93	1.03	1.12	1.24	0.59	0.71	0.83	0.90	0.97	1.06
65	0.69	0.74	0.77	0.79	0.81	0.83	0.71	0.85	1.00	1.10	1.20	1.34	0.62	0.75	0.87	0.95	1.02	1.11
70	0.70	0.74	0.78	0.80	0.82	0.84	0.76	0.91	1.07	1.18	1.29	1.43	0.66	0.78	0.91	0.99	1.07	1.16
75	0.71	0.75	0.79	0.81	0.83	0.85	0.82	0.98	1.15	1.27	1.39	1.54	0.69	0.82	0.95	1.04	1.12	1.22
80	0.72	0.76	0.80	0.82	0.83	0.85	0.87	1.05	1.23	1.36	1.49	1.65	0.73	0.86	1.00	1.09	1.17	1.27
84	0.73	0.77	0.80	0.82	0.84	0.86	0.92	1.11	1.30	1.44	1.58	1.75	0.76	0.90	1.04	1.13	1.21	1.32

**Reflection Index (RIx)**																		
3	0.35	0.37	0.39	0.40	0.40	0.41	0.39	0.42	0.44	0.46	0.47	0.49	0.30	0.33	0.36	0.38	0.39	0.41
5	0.35	0.37	0.39	0.40	0.41	0.41	0.34	0.37	0.40	0.42	0.43	0.45	0.27	0.31	0.34	0.36	0.38	0.40
10	0.36	0.38	0.39	0.40	0.41	0.42	0.30	0.34	0.36	0.38	0.40	0.42	0.26	0.30	0.33	0.35	0.37	0.39
15	0.37	0.38	0.40	0.41	0.42	0.42	0.30	0.33	0.36	0.38	0.40	0.42	0.26	0.30	0.34	0.36	0.38	0.40
20	0.37	0.39	0.40	0.41	0.42	0.43	0.30	0.33	0.37	0.39	0.40	0.43	0.27	0.31	0.35	0.37	0.39	0.41
25	0.38	0.40	0.41	0.42	0.42	0.43	0.31	0.34	0.37	0.40	0.42	0.44	0.28	0.32	0.36	0.38	0.40	0.42
30	0.38	0.40	0.41	0.42	0.43	0.44	0.32	0.35	0.39	0.41	0.43	0.45	0.29	0.34	0.37	0.40	0.42	0.44
35	0.39	0.40	0.42	0.43	0.43	0.44	0.33	0.36	0.40	0.42	0.44	0.47	0.31	0.35	0.39	0.41	0.43	0.45
40	0.39	0.41	0.42	0.43	0.44	0.44	0.34	0.38	0.42	0.44	0.46	0.49	0.32	0.36	0.40	0.42	0.44	0.47
45	0.40	0.41	0.42	0.43	0.44	0.45	0.35	0.39	0.43	0.46	0.48	0.50	0.33	0.37	0.41	0.44	0.46	0.48
50	0.40	0.42	0.43	0.44	0.44	0.45	0.37	0.41	0.45	0.47	0.50	0.52	0.34	0.39	0.43	0.45	0.47	0.50
55	0.40	0.42	0.43	0.44	0.44	0.45	0.38	0.43	0.47	0.49	0.51	0.54	0.36	0.40	0.44	0.46	0.49	0.51
60	0.41	0.42	0.43	0.44	0.45	0.45	0.40	0.44	0.48	0.51	0.53	0.56	0.37	0.41	0.45	0.48	0.50	0.52
65	0.41	0.42	0.44	0.44	0.45	0.46	0.42	0.46	0.50	0.53	0.55	0.58	0.38	0.43	0.47	0.49	0.51	0.54
70	0.41	0.43	0.44	0.45	0.45	0.46	0.43	0.48	0.52	0.55	0.57	0.60	0.40	0.44	0.48	0.50	0.53	0.55
75	0.42	0.43	0.44	0.45	0.45	0.46	0.45	0.50	0.54	0.57	0.59	0.62	0.41	0.45	0.49	0.52	0.54	0.56
80	0.42	0.43	0.44	0.45	0.46	0.46	0.47	0.51	0.56	0.59	0.61	0.64	0.42	0.47	0.51	0.53	0.55	0.58
84	0.42	0.43	0.45	0.45	0.46	0.46	0.48	0.53	0.58	0.60	0.63	0.66	0.43	0.48	0.52	0.54	0.56	0.59

*SCOR, SphygmoCor device; MOG, Mobil-O-Graph device; Percentiles, 50th, 75th, 90th, 95th, 97.5th, and 99th; y, years old.*

**TABLE 5:** Body height-related reference intervals for central waveform-derived indexes (All: Females and Males).

	Brachial Oscillometry (MOG)						Radial Artery Tonometry (SCOR)						Carotid Artery Tonometry (SCOR)					
BH [m]	50th	75th	90th	95th	97.5th	99th	50th	75th	90th	95th	97.5th	99th	50th	75th	90th	95th	97.5th	99th
**Augmentation Pressure (AP; mmHg)**																		
1.0	4.42	6.28	8.78	10.85	13.12	16.51	2.31	3.65	4.86	5.59	6.22	6.97	−5.89	−3.03	−0.50	0.99	2.27	3.75
1.1	4.42	6.02	8.06	9.67	11.39	13.87	2.28	3.95	5.46	6.37	7.17	8.10	−5.73	−2.29	0.74	2.52	4.05	5.81
1.2	4.49	5.99	7.88	9.34	10.89	13.07	2.25	4.24	6.06	7.16	8.12	9.24	−5.58	−1.55	1.97	4.04	5.81	7.86
1.3	4.60	6.16	8.10	9.62	11.21	13.48	2.21	4.54	6.67	7.95	9.08	10.39	−5.42	−0.82	3.19	5.54	7.56	9.87
1.4	4.76	6.48	8.69	10.43	12.29	14.97	2.18	4.84	7.27	8.75	10.04	11.55	−5.27	−0.10	4.40	7.03	9.28	11.87
1.5	4.97	6.98	9.66	11.84	14.22	17.75	2.15	5.14	7.88	9.55	11.01	12.71	−5.11	0.63	5.60	8.51	10.99	13.84
1.6	5.22	7.66	11.08	14.01	17.30	22.37	2.12	5.45	8.50	10.35	11.98	13.88	−4.96	1.35	6.79	9.97	12.69	15.79
1.7	5.51	8.55	13.10	17.22	22.08	29.92	2.09	5.75	9.11	11.16	12.95	15.06	−4.81	2.06	7.98	11.43	14.37	17.73
1.8	5.85	9.69	15.94	21.99	29.54	42.54	2.05	6.05	9.73	11.97	13.94	16.25	−4.65	2.78	9.15	12.87	16.03	19.64
1.9	6.23	11.15	19.93	29.17	41.52	64.58	2.02	6.35	10.35	12.78	14.92	17.44	−4.50	3.49	10.32	14.30	17.68	21.53

**Augmentation Index (AIx; %)**																		
1.0	20.29	25.18	29.92	32.92	35.63	38.89	12.66	19.00	24.83	28.37	31.48	35.13	−18.65	−10.64	−2.90	1.98	6.36	11.62
1.1	19.16	23.95	28.62	31.58	34.25	37.47	10.69	17.96	24.65	28.74	32.34	36.56	−17.70	−8.71	0.03	5.57	10.55	16.55
1.2	18.21	23.04	27.75	30.74	33.45	36.71	9.18	17.19	24.60	29.13	33.11	37.81	−16.75	−6.74	3.04	9.26	14.87	21.63
1.3	17.41	22.36	27.21	30.30	33.10	36.49	8.04	16.65	24.63	29.52	33.83	38.90	−15.78	−4.74	6.11	13.04	19.29	26.85
1.4	16.73	21.87	26.94	30.18	33.12	36.69	7.23	16.31	24.75	29.93	34.49	39.87	−14.81	−2.71	9.25	16.90	23.83	32.21
1.5	16.15	21.55	26.89	30.33	33.45	37.25	6.69	16.14	24.94	30.34	35.10	40.72	−13.83	−0.64	12.45	20.86	28.48	37.71
1.6	15.66	21.35	27.04	30.70	34.04	38.12	6.38	16.12	25.19	30.76	35.68	41.48	−12.83	1.46	15.72	24.90	33.23	43.35
1.7	15.24	21.27	27.34	31.27	34.86	39.25	6.29	16.23	25.49	31.19	36.21	42.14	−11.83	3.59	19.05	29.03	38.10	49.13
1.8	14.87	21.28	27.77	32.00	35.88	40.63	6.37	16.45	25.84	31.62	36.72	42.74	−10.82	5.76	22.45	33.24	43.08	55.05
1.9	14.56	21.38	28.34	32.89	37.08	42.22	6.61	16.77	26.23	32.06	37.19	43.26	−9.80	7.95	25.90	37.54	48.16	61.10

**Augmentation Index adjusted by heart rate 75 beats/minute (AIx@75; %)**																		
1.0	32.40	36.84	40.93	43.44	45.64	48.24	18.41	23.51	28.09	30.84	33.23	36.01	−8.09	−2.10	3.41	6.77	9.72	13.19
1.1	29.06	34.82	40.19	43.50	46.42	49.87	16.88	23.73	29.91	33.61	36.84	40.59	−10.17	−1.88	5.83	10.56	14.73	19.66
1.2	25.81	32.43	38.67	42.53	45.95	50.02	15.13	23.30	30.66	35.09	38.93	43.41	−11.70	−1.59	7.87	13.71	18.87	24.98
1.3	22.66	29.79	36.55	40.76	44.50	48.95	13.21	22.30	30.49	35.42	39.70	44.69	−12.78	−1.27	9.58	16.30	22.25	29.31
1.4	19.63	26.97	33.98	38.36	42.26	46.92	11.12	20.78	29.50	34.74	39.30	44.61	−13.48	−0.90	11.01	18.41	24.97	32.76
1.5	16.73	24.04	31.07	35.48	39.41	44.11	8.88	18.81	27.77	33.16	37.85	43.30	−13.86	−0.49	12.20	20.09	27.10	35.43
1.6	13.94	21.05	27.91	32.23	36.09	40.71	6.52	16.44	25.38	30.76	35.44	40.90	−13.97	−0.06	13.16	21.40	28.71	37.41
1.7	11.29	18.04	24.58	28.71	32.41	36.84	4.04	13.69	22.39	27.63	32.19	37.49	−13.82	0.40	13.94	22.37	29.87	38.78
1.8	8.77	15.04	21.15	25.01	28.48	32.63	1.46	10.61	18.86	23.83	28.15	33.19	−13.46	0.88	14.54	23.05	30.61	39.60
1.9	6.39	12.09	17.67	21.20	24.38	28.19	−1.21	7.23	14.84	19.42	23.41	28.05	−12.91	1.39	14.99	23.46	30.98	39.93

**Forward pressure (Pf; mmHg)**																		
1.0	15.29	17.09	19.04	20.39	21.69	23.40	17.03	19.76	22.55	24.40	26.13	28.28	27.84	32.96	38.29	41.85	45.18	49.36
1.1	16.39	18.57	20.98	22.69	24.36	26.58	19.42	23.02	26.78	29.32	31.70	34.70	30.51	36.87	43.60	48.15	52.45	57.89
1.2	17.57	20.16	23.07	25.16	27.25	30.07	21.71	26.12	30.80	33.98	36.99	40.82	32.94	40.37	48.33	53.77	58.94	65.52
1.3	18.85	21.86	25.32	27.85	30.41	33.92	23.87	28.99	34.48	38.23	41.80	46.36	35.10	43.43	52.42	58.60	64.49	72.03
1.4	20.22	23.70	27.75	30.77	33.86	38.16	25.87	31.58	37.73	41.95	45.98	51.12	37.00	46.03	55.81	62.56	69.01	77.28
1.5	21.72	25.68	30.39	33.95	37.64	42.85	27.70	33.87	40.50	45.06	49.41	54.97	38.64	48.16	58.50	65.63	72.46	81.22
1.6	23.34	27.85	33.28	37.44	41.80	48.04	29.36	35.81	42.75	47.50	52.04	57.83	40.04	49.85	60.49	67.83	74.85	83.85
1.7	25.12	30.21	36.43	41.26	46.38	53.80	30.83	37.42	44.46	49.28	53.86	59.70	41.20	51.10	61.81	69.19	76.23	85.24
1.8	27.07	32.80	39.89	45.47	51.44	60.22	32.12	38.68	45.65	50.39	54.89	60.59	42.13	51.95	62.51	69.76	76.66	85.47
1.9	29.23	35.65	43.71	50.12	57.06	67.38	33.22	39.61	46.34	50.88	55.17	60.59	42.85	52.42	62.64	69.62	76.24	84.66

**Backward pressure (Pb; mmHg)**																		
1.0	8.37	9.54	10.77	11.61	12.40	13.40	9.02	10.35	11.69	12.55	13.34	14.31	10.25	12.29	14.42	15.83	17.16	18.81
1.1	8.82	10.29	11.89	12.99	14.06	15.44	9.95	11.57	13.20	14.26	15.23	16.44	11.29	13.61	16.03	17.64	19.16	21.06
1.2	9.41	11.19	13.15	14.53	15.88	17.66	10.82	12.69	14.59	15.83	16.97	18.39	12.30	14.89	17.58	19.39	21.09	23.21
1.3	10.16	12.24	14.57	16.24	17.88	20.07	11.64	13.73	15.85	17.24	18.53	20.13	13.30	16.13	19.09	21.07	22.94	25.28
1.4	11.08	13.46	16.18	18.14	20.08	22.69	12.40	14.67	16.98	18.50	19.91	21.65	14.27	17.33	20.53	22.68	24.70	27.24
1.5	12.17	14.89	17.99	20.25	22.50	25.53	13.11	15.52	17.97	19.59	21.09	22.94	15.22	18.49	21.92	24.22	26.38	29.10
1.6	13.48	16.54	20.05	22.61	25.17	28.61	13.76	16.27	18.84	20.52	22.08	24.01	16.15	19.61	23.25	25.69	27.98	30.86
1.7	15.04	18.46	22.38	25.24	28.10	31.96	14.36	16.94	19.57	21.29	22.89	24.86	17.05	20.70	24.52	27.08	29.48	32.51
1.8	16.90	20.69	25.03	28.19	31.34	35.58	14.91	17.53	20.18	21.92	23.52	25.50	17.94	21.75	25.73	28.40	30.90	34.05
1.9	19.11	23.29	28.05	31.49	34.91	39.50	15.41	18.03	20.67	22.39	23.98	25.95	18.80	22.75	26.88	29.65	32.24	35.49

**Reflection Magnitude (RM)**																		
1.0	0.55	0.59	0.62	0.64	0.65	0.67	0.52	0.58	0.64	0.69	0.72	0.77	0.38	0.44	0.49	0.53	0.56	0.60
1.1	0.55	0.60	0.63	0.66	0.68	0.70	0.51	0.58	0.65	0.70	0.74	0.80	0.38	0.45	0.52	0.56	0.60	0.65
1.2	0.55	0.60	0.65	0.67	0.70	0.72	0.50	0.57	0.65	0.71	0.76	0.83	0.38	0.46	0.54	0.59	0.64	0.70
1.3	0.56	0.61	0.66	0.69	0.71	0.74	0.49	0.57	0.66	0.72	0.78	0.85	0.38	0.47	0.56	0.62	0.68	0.75
1.4	0.56	0.62	0.67	0.70	0.72	0.75	0.48	0.57	0.66	0.73	0.79	0.87	0.39	0.48	0.58	0.65	0.71	0.79
1.5	0.58	0.64	0.68	0.71	0.73	0.76	0.47	0.56	0.67	0.74	0.81	0.90	0.40	0.50	0.60	0.67	0.74	0.82
1.6	0.59	0.65	0.69	0.72	0.74	0.77	0.47	0.56	0.67	0.75	0.82	0.92	0.41	0.51	0.62	0.69	0.76	0.85
1.7	0.60	0.66	0.70	0.73	0.75	0.77	0.46	0.56	0.67	0.75	0.83	0.94	0.42	0.53	0.64	0.71	0.78	0.87
1.8	0.62	0.67	0.71	0.74	0.75	0.78	0.46	0.56	0.68	0.76	0.85	0.95	0.44	0.55	0.66	0.73	0.80	0.89
1.9	0.64	0.68	0.72	0.74	0.76	0.78	0.45	0.56	0.68	0.77	0.86	0.97	0.45	0.56	0.68	0.75	0.82	0.91

**Reflection Index (RIx)**																		
1.0	0.36	0.37	0.38	0.39	0.40	0.40	0.34	0.37	0.39	0.41	0.42	0.44	0.28	0.30	0.33	0.35	0.36	0.38
1.1	0.35	0.37	0.39	0.40	0.40	0.41	0.34	0.37	0.39	0.41	0.43	0.44	0.27	0.31	0.34	0.36	0.38	0.40
1.2	0.35	0.38	0.39	0.40	0.41	0.42	0.33	0.36	0.39	0.41	0.43	0.45	0.27	0.31	0.35	0.37	0.39	0.41
1.3	0.36	0.38	0.40	0.41	0.42	0.42	0.33	0.36	0.40	0.42	0.43	0.46	0.28	0.32	0.36	0.38	0.40	0.43
1.4	0.36	0.38	0.40	0.41	0.42	0.43	0.32	0.36	0.40	0.42	0.44	0.46	0.28	0.32	0.37	0.39	0.42	0.44
1.5	0.37	0.39	0.41	0.42	0.42	0.43	0.32	0.36	0.40	0.42	0.44	0.47	0.28	0.33	0.37	0.40	0.43	0.45
1.6	0.37	0.39	0.41	0.42	0.43	0.44	0.32	0.36	0.40	0.43	0.45	0.47	0.29	0.34	0.38	0.41	0.43	0.46
1.7	0.38	0.40	0.41	0.42	0.43	0.44	0.32	0.36	0.40	0.43	0.45	0.48	0.30	0.34	0.39	0.42	0.44	0.47
1.8	0.38	0.40	0.42	0.42	0.43	0.44	0.32	0.36	0.40	0.43	0.46	0.49	0.30	0.35	0.40	0.42	0.45	0.48
1.9	0.39	0.41	0.42	0.43	0.43	0.44	0.31	0.36	0.41	0.43	0.46	0.49	0.31	0.36	0.40	0.43	0.45	0.48

*BH, body height in meters.*

The minimum sample size required was 377 (Bellera and Hanley, 2007). Like in previous works and according to the central limit theorem, normal distribution was considered (considering Kurtosis and Skewness coefficients distribution and sample size >30) (Lumley et al., 2002). Data analysis was done using MedCalc-Statistical software (v.18.5, MedCalc Inc., Ostend, Belgium) and IBM-statistical package for the social sciences (SPSS) software (v.26, SPSS Inc., IL, United States). PROCESS v.3.5 (SPSS extension) was used for moderation (interaction) analysis (Hayes, 2020). A *p* < 0.05 was considered statistically significant.

## Results

### Agreement of Waveform-Derived Indices Obtained From Carotid, Radial, and Brachial Recordings

Table 3 (Supplementary Table 4) shows correlation coefficients and Bland–Altman analyses carried out to determine the agreement between data of a “similar waveform-derived index” obtained with different devices and/or approaches. Although the associations between indexes (e.g., Pb obtained with different approaches) were significant (95% CI did not cross zero), they were “weak” or “moderate.” Then, regardless of whether AP, AIx@75, Pf, Pb, RM, or RIx were considered: (i) the levels of association were always statistically significant and (ii) in all cases, concordance correlation coefficients showed low agreement between data (values were always <0.66). For instance, concordance correlation coefficients [95% CI] were 0.43 [0.38–0.47], 0.30 [0.25–0.35], and 0.62 [0.59–0.64] for the association between (i) Pb_MOG and Pb_SCOR_Radial, (ii) Pb_MOG and Pb_SCOR_Carotid and (iii) Pb_SCOR_Radial and Pb_SCOR_Carotid, respectively. It is to note that the highest levels of association were obtained when analyzing carotid and radial tonometry data (SCOR_Radial and SCOR_Carotid), whereas data from brachial oscillometry (MOG) and carotid tonometry (SCOR_Carotid) showed the lowest levels of association (Table 3 and Supplementary Table 4).

Bland–Altman tests showed not only significant systematic, but also proportional differences (errors). Then the differences varied in magnitude depending on the index values (Table 3 and Supplementary Table 4). The highest systematic (mean) errors were obtained when analyzing agreement between MOG and carotid tonometry (SCOR_Carotid). As a result, specific RIs for all the waveform-derived indexes obtained were defined as necessary.

###  Age-, Sex-, and/or Body Height-Related Differences

Age, BH, and/or sex-specific RIs for a given waveform-derived index (e.g., AIx@75) may (or may not) be required, depending on the approach used for its measurement (MOG *vs.* SCOR_Carotid *vs.* SCOR_Radial). For instance, Pb_MOG was independently associated with sex and BH, but not with age. In turn, Pb_Radial_SCOR and Pb_Carotid_SCOR showed independent associations with age and BH (but not with sex). AIx@75_MOG was independently associated with sex and age (and BH), without age-dependent changes in the association with sex (non-significant Johnson-Neyman regions).

On the other hand, AIx@75_SCOR_Radial and AIx@75_SCOR_Carotid were associated with age, but not with sex, but in subjects aged 7–8 y and older, sex moderated the “AIx@75-age” association. Regardless of the method (MOG or SCOR) considered, RM and RIx were not independently associated with sex. However, for SCOR_Radial and SCOR_Carotid records, in subjects aged 17 years and older, sex moderated the relationship with age.

Regardless of the measurement method, almost all waveform-derived indexes showed an independent association with age and BH (and/or an association through their interaction with sex). Consequently, both age- and BH-related RIs were necessary. In contrast, we identified waveform-derived indexes that: (i) required sex-specific RIs only from a certain age (e.g., AP_SCOR_Radial) or BH (e.g., Pf_SCOR_Radial), (ii) required sex-specific RIs regardless of age or BH (e.g., Pf_MOG, Pb_MOG), (iii) did not require sex-specific RIs (e.g., Pb_SCOR_Radial), or (iv) did not require sex-specific and/or BH-related RIs (e.g., RI_MOG) (Supplementary Tables 5–11).

###  Age-, Sex-, and/or Body Height-Related Reference Intervals

Year-by-year (for age) and decimeter-by-decimeter (for BH) RIs data can be found in Supplementary Tables 14–139. Supplementary Materials 2 and 3 show age-related (Supplementary Figures 1–21) and BH-related (Supplementary Figures 22–42) percentile curves for waveform-derived indexes. Tables 4, 5 show a summary (5 y and 1 decimeter intervals) of age- and BH-related RIs data for each waveform-derived index.

**FIGURE 3:**
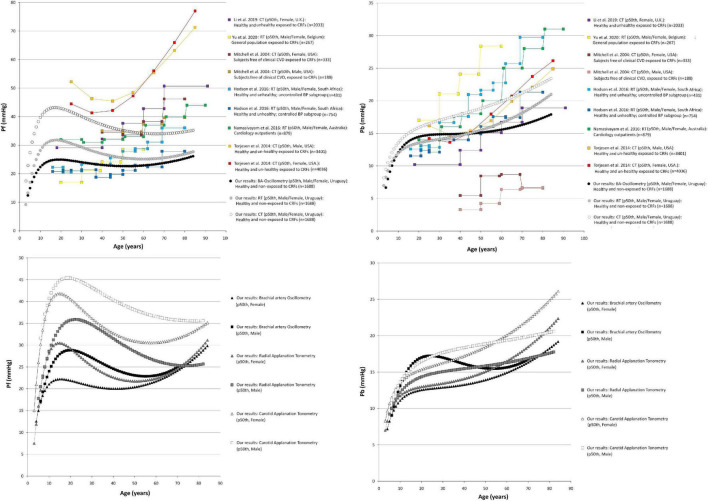
Forward pressure (Pf) and backward pressure (Pb) age-related profiles. Top: Comparison between curves obtained in our study (p50th) and mean values or p50th obtained by other authors. CT, carotid applanation tonometry; CRFs, cardiovascular risk factors; RT, radial applanation tonometry; U.K., United Kingdom; United States, United States of America; BP, blood pressure; BA, brachial artery; Bottom: Age-related profiles (p50th) for males and females.

**FIGURE 4:**
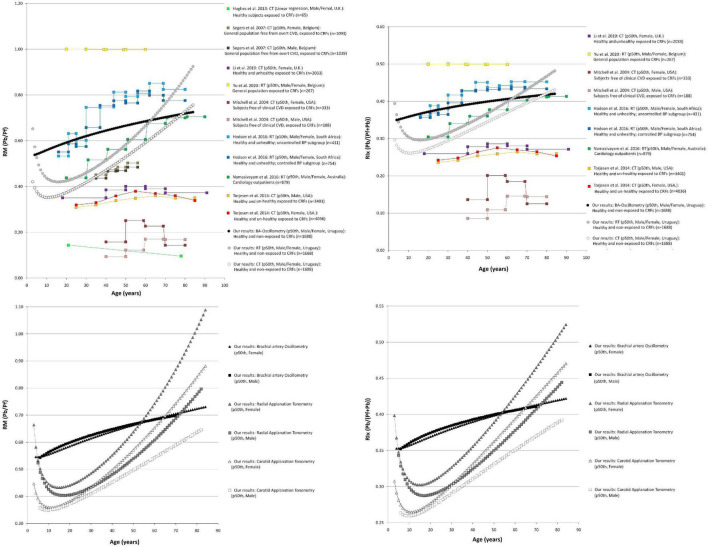
Reflection magnitude (RM) and Reflection index (RIx) age-related profiles. Top: Comparison between curves obtained in our study (p50th) and mean values or p50th obtained by other authors. Pb and Pf, backward and forward pressure; CT, carotid applanation tonometry; CVD, cardiovascular disease; CRFs, cardiovascular risk factors; RT, radial applanation tonometry; U.K., United Kingdom; United States, United States of America; BP, blood pressure; BA, brachial artery. Bottom: Age-related profiles (p50th) for males and females.

**FIGURE 5:**
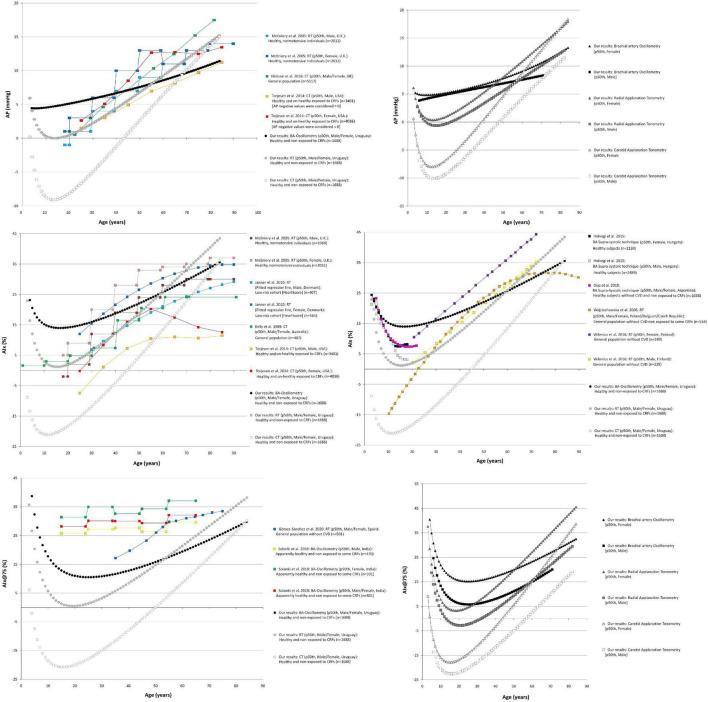
Augmentation pressure (AP), Augmentation Index (AIx), and heart rate-corrected AIx (AIx@75) age-related profiles. Left and right middle panel: Comparison between curves obtained in our study (p50th) and mean values or p50th obtained by other authors. CT, carotid applanation tonometry; CVD, cardiovascular disease; CRFs, cardiovascular risk factors; RT, radial applanation tonometry; U.K., United Kingdom; United States, United States of America; BP, blood pressure; BA, brachial artery. Right top and bottom panel: Age-related profiles (p50th) for males and females.

**FIGURE 6:**
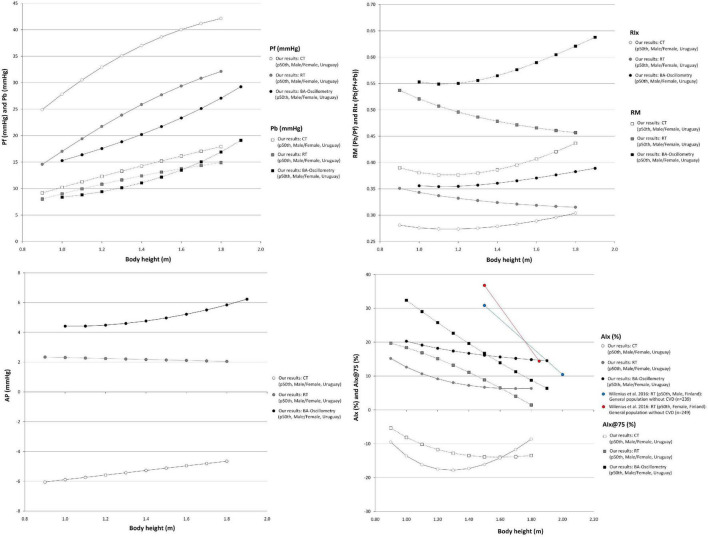
Waveform-derived indexes body height-related profiles. Comparison between curves for waveform derived indexes obtained in our study (p50th) using three different approaches. Pf, Forward pressure; Pb, Backward pressure; RM, Reflection magnitude; RIx, Reflection index; AP, Augmentation pressure; AIx and AIx@75, Augmentation index and heart rate-corrected AIx; CT, carotid applanation tonometry; CVD, cardiovascular disease; RT, radial applanation tonometry; BA, brachial artery.

Comparisons of data from this study (p50th for all subjects), with data obtained by other authors (p50th or mean value), are shown in Figures 3–6.

## Discussion

### Main Findings

Considering their demonstrated value (e.g., as prognostic tool), there is growing interest in assessing central BP waveform-derived indexes in clinical practice. Their accurate clinical use requires knowing their physiological age-related profiles and the expected values for a specific subject. To our knowledge, this is the first time RIs for different waveform-derived indexes (obtained from different widely used measurement approaches) were defined (at the same time) in large population of healthy children, adolescents, and adults (2–84 y). The main findings can be summarized as follows:

First, methods used to quantify aoBP waveform-derived indexes (brachial oscillometry; carotid and radial tonometry) showed mostly little (statistically significant) association with each other. Furthermore, in no case, the association was very strong (*r* ≥ 0.80) (Table 3). Methodological approaches used to quantify similar waveform-derived indexes (e.g., Pb) were not equivalent, but showed systematic and proportional errors (Table 3).

Second, the need for sex-specific RIs relied on both physiological (e.g., age and/or waveform-derived index) and non-physiological factors (e.g., methodological approach used) (Supplementary Table 11). Then, the associations of a given arterial propagate property (e.g., reflection coefficient) with subjects’ characteristics (e.g., sex, age) could vary depending on the approach used in the evaluation.

Third, the population-based RIs for waveform-derived indexes were defined from data obtained in the same group of healthy children, adolescents, and adults (Tables 4, 5 and Supplementary Tables 14–139). Defining RIs is an important step when considering the use of waveform-derived indexes in laboratory and clinical practice, for example, as a tool to identify conditions associated with data deviation from anticipated values in physiological settings and/or to detect subclinical target organ (vascular) damage.

Fourth, aiming at contributing with other groups and/or researchers, sex-specific BH- and age-related equations for mean value, SD, and percentile values were included in text and spreadsheet formats (Supplementary Material 1). Thus, the expected values for a given subject could be calculated.

### Wave Separation Analysis-Derived Indexes: Forward Pressure, Backward Pressure, Reflection Magnitude, and Reflection Index

#### Age-Related Profiles

The age-related profiles obtained for WSA-derived indexes showed similarities and differences with data reported by other authors (Figures 3, 4). However, it is to note that although some articles showed age-related changes in waveform-derived indexes, data from large populations of healthy subjects, with minimized exposure to traditional CRFs, are scarce, limiting the possibility of comparing our findings with those of other authors. Moreover, we found no work including children, adolescents, and adults.

Regardless of the methodological approach considered, the Pf increased in childhood and adolescence, reaching a maximum at ∼20 y. Thereafter, it remained stable and finally showed a tendency to increase after 60–70 y (Figure 3). At least in theory, the significant increase in Pf during childhood and adolescence could be related to (explained by) changes in stroke volume. In this regard, it was previously described that early in life, there is a rapid increase in stroke volume, reaching a peak at ∼20 y, followed by a slow decline from the beginning of the third decade of life (Cattermole et al., 2017; Zócalo et al., 2020). The (relative) Pf stability observed within the range of 30 and 60 y of age is in agreement with previous findings in subjects with controlled and uncontrolled blood pressure (Hodson et al., 2016), in healthy and unhealthy subjects (Li et al., 2019) and in subjects with cardiovascular disease (Namasivayam et al., 2016). On the other hand, whereas in this work, the increase in Pf after 70 y of age was discrete, works that included subjects with cardiovascular disease and/or exposed to CRFs described a greater (steeper) increase (Figure 3). At least in theory, the dissimilar findings could be explained by a cumulative effect of cardiovascular disease and/or CRFs on Pf determinants (e.g., aortic root impedance). In this regard, in Hodson et al. work, it was observed that compared with subjects with controlled blood pressure, those with uncontrolled pressure showed almost identical Pf (and Pb) levels at early ages (e.g., <21, 21–25, and 30–37 y). Subsequently, the Pf levels were gradually higher in subjects with uncontrolled blood pressure (with the differences being statistically significant from ∼45 y) (Figure 3; Hodson et al., 2016). In addition, since the Pf component actually integrates forward wave and re-reflections of backward waves at the ventricular-aorta interface, the increase in Pf could also be explained by increased reflections (and the subsequent increase in re-reflections). In this regard, it should be noted that aging has been associated with arterial stiffening and increased wave reflections. Finally, it is worth noting that despite of the differences in Pf values, the different methodological approaches considered enabled to obtain similar age-related profiles.

Taking into account the above mentioned, the differences in Pf values among studies could be explained (at least partially) by differences in the calibration methods, methodological approaches, and/or subjects considered.

Similarly, Pb showed a significant rate of increase in the first decade of life, and then it continued to increase steadily throughout life. The age-related profiles obtained for adults are in agreement with data from other works. However, the age-related increase in Pb observed in this work was (apparently) smaller compared with data from other works. Furthermore, for young subjects (e.g., 20–30 y), data from this and other works overlapped, but the maximum values reported by other authors for old subjects (e.g., 80–90 y) were almost always higher than the observed in this work (Figure 3). The differences could be ascribed to differences in the populations studied (Figure 3). In this regard, it is to note that this work was carried out in healthy subjects with minimized exposure to risk factors.

Jointly considering the above factors, it could be said that more flattened curves would be expected for both Pf and Pb in adults without cardiovascular disease and minimally exposed to traditional CRFs. Finally, the differences in the rate of Pf and Pb change observed in adults in this work are consistent with data from other studies (Cecelja et al., 2009; Namasivayam et al., 2009, 2016).

The age-related changes in RM (Pb/Pf) and RIx [Pb/(Pf + Pb)] showed great heterogeneity (Figure 4). Both RM and RIx tend to increase in adult life, but the rate of increase differed depending on the methodological approach considered (MOG or SCOR). In addition, while RM and RIx obtained with tonometry (SCOR) showed a reduction in the first years of life and started to increase after the age of 10 y, the data obtained from MOG increased through all the age-range considered (Figure 4). The finding of an age-related increase in RM and RIx is in agreement with other authors (Segers et al., 2007; Hodson et al., 2016; Namasivayam et al., 2016). On the other hand, Torjesen et al. (2014) and Li et al. (2019) reported a slight increase in RM and RIx until ∼60 y, followed by a subsequent reduction. In turn, Hughes et al. (2013) (in a small sample of subjects exposed to CRFs) found an age-associated decrease in RM. Finally, there were differences in RM and RIx data among works, even at ages in which neither the cardiovascular disease presence nor the exposure to CRFs could contribute to explain them (e.g., RM values equal to ∼1.0 [Yu et al., 2020], 0.55 [Hodson et al., 2016], 0.45 [Namasivayam et al., 2016], 0.35 [Li et al., 2019], and 0.15 [Hughes et al., 2013] were observed in subjects aged ∼20 y) (Figure 4).

#### Sex-Related Differences

Data from multiple linear regression models (Supplementary Table 11) and age-related profiles (Figures 3–5) showed that the association between waveform-derived indexes (Pf, Pb, RM, and RIx) and sex differed depending on the approach considered.

Figure 3 shows that after approximately 10–15 y, the p50th for Pf and Pb was higher in males than in females, but Pf values tend to be similar at ages over 70 y. In turn, for subjects over 50–60 y, the Pb values were higher in females than in males (Figure 3). In regression analysis, adjusted by BH, the Pf and Pb remained associated with sex only when considering data from MOG. This is in agreement with other authors (Lieber et al., 2010; Liao et al., 2011; Hughes et al., 2013).

The above data should be carefully analyzed. Whereas we (and other authors) analyzed Pf and Pb and absolute values, and other authors analyzed waveform components in terms relative to aoPP. In subjects younger than 60 y of age, Namasivayam et al. found that both incident and reflected waves showed a greater contribution to the age-related increase in aortic pressure in females than in males (Namasivayam et al., 2009; Torjesen et al., 2014; Hodson et al., 2017). Our curves for RM and RIx (indexes that allow relativizing Pb amplitude) tended to be higher in females from ∼20 y; except for MOG-derived data (Figure 4). Opposite to the described levels for Pf and Pb, RM and RIx levels obtained with SCOR showed sex-related differences (higher values in females), from 13.5 to 12.6 y for SCOR_Radial and from 17.1 to 17.2 y for SCOR_Carotid. Our findings add to those reported by Namasivayam et al. (2009). These authors found that the higher relative contribution of Pb in females would be observed from ∼12 to ∼17 y for radial and carotid recordings, respectively. Additionally, the authors described that the sex-related differences remained thorough adult life.

The similarity observed in girls and boys agrees with previous works that reported no sex-related differences in structural and functional arterial parameters at prepubertal ages (4–8 y), but showed sex-related differences in adolescents (∼15 y) (Curcio et al., 2016).

#### Body Height-Related Profiles

Regardless of the methodological approach considered, and with independence of sex and age, Pf and Pb were positively associated with BH (Figure 6 and Supplementary Table 11). In turn, RM and RIx data obtained with MOG did not show an independent association with BH, whereas SCOR-derived RM and RIx data were independently associated with BH (higher BH, lower RM and RIx) (Supplementary Tables 9, 10). The negative association of BH with RM and RIx could be explained (at least partially) by the well-known inverse relationship between the magnitude of reflections measured at the central aorta and the distance between the site of wave generation (heart) and reflection (which move away as BH increases).

Our findings could be considered opposite to that reported by Hughes et al. (2013) who did not find association between RM and BH in healthy normotensive subjects (*n* = 65; 21–78 y; 43 male) evaluated with carotid tonometry. However, the results were alike when considering the association of BH with RM and RIx data obtained with SCOR_Carotid without adjusting for cofactors (Figure 6; note the flattened profiles).

### Pulse Wave Analysis-Derived Indexes: Augmentation Pressure, Augmentation Index, and Augmentation Index Corrected for Heart Rate 75 Beats/Minute

#### Age-Related Profiles

Like in previous works, the AP showed an age-related increase in adults (Figure 5). When considering SCOR data, it was observed as a decrease in AP during childhood and adolescence and an increase from the age of ∼15 onward. The AP levels obtained with MOG showed an increase from childhood onward. Additionally, there were differences in the AP levels obtained with the different methodological approaches (Figure 5).

Regardless of the methodological approach considered, the AIx and AIx@75 showed a reduction in childhood and adolescence, but they increased from ∼10–15 y onward (Figure 5). This is in agreement with previous findings in children and adolescents (Hidvégi et al., 2015; Díaz et al., 2018), and in adults (McEniery et al., 2005). We found a linear relationship with age that did not become non-linear in subjects aged 70 y and older as was described by other authors (McEniery et al., 2005). The dissimilar findings could be explained by differences in studied subjects’ characteristics (e.g., we evaluated healthy subjects minimally exposed to traditional CRFs, whereas other authors included subjects with extensive exposure to CRFs) and/or by demographics differences between the populations considered. About this, Janner et al. (2010) found that the association between AIx and age became progressively less linear (above 60–70 y) when considering subjects with increased cardiovascular risk (associated with exposure to CRFs). Other hypotheses have been proposed to explain the findings. Namasivayam et al. suggested that the ratio of two linear relationships, such as AP *vs.* age and aoPP *vs.* age, resulted in a curvilinear trend (AIx *vs.* age). This mathematical phenomenon could contribute to the “flattening” of age-AIx curve (described by other authors) (Hickson et al., 2016).

Finally, it is to note that only few authors reported reference data or age-related profiles for AIx@75. The available works described an age-related increase, but whereas Gómez-Sánchez et al. showed a significant constant increase in AIx@75 with aging (like in this work), and Solanki et al. found a slight age-related increase (Figure 5; Solanki et al., 2018; Gómez-Sánchez et al., 2020).

#### Sex-Related Differences

The augmentation pressure levels tended to be higher in females than in males (Figure 5), but the differences were statistically significant only in case of SCOR_Radial data from subjects aged > 12.9 y (Supplementary Table 11). This is in agreement with data from other authors (adult subjects) (Mitchell et al., 2004; McEniery et al., 2005; Segers et al., 2007; Torjesen et al., 2014).

Regardless of BH, compared to males, females showed higher AIx and AIx@75 (Figure 5): (i) for all ages when considering MOG records, (ii) from 9.5 and 7.8 y when considering SCOR_Radial records, and (iii) from 2.8 and 7.0 y when considering SCOR_Carotid records (Supplementary Table 11). The finding of higher AIx and AIx@75 in females than in males is in agreement with data from previous works (Mitchell et al., 2004; McEniery et al., 2005; Wojciechowska et al., 2006; Segers et al., 2007; Janner et al., 2010; Hughes et al., 2013; Torjesen et al., 2014; Wilenius et al., 2016; Solanki et al., 2018).

#### Body Height-Related Profiles

When analyzing the (simple) association between AP and BH data, no clear trend was observed (Figure 6). However, in multivariate analysis, it was observed a negative association between BH and AP. The association was independent of age and sex in case of SCOR and of age for MOG records (Supplementary Tables 8–11). In turn, the AIx and AIx@75 showed a negative association with BH, regardless of sex and age (Supplementary Tables 8–11). This is in agreement with Wilenius et al. (2016).

### Strengths and Limitations

Our results should be analyzed in the context of the work’s strengths and limitations. First, since this is a cross-sectional study, it provides no data on longitudinal age-related variations in waveform-derived indexes. Although useful for quantifying RIs, our data do not allow to determine with certainty the true impact of aging on arterial system properties (Campos-Arias et al., 2021). Second, the outcome data were not considered. Thus, cutoff points (e.g., p90th, p95th) could not be selected based on the association with increased cardiovascular risk, but on data distribution in the RIs group. Whether or not the RIs values should be used as cutoff values for central hemodynamic alterations diagnose and/or treatment is not known. Third, in this work, the concept of “waveform-derived index” was mainly presented as “static or unchanged,” rather than the composite of (i) “fixed or stable” (e.g., age-dependent arterial pulse propagation and reflection capabilities) and (ii) “variable or adjustable” (e.g., vascular smooth muscle capability to temporally adjust arterial pulse propagation or reflection properties) (Bia et al., 2003, 2008). Fourth, the RIs subgroup consisted of people whose CRFs levels did not exceed accepted thresholds for abnormality (e.g., hypertensive subjects were excluded). Given that the prevalence of many CRFs increases with age (e.g., arterial hypertension), by definition (older), “superhealthy” individuals would have had a greater possibility of being included in the RIs group. Consequently, at least for adults, this work’s data probably describe an “optimal,” rather than a “typical” aging trajectory.

Fifth, as a strength, in this study, waveform-derived indexes were obtained in a large population sample (of children, adolescents, and adults) that included subjects within a wide age-range (almost the whole range of life expectancy), as a continuum. This would contribute to understand the arterial pulse propagations and their impact on waveform-derived indexes throughout life, providing important information for clinical diagnosis and cardiovascular research. To our knowledge, this is the first study of its sort in South Americans. Sixth, despite we previously demonstrated that aoBP and some waveform-derived indexes could vary depending on the calibration schema considered (Zinoveev et al., 2019), in this work, we opted for the schema most used in the literature (use of baDBP and baMBP). In this regard, it is to note that whereas some indexes do not depend on the calibration schema (e.g., AIx), others are highly dependent on the methodological approach used (e.g., Pf and Pb). Hence, data should be analyzed/used being aware of this. Seventh, it is to note that an accurate optimal analysis of wave reflections would require pressure and flow measurement, rather than BP waveform recording and analysis alone. However, the simultaneous measurement of pressure and flow might not always be feasible. On the other hand, the different approaches used to quantify waveform-reflection indexes may differ in the obtained data (e.g., differences in the algorithms used to analyze the waveform and to identify the “inflection point” may yield different indexes values). In this context, we opted for working with widely used devices and algorithms of analysis (SphygmoCor and Mobil-O-Graph). Finally, it should be noted that while in this work we focused on the most frequently used waveform-derived indexes, we recognize (i) the existence of other indexes and (ii) the limitations of the quantified indexes. For instance, the AIx reflects both cardiac and vascular properties. Hence, it has limitations as a measure of wave reflection (Hughes et al., 2013; Heusinkveld et al., 2019).

## Conclusion

This study adds to the knowledge of the physiological variations in waveform-derived indexes and arterial pulse propagative properties that would be expected during growth and aging, analyzing at the same time (and comparatively) the behavior of different indexes, obtained with three different approaches. Our data showed that the methods used to quantify aoBP waveform-derived indexes (brachial oscillometry, carotid, and radial tonometry) showed little association with each other. Waveform-derived data from different approaches were not equivalent, but showed systematic and proportional errors. These results evidenced that the non-invasive methodological approach used is an important determinant of the results (e.g., RIs levels). Our study strongly emphasizes the need for consensus on non-invasive assessment of waveform-derived indexes.

There were not uniform behaviors that standardize the need for sex-related RIs (normative data), but the need for sex-specific waveform-derived indexes RIs relied on the index and/or age considered. Population-based RIs for waveform-derived indexes were defined from data obtained in the same group of healthy children, adolescents, and adults. Aiming at contributing with other groups and/or researchers, sex-specific BH- and age-related equations for mean value, SD, and percentiles values were included in text and spreadsheet formats. Thus, the expected values for a given subject could be calculated for clinical and/or research purposes.

## Data Availability Statement

The original contributions presented in the study are included in the article/Supplementary Material, further inquiries can be directed to the corresponding author/s.

## Ethics Statement

The studies involving human participants were reviewed and approved by Comité de Ética de Investigación, Centro Hospitalario Pereira Rossell, ASSE, Universidad de la República. Written informed consent to participate in this study was provided by the participants’ legal guardian/next of kin.

## Author Contributions

YZ and DB contributed to conception and design of the study, performed the cardiovascular recordings, constructed and organized the database, performed the statistical analysis, wrote the first draft and final version of the manuscript, contributed to manuscript revision, read, and approved the submitted version. Both authors contributed to the article and approved the submitted version.

## Conflict of Interest

The authors declare that the research was conducted in the absence of any commercial or financial relationships that could be construed as a potential conflict of interest.

## Publisher’s Note

All claims expressed in this article are solely those of the authors and do not necessarily represent those of their affiliated organizations, or those of the publisher, the editors and the reviewers. Any product that may be evaluated in this article, or claim that may be made by its manufacturer, is not guaranteed or endorsed by the publisher.

## Abbreviations

AIx: augmentation index
AIx@75: augmentation index corrected for heart rate 75 beats/minute
aoBP: central aortic blood pressure
aoDBP: central aortic diastolic blood pressure
aoPP: central aortic pulse pressure
aoSBP: central aortic systolic blood pressure
AP: augmentation pressure
baDBP: brachial artery diastolic blood pressure
baMBP: brachial artery mean blood pressure
baPP: brachial artery pulse pressure
baSBP: brachial artery systolic blood pressure
BH: body height
BMI: body mass index
BP: blood pressure
CRFs: cardiovascular risk factors
MOG: Mobil-O-Graph PWA-monitor system
Pb: backward (reflected) component of pressure waveform
Pf: forward (incident) component of pressure waveform
PWA: pulse wave analysis
RIs: reference intervals
RIx: reflection index
RM: reflection magnitude
SCOR: SphygmoCor-CvMS system
WSA: wave separation analysis
y: years.
