# Supplementary material for: Central Pressure Waveform-Derived Indexes Obtained From Carotid and Radial Tonometry and Brachial Oscillometry in Healthy Subjects (2–84 Y): Age-, Height-, and Sex-Related Profiles and Analysis of Indexes Agreement
Source: Front Physiol. 2022 Jan 20;12:774390. doi: 10.3389/fphys.2021.774390 (PMC8811372; doi:10.3389/fphys.2021.774390)
Supplement: Supplementary file 1 [file Data_Sheet_1.docx]

**Supplementary Figures: Central aortic waveform-derived parameters obtained using Mobil-O-Graph device: Age-related percentiles**


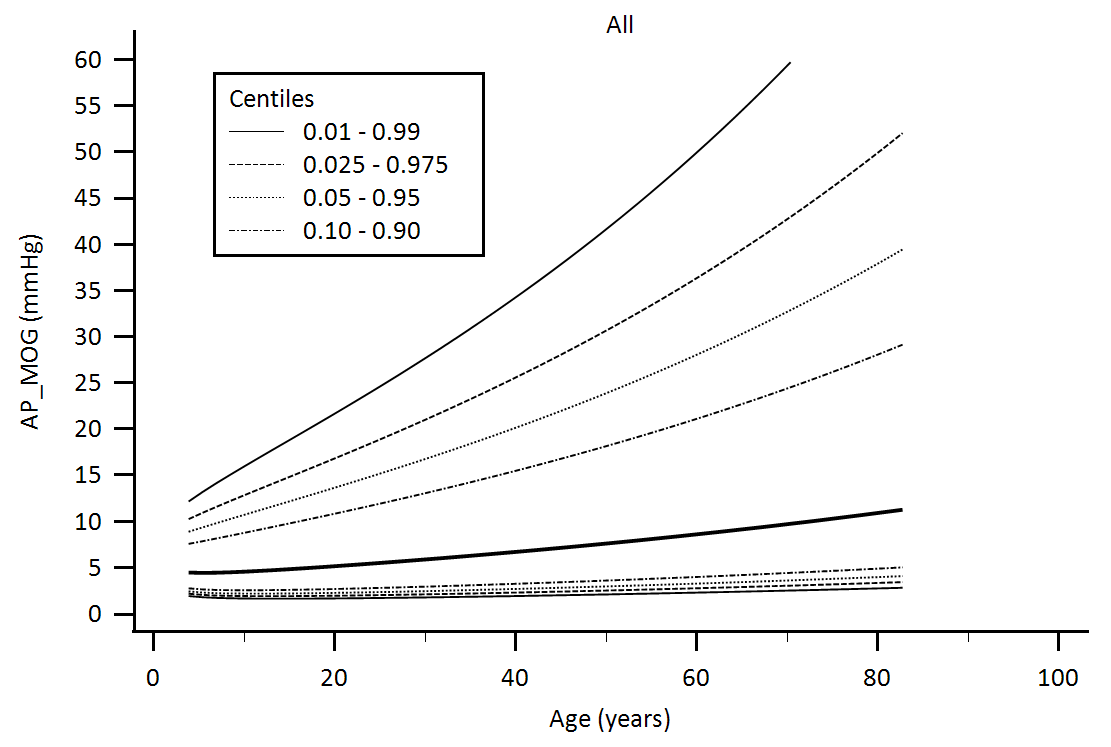


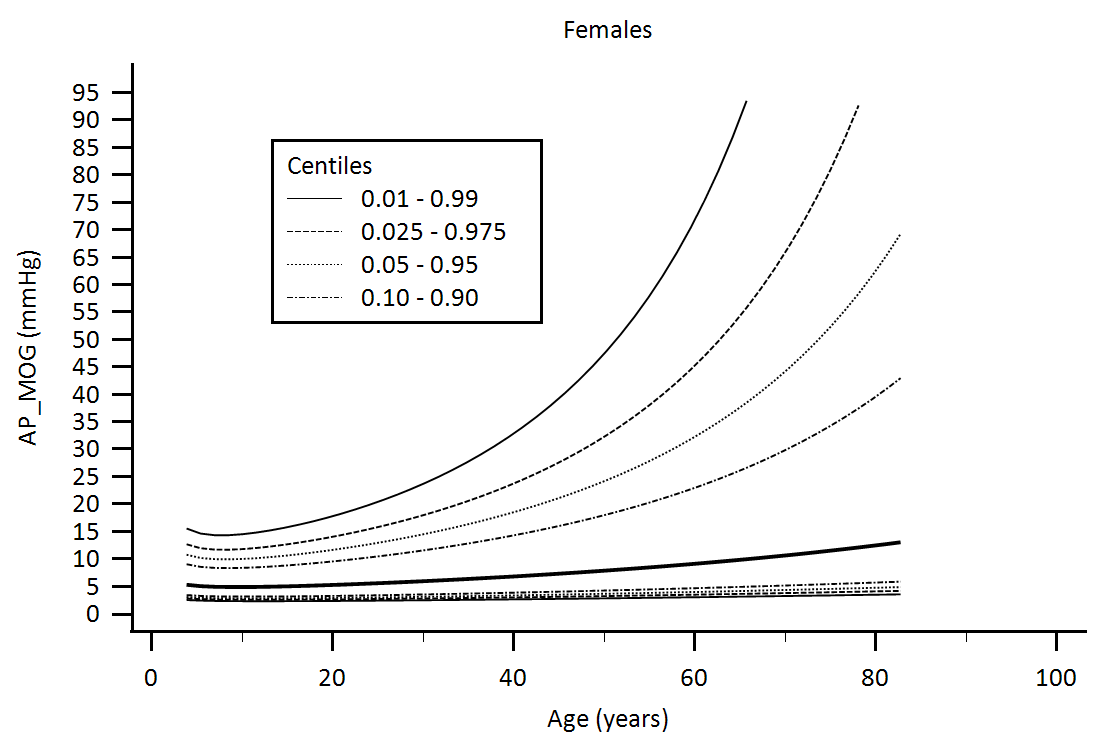


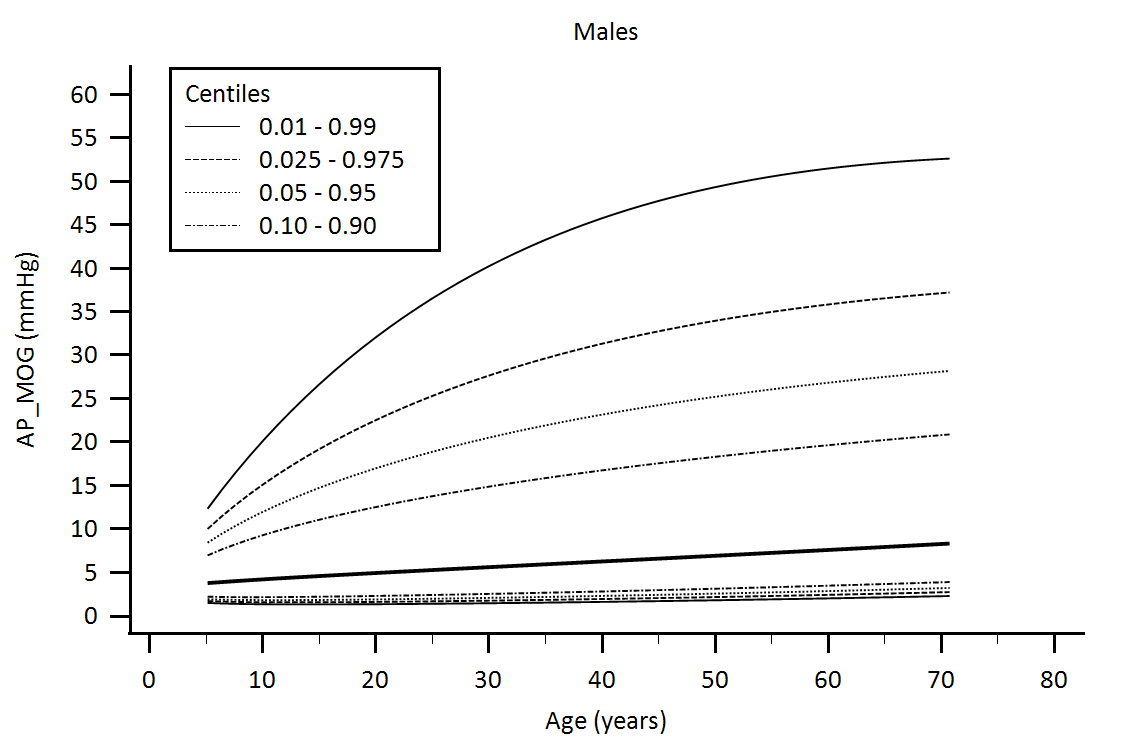


Supplementary Figure 1. Central aortic waveform-derived parameters obtained using Mobil-O-Graph device (MOG): Augmentation pressure (AP) age-related percentiles.


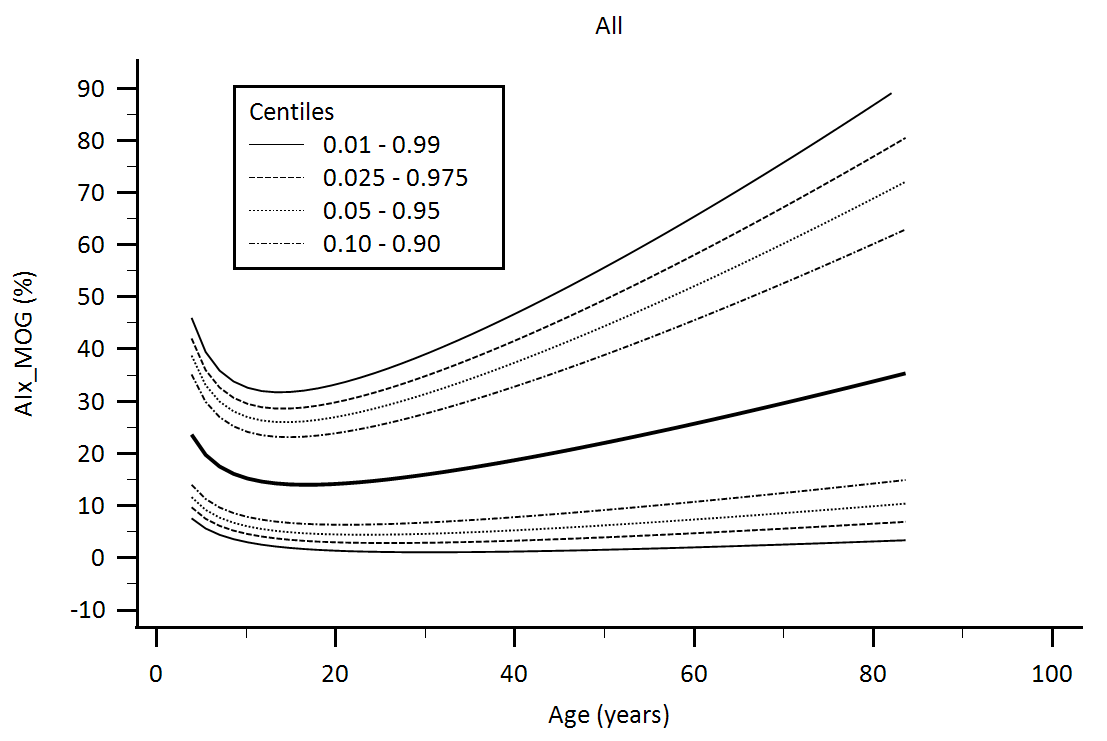


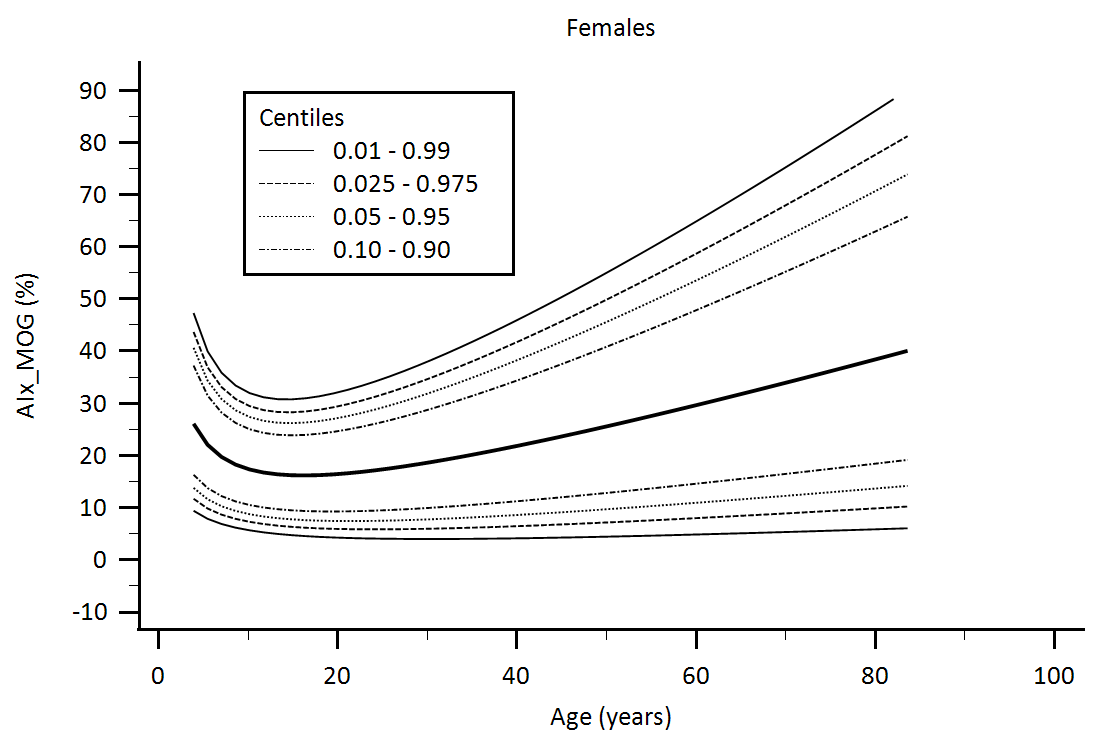


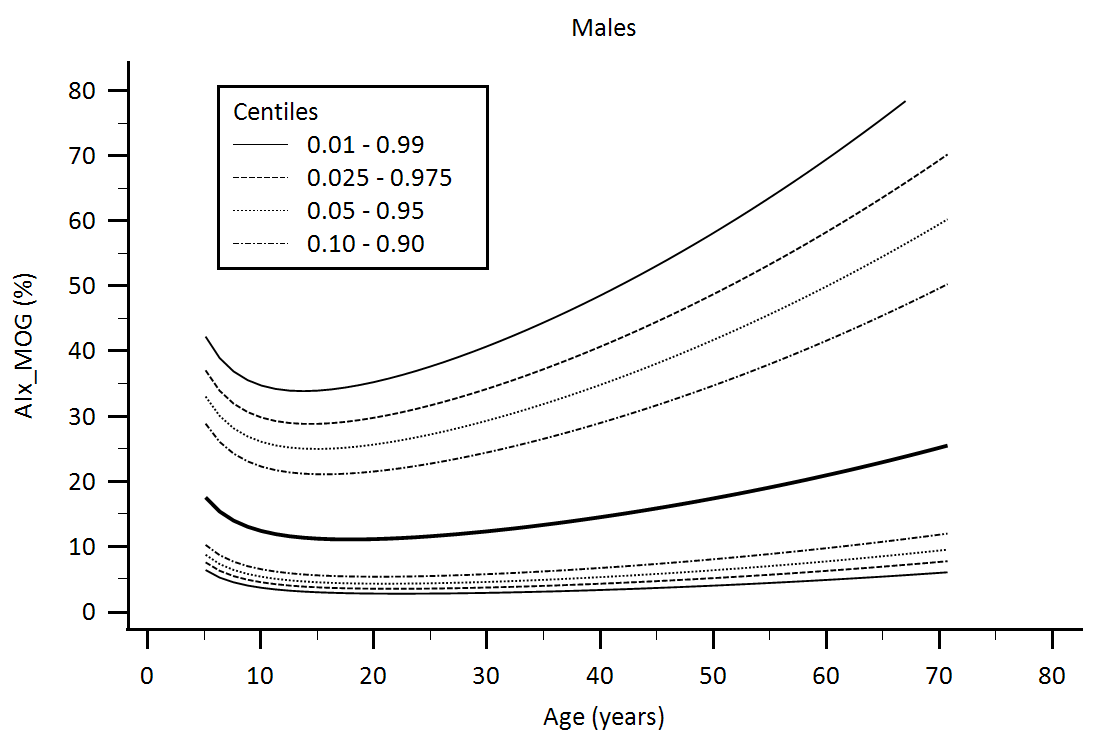


Supplementary Figure 2. Central aortic waveform-derived parameters obtained using Mobil-O-Graph device (MOG): Augmentation Index (AIx) age-related percentiles.


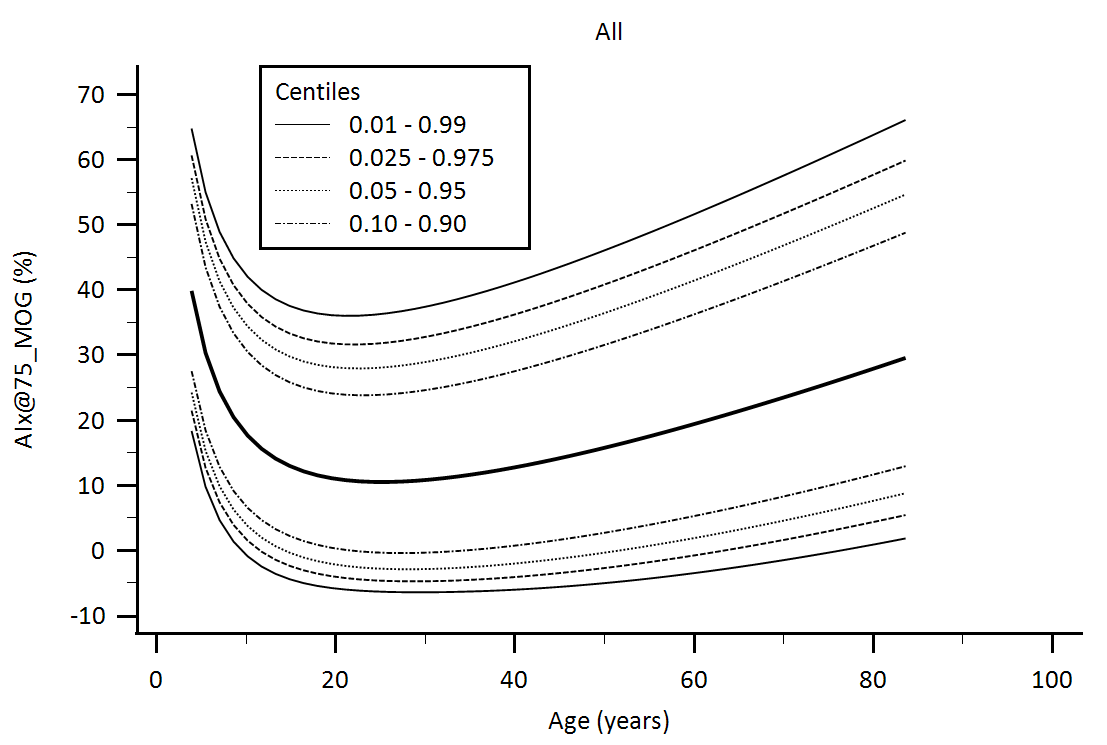


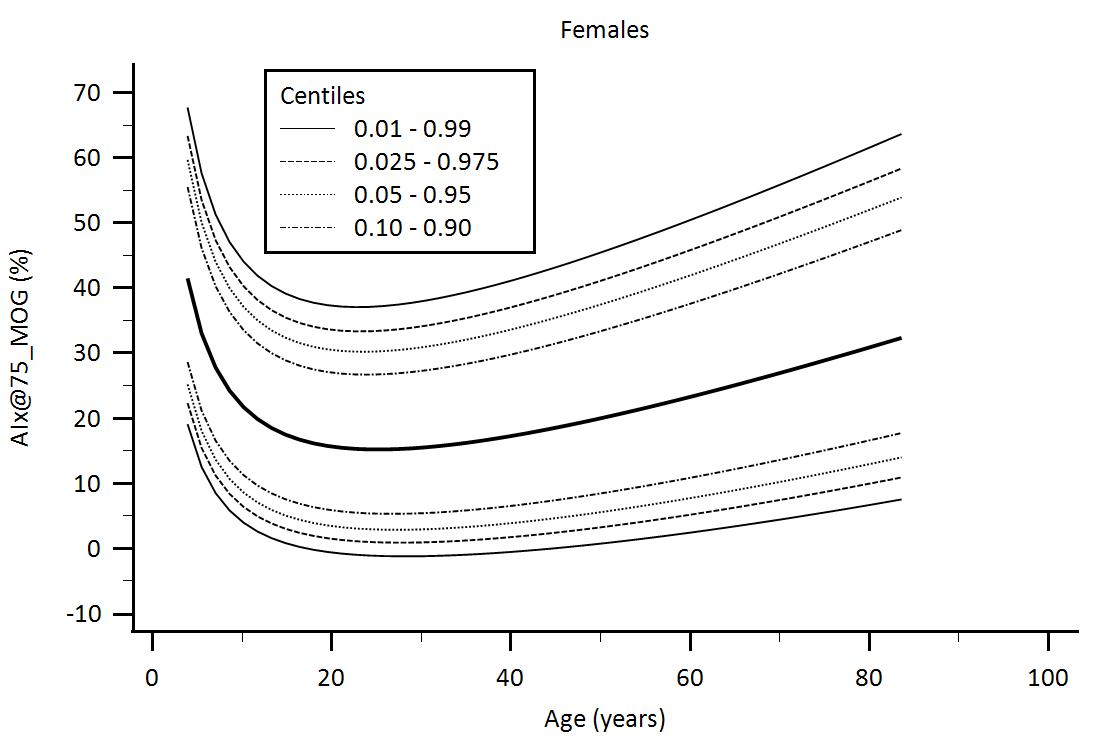

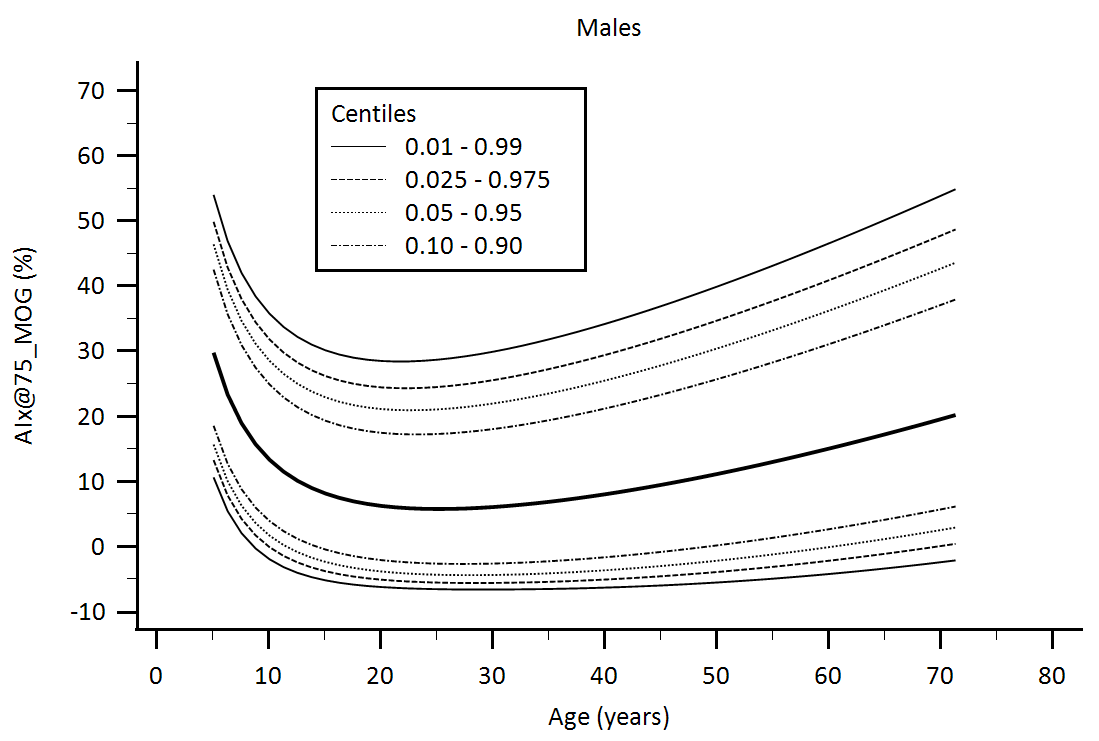


Supplementary Figure 3. Central aortic waveform-derived parameters obtained using Mobil-O-Graph device (MOG): Augmentation Index adjusted for heart rate equal 75 beats/minute (AIx@75) age-related percentiles.


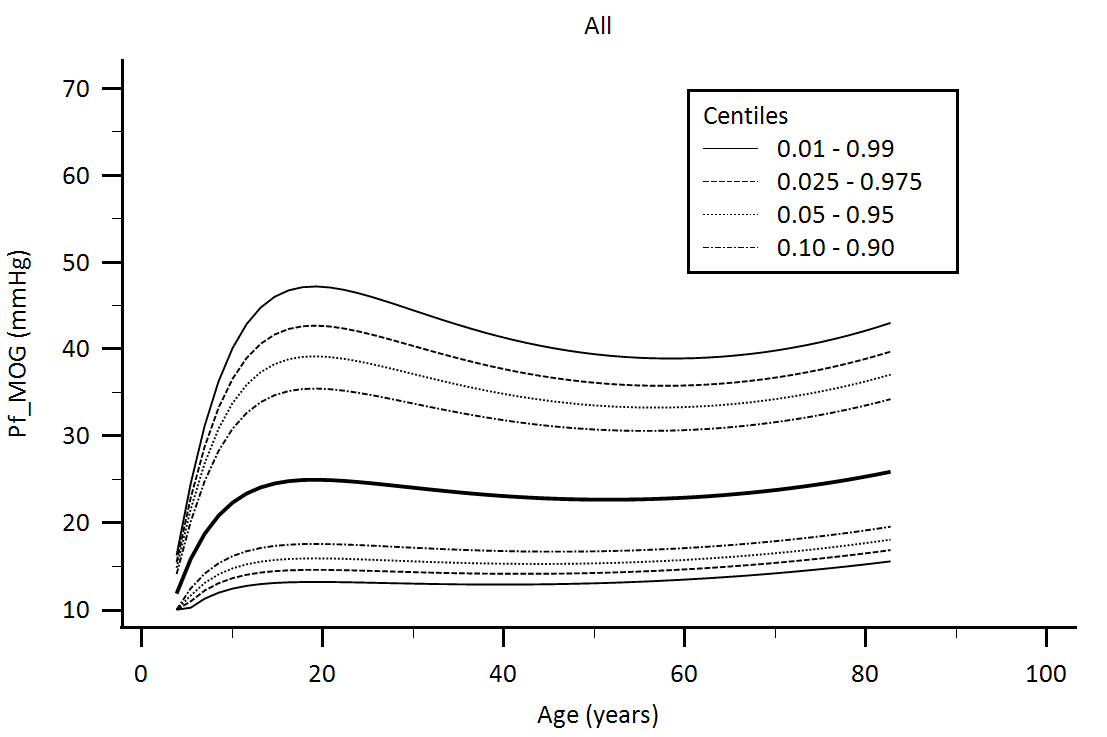

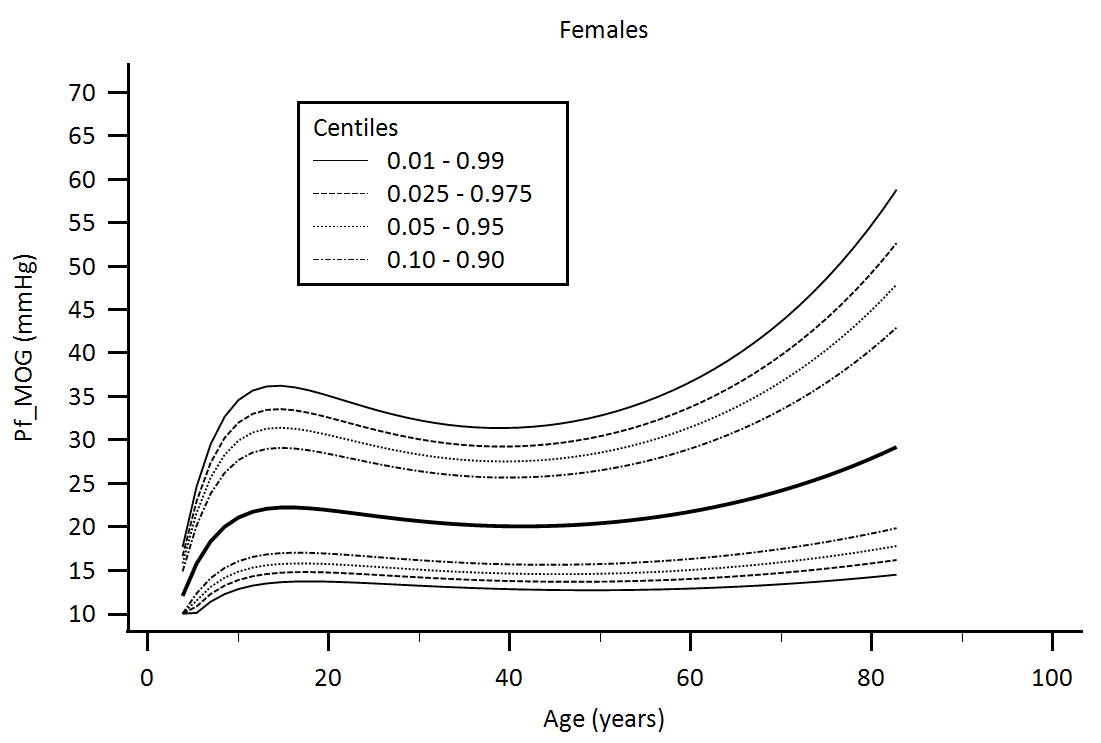


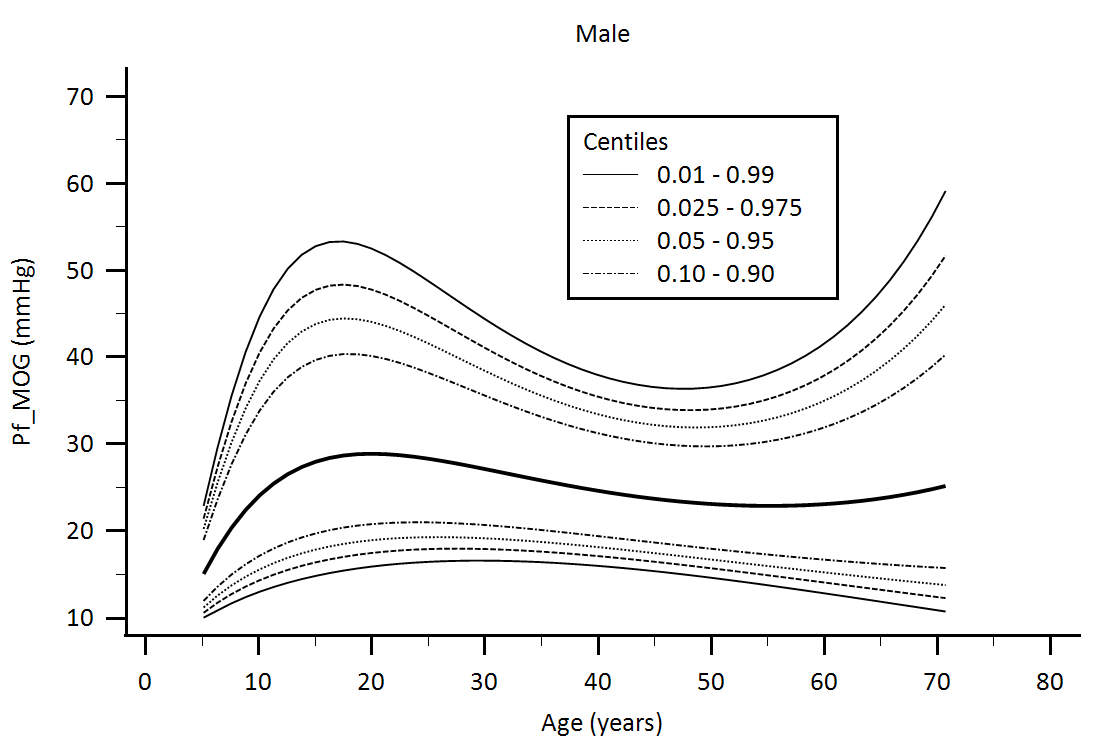


Supplementary Figure 4. Central aortic waveform-derived parameters obtained using Mobil-O-Graph device (MOG): Forward pressure (Pf) age-related percentiles.


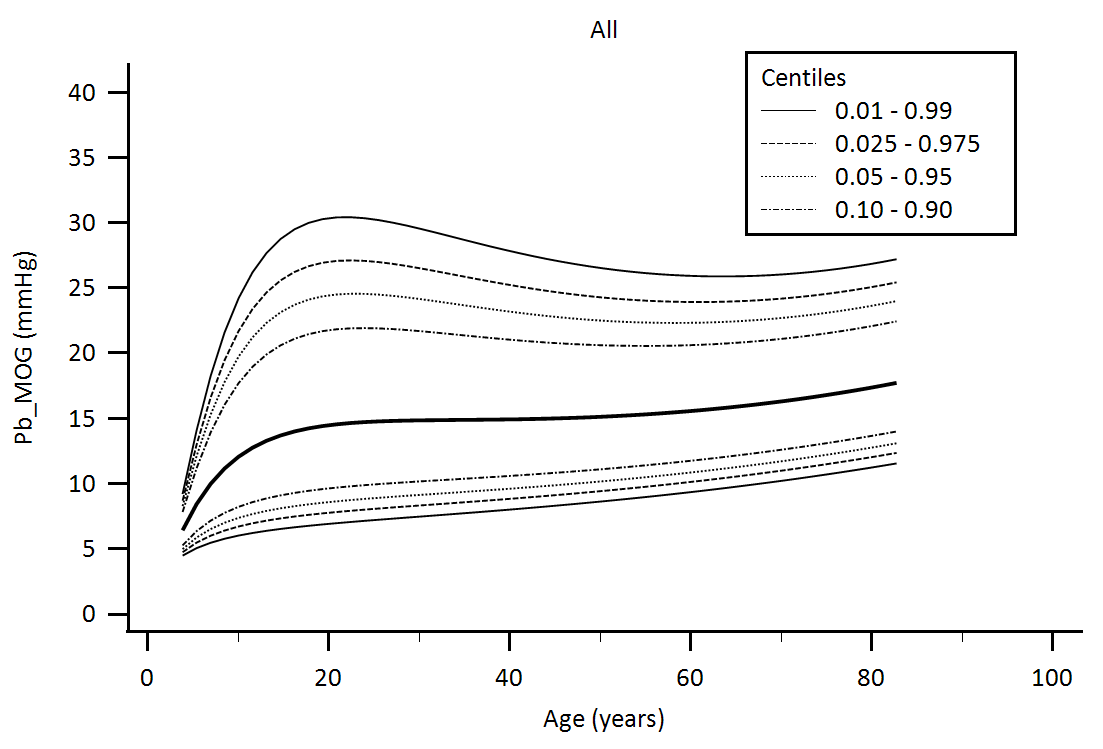


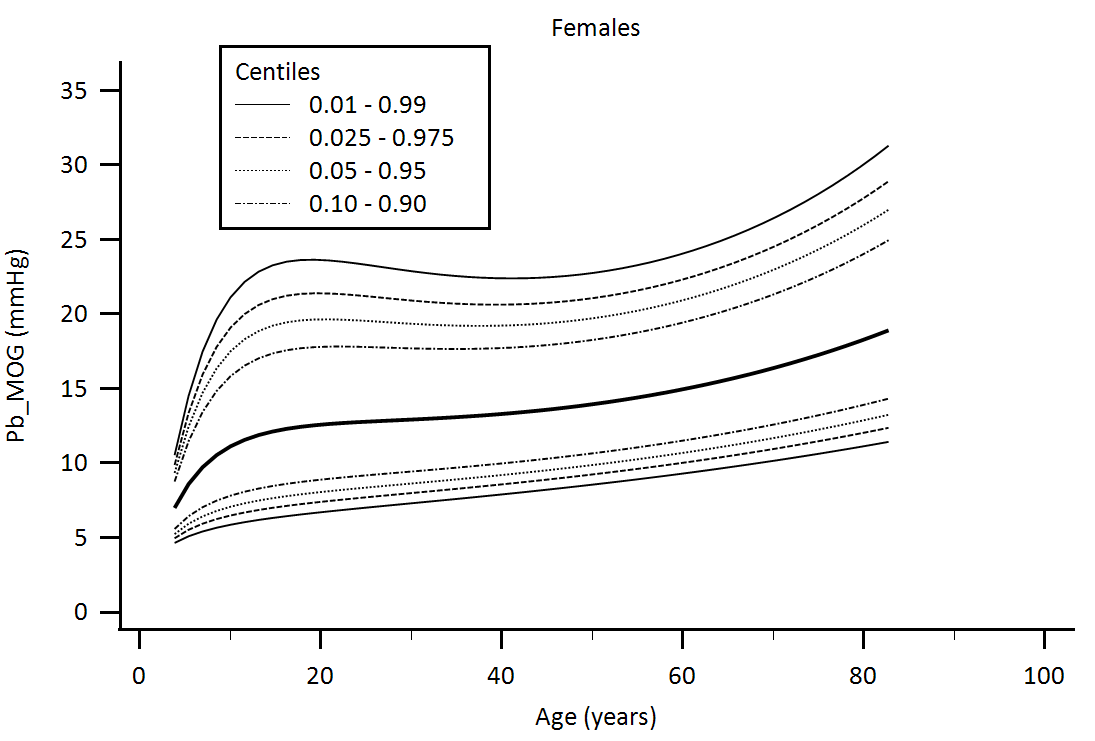


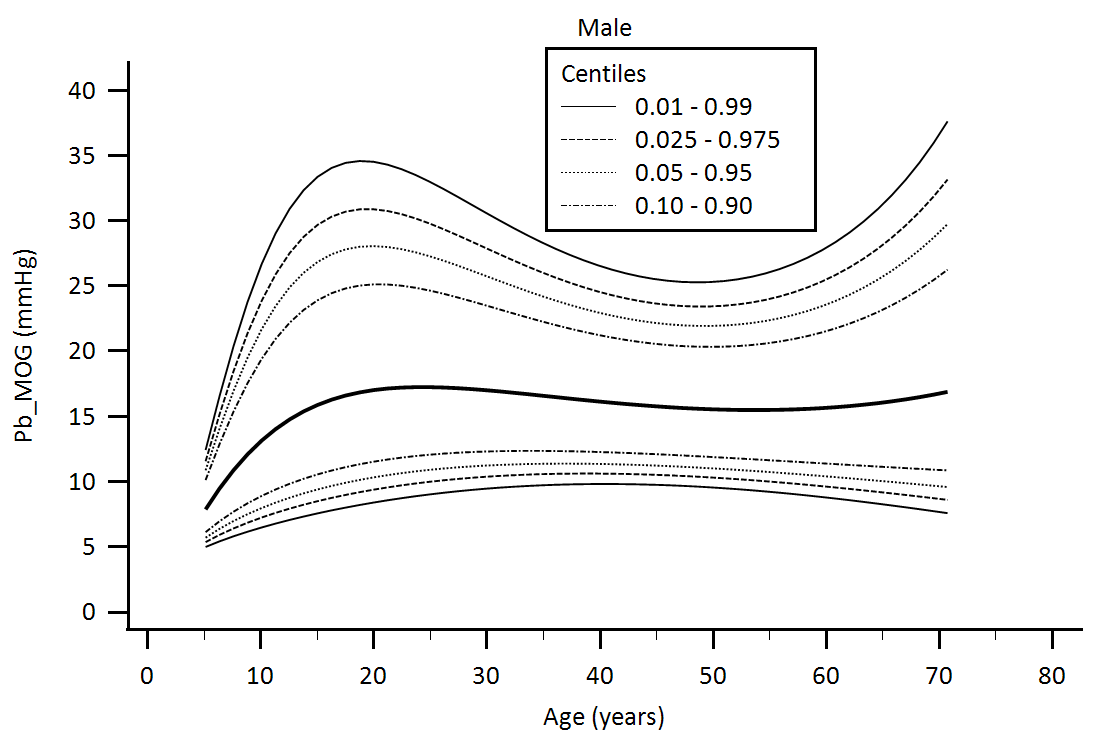


Supplementary Figure 5. Central aortic waveform-derived parameters obtained using Mobil-O-Graph device (MOG): Backward pressure (Pb) age-related percentiles.


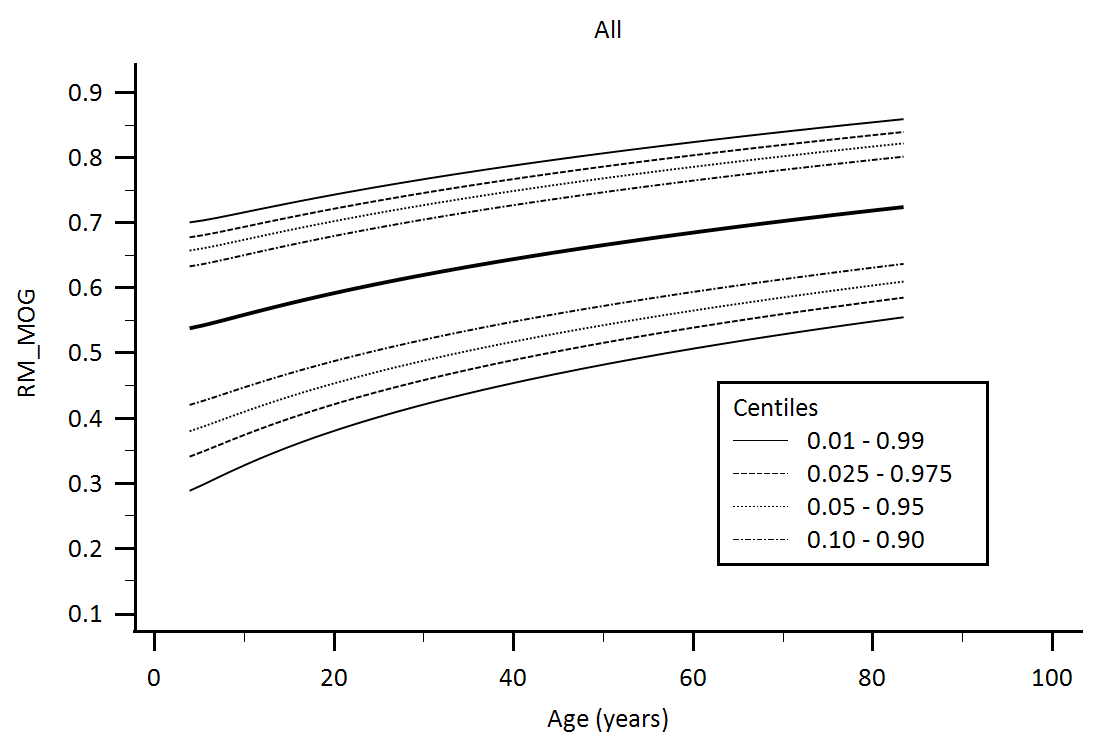


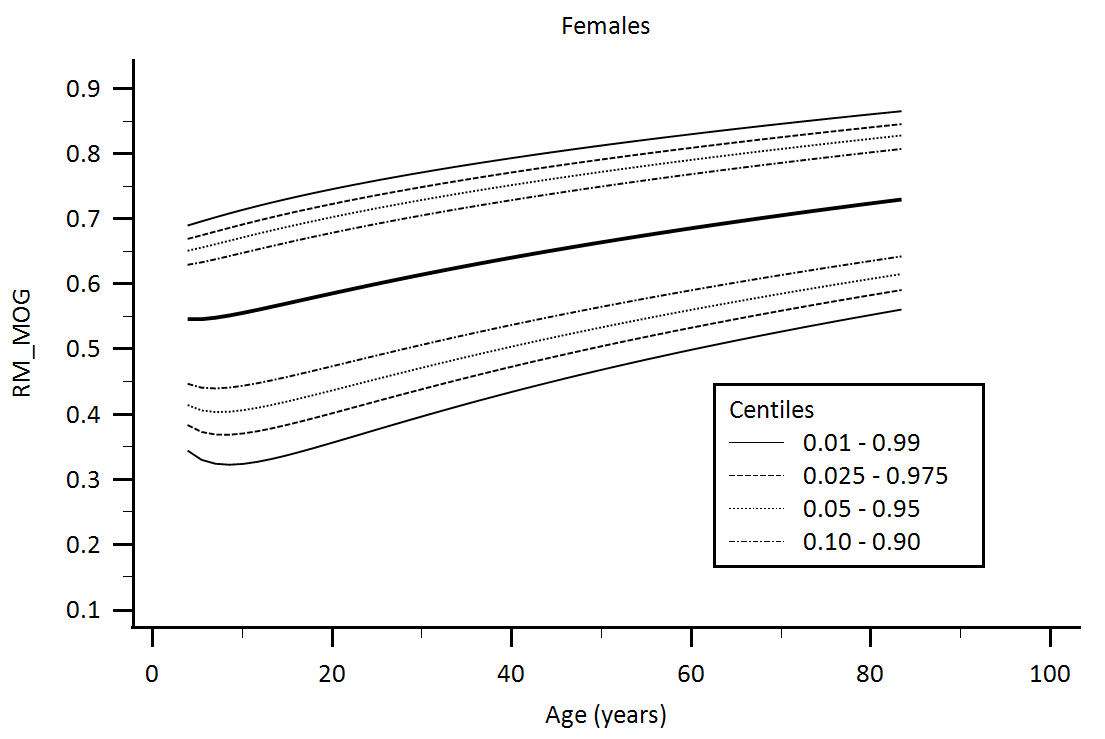


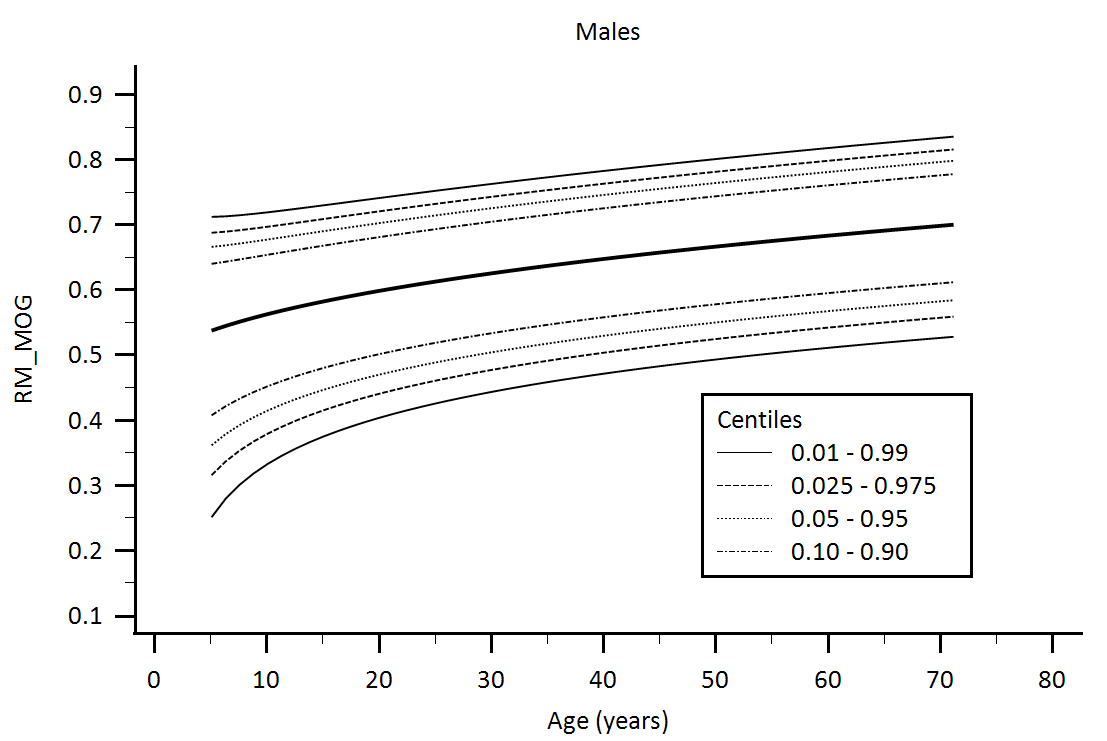


Supplementary Figure 6. Central aortic waveform-derived parameters obtained using Mobil-O-Graph device (MOG): Reflection Magnitude (RM) age-related percentiles.


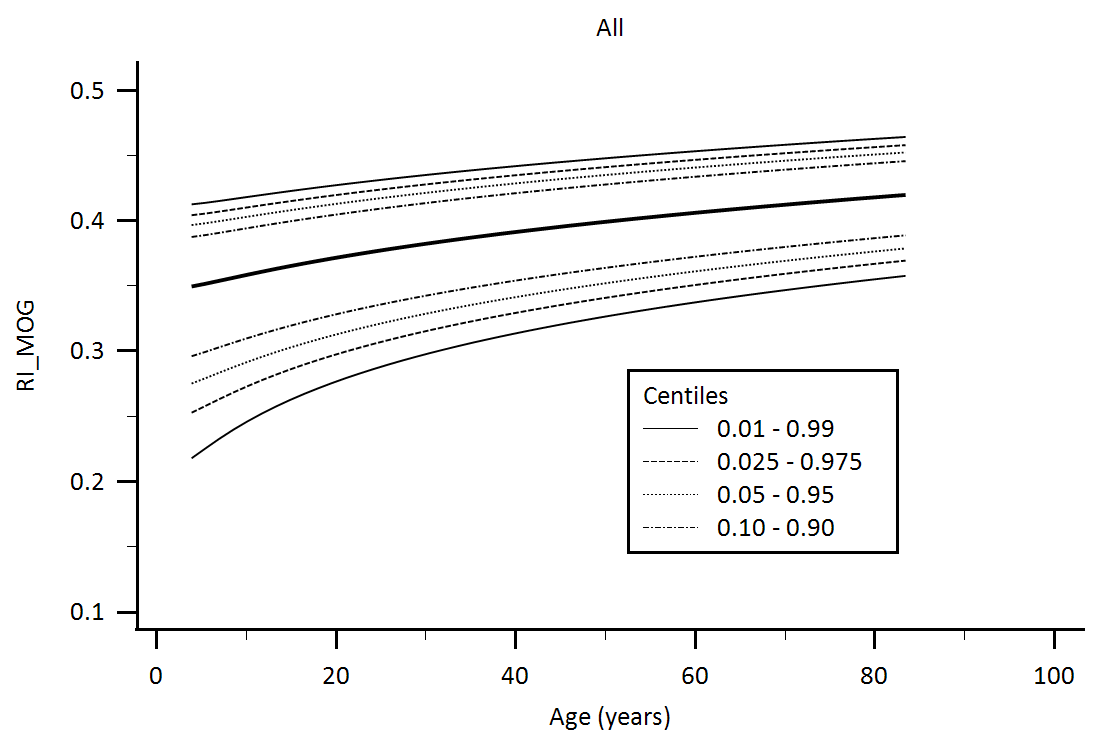


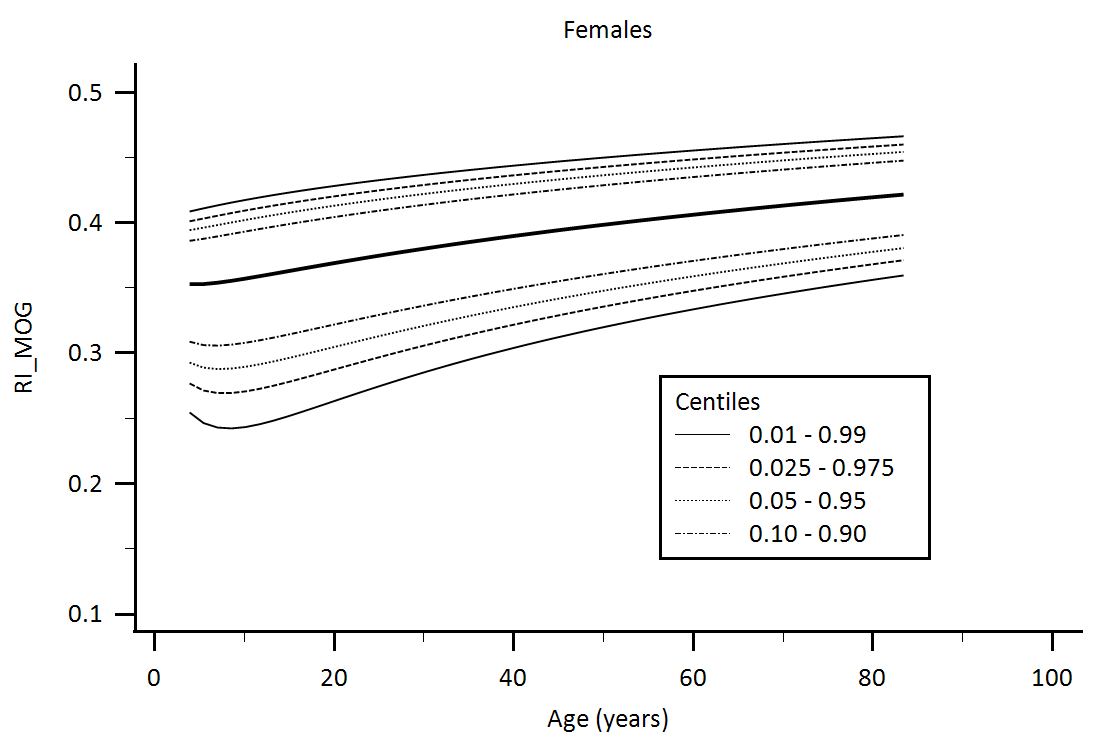

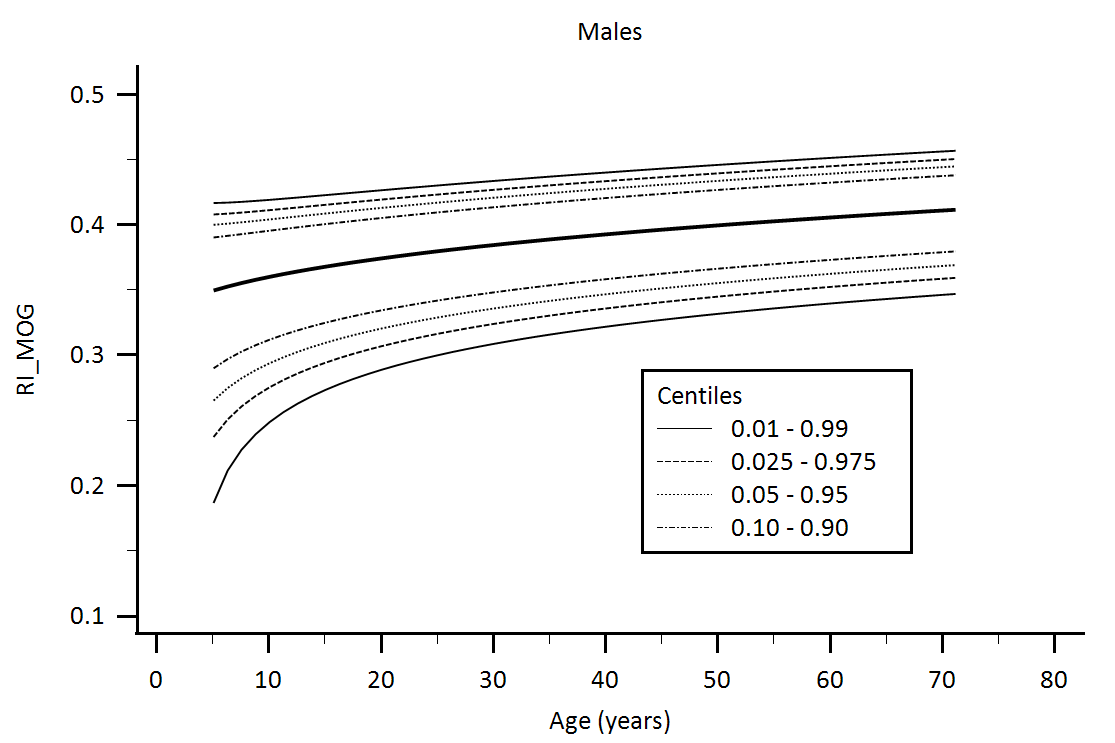


Supplementary Figure 7. Central aortic waveform-derived parameters obtained using Mobil-O-Graph device (MOG): Reflection Index (RI) age-related percentiles.

**Supplementary Figures: Central aortic waveform-derived parameters obtained using Radial Artery Applanation Tonometry using SphygmoCor device: Age-related percentiles**


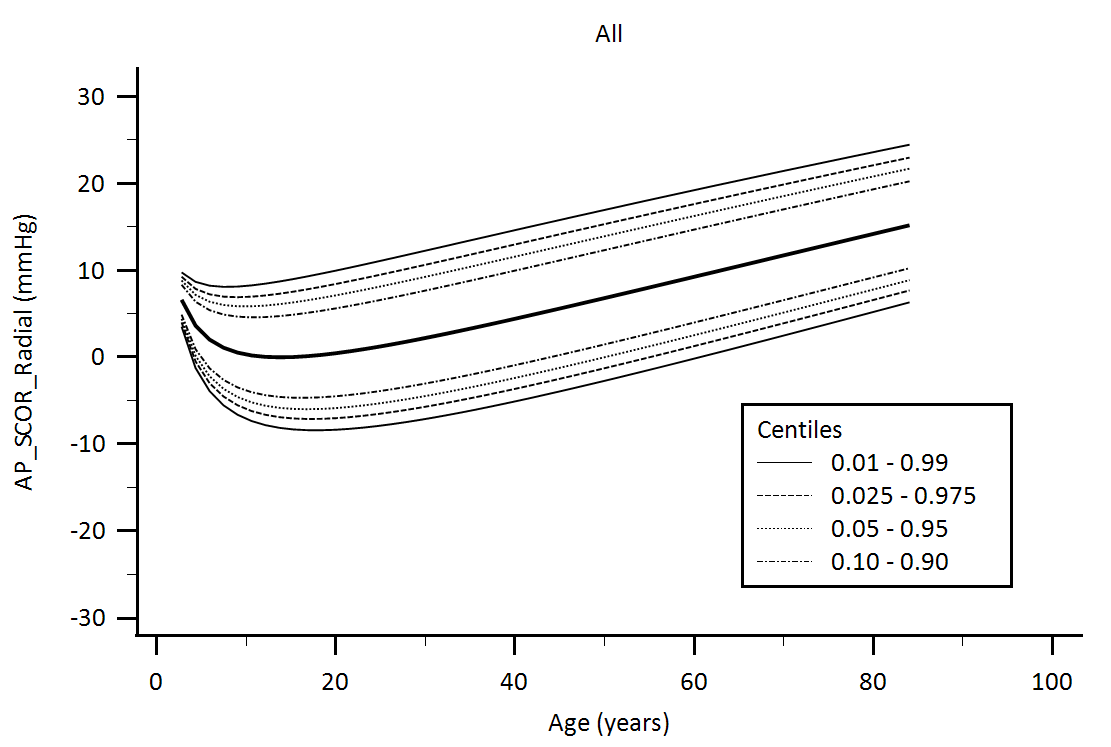

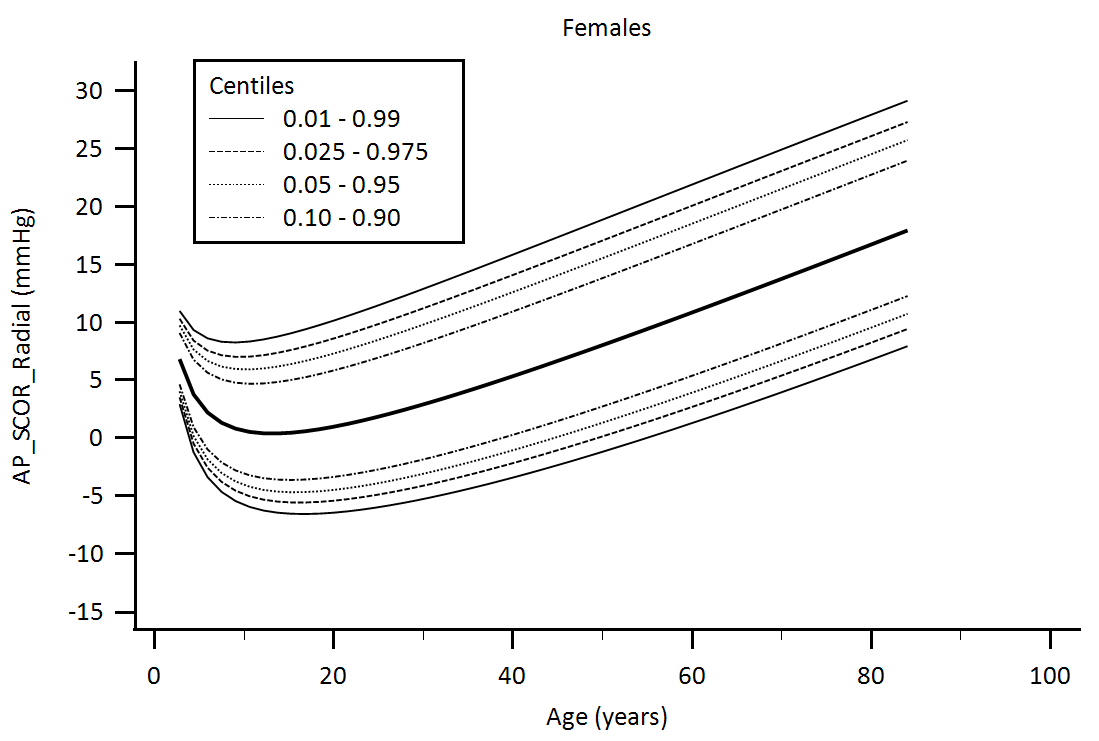

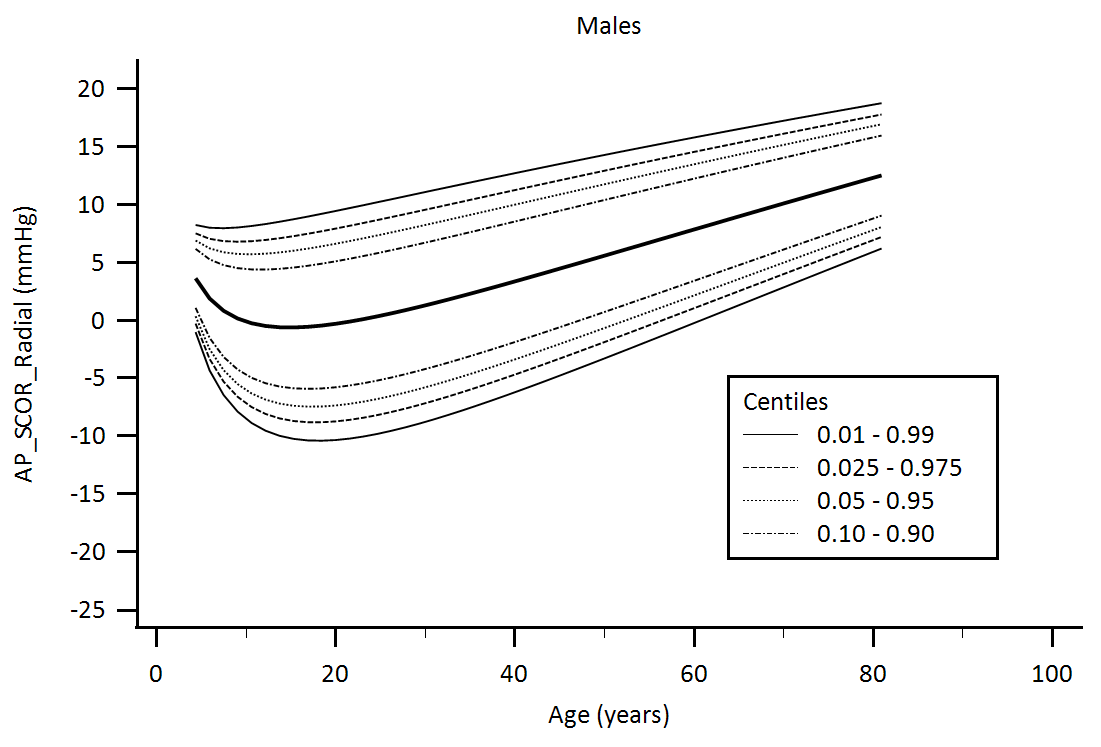


Supplementary Figure 8. Central aortic waveform-derived parameters obtained using radial artery applanation tonometry (SphygmoCor device, SCOR): Augmentation pressure (AP) age-related percentiles.


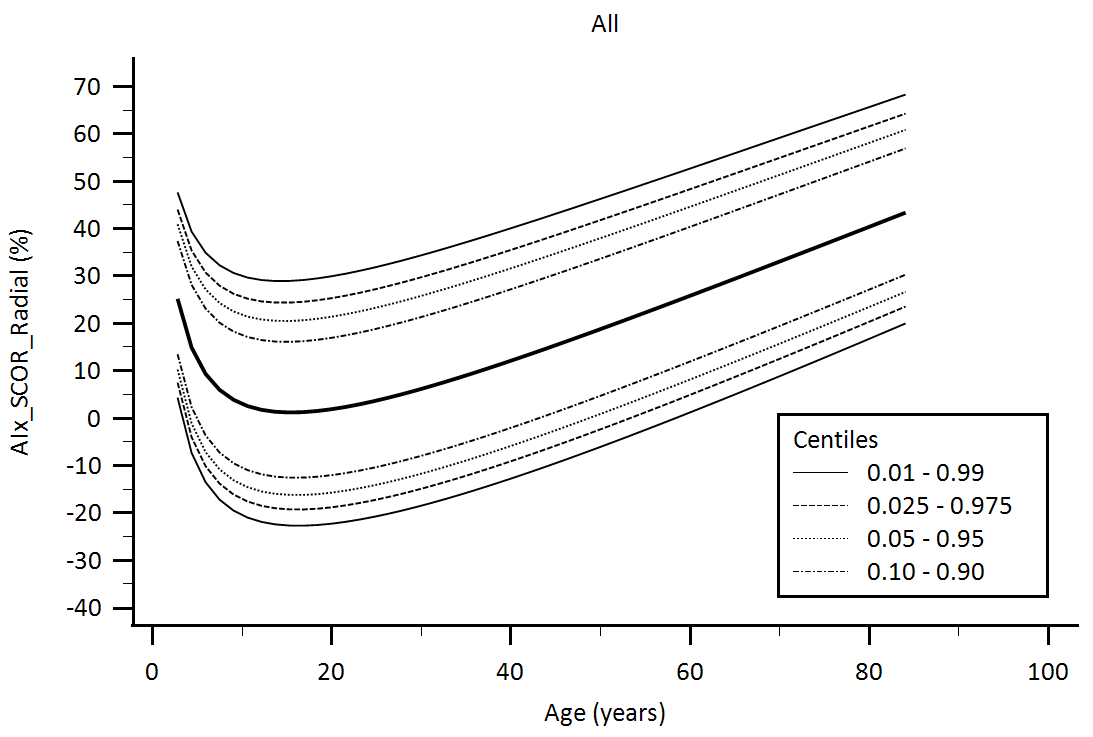


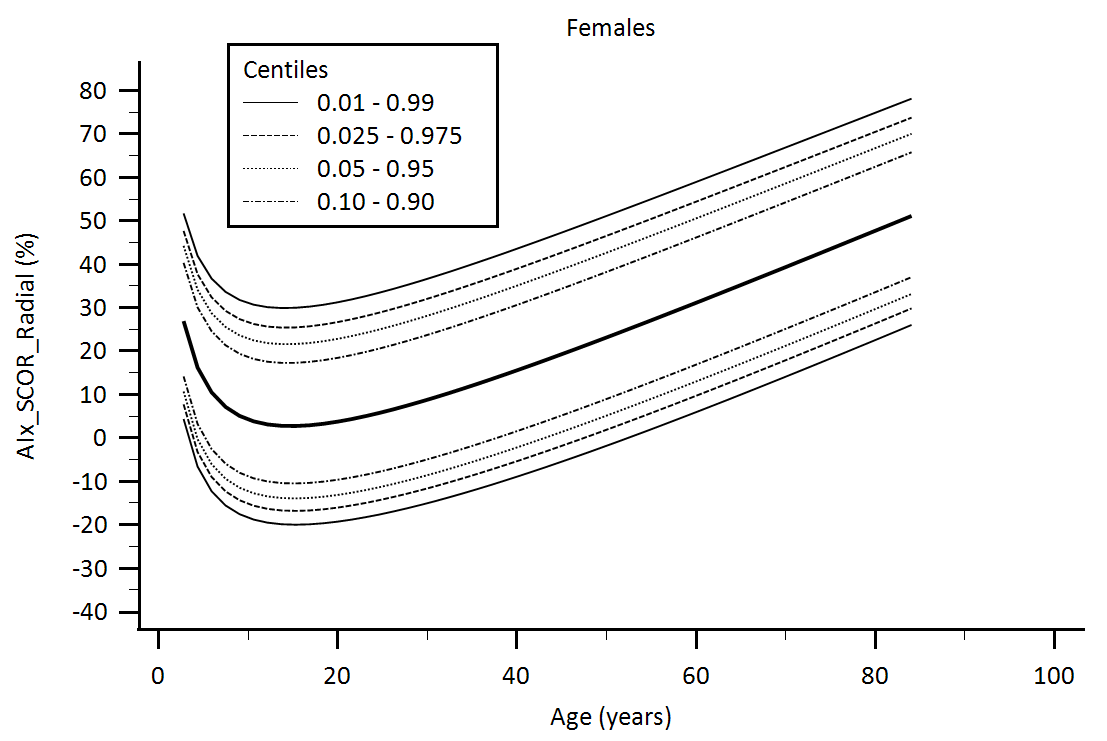


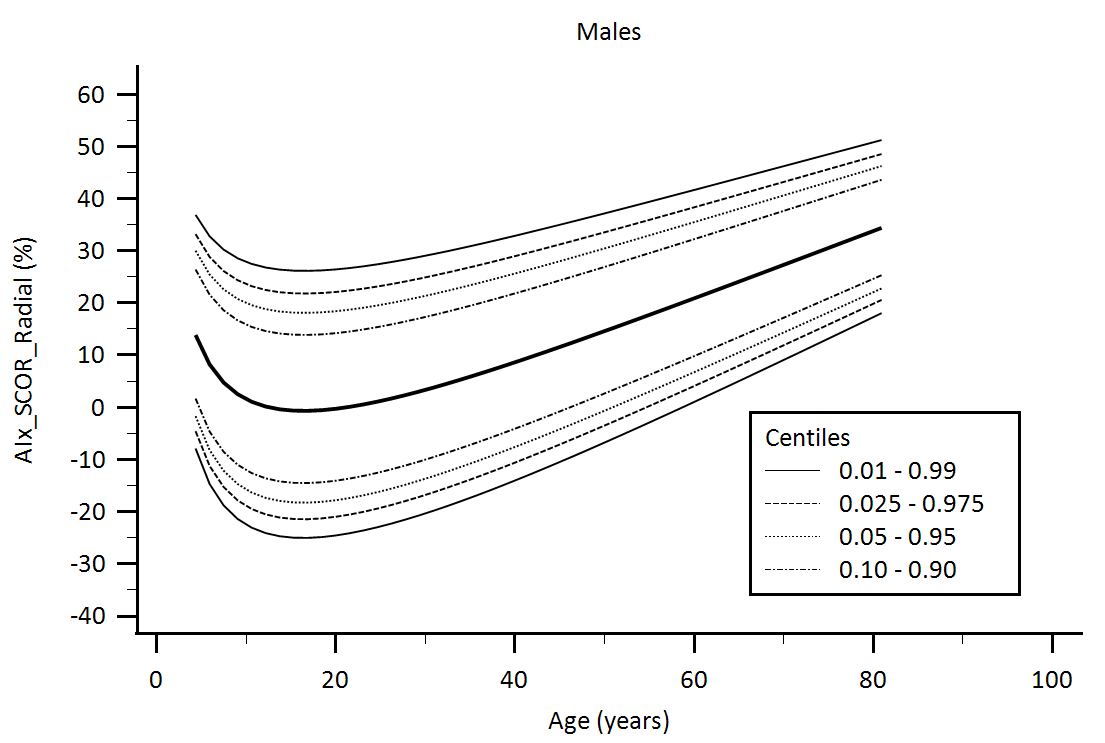


Supplementary Figure 9. Central aortic waveform-derived parameters obtained using radial artery applanation tonometry (SphygmoCor device, SCOR): Augmentation Index (AIx) age-related percentiles.


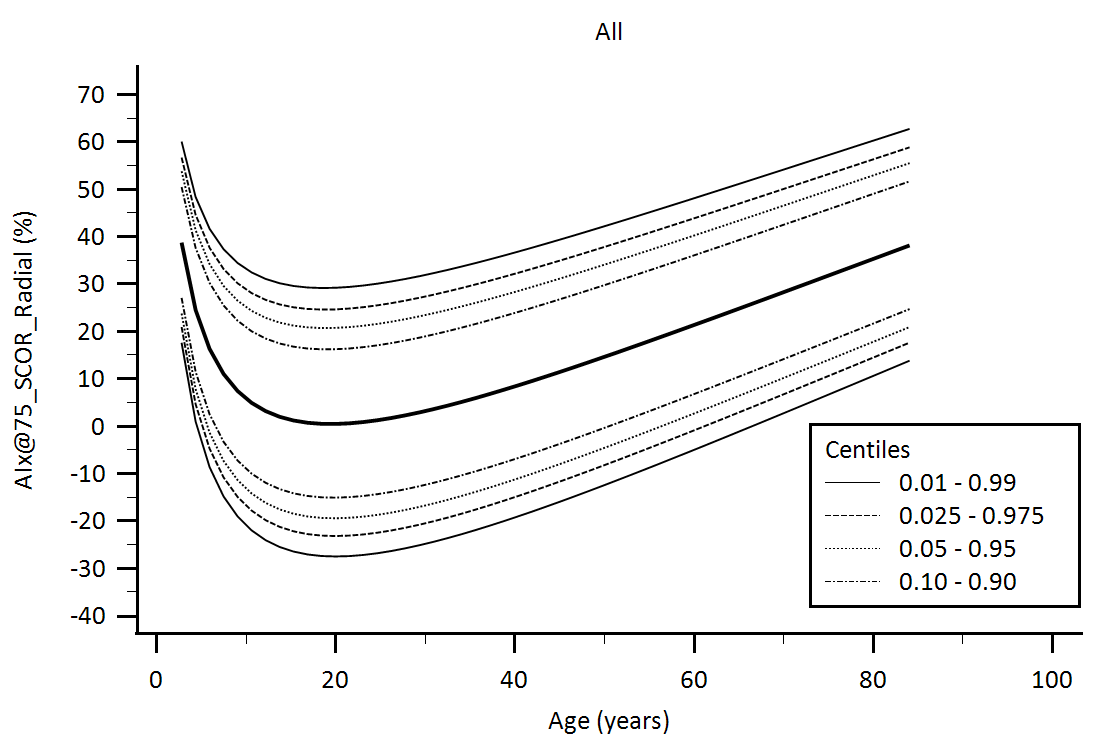


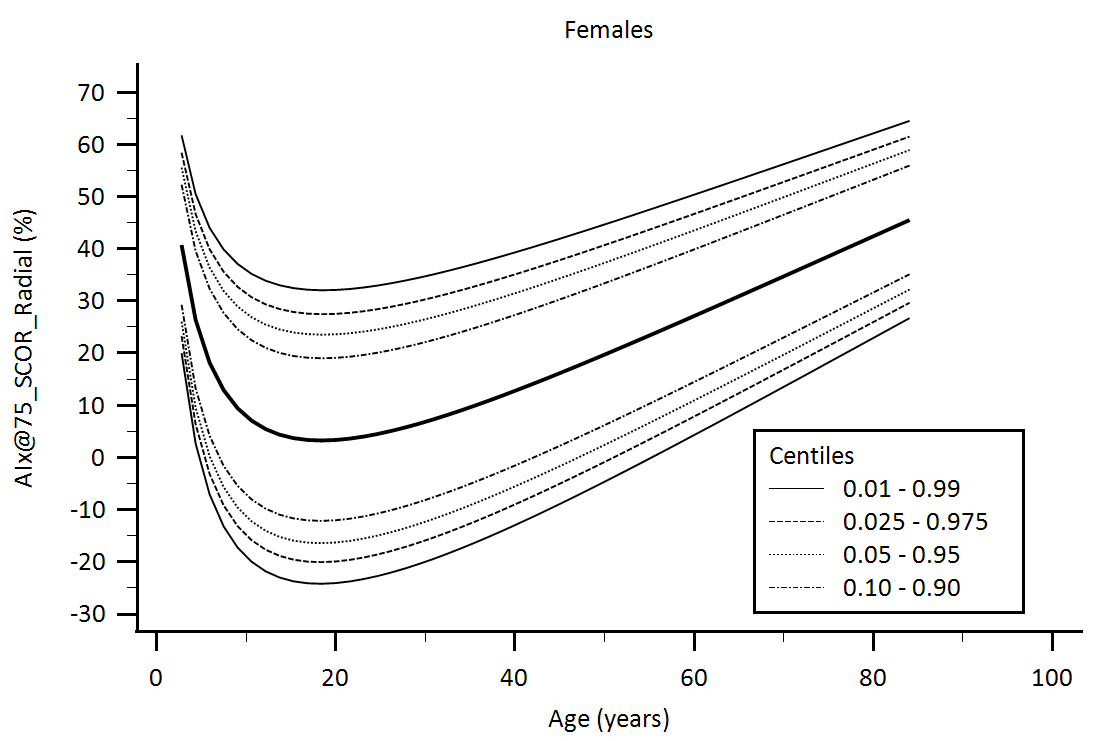


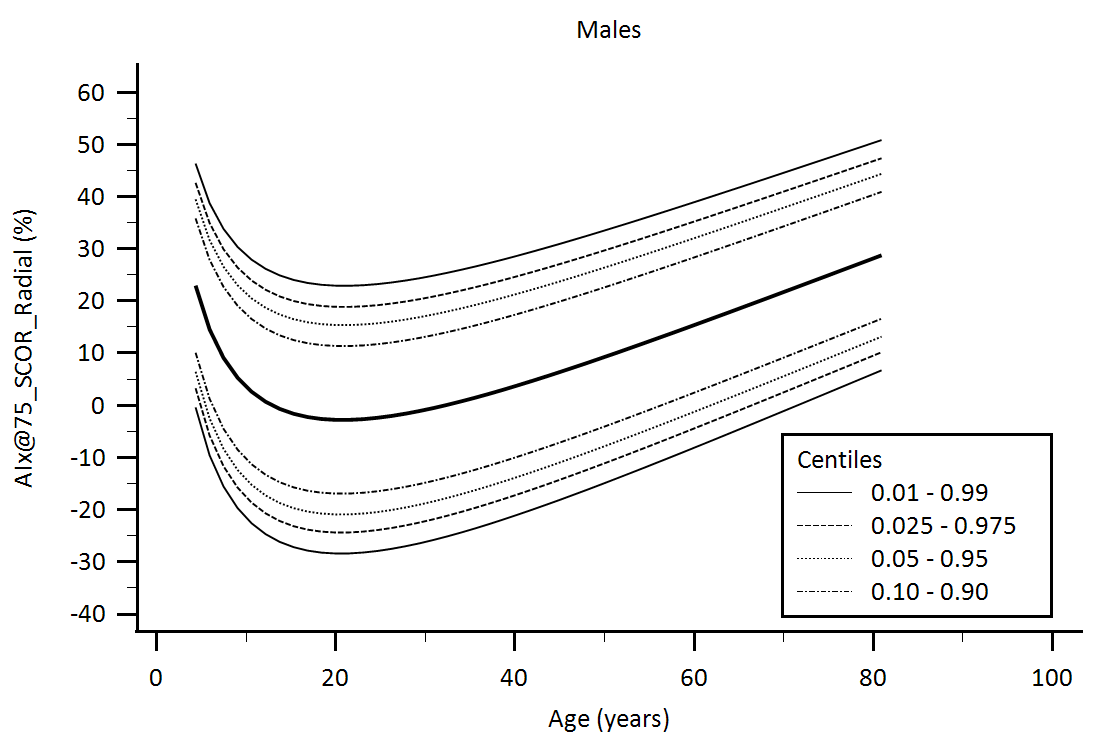


Supplementary Figure 10. Central aortic waveform-derived parameters obtained using radial artery applanation tonometry (SphygmoCor device, SCOR): Augmentation Index adjusted for heart rate equal 75 beats/minute (AIx@75) age-related percentiles.


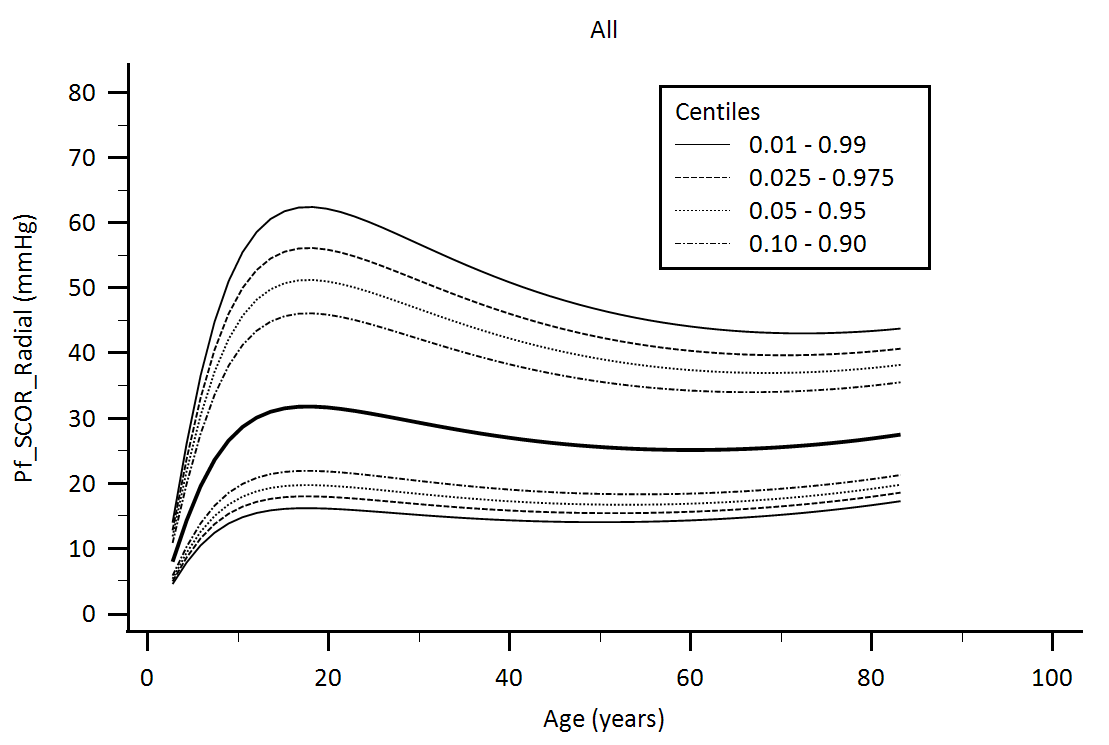

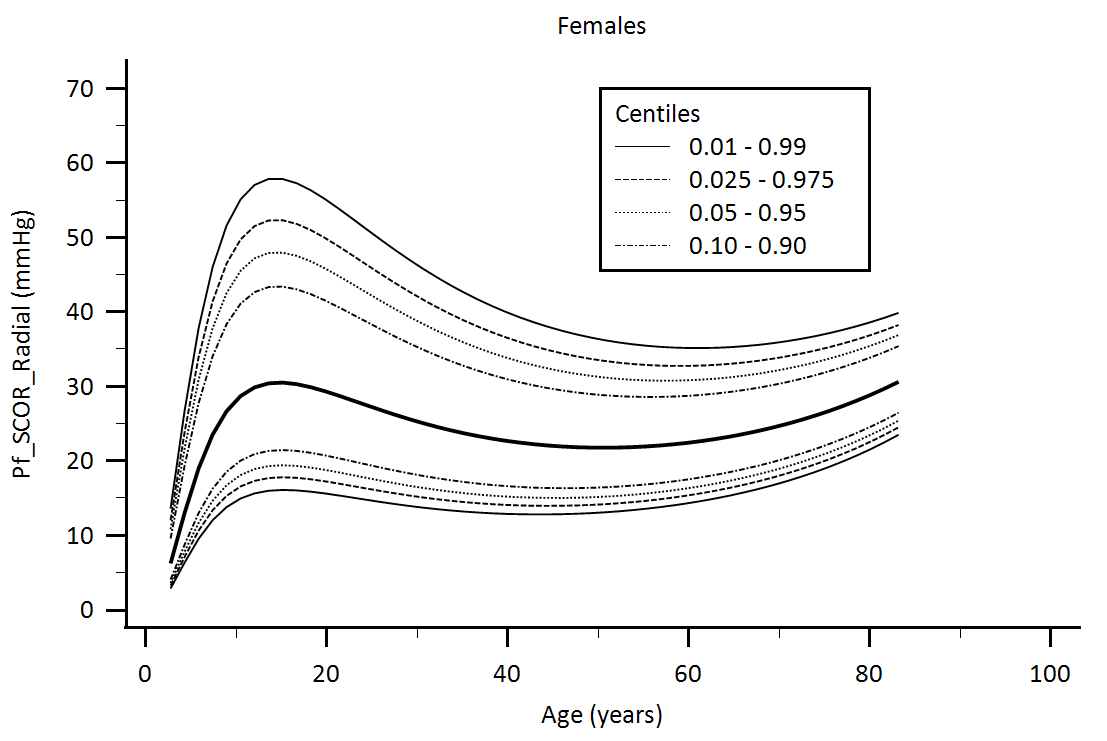


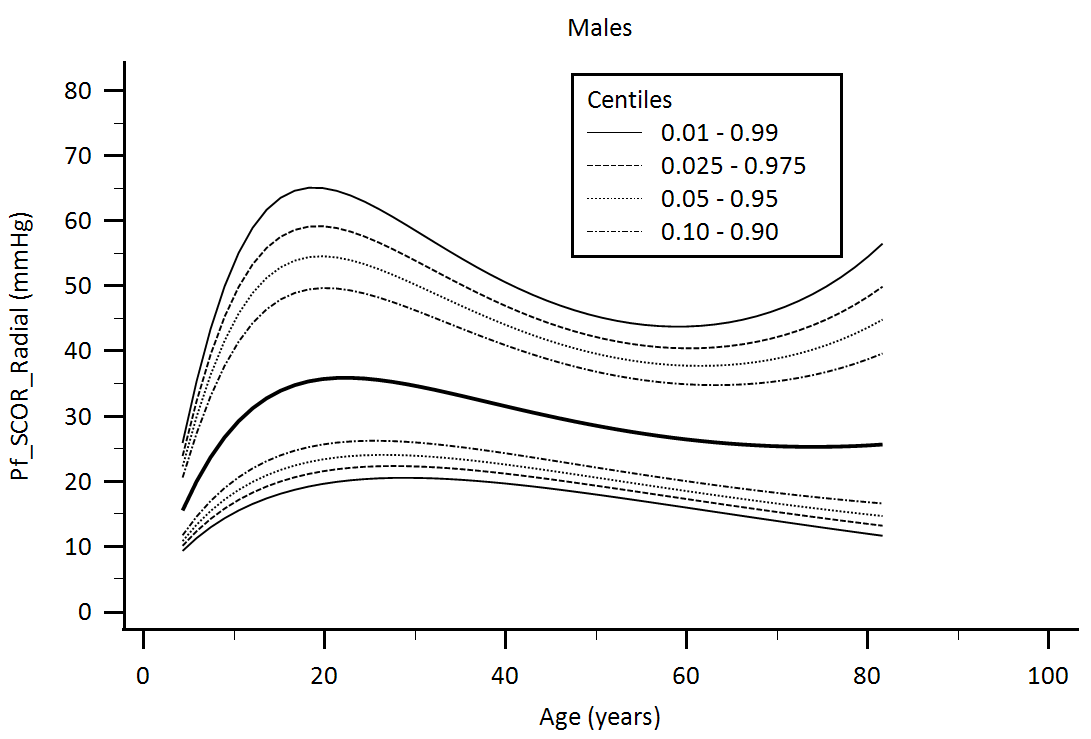


Supplementary Figure 11. Central aortic waveform-derived parameters obtained using radial artery applanation tonometry (SphygmoCor device, SCOR): Forward pressure (Pf) age-related percentiles.


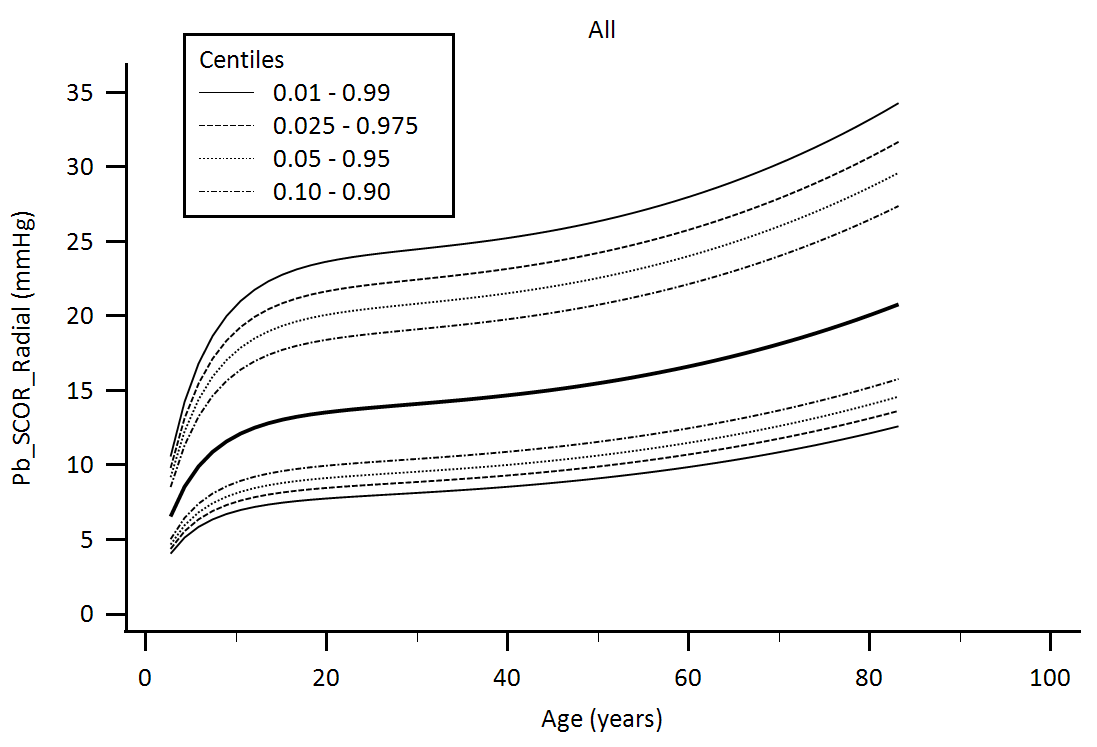

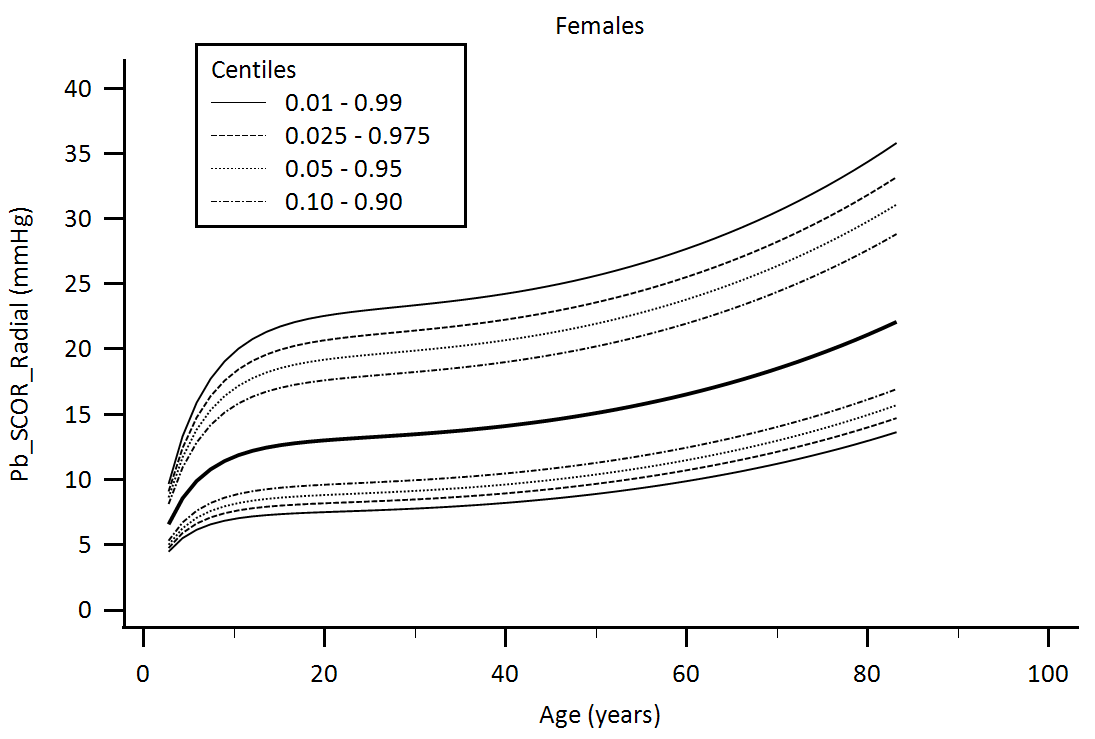

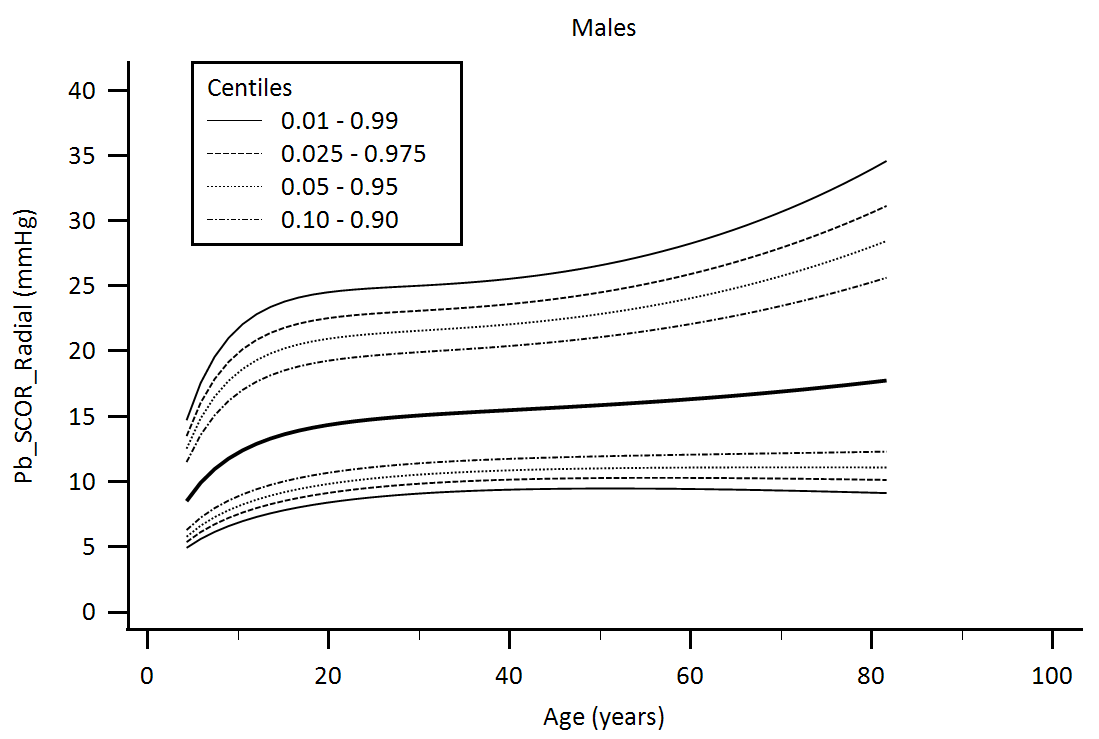


Supplementary Figure 12. Central aortic waveform-derived parameters obtained using radial artery applanation tonometry (SphygmoCor device, SCOR): Backward pressure (Pb) age-related percentiles.


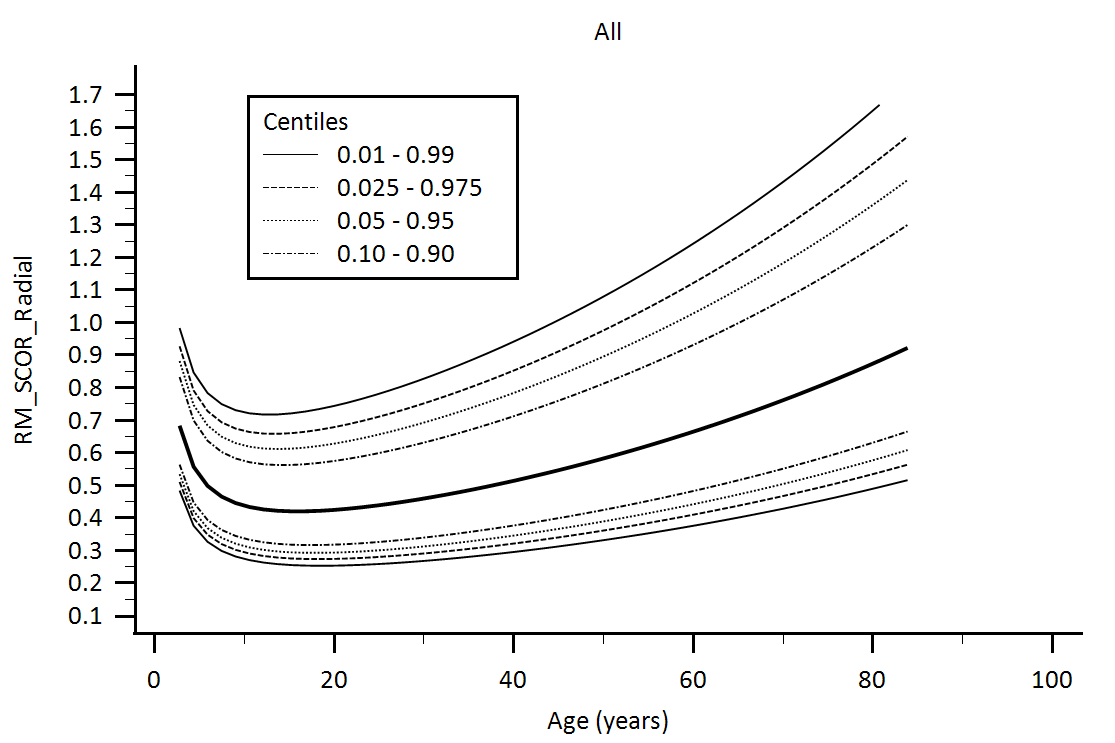

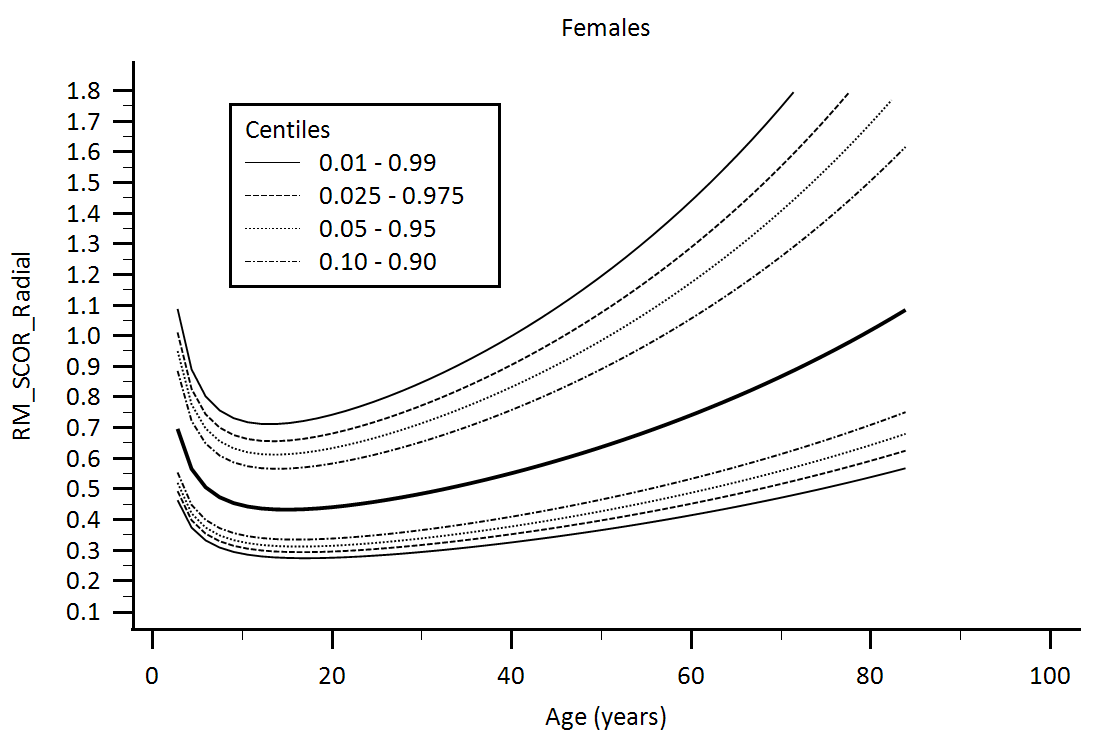

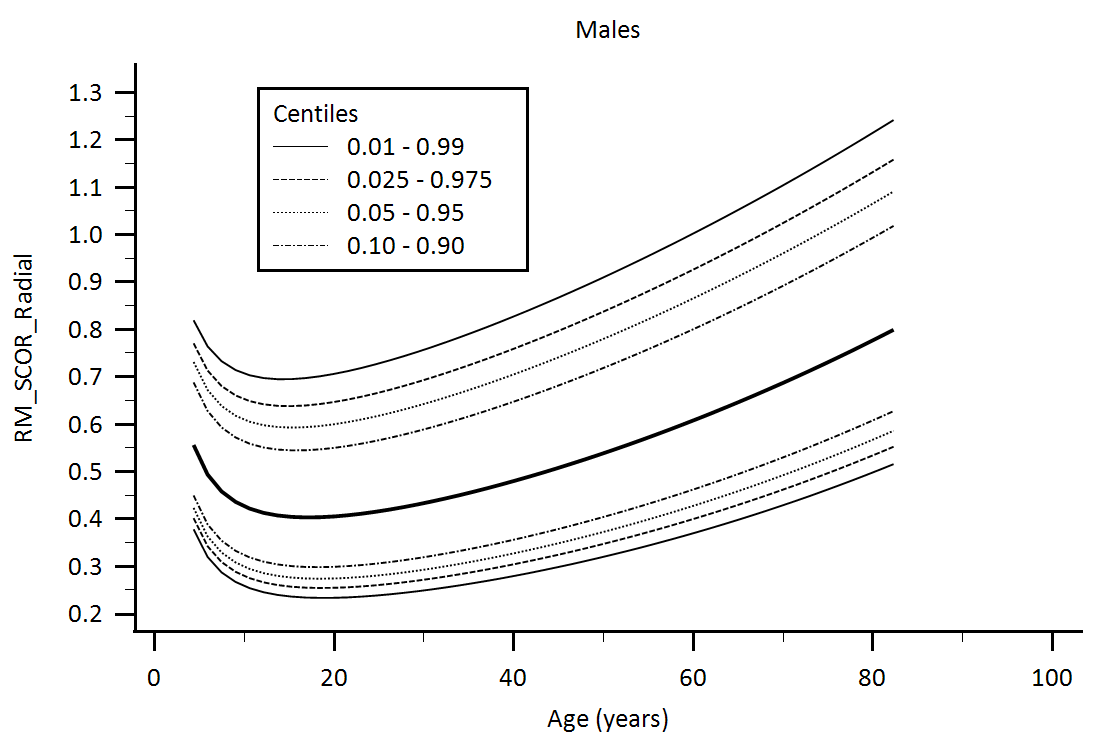


Supplementary Figure 13. Central aortic waveform-derived parameters obtained using radial artery applanation tonometry (SphygmoCor device, SCOR): Reflection Magnitude (RM) age-related percentiles.


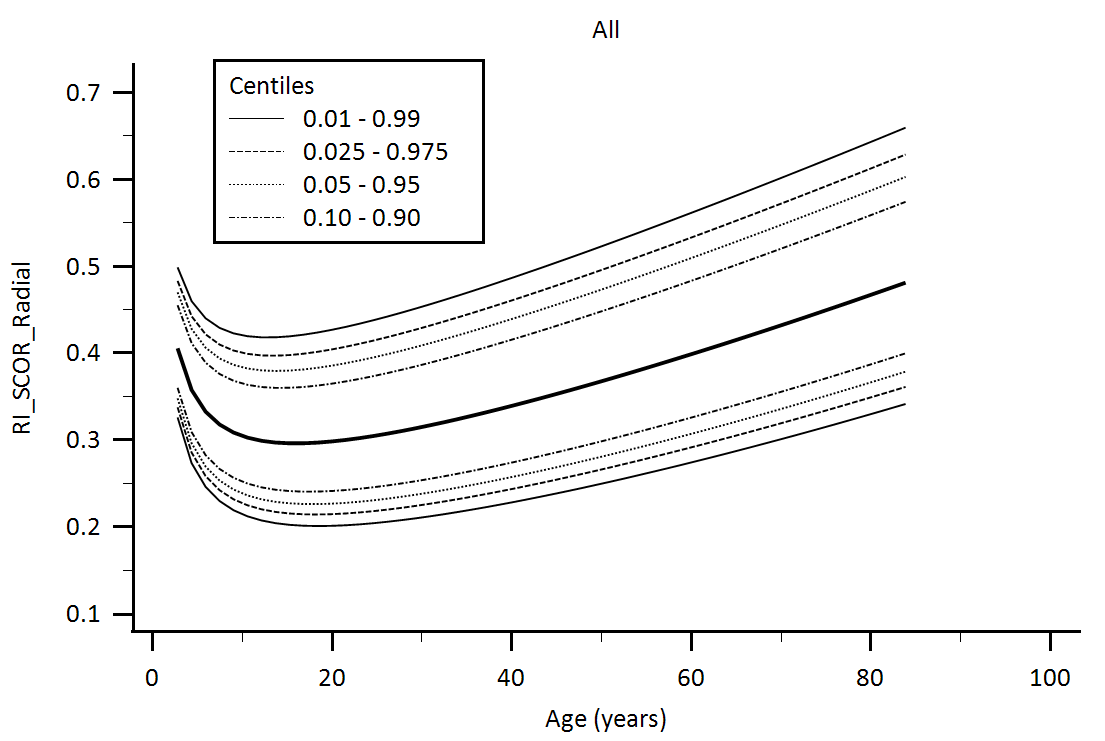


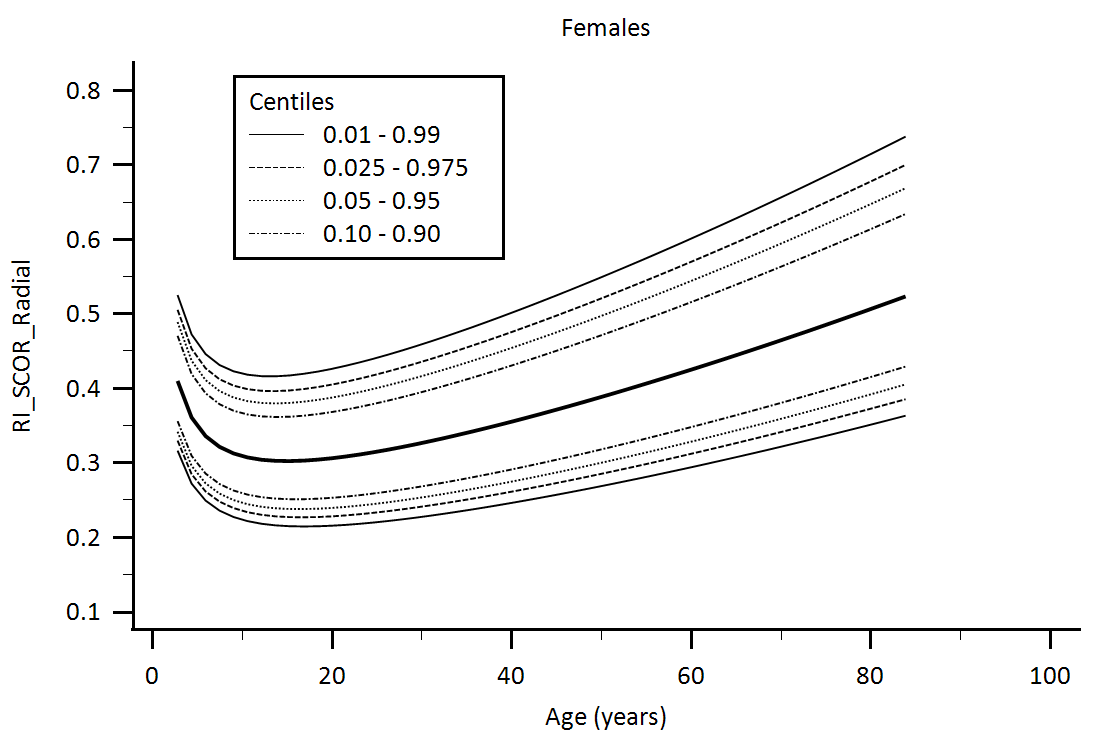

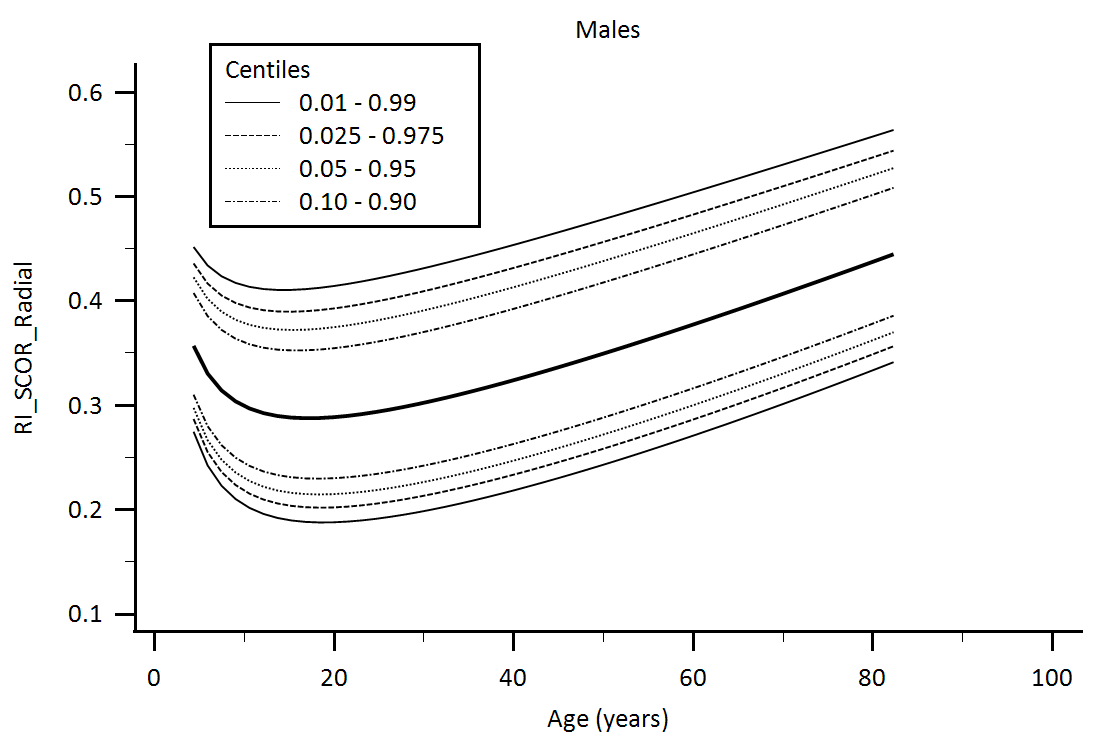


Supplementary Figure 14. Central aortic waveform-derived parameters obtained using radial artery applanation tonometry (SphygmoCor device, SCOR): Reflection Index (RI) age-related percentiles.

**Supplementary Figures: Central aortic waveform-derived parameters obtained using Carotid Artery Applanation Tonometry using SphygmoCor device: Age-related percentiles**


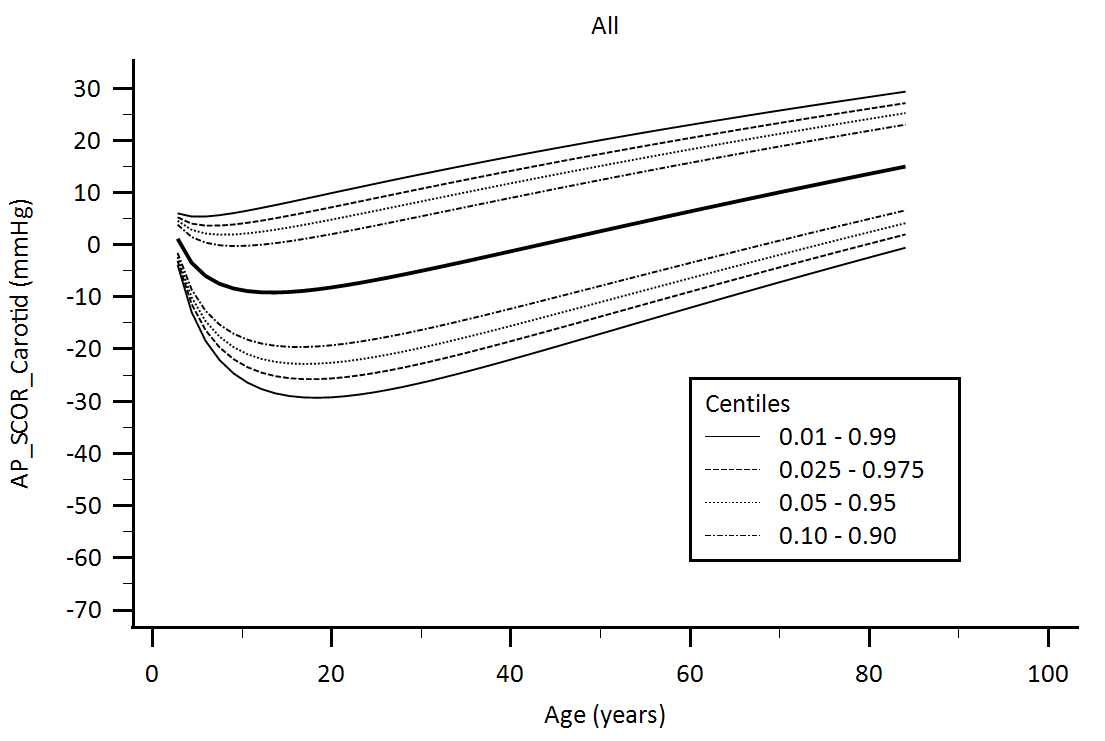


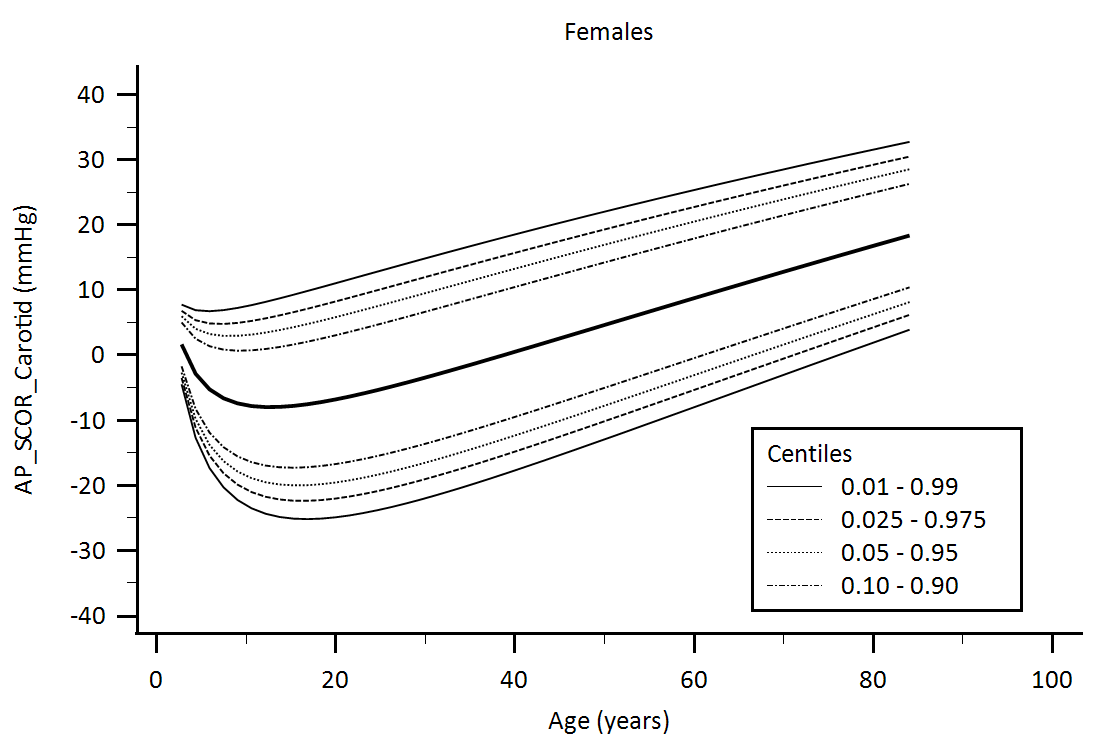


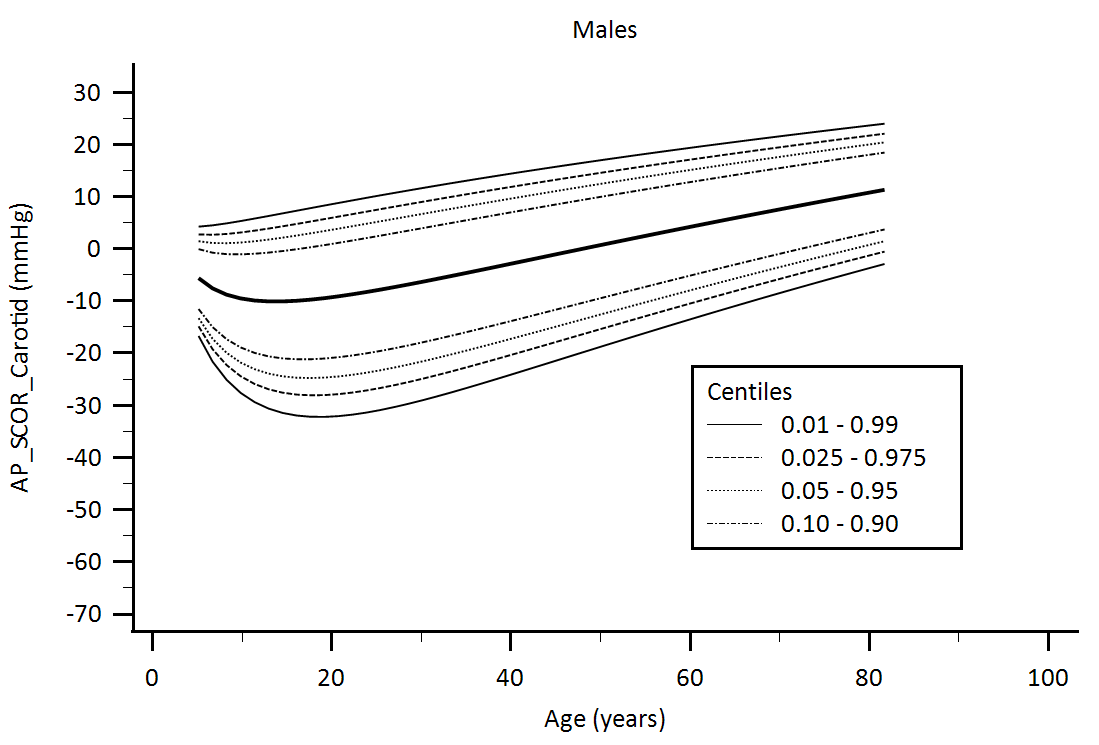


Supplementary Figure 15. Central aortic waveform-derived parameters obtained using carotid artery applanation tonometry (SphygmoCor device, SCOR): Augmentation pressure (AP) age-related percentiles.


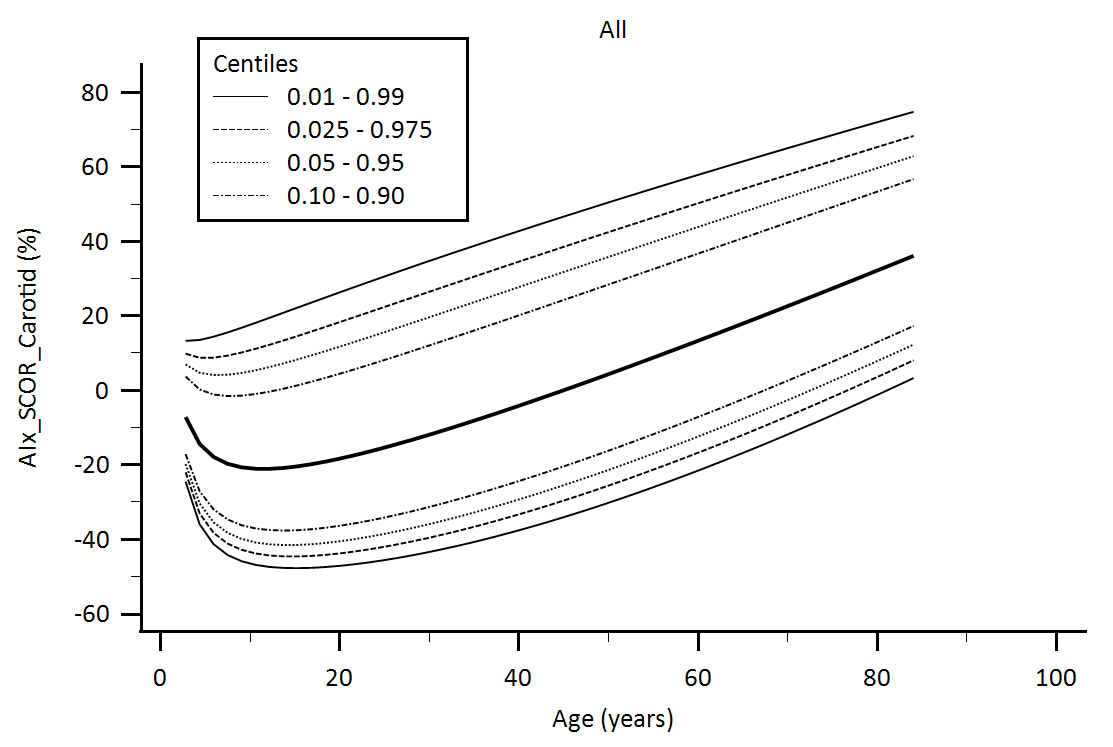

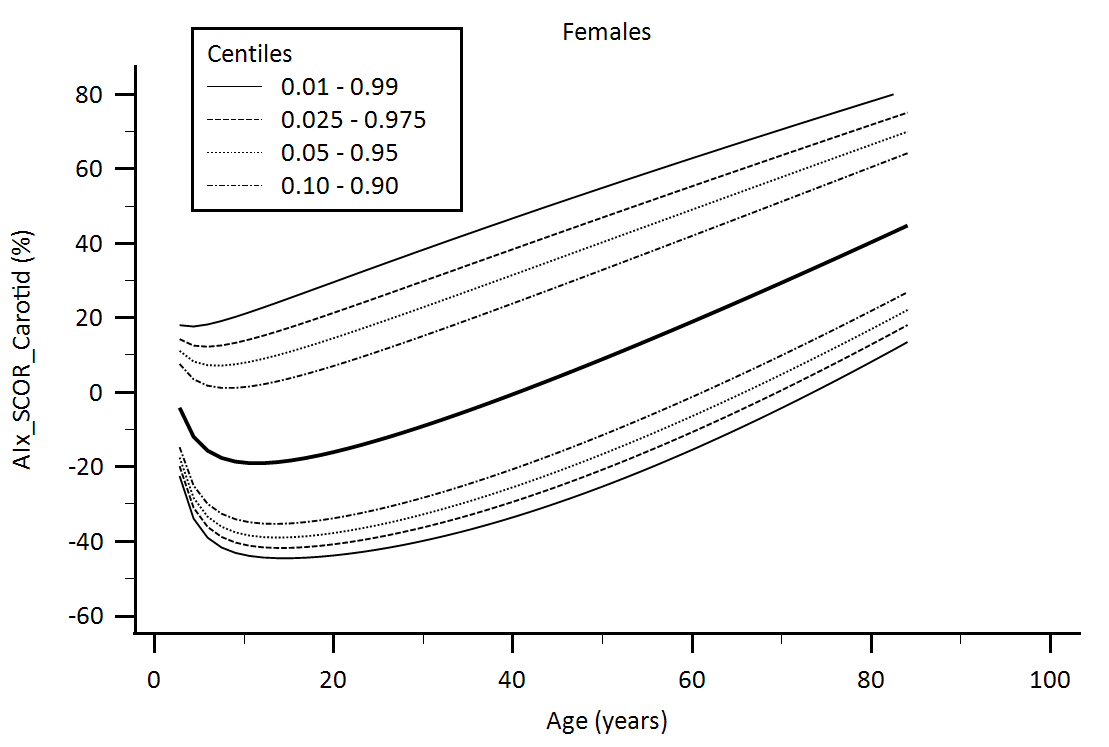

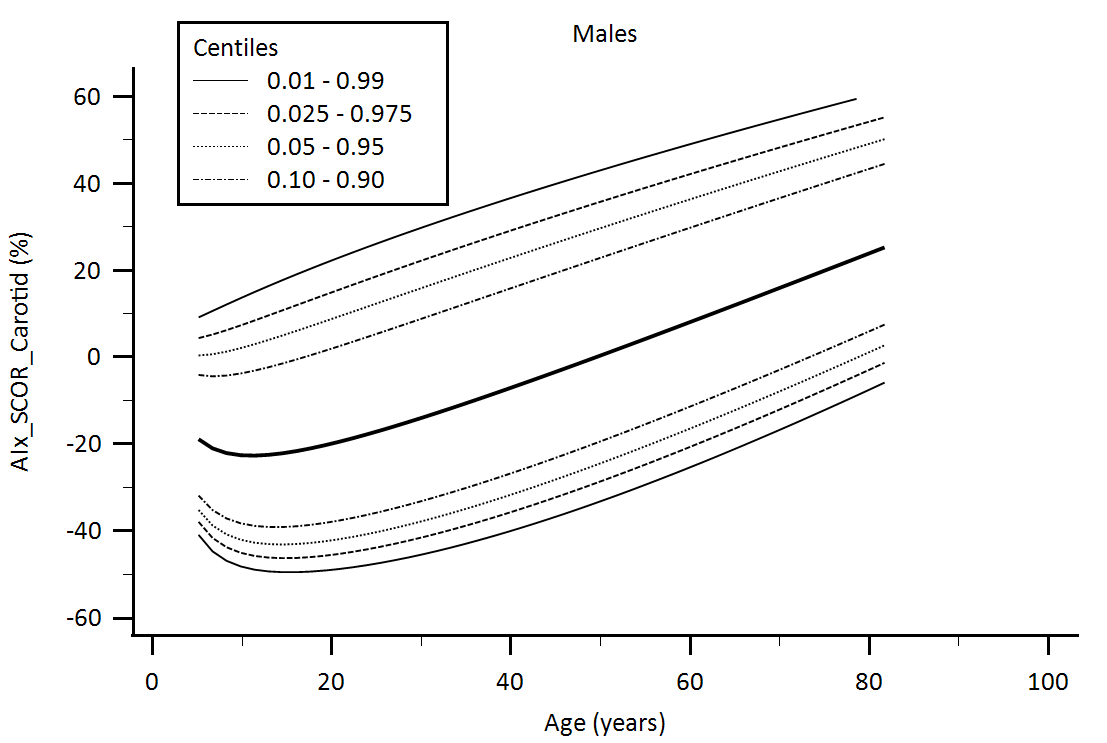


Supplementary Figure 16. Central aortic waveform-derived parameters obtained using carotid artery applanation tonometry (SphygmoCor device, SCOR): Augmentation Index (AIx) age-related percentiles.


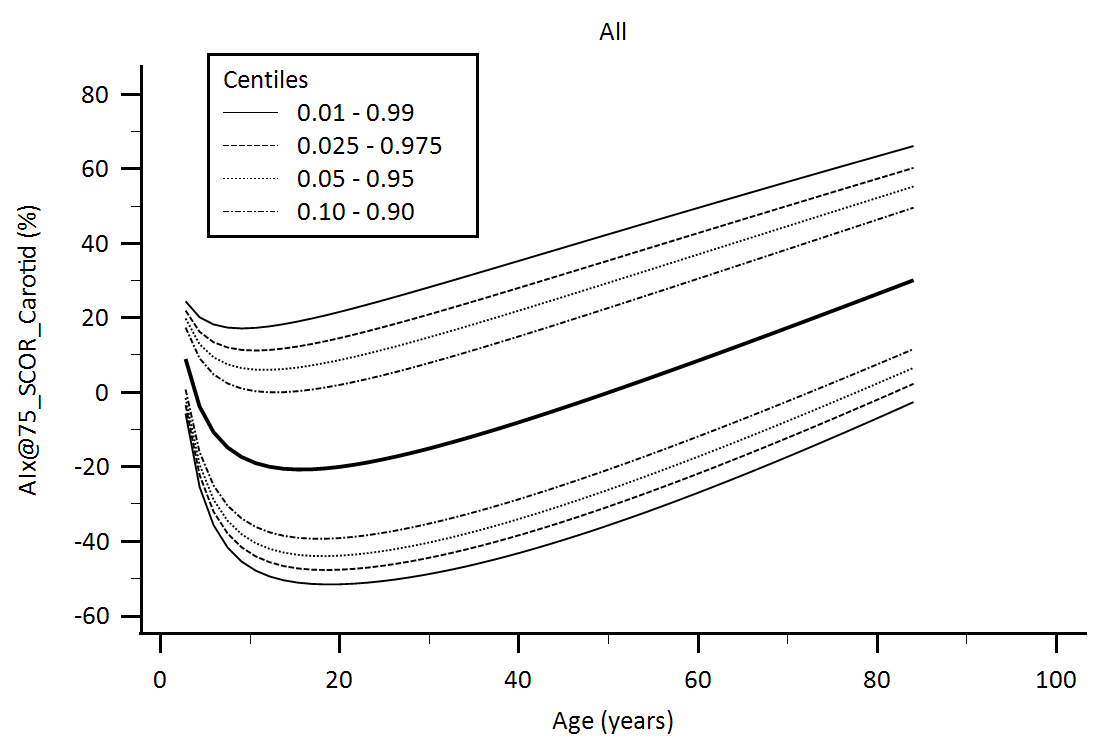

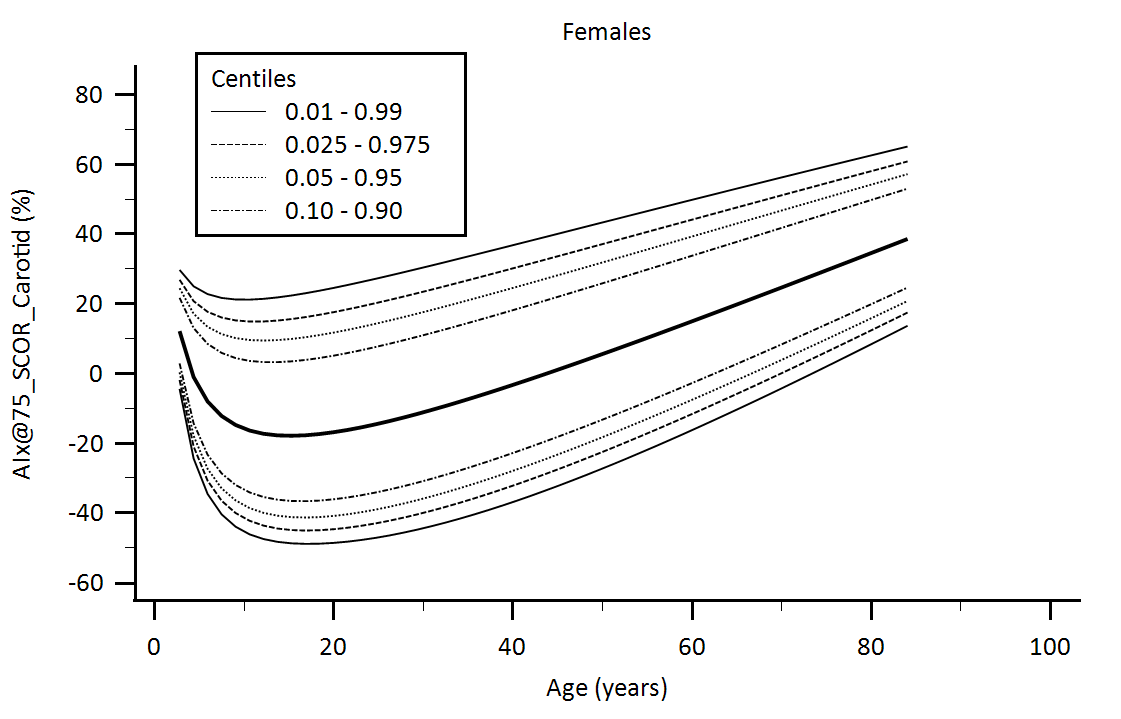

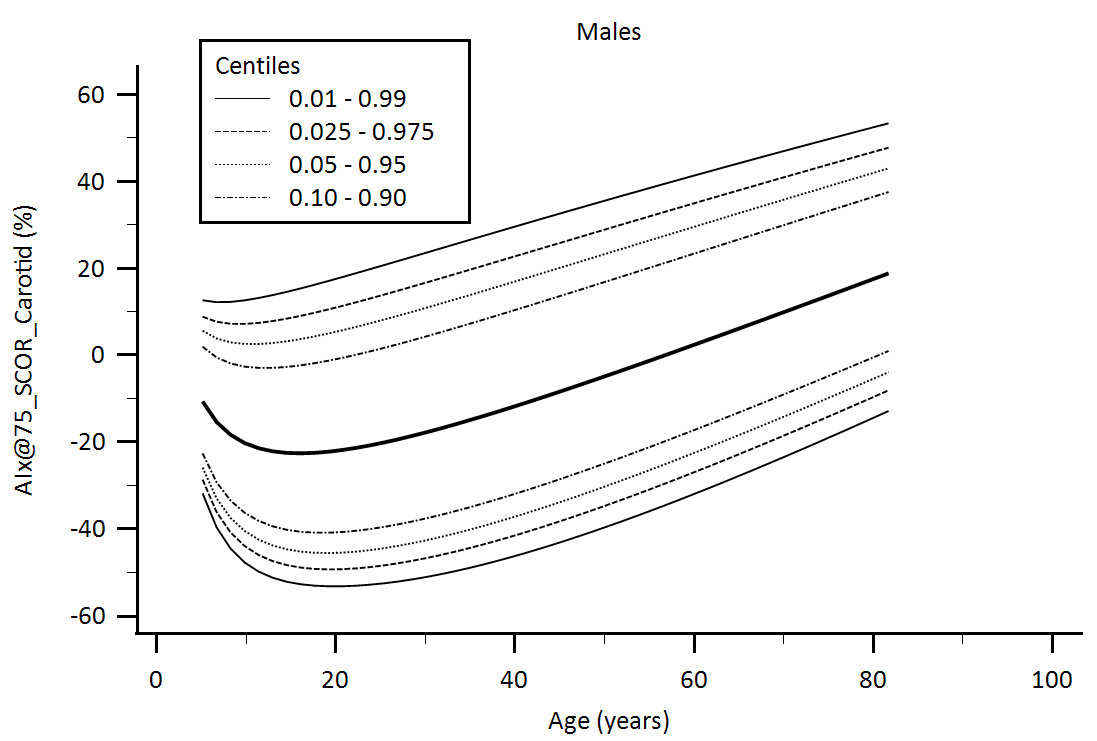


Supplementary Figure 17. Central aortic waveform-derived parameters obtained using carotid artery applanation tonometry (SphygmoCor device, SCOR): Augmentation Index adjusted for heart rate equal 75 beats/minute (AIx@75) age-related percentiles.


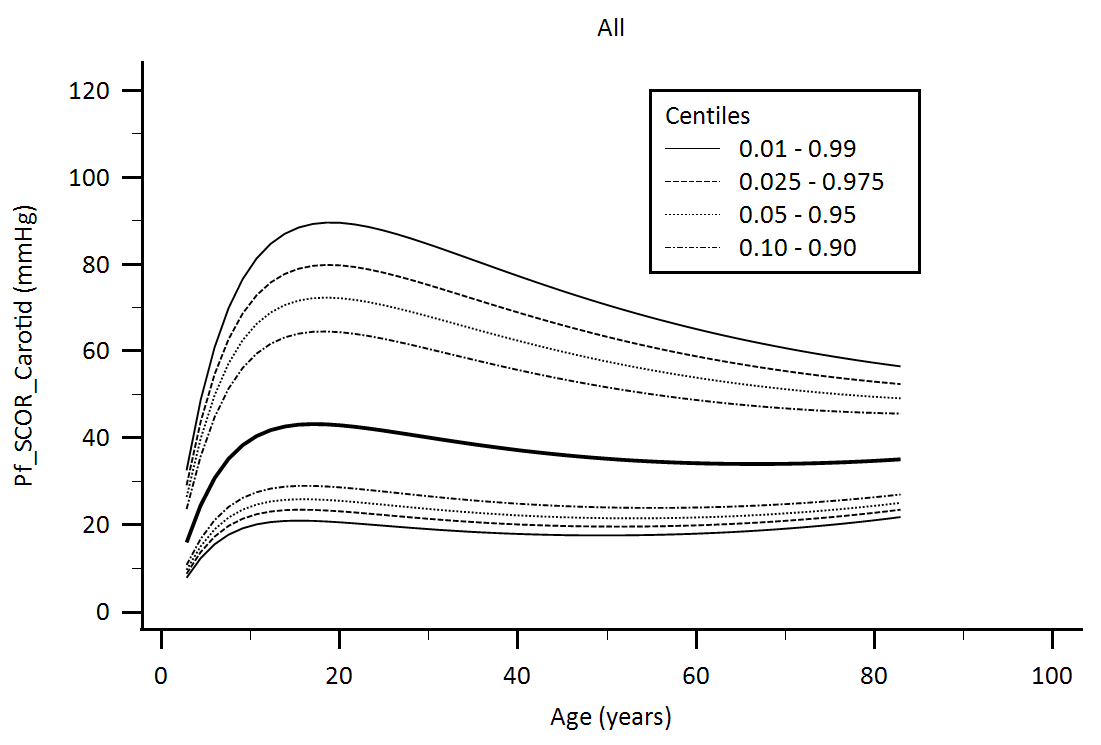

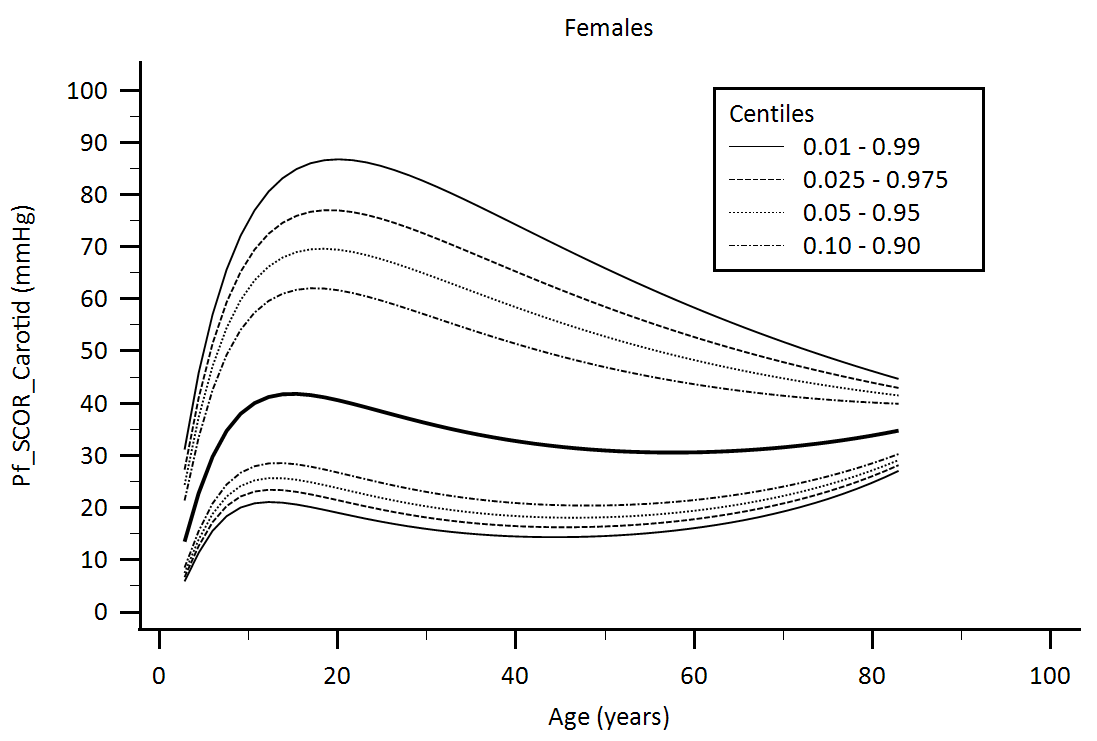


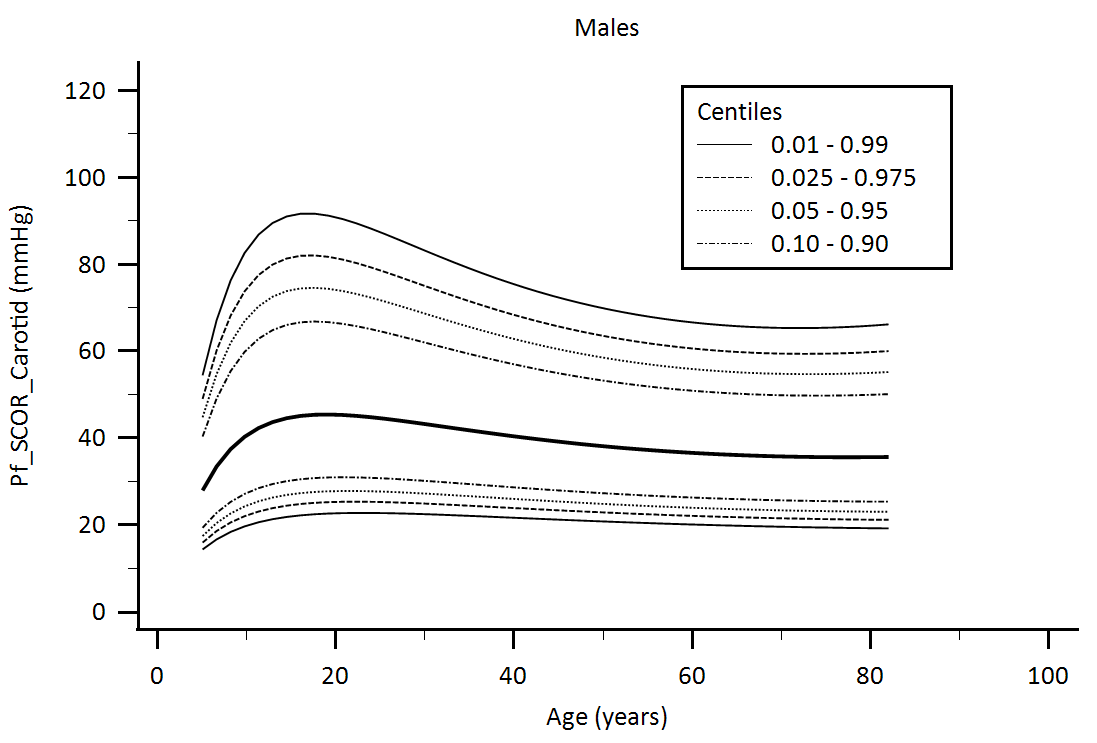


Supplementary Figure 18. Central aortic waveform-derived parameters obtained using carotid artery applanation tonometry (SphygmoCor device, SCOR): Forward pressure (Pf) age-related percentiles.


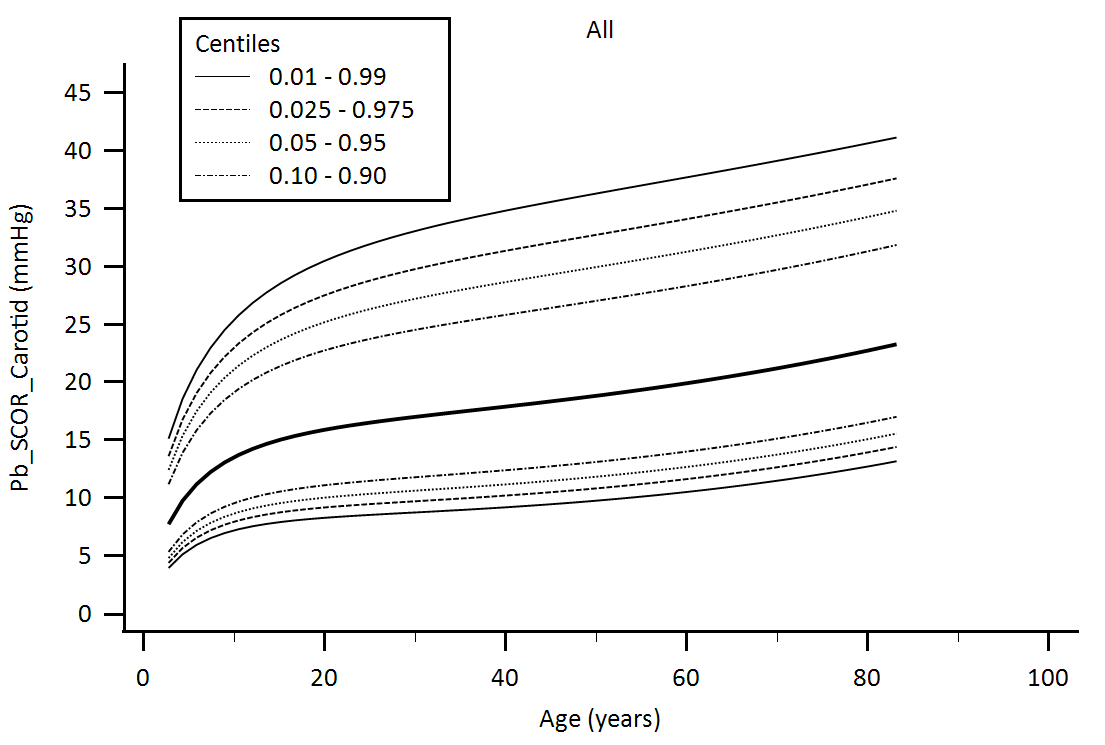

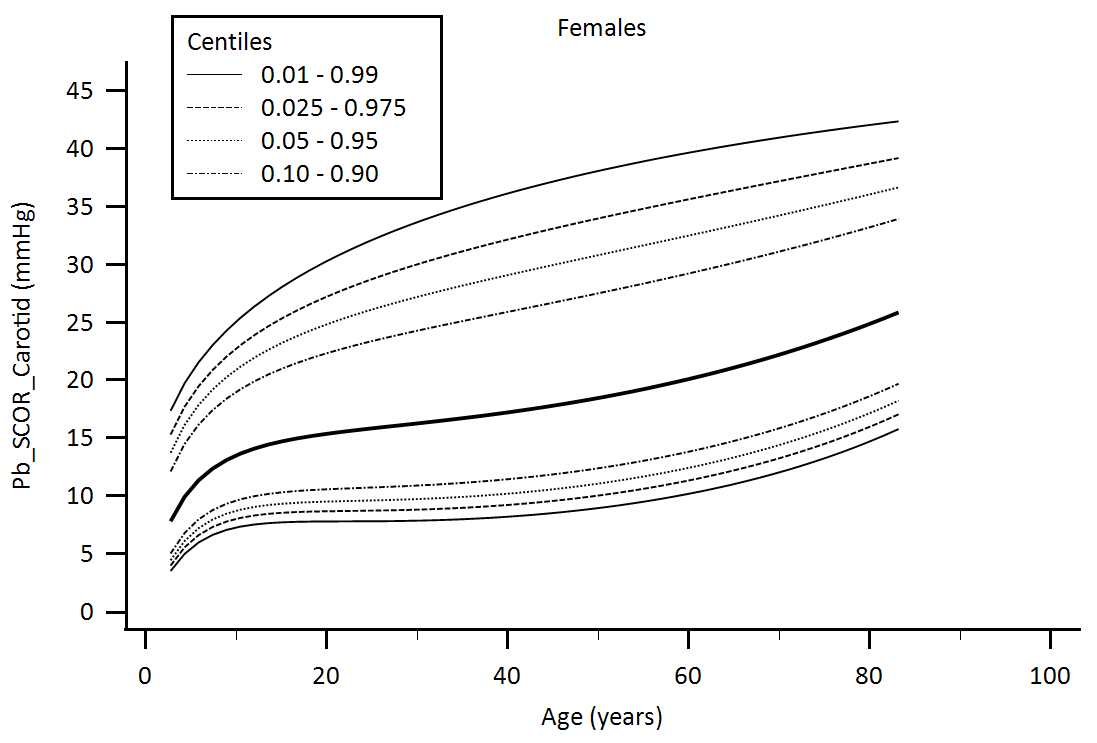


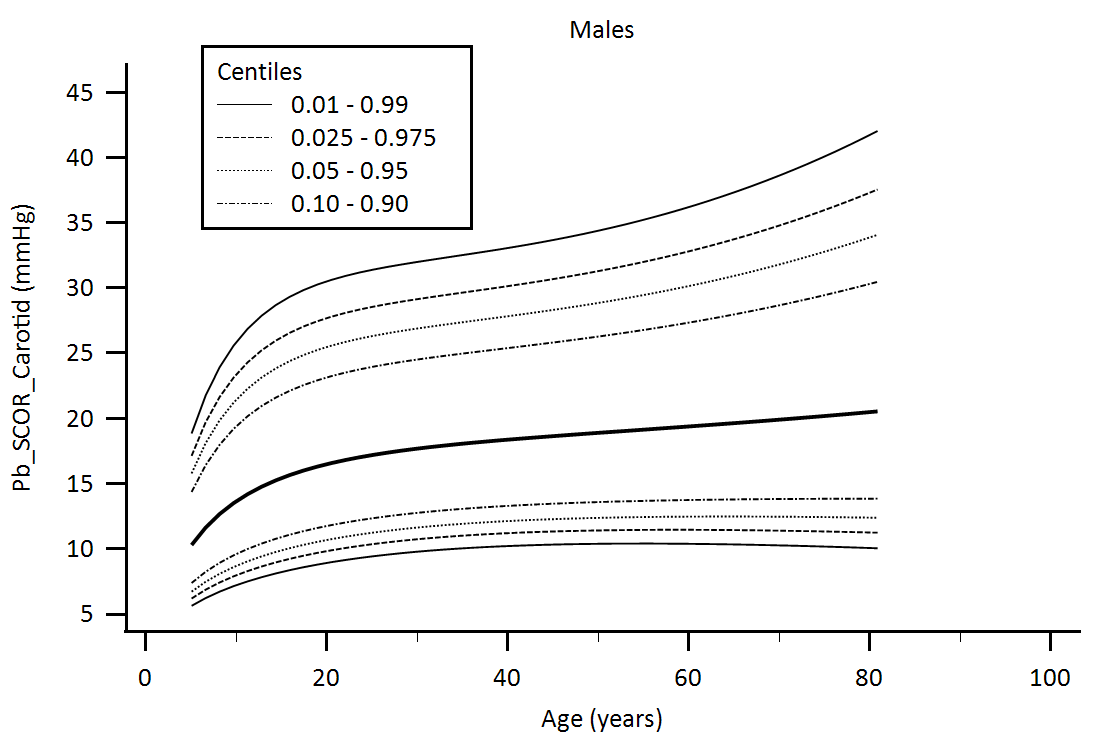


Supplementary Figure 19. Central aortic waveform-derived parameters obtained using carotid artery applanation tonometry (SphygmoCor device, SCOR): Backward pressure (Pb) age-related percentiles.


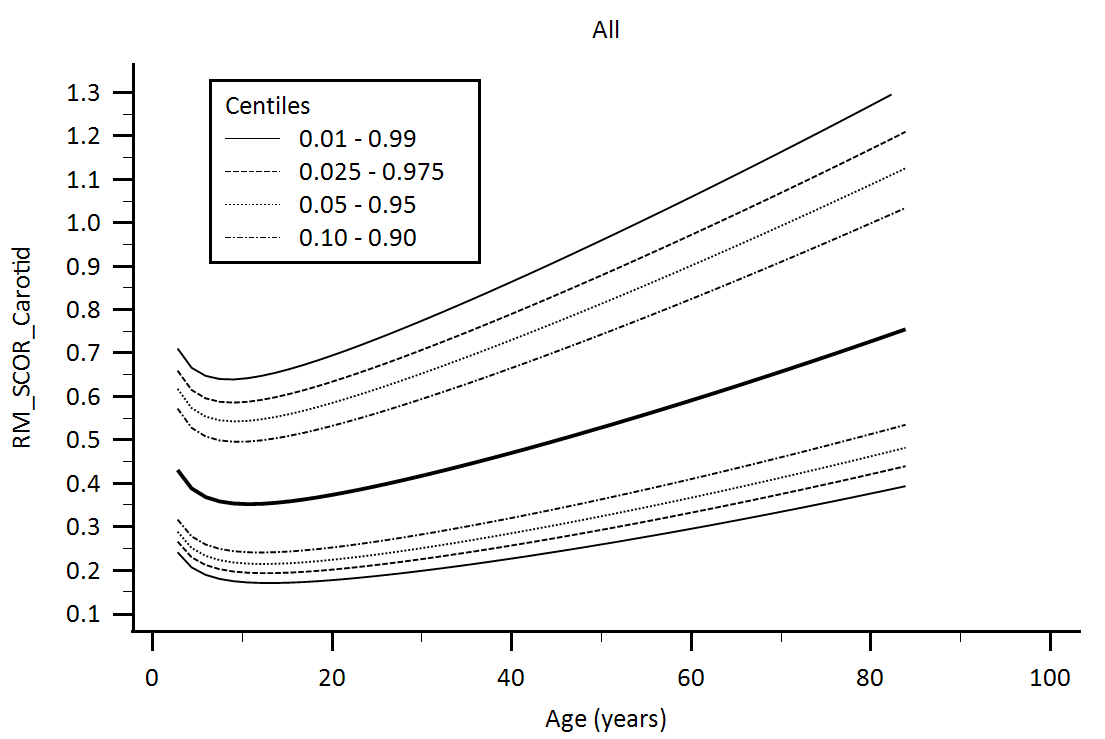


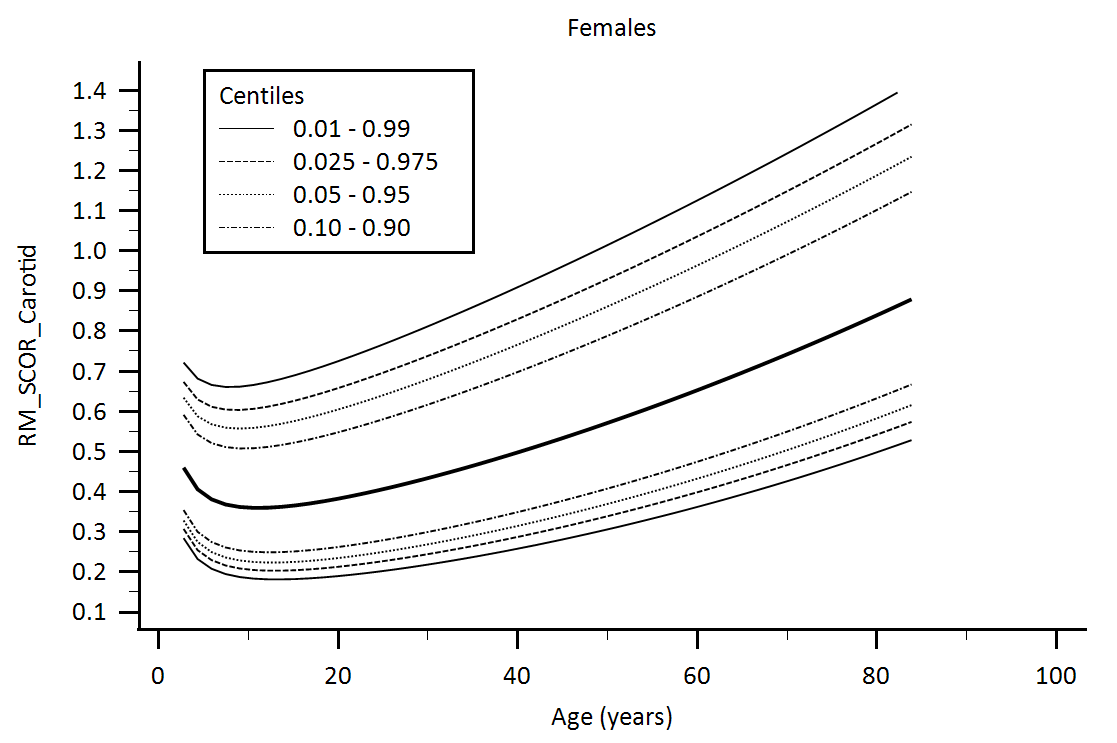


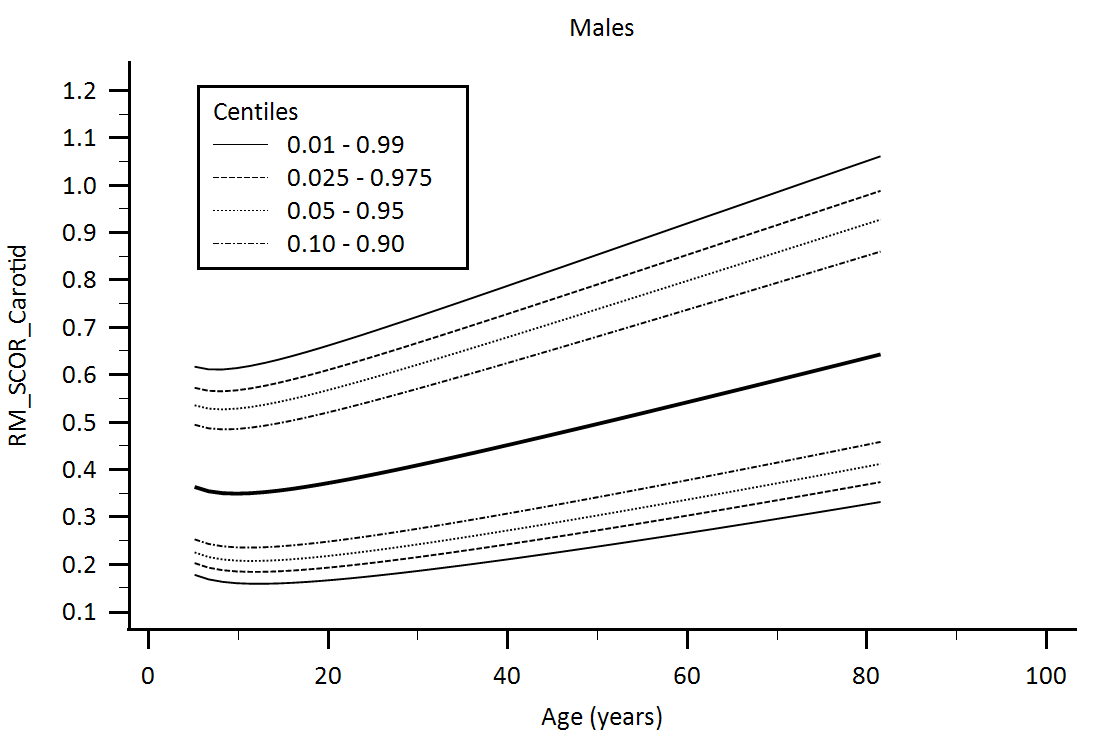


Supplementary Figure 20. Central aortic waveform-derived parameters obtained using carotid artery applanation tonometry (SphygmoCor device, SCOR): Reflection Magnitude (RM) age-related percentiles.


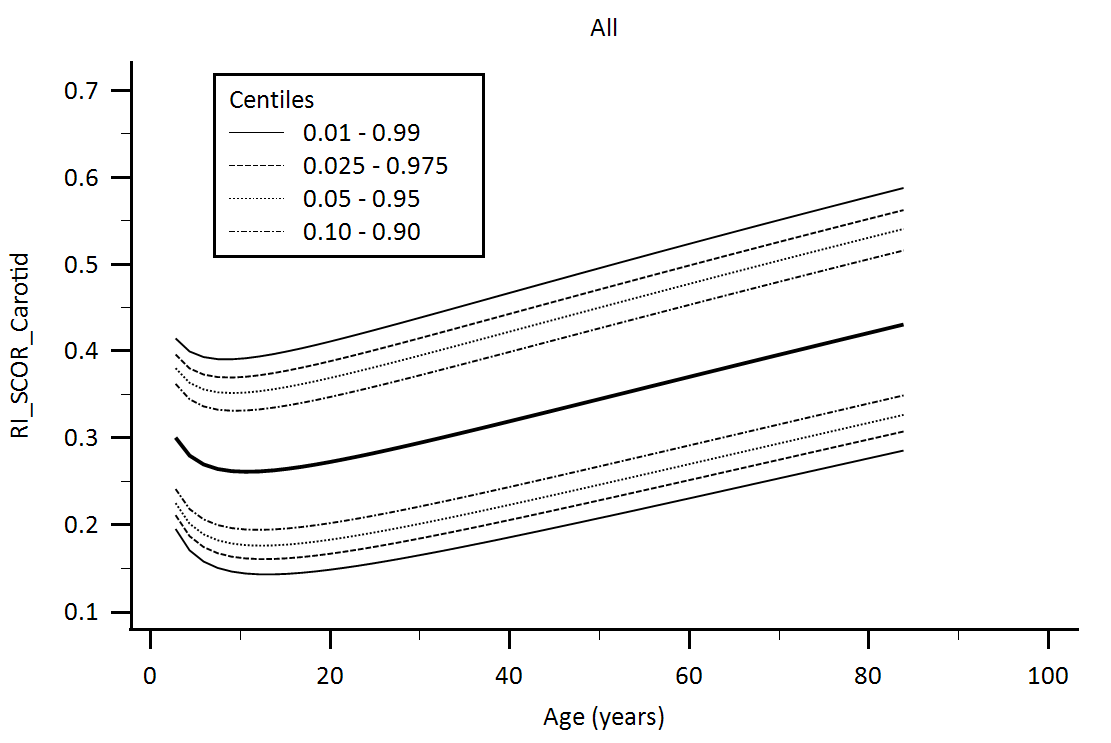


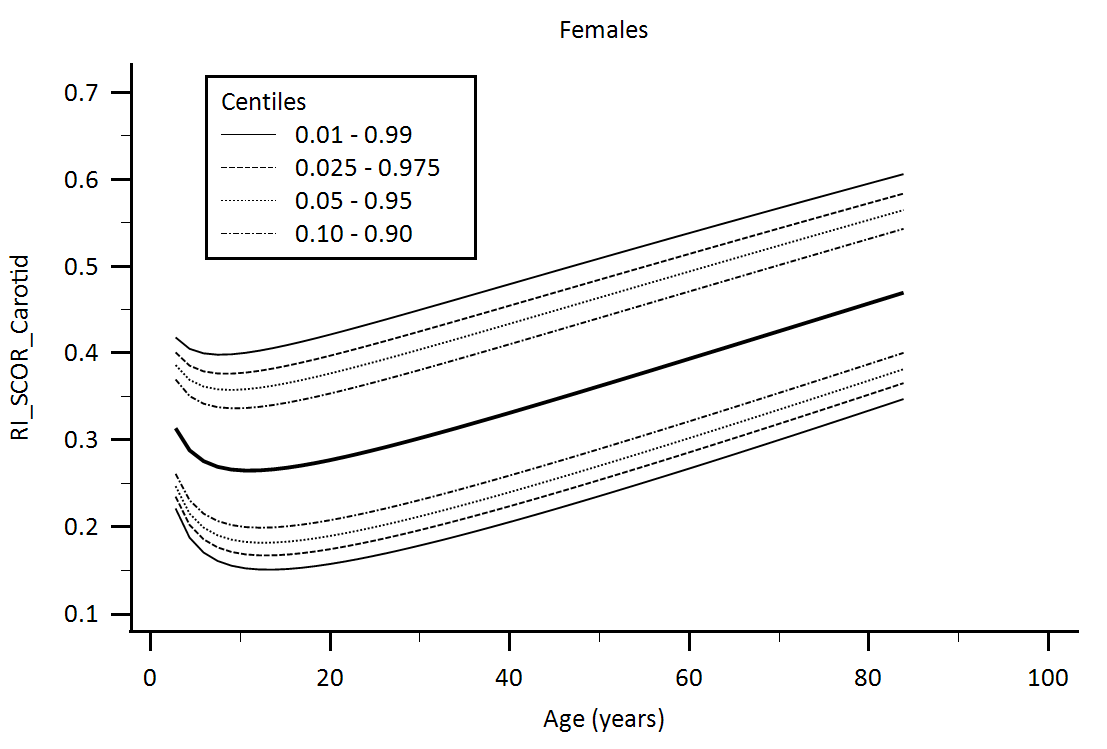


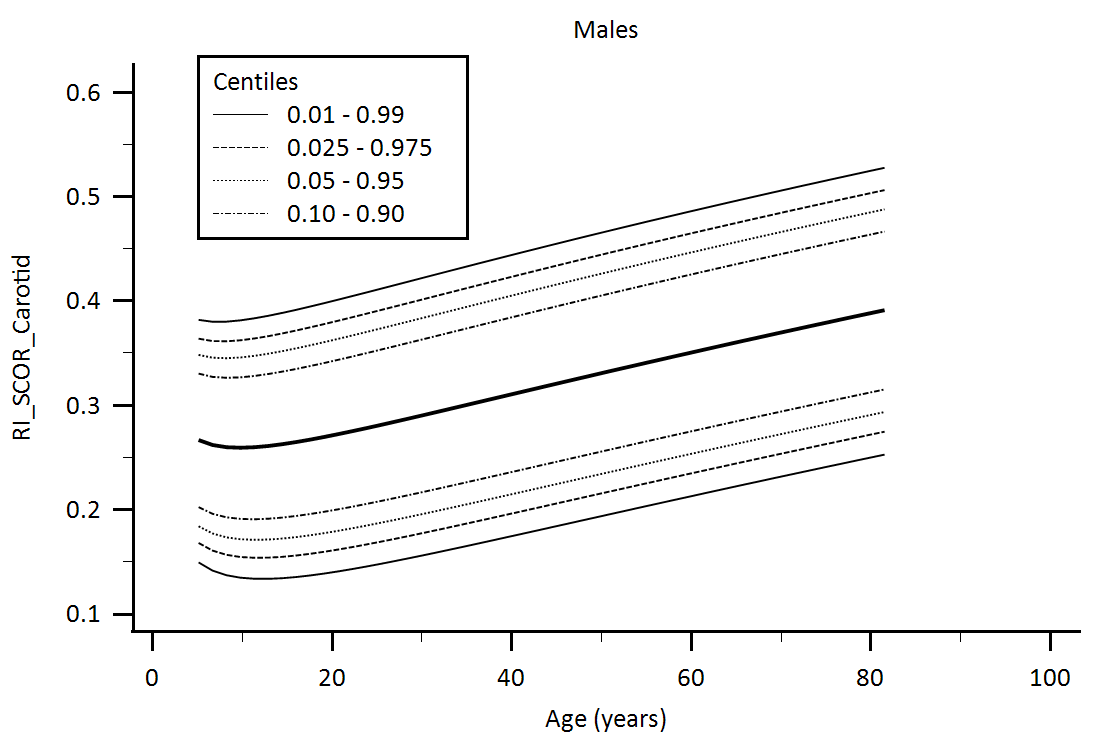


Supplementary Figure 21. Central aortic waveform-derived parameters obtained using carotid artery applanation tonometry (SphygmoCor device, SCOR): Reflection Index (RI) age-related percentiles.
