# Supplementary material for: Central Pressure Waveform-Derived Indexes Obtained From Carotid and Radial Tonometry and Brachial Oscillometry in Healthy Subjects (2–84 Y): Age-, Height-, and Sex-Related Profiles and Analysis of Indexes Agreement
Source: Front Physiol. 2022 Jan 20;12:774390. doi: 10.3389/fphys.2021.774390 (PMC8811372; doi:10.3389/fphys.2021.774390)
Supplement: Supplementary file 2 [file Data_Sheet_2.docx]

**Supplementary Figures: Central aortic waveform-derived parameters obtained using Mobil-O-Graph device: Body height-related percentiles**


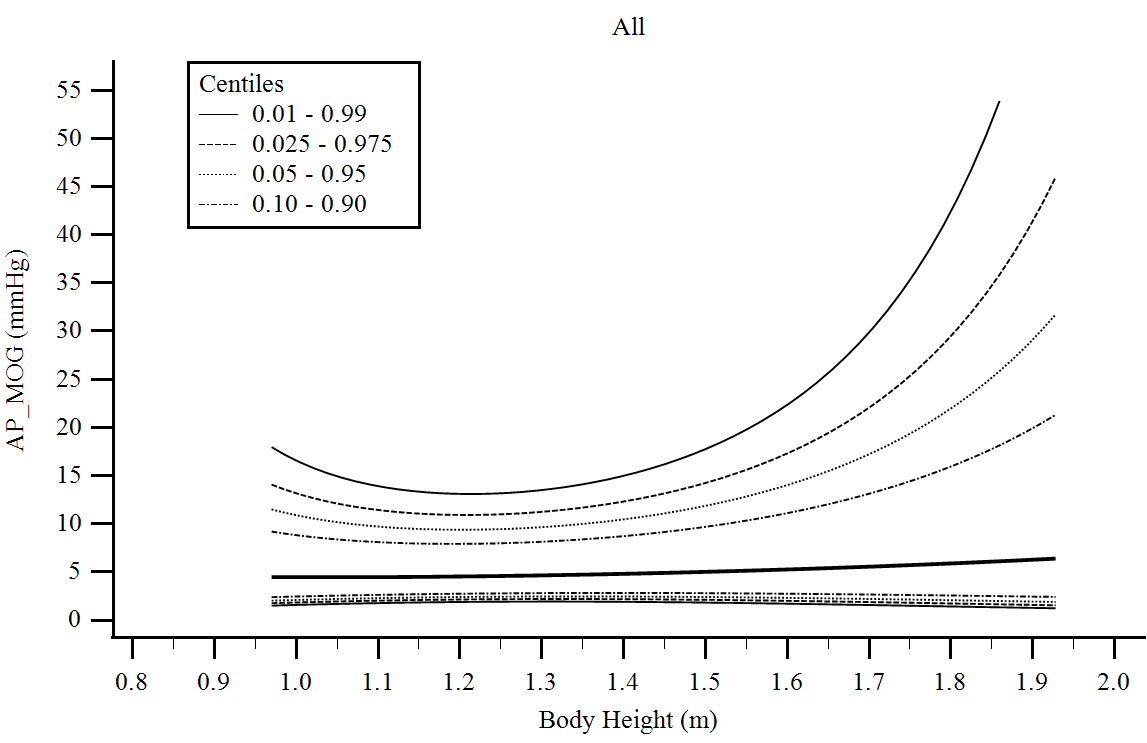


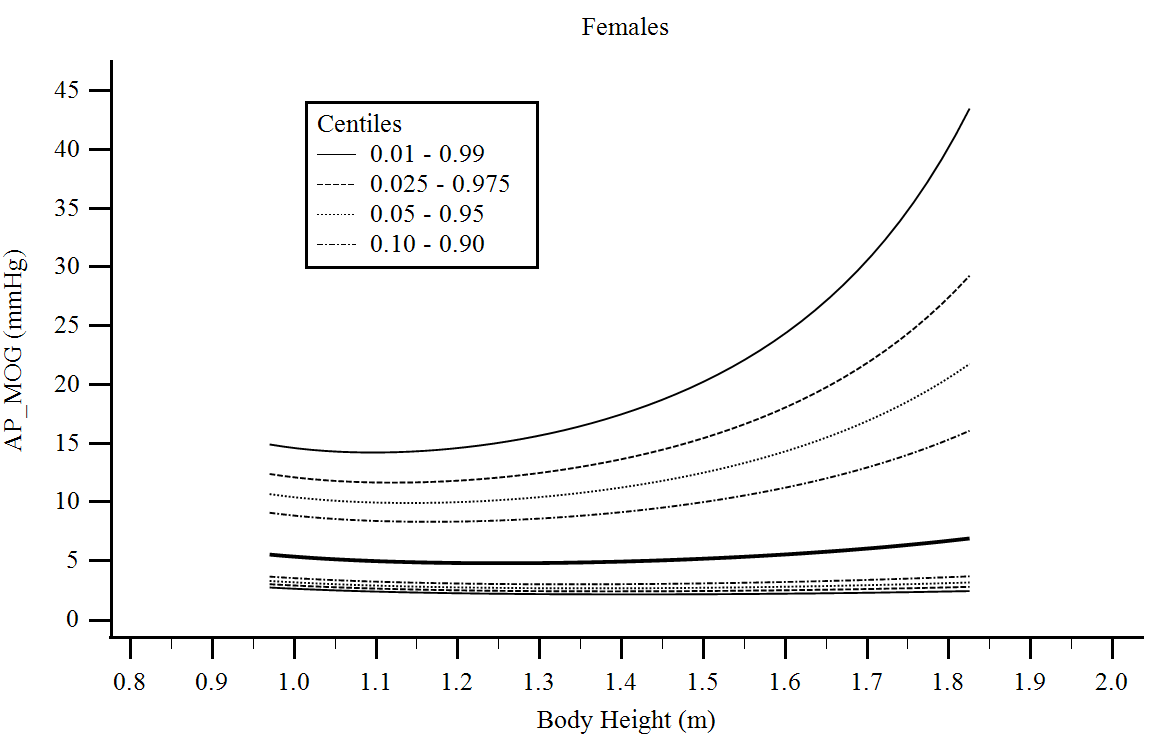

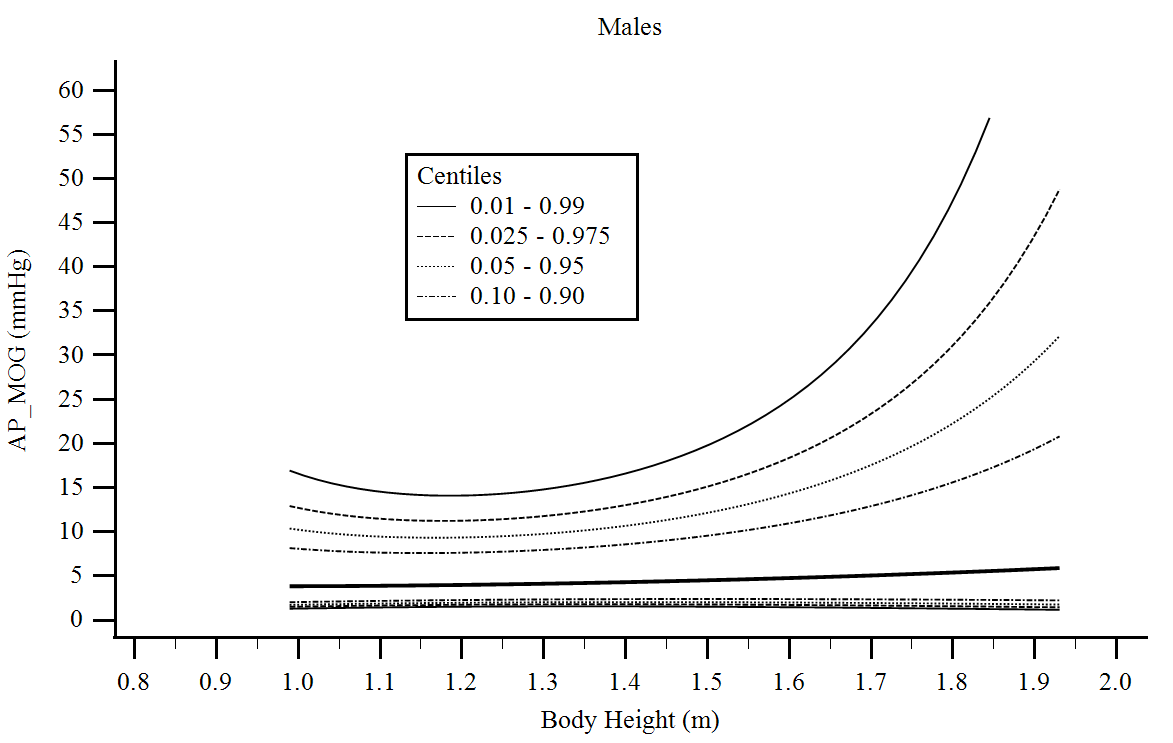


Supplementary Figure 22. Central aortic waveform-derived parameters obtained using Mobil-O-Graph device (MOG): Augmentation Pressure (AP) body height-related percentiles.


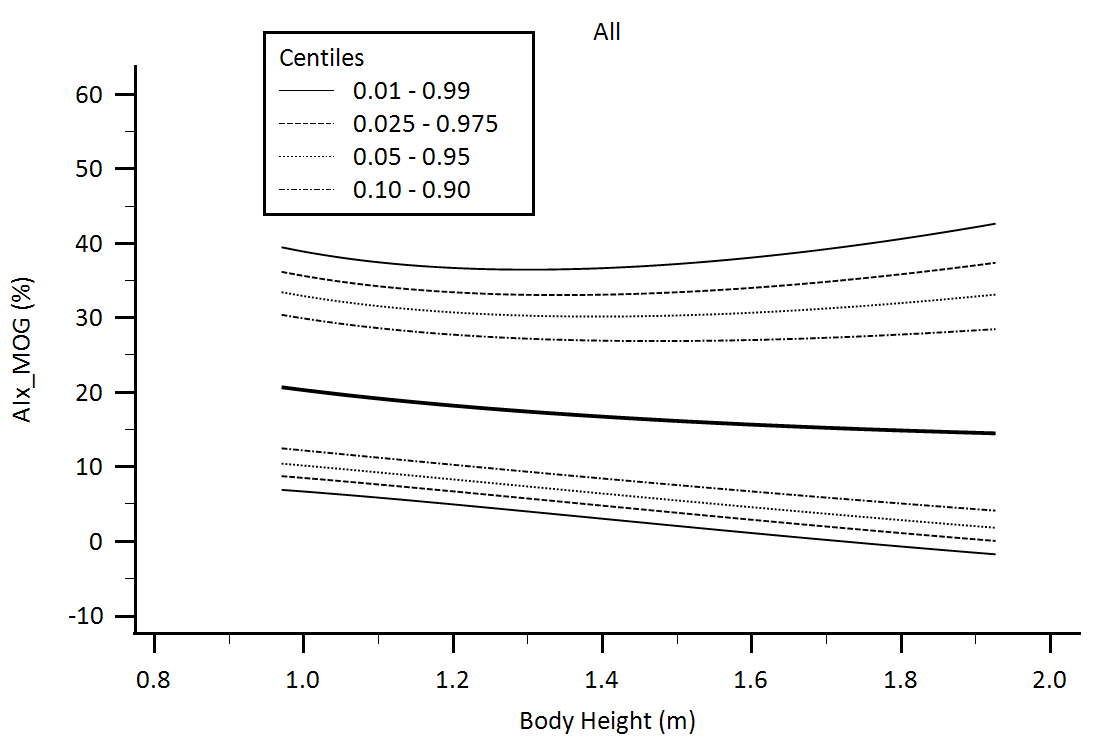

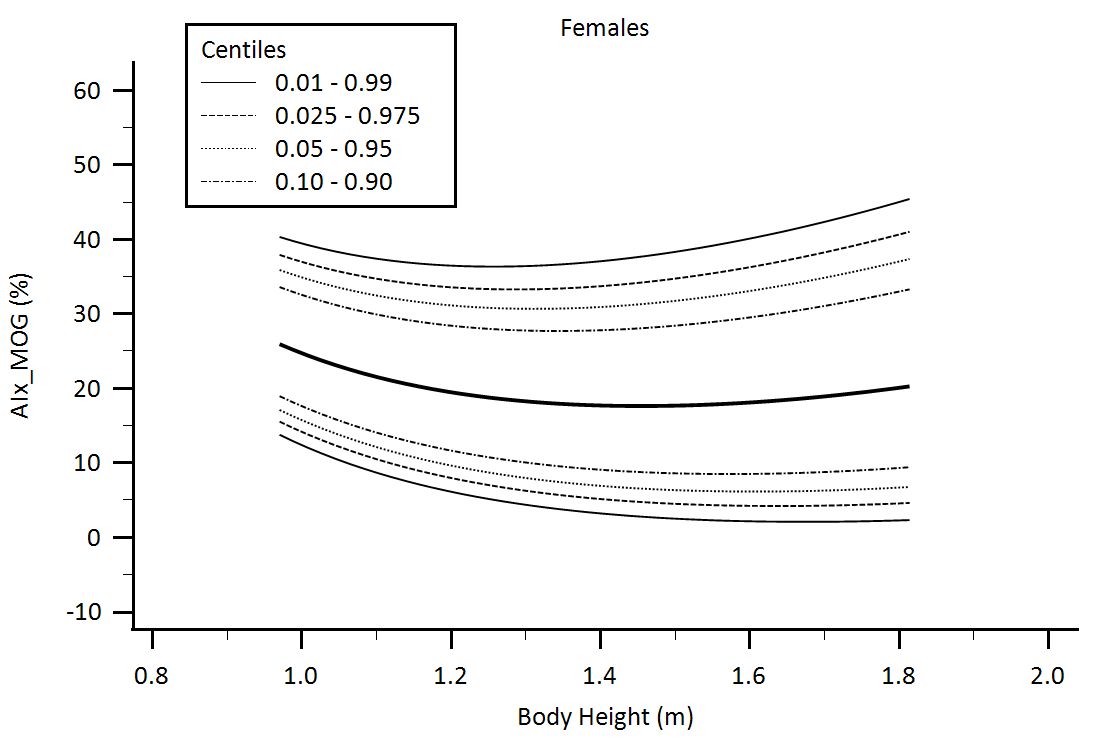

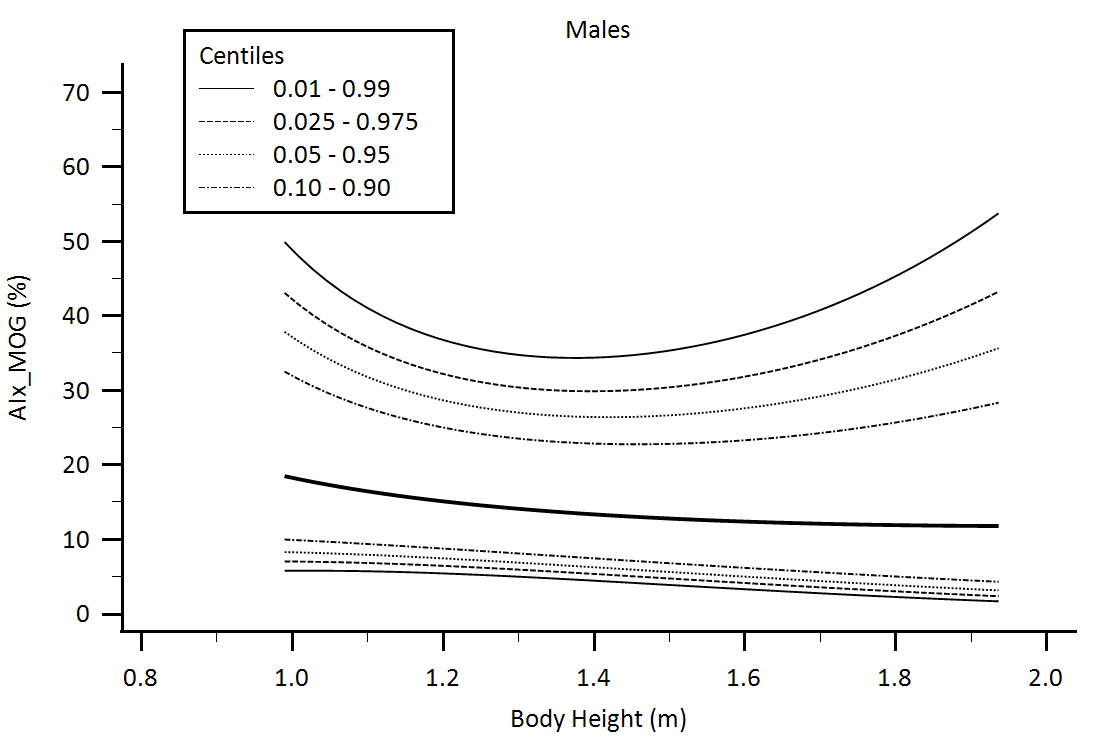


Supplementary Figure 23. Central aortic waveform-derived parameters obtained using Mobil-O-Graph device (MOG): Augmentation Index (AIx) body height-related percentiles.


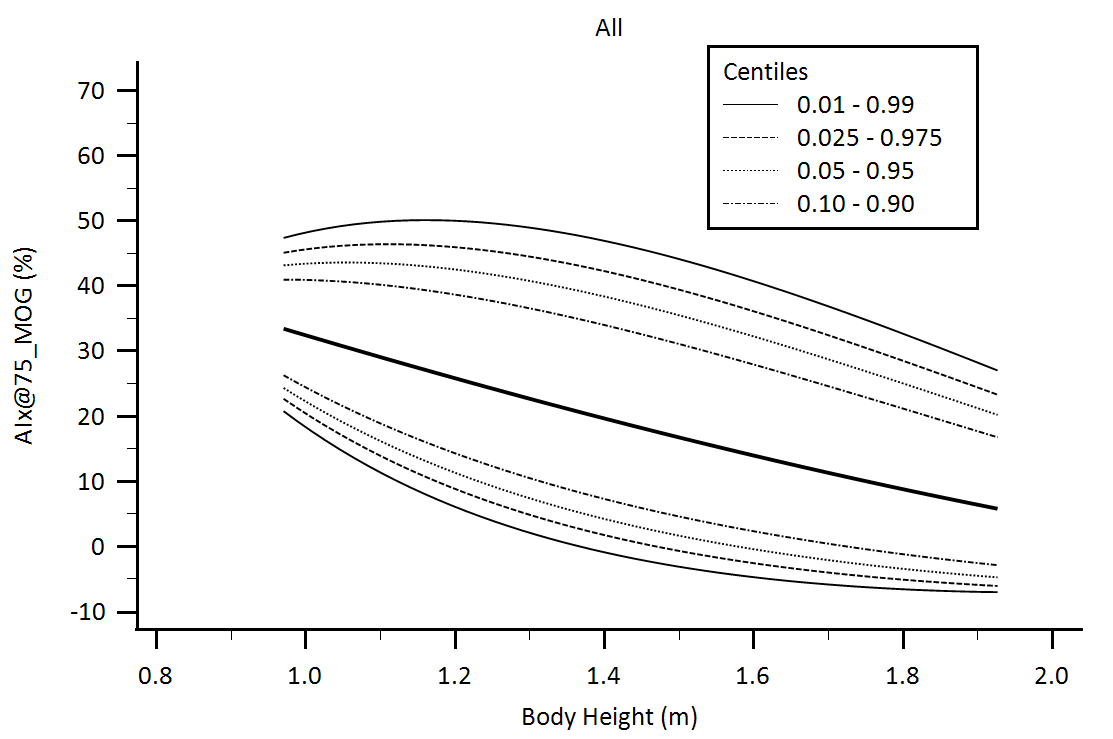

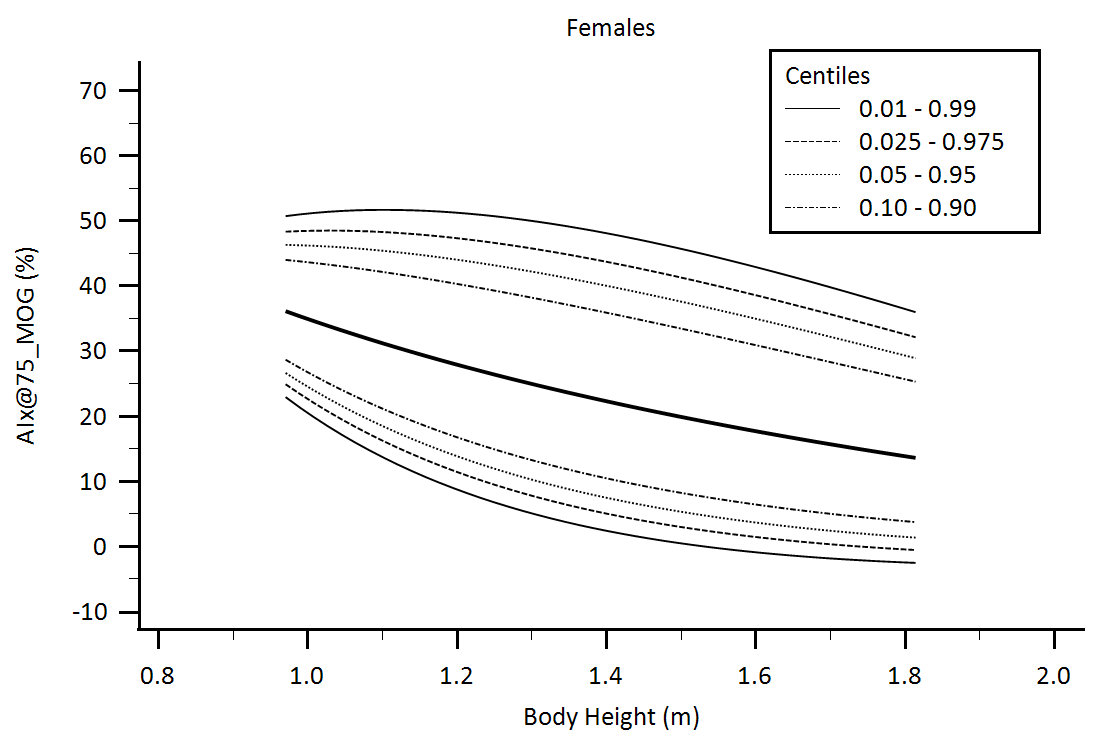

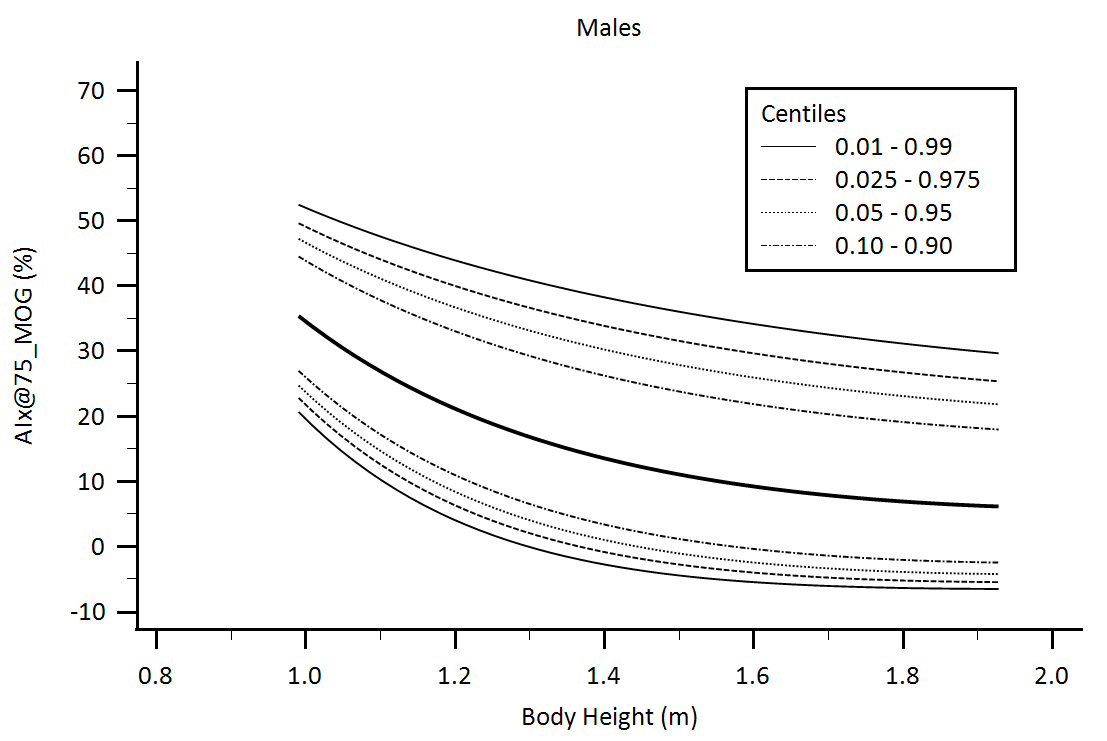


Supplementary Figure 24. Central aortic waveform-derived parameters obtained using Mobil-O-Graph device (MOG): Augmentation Index adjusted for heart rate equal 75 beats/minute (AIx@75) body height-related percentiles.


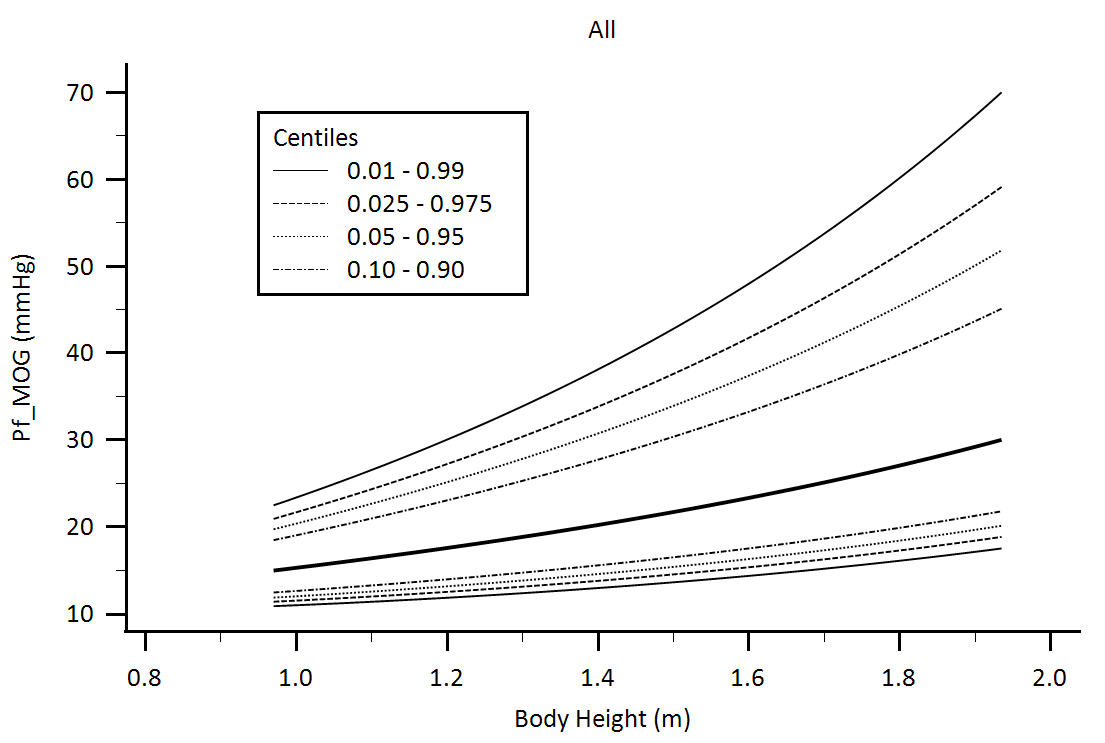

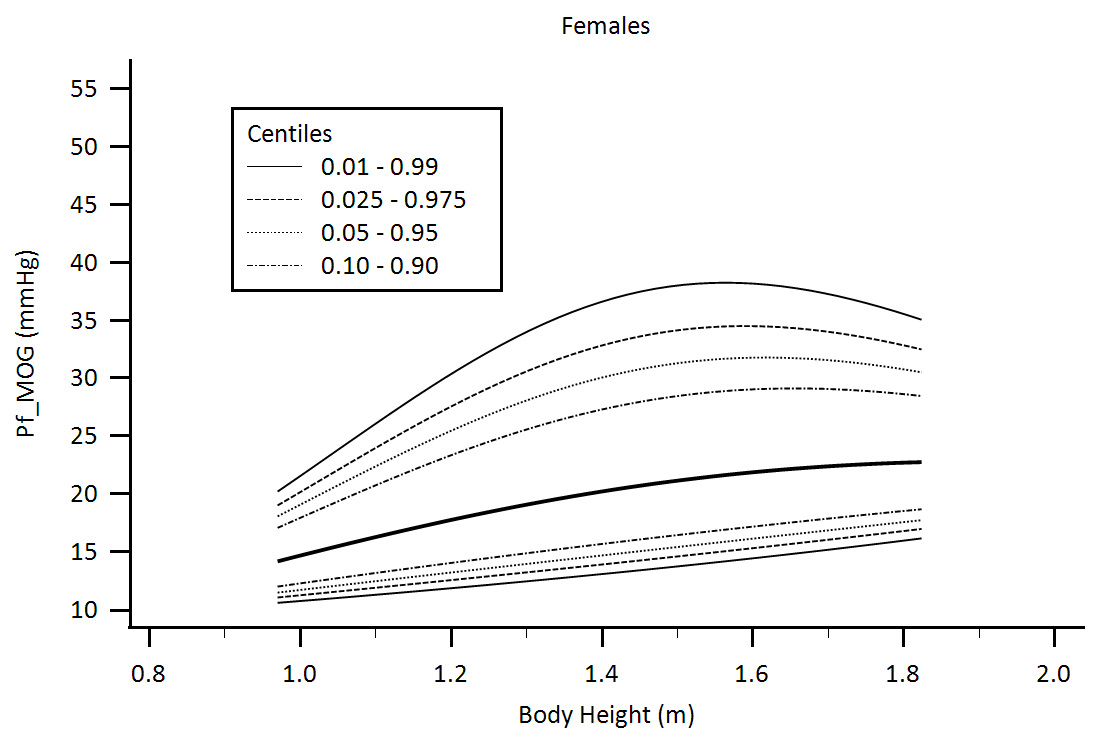

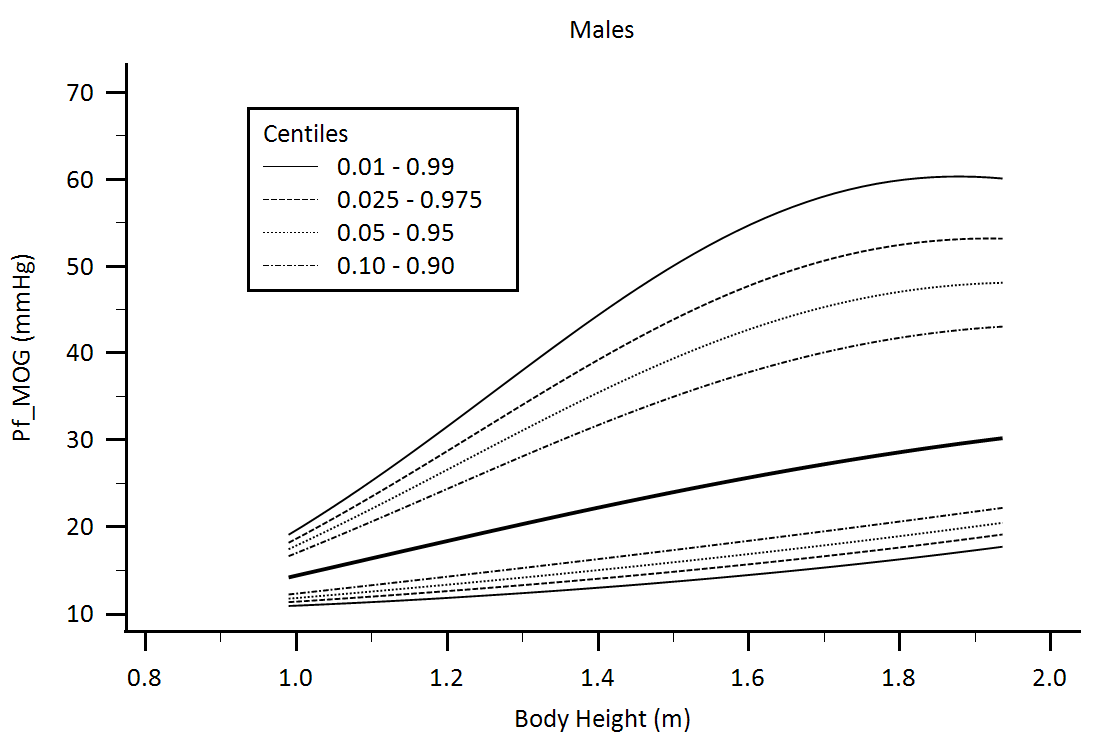


Supplementary Figure 25. Central aortic waveform-derived parameters obtained using Mobil-O-Graph device (MOG): Forward pressure (Pf) body height-related percentiles.


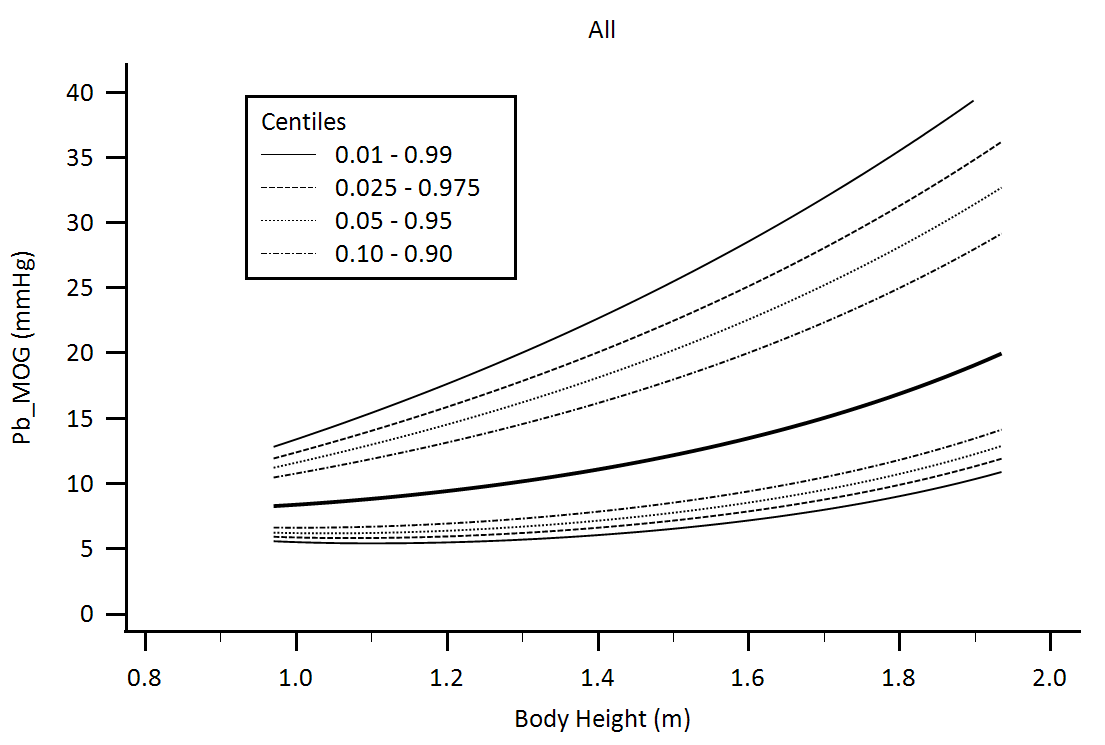


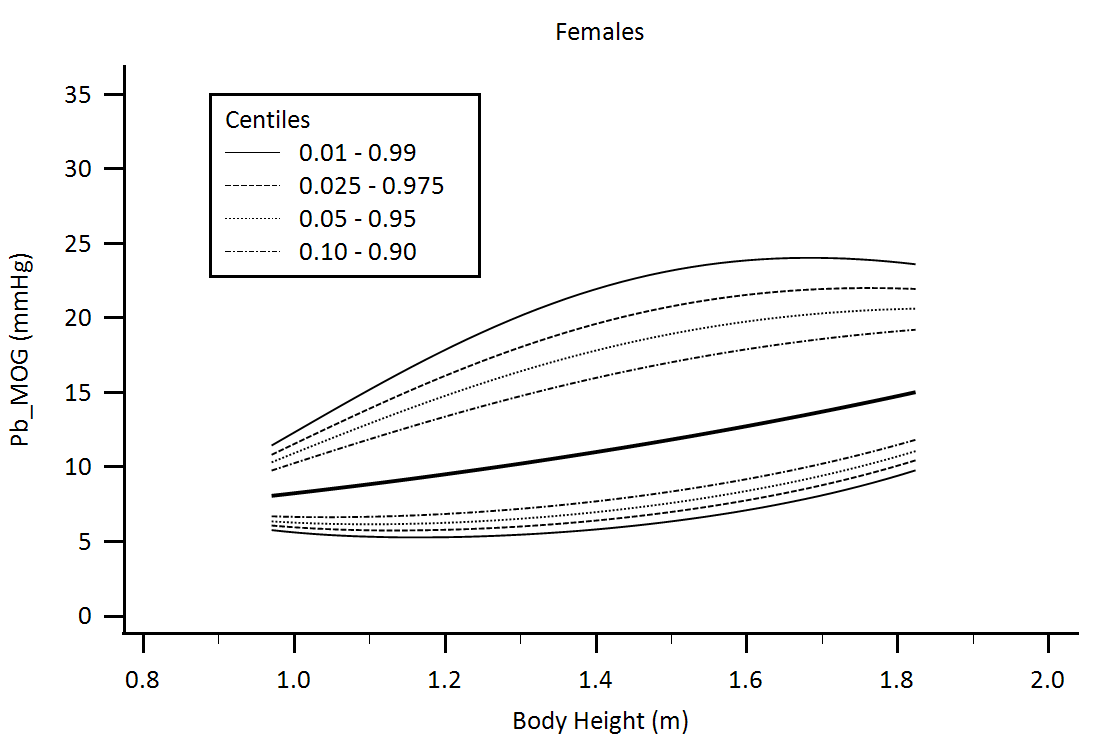

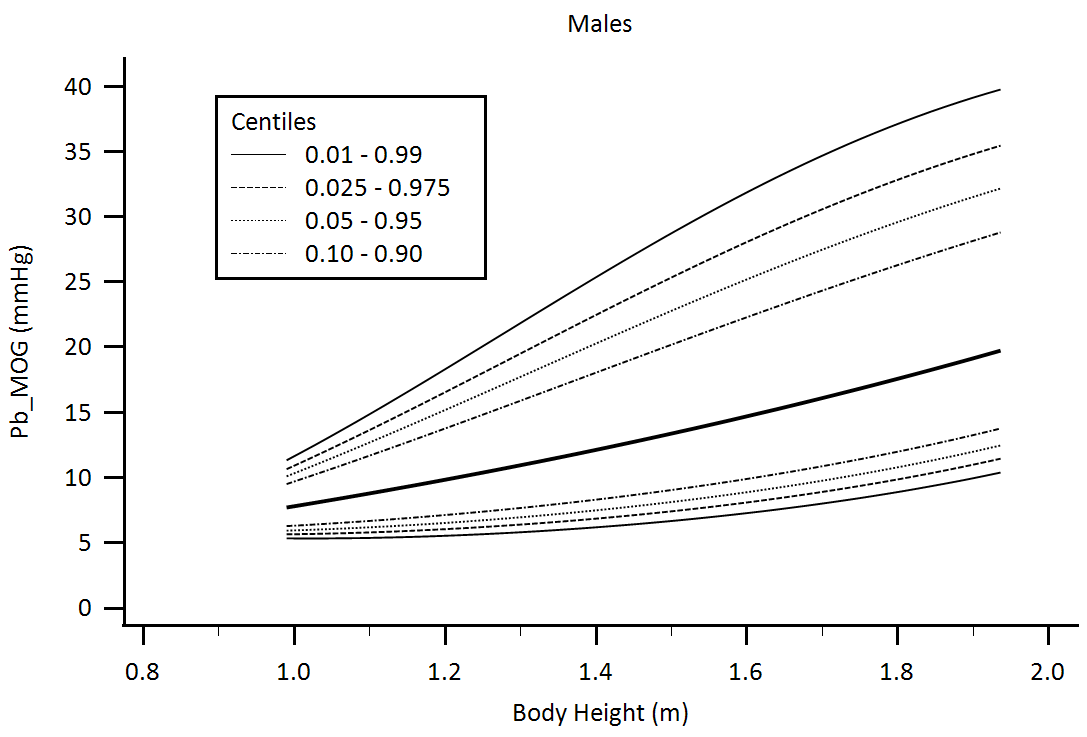


Supplementary Figure 26. Central aortic waveform-derived parameters obtained using Mobil-O-Graph device (MOG): Backward pressure (Pb) body height-related percentiles.


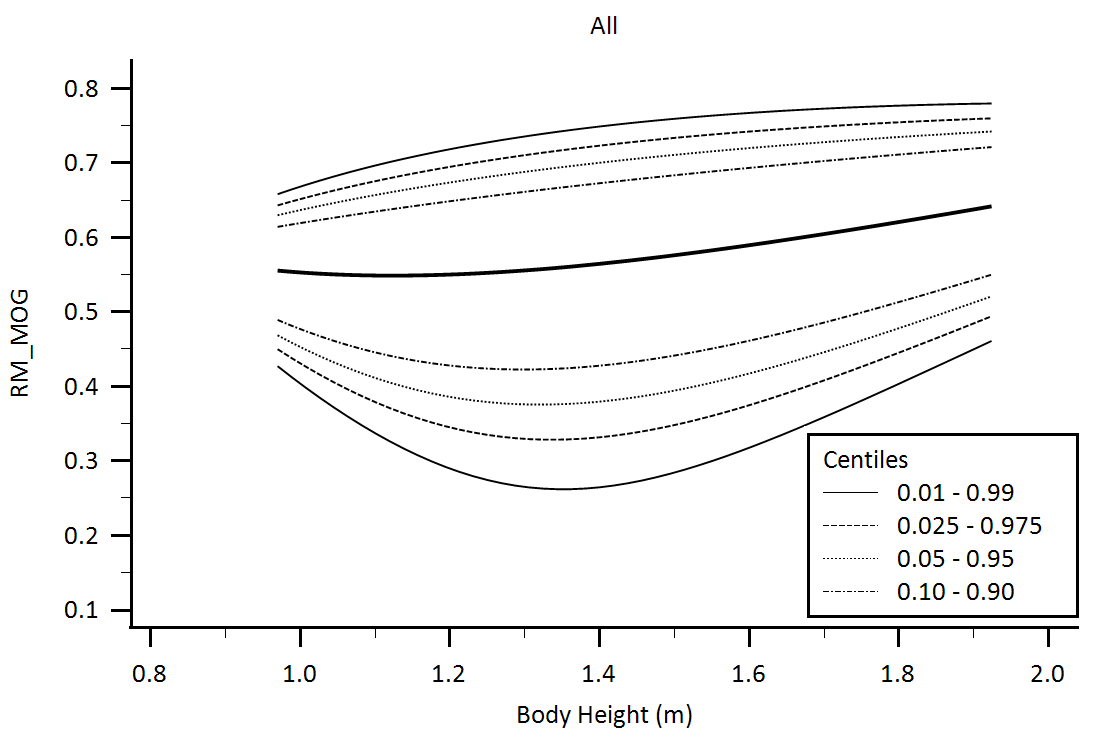


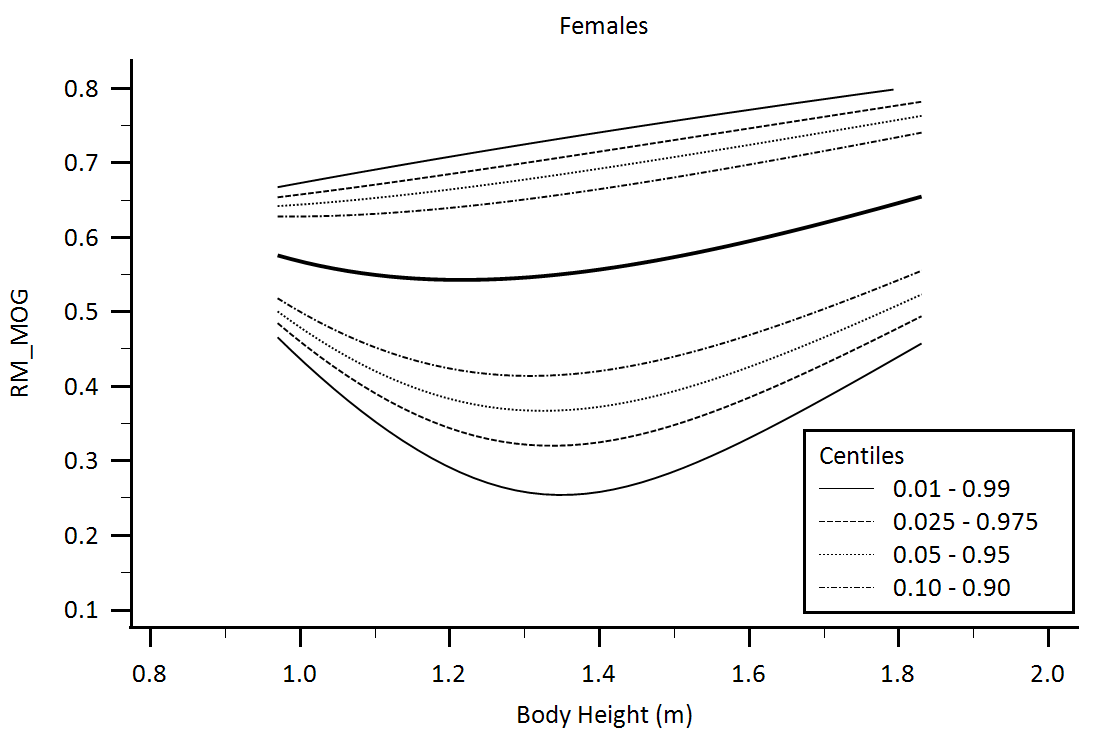

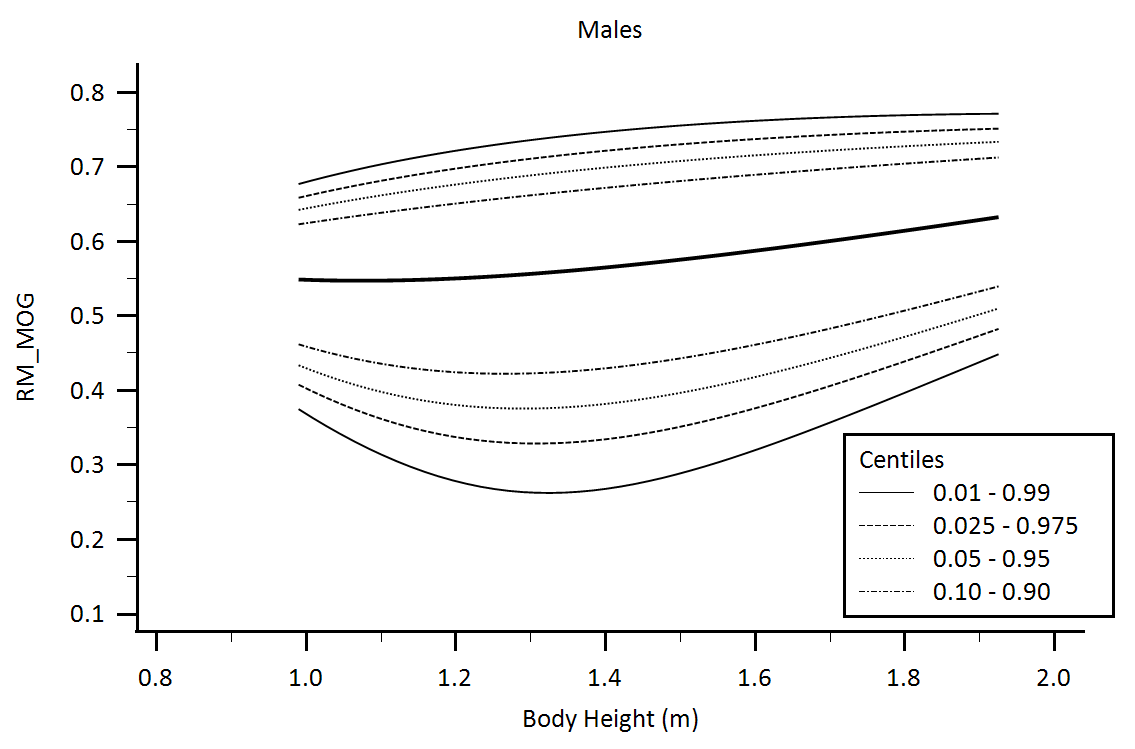


Supplementary Figure 27. Central aortic waveform-derived parameters obtained using Mobil-O-Graph device (MOG): Reflection Magnitude (RM) body height-related percentiles.


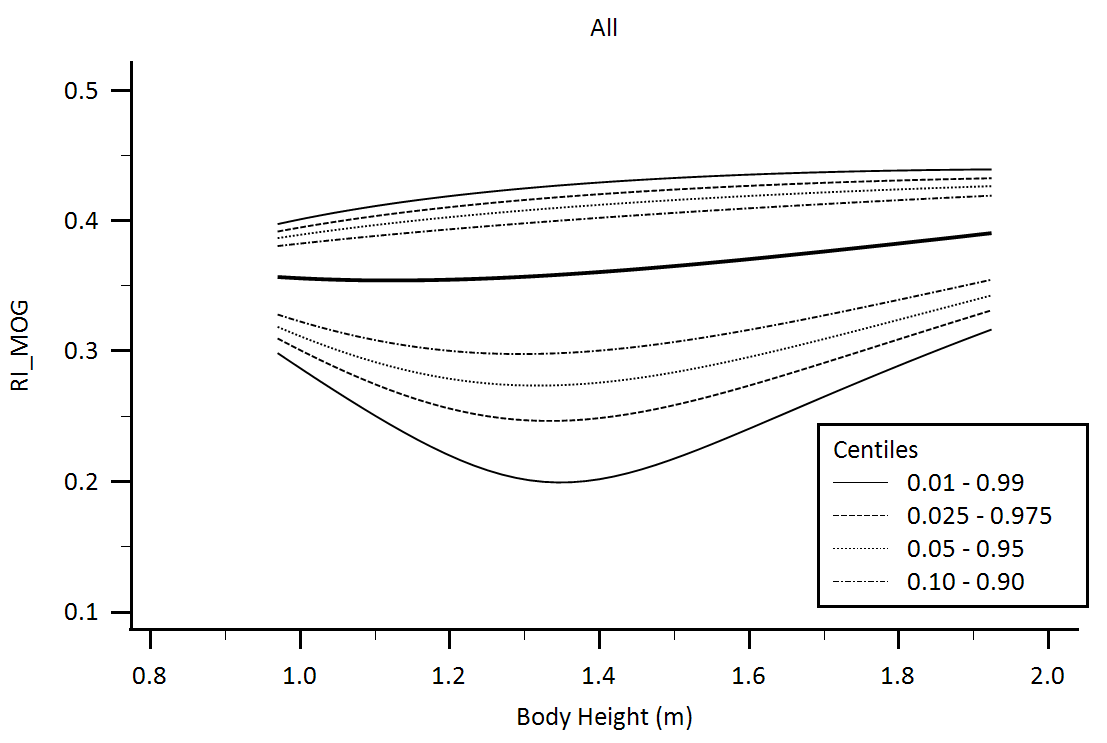


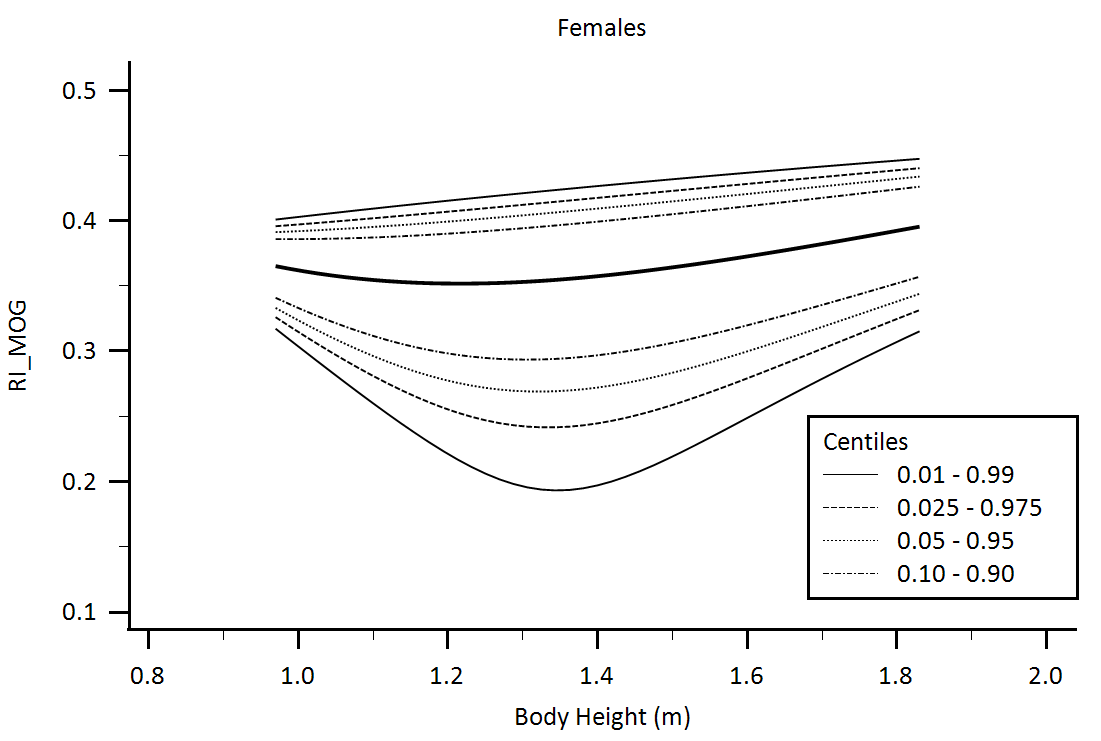

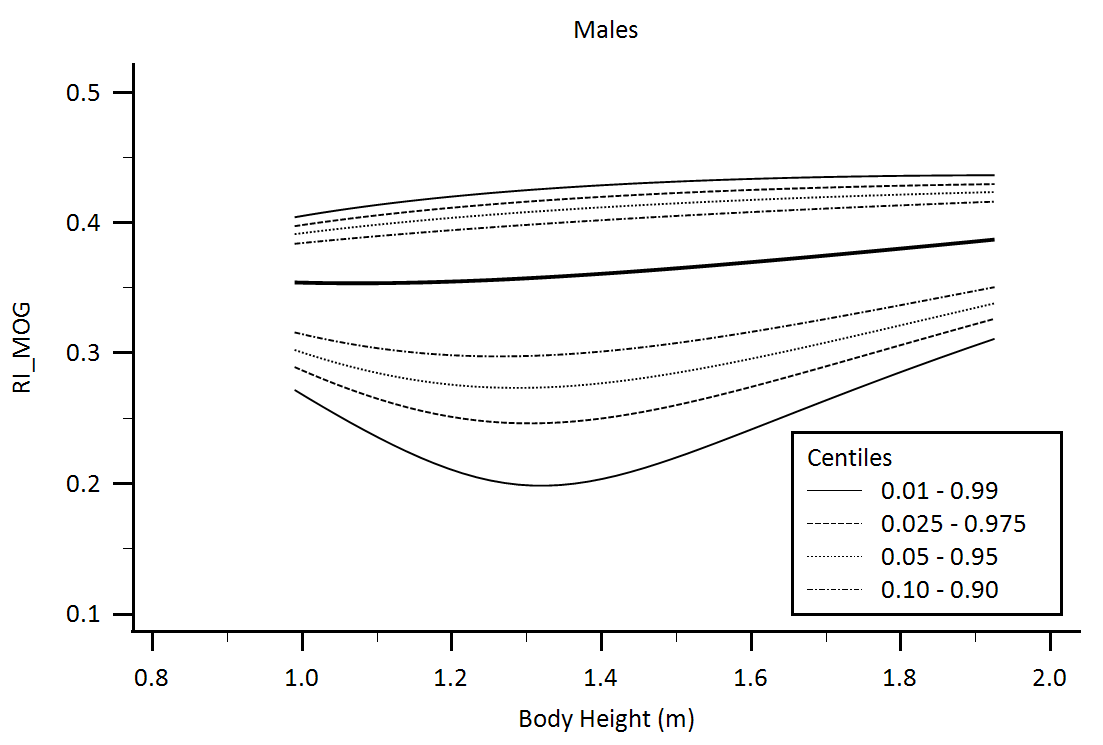


Supplementary Figure 28. Central aortic waveform-derived parameters obtained using Mobil-O-Graph device (MOG): Reflection Index (RI) body height-related percentiles.


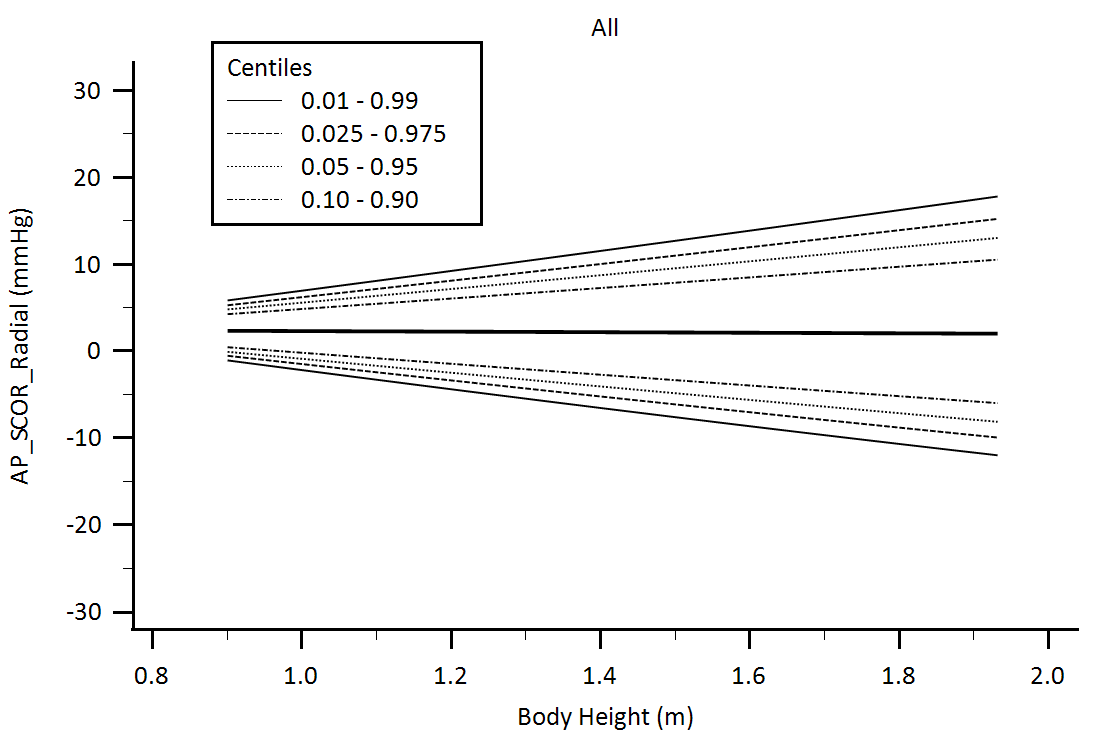

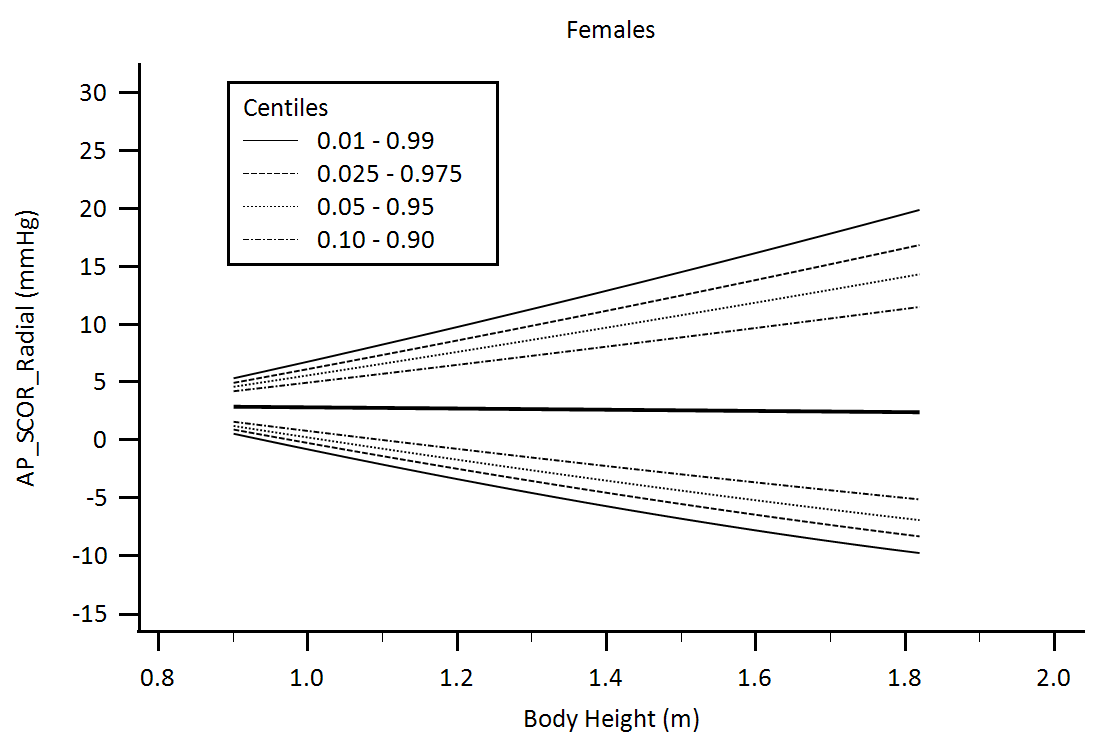

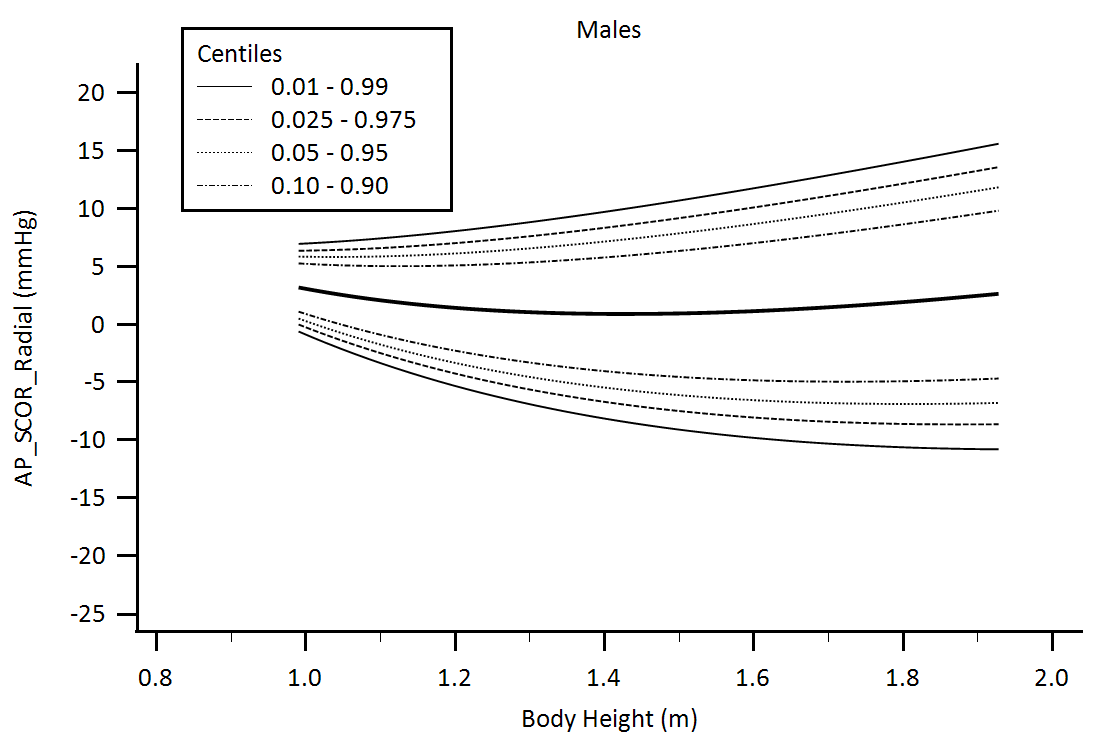


Supplementary Figure 29. Central aortic waveform-derived parameters obtained using radial artery applanation tonometry (SphygmoCor device, SCOR): Augmentation pressure (AP) body height-related percentiles.


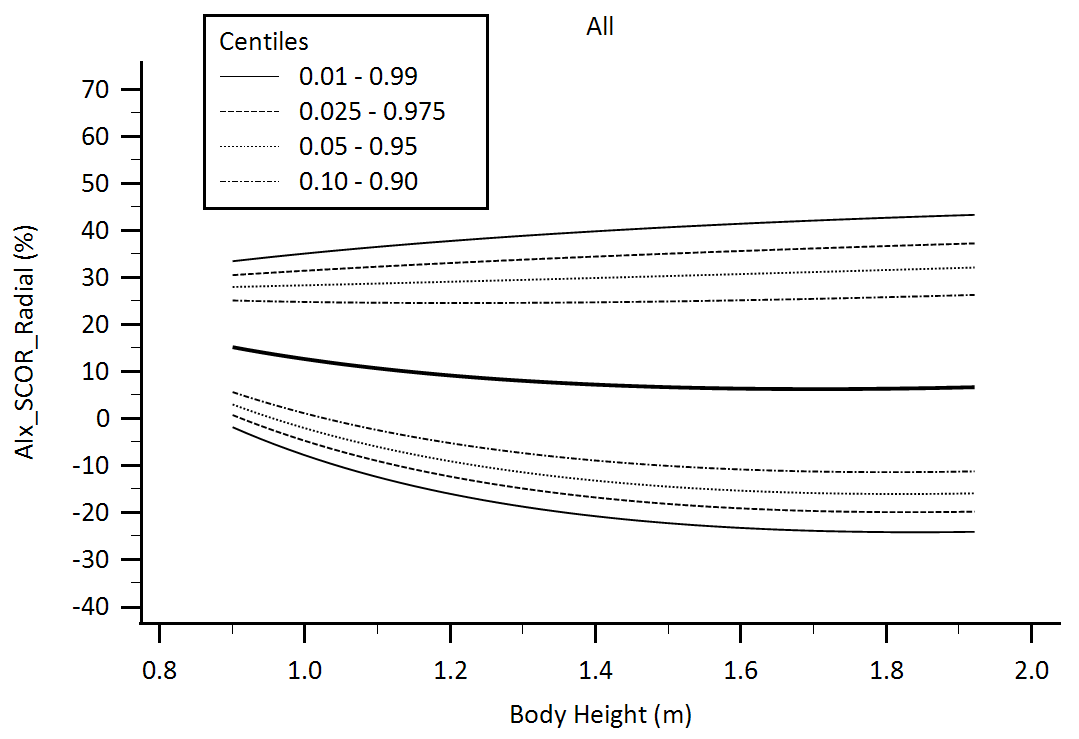

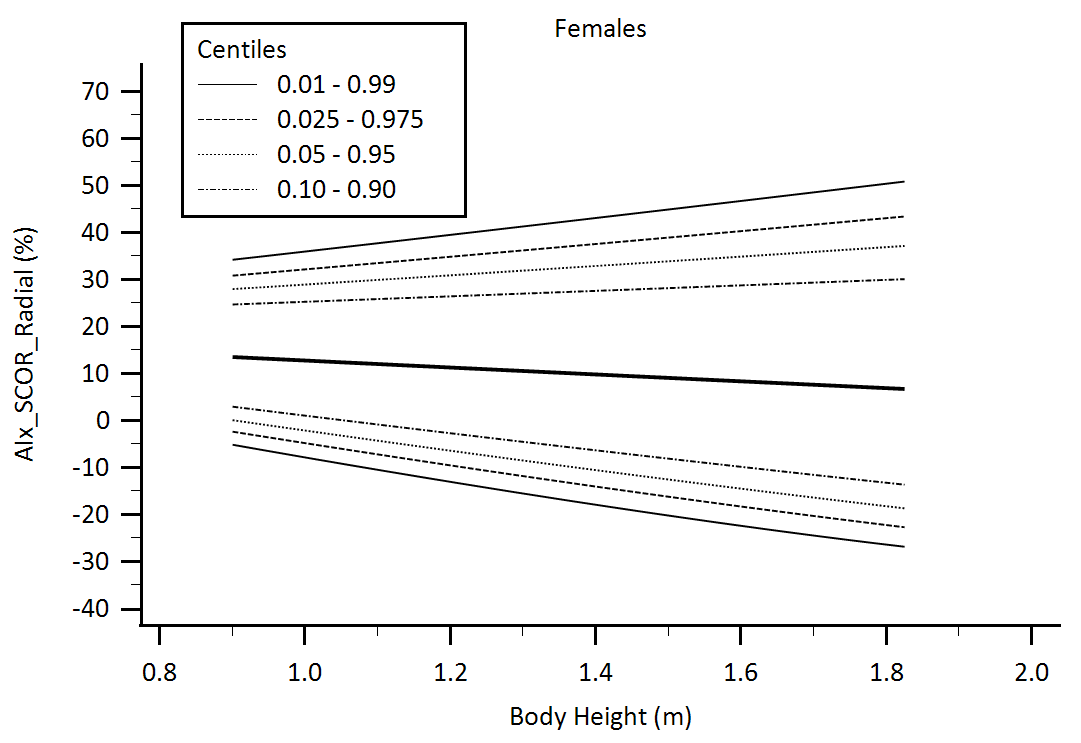

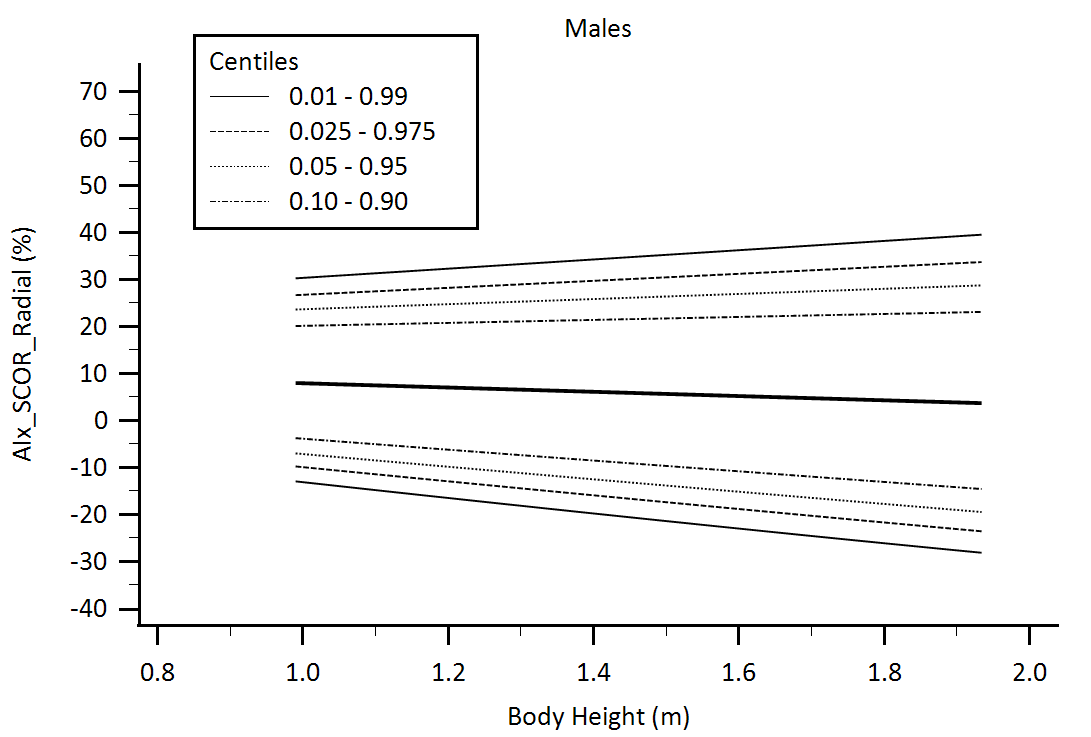


Supplementary Figure 30. Central aortic waveform-derived parameters obtained using radial artery applanation tonometry (SphygmoCor device, SCOR): Augmentation Index (AIx) body height-related percentiles.


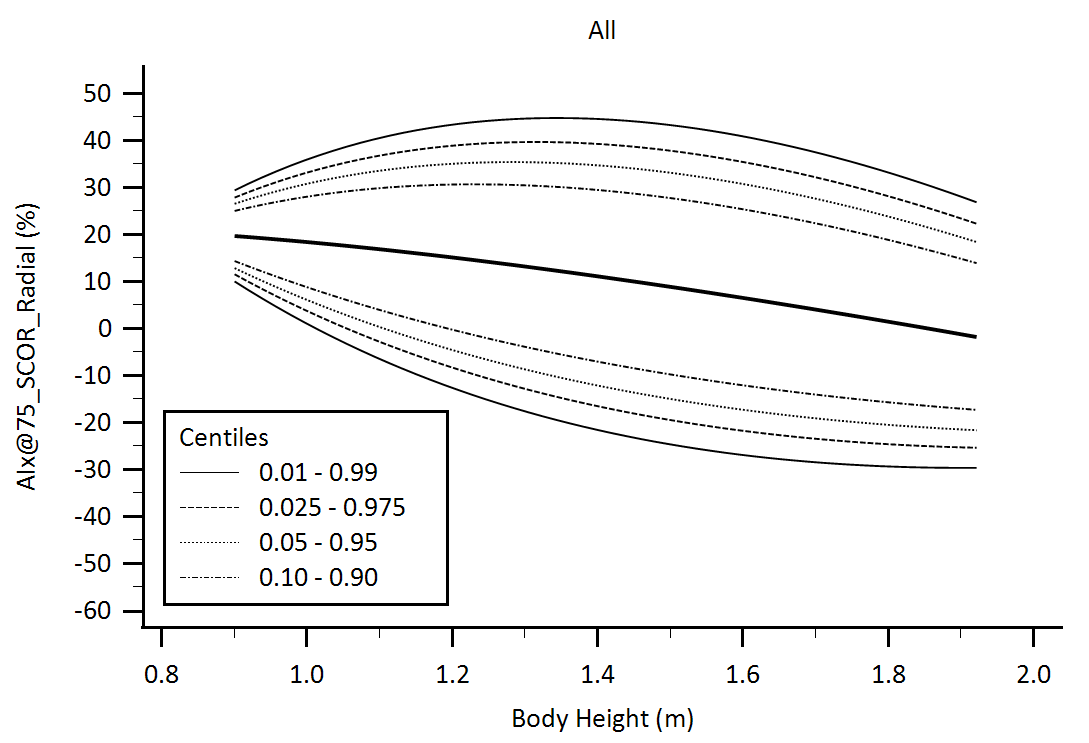


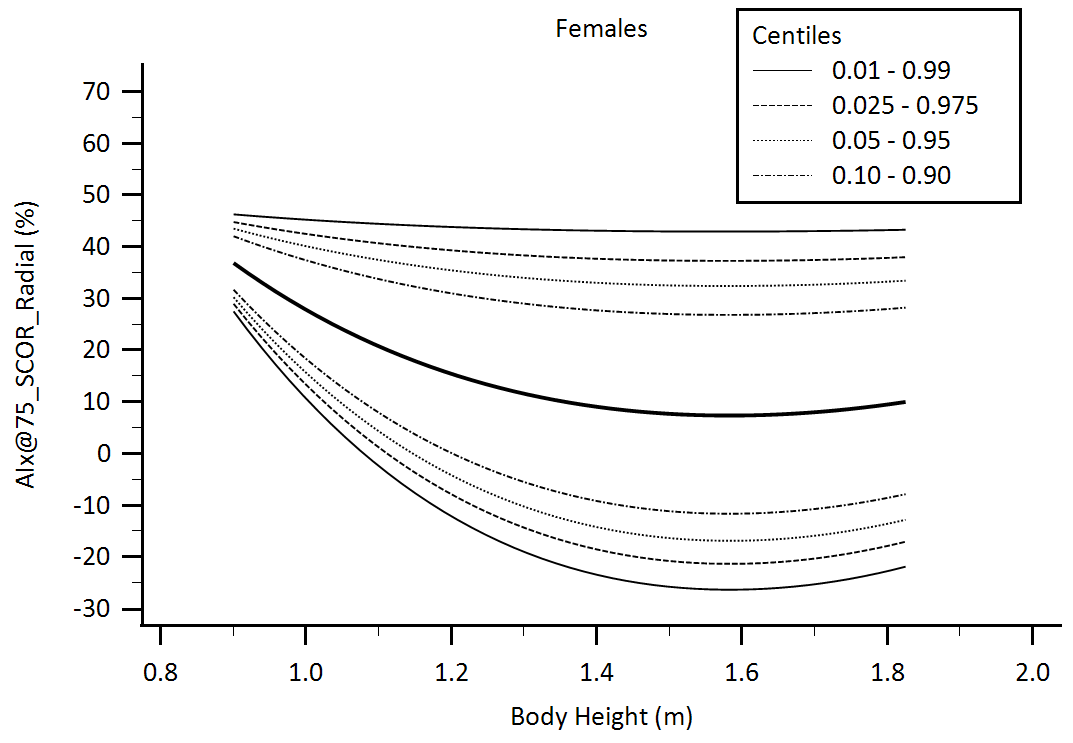


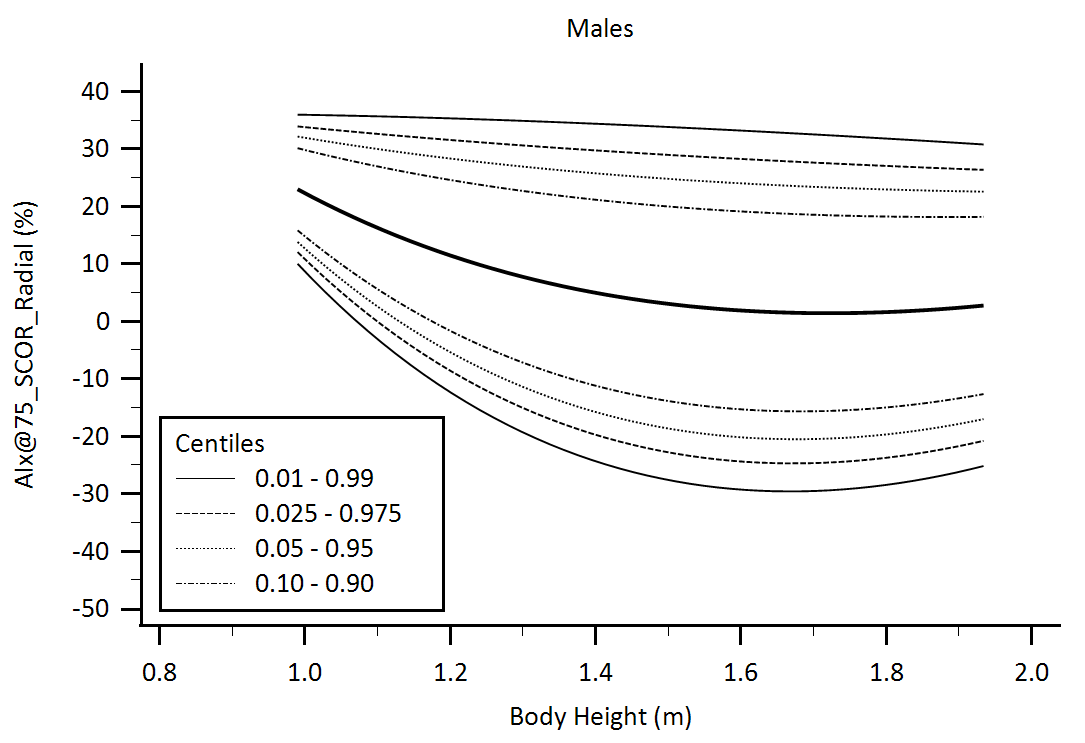


Supplementary Figure 31. Central aortic waveform-derived parameters obtained using radial artery applanation tonometry (SphygmoCor device, SCOR): Augmentation Index adjusted for heart rate equal 75 beats/minute (AIx@75) body height-related percentiles.


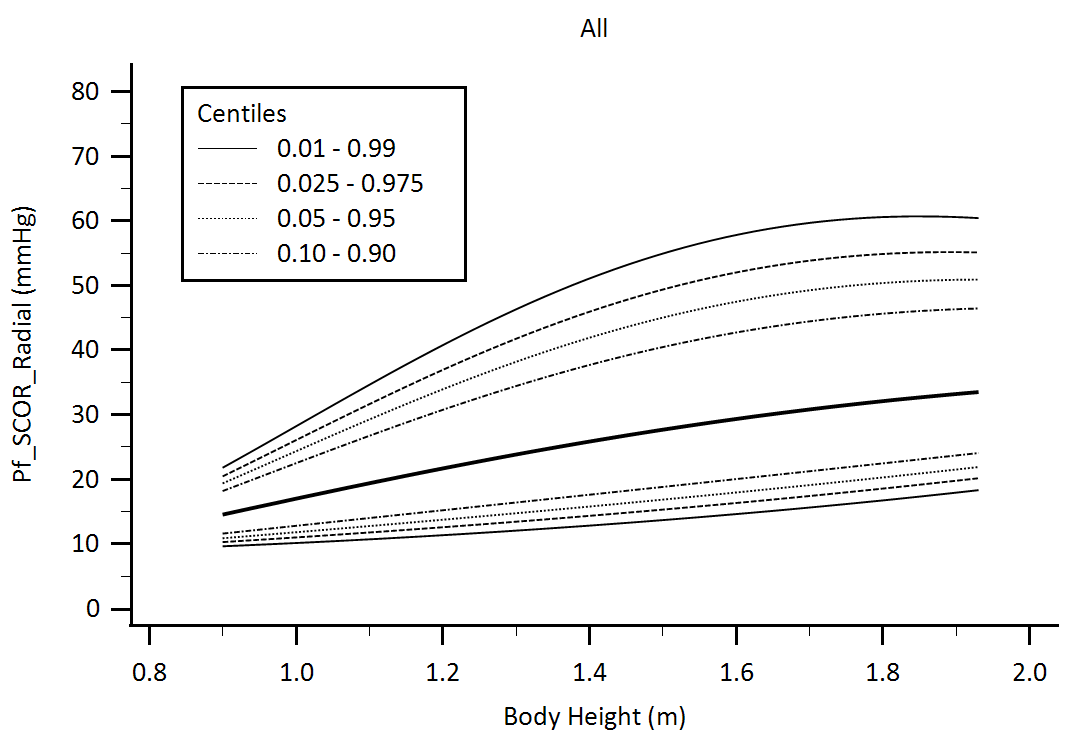

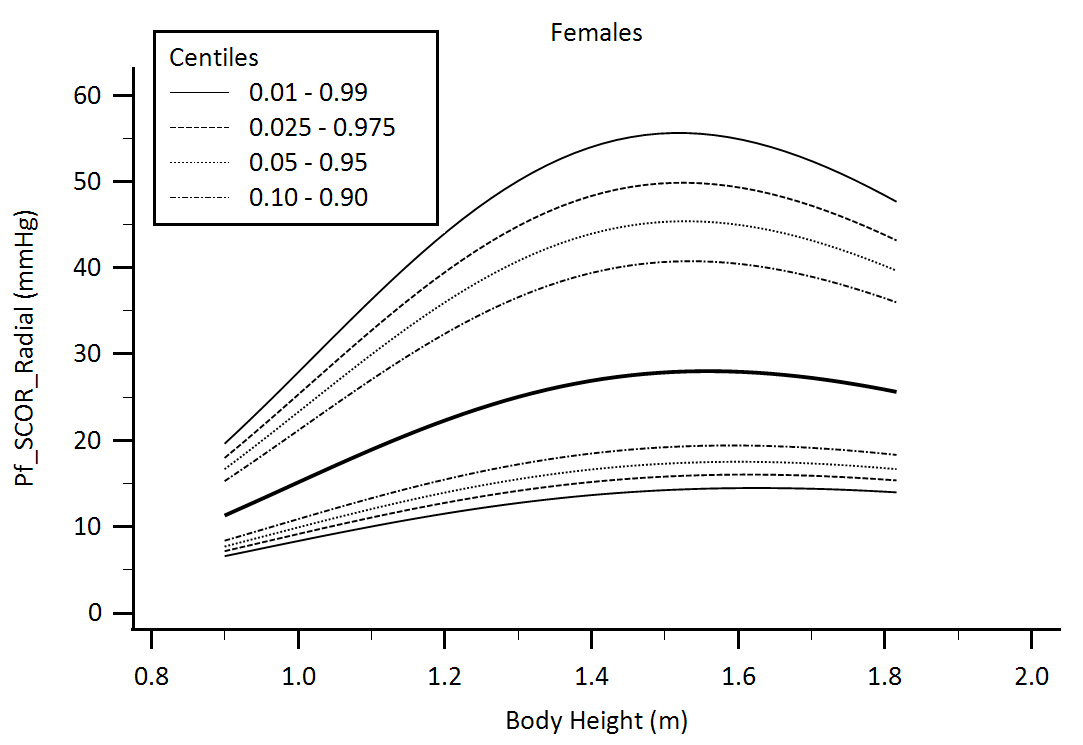

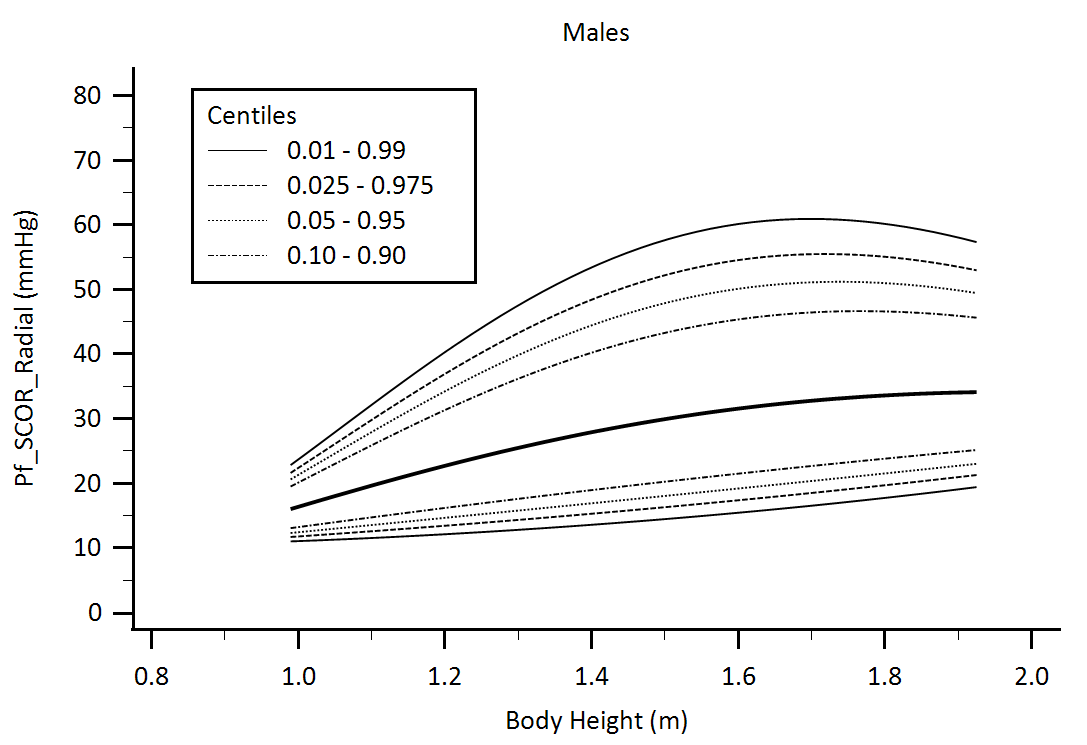


Supplementary Figure 32. Central aortic waveform-derived parameters obtained using radial artery applanation tonometry (SphygmoCor device, SCOR): Forward Pressure (Pf) body height-related percentiles.


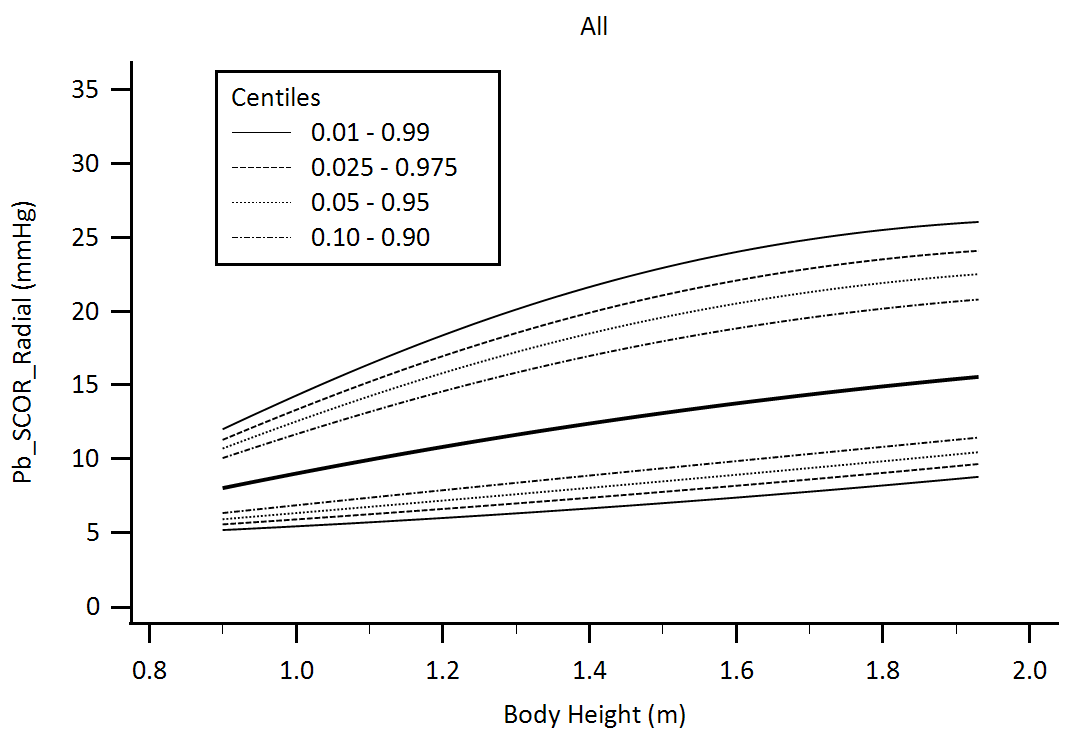


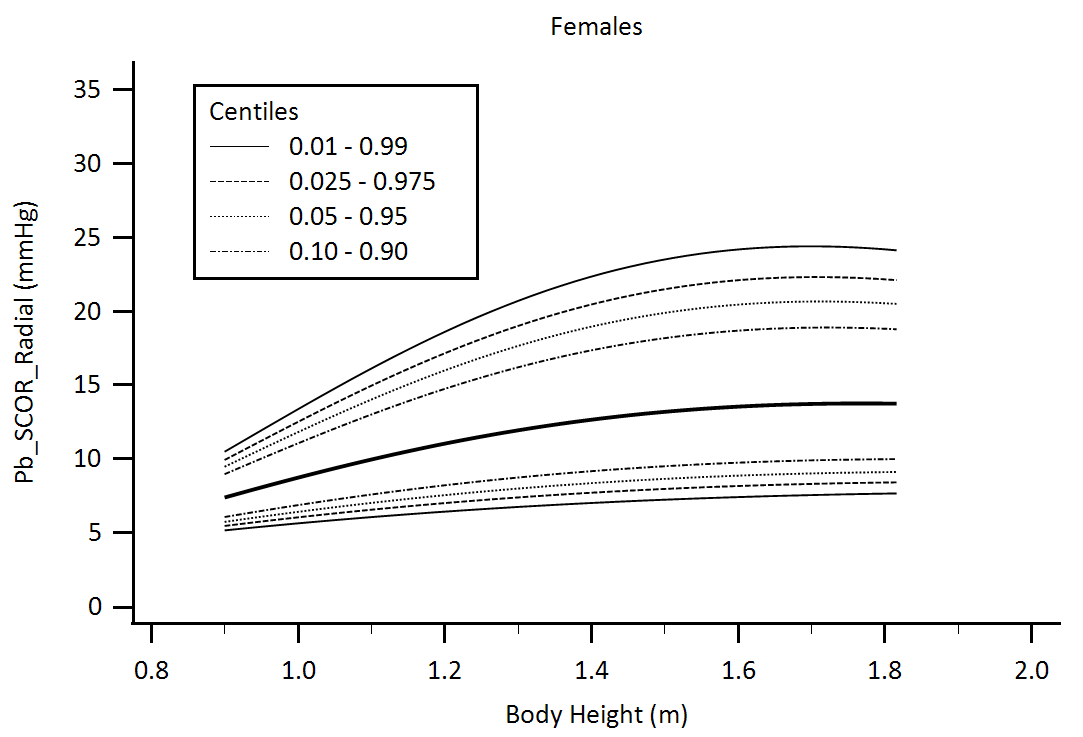


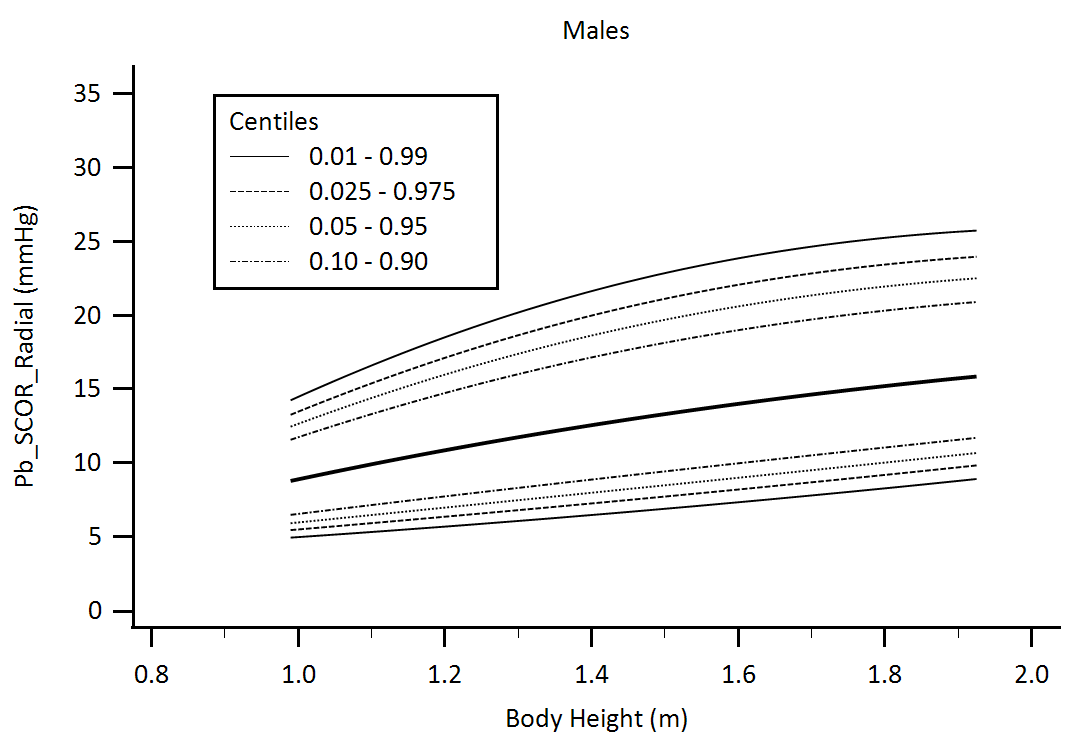


Supplementary Figure 33. Central aortic waveform-derived parameters obtained using radial artery applanation tonometry (SphygmoCor device, SCOR): Backward Pressure (Pb) body height-related percentiles.


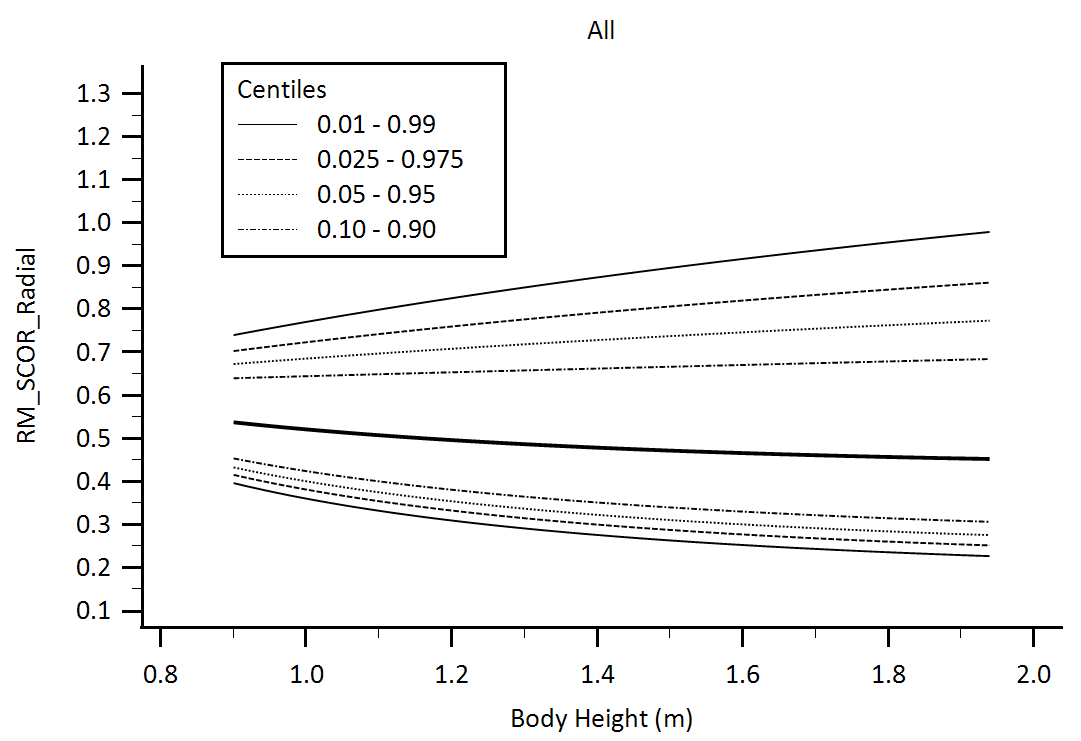


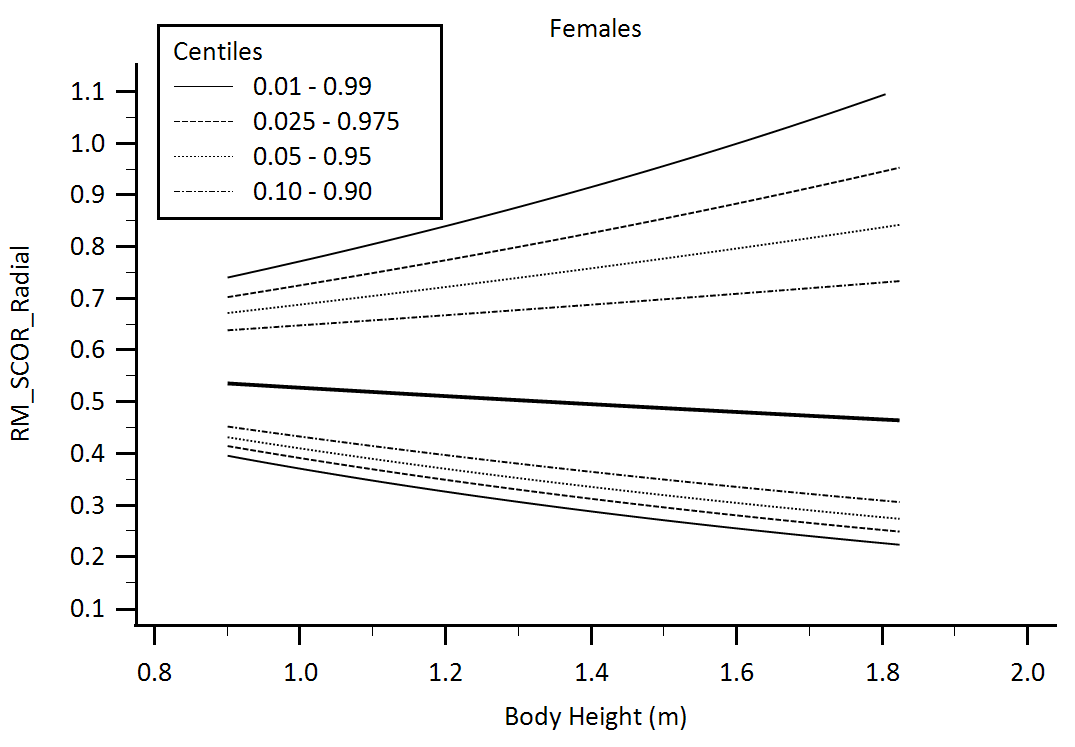

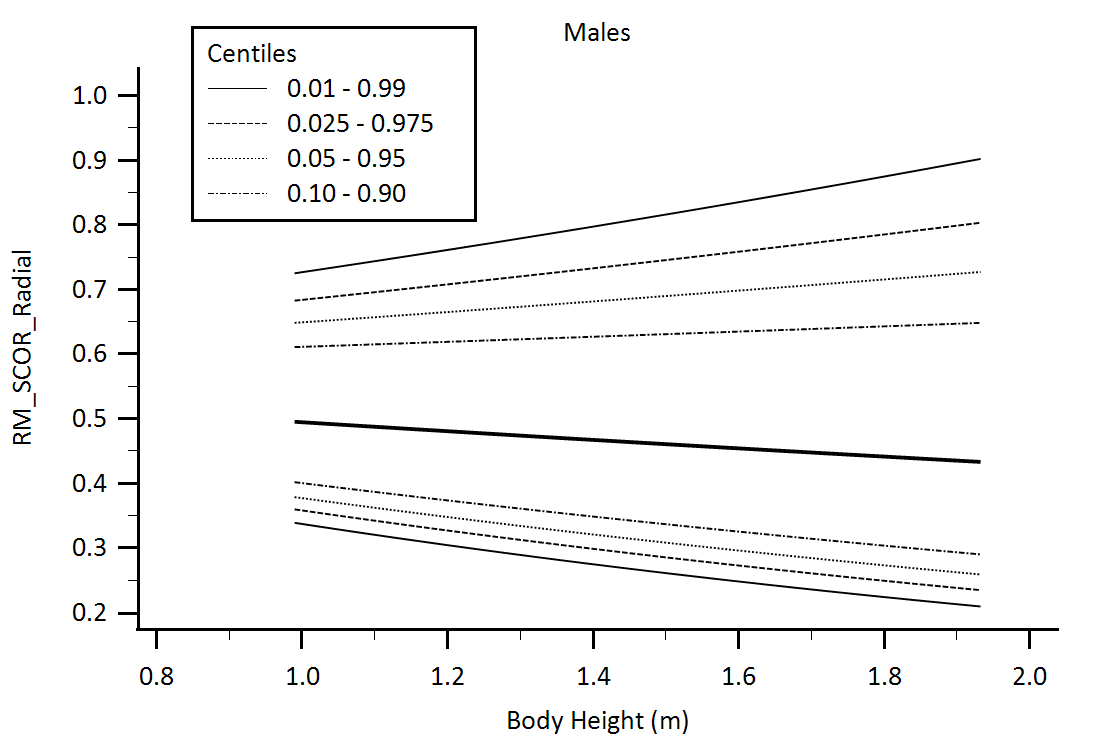


Supplementary Figure 34. Central aortic waveform-derived parameters obtained using radial artery applanation tonometry (SphygmoCor device, SCOR): Reflection Magnitude (RM) body height-related percentiles.


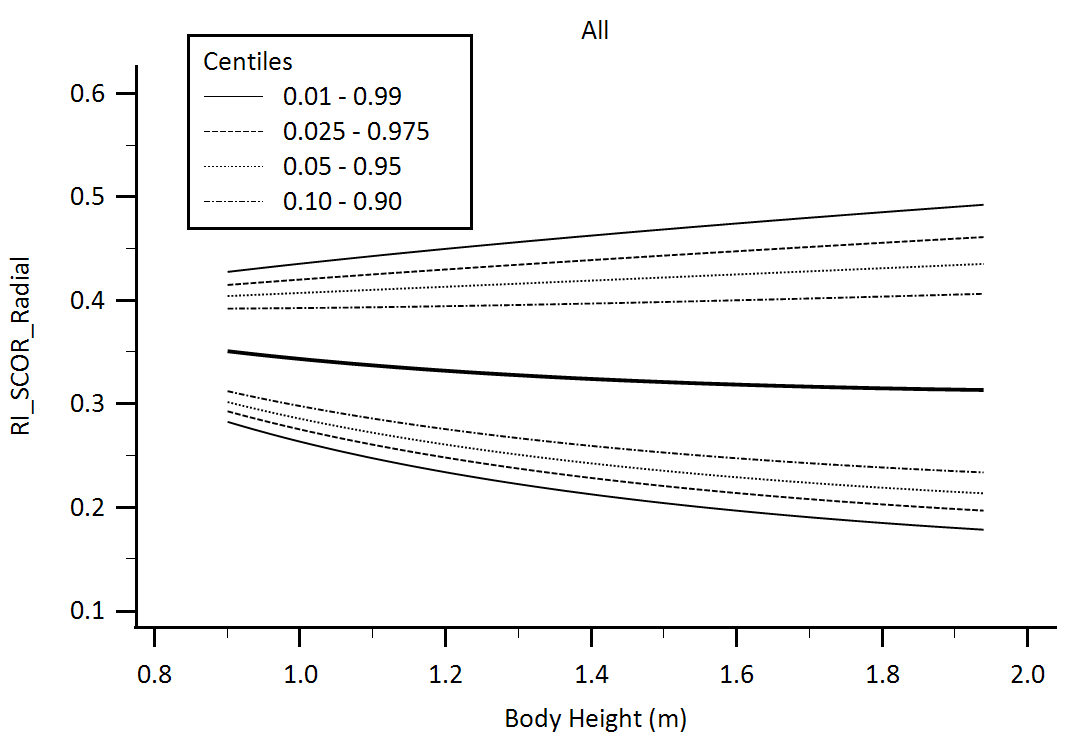

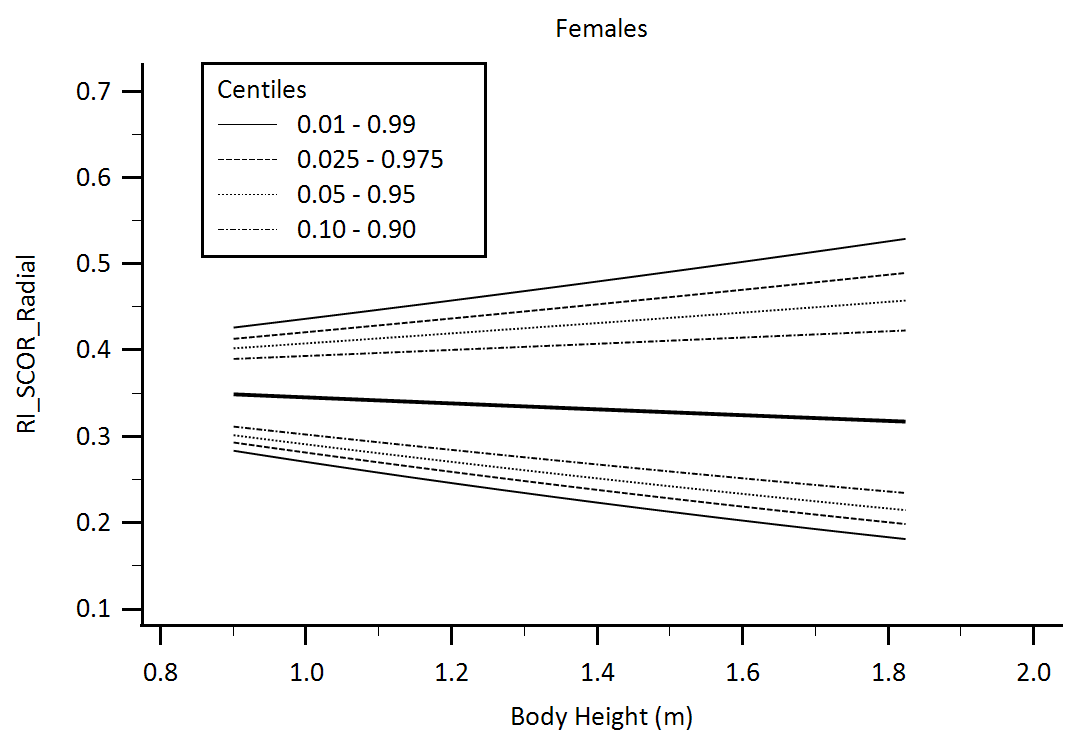

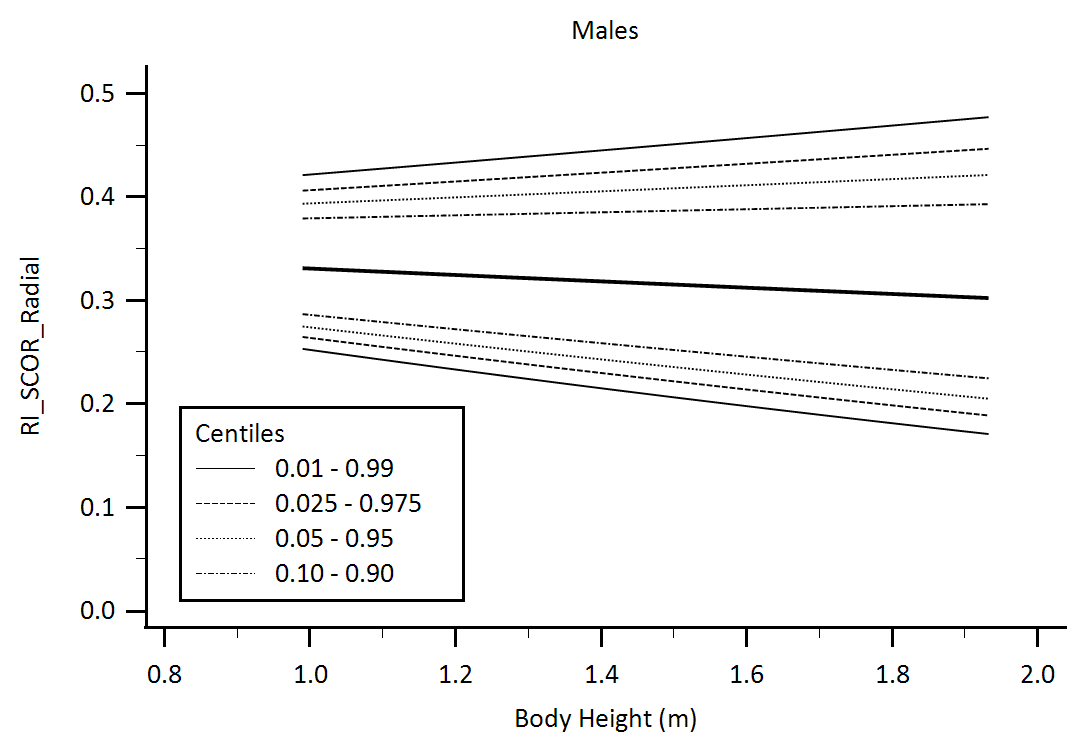


Supplementary Figure 35. Central aortic waveform-derived parameters obtained using radial artery applanation tonometry (SphygmoCor device, SCOR): Reflection Index (RI) body height-related percentiles.


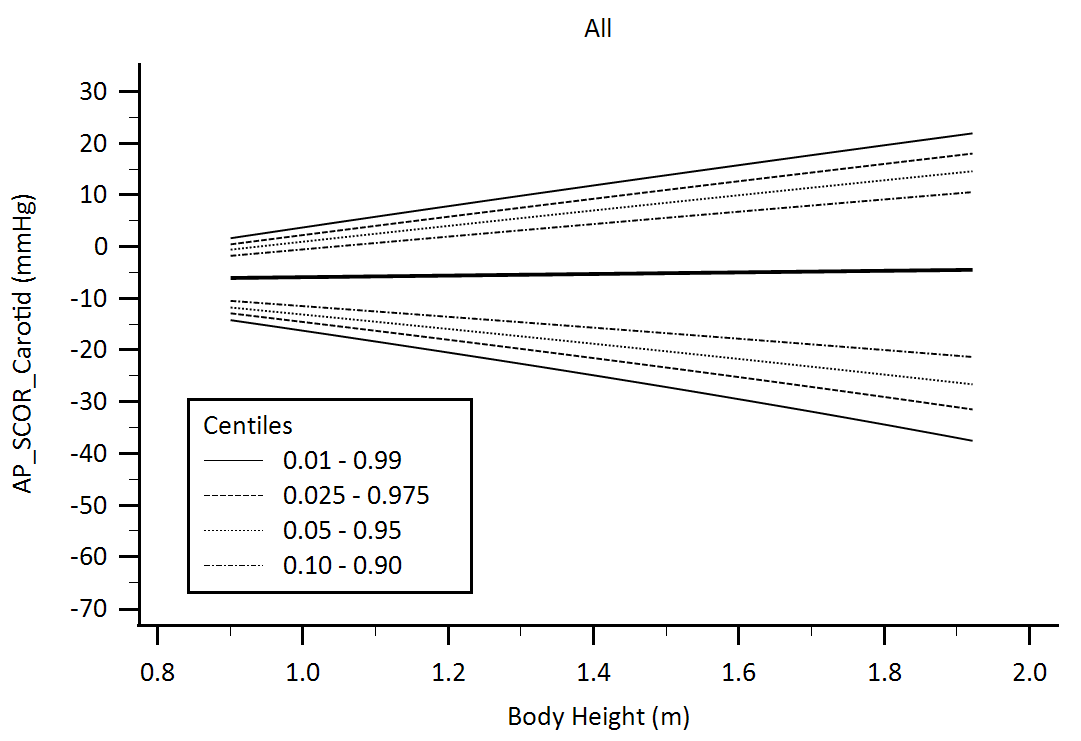


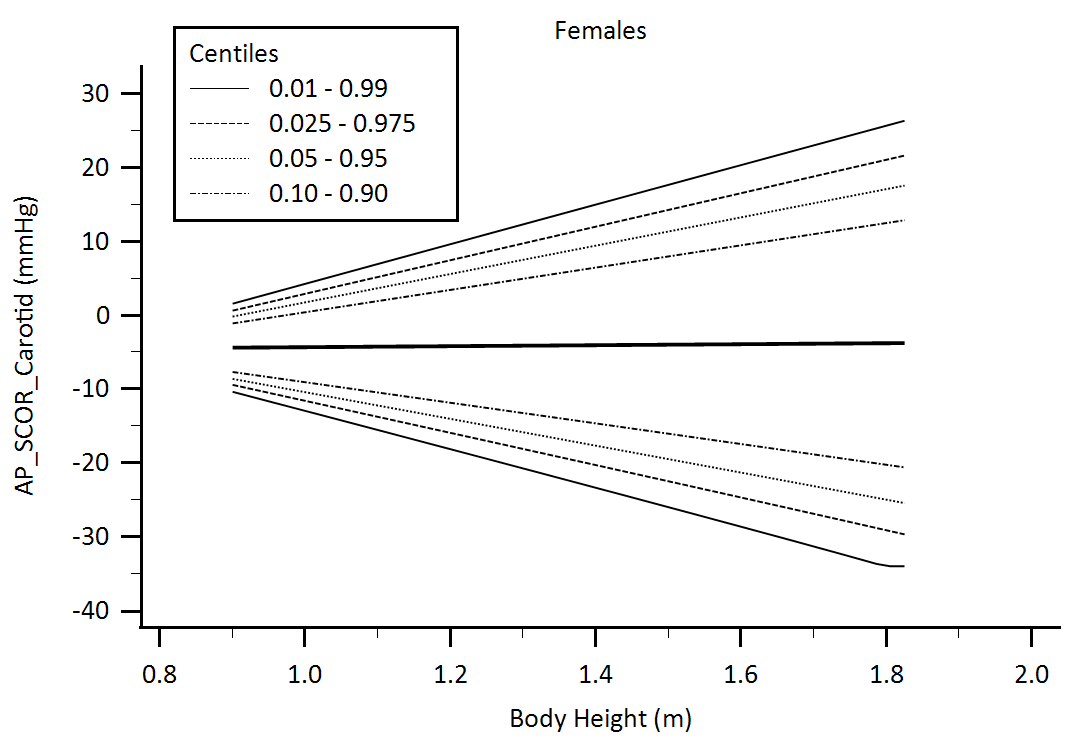

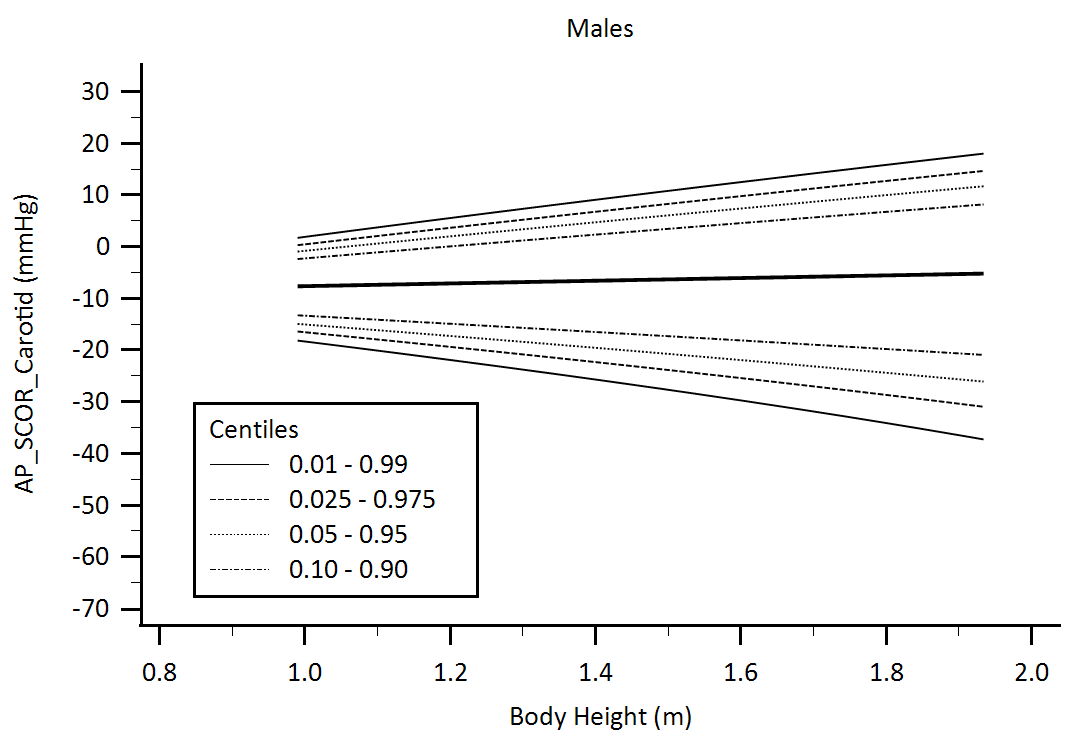


Supplementary Figure 36. Central aortic waveform-derived parameters obtained using carotid artery applanation tonometry (SphygmoCor device, SCOR): Augmentation Pressure (AP) body height-related percentiles.


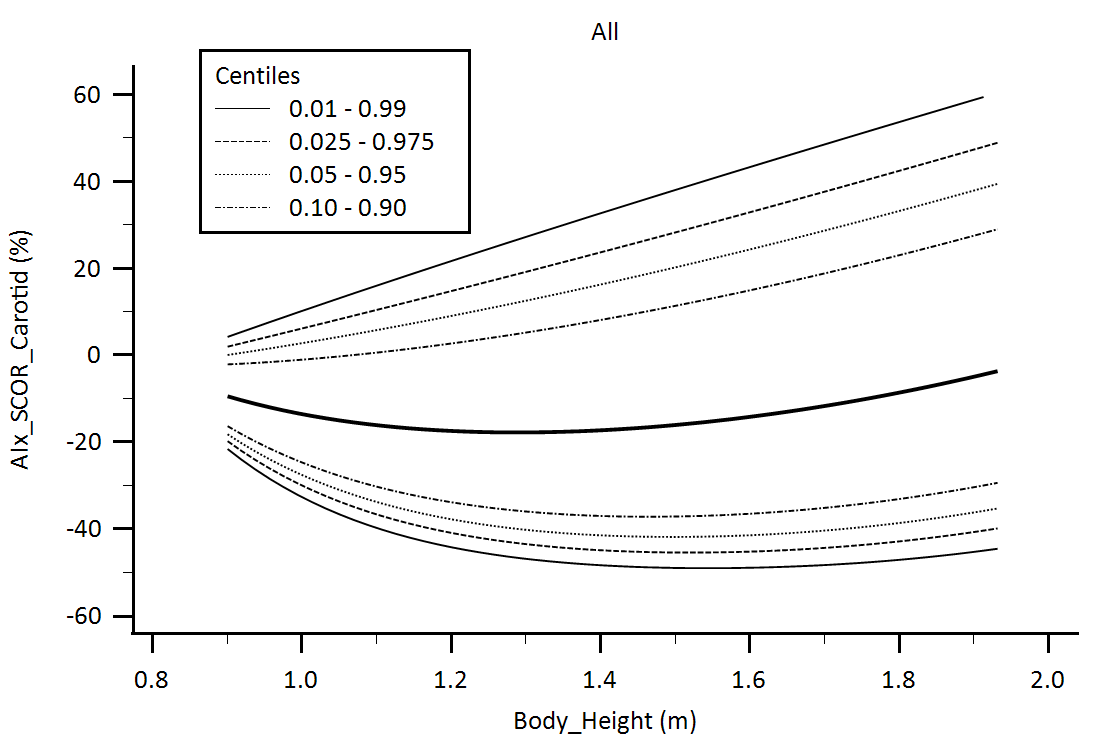

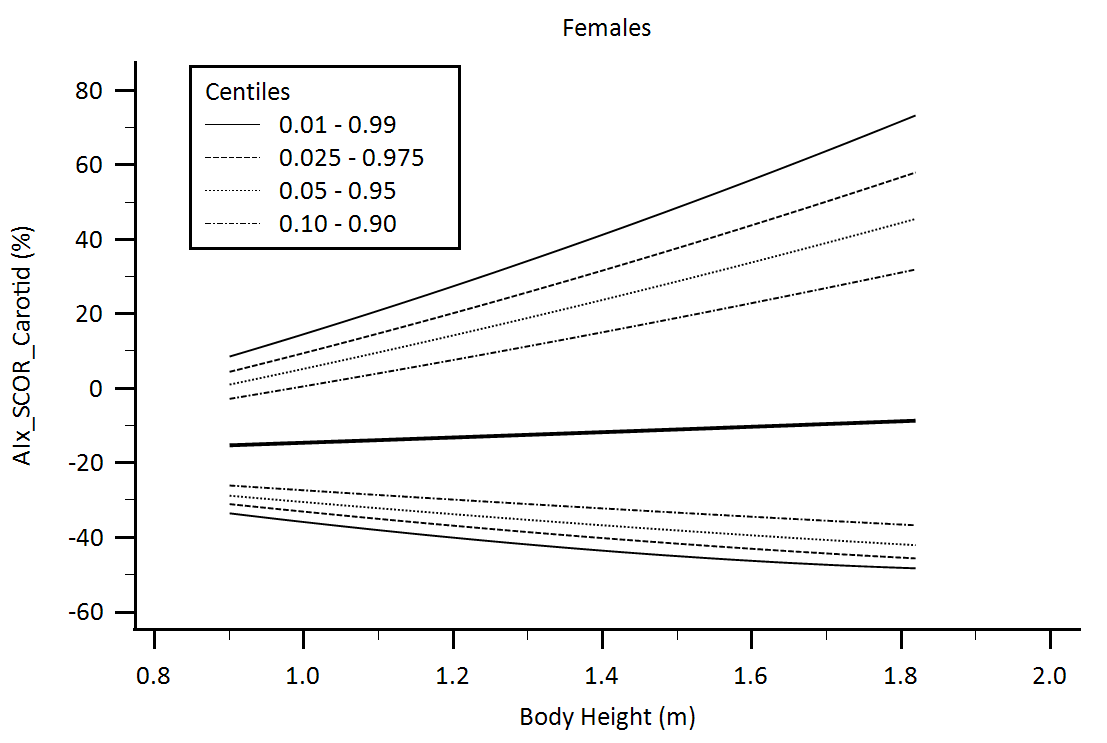

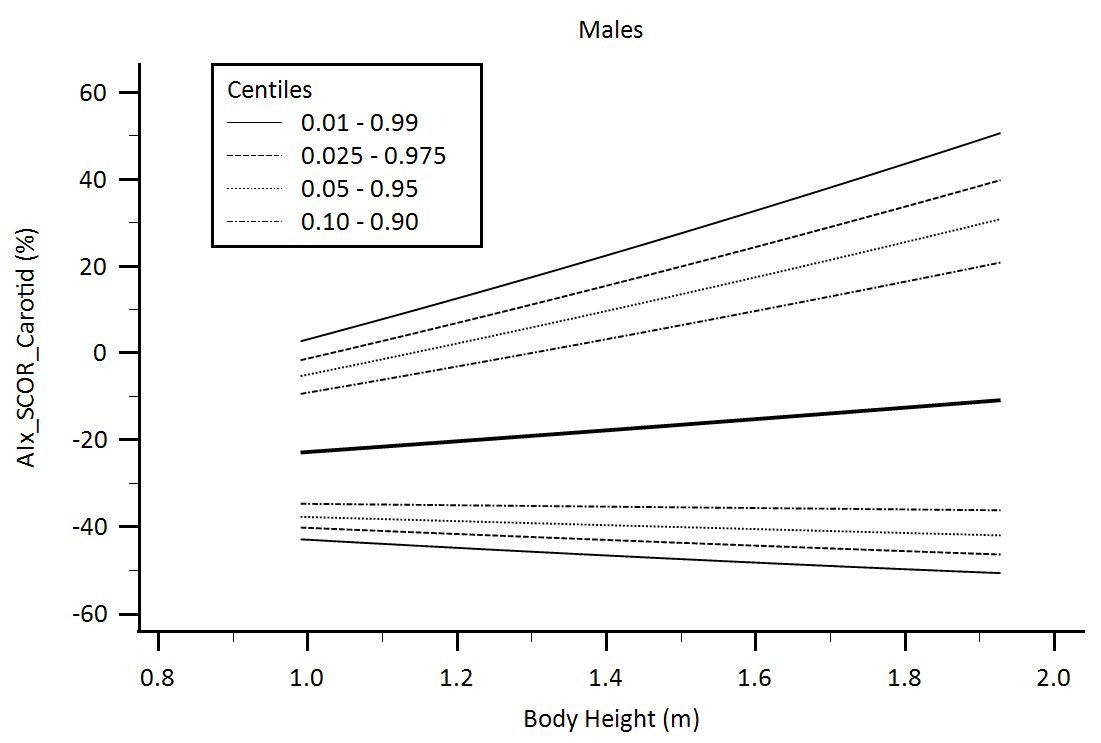


Supplementary Figure 37. Central aortic waveform-derived parameters obtained using carotid artery applanation tonometry (SphygmoCor device, SCOR): Augmentation Index (AIx) body height-related percentiles.


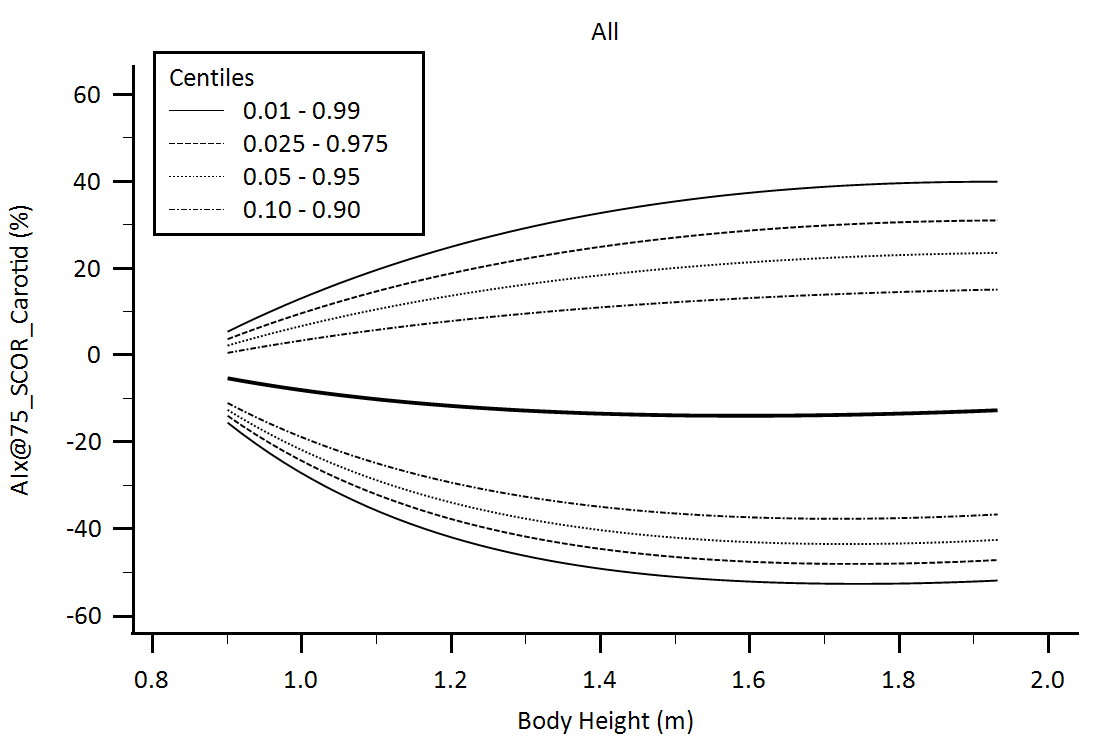

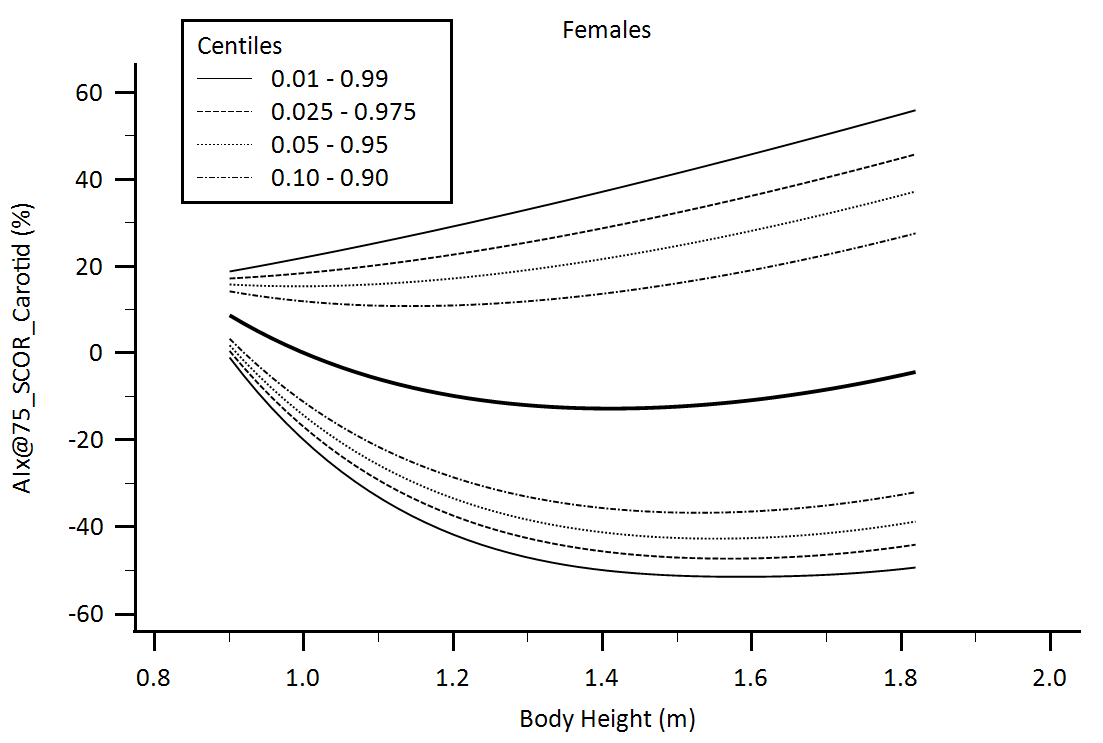

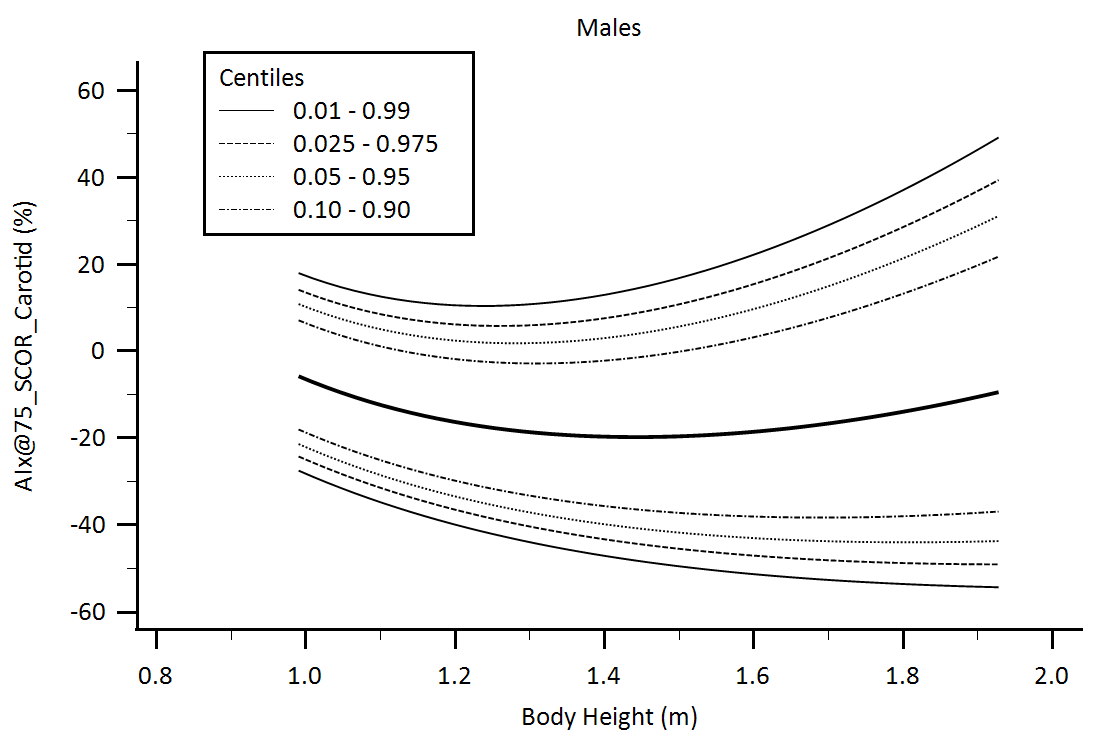


Supplementary Figure 38. Central aortic waveform-derived parameters obtained using carotid artery applanation tonometry (SphygmoCor device, SCOR): Augmentation Index adjusted for heart rate equal 75 beats/minute (AIx@75) body height-related percentiles.


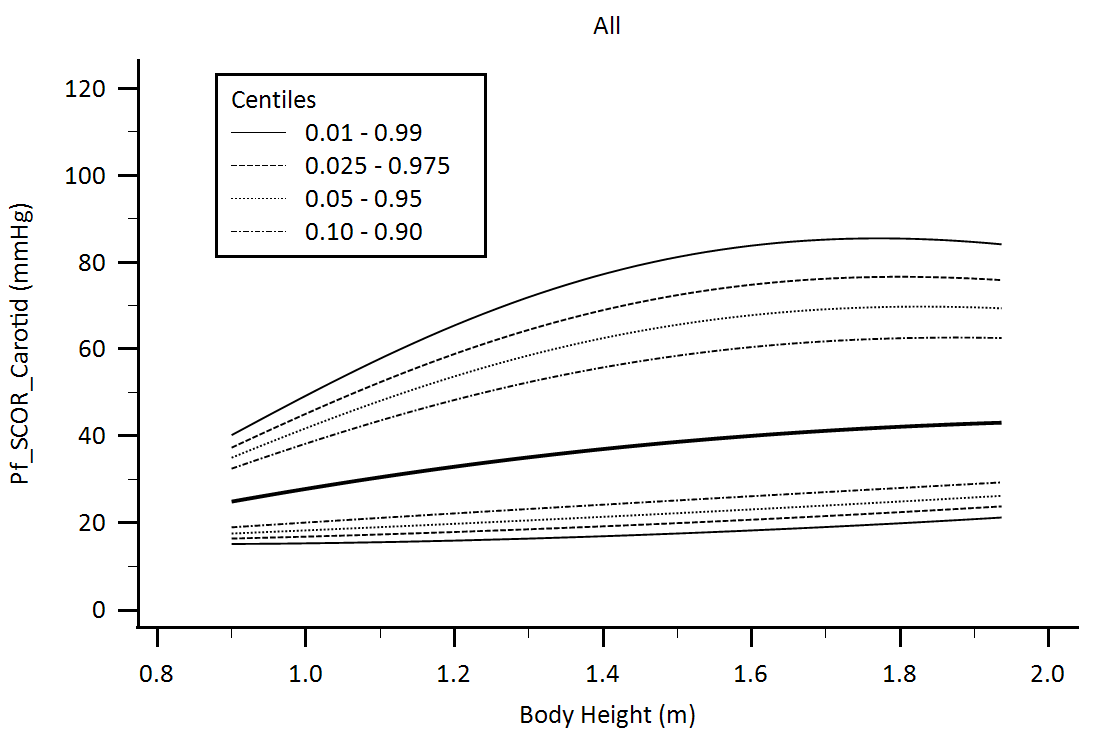

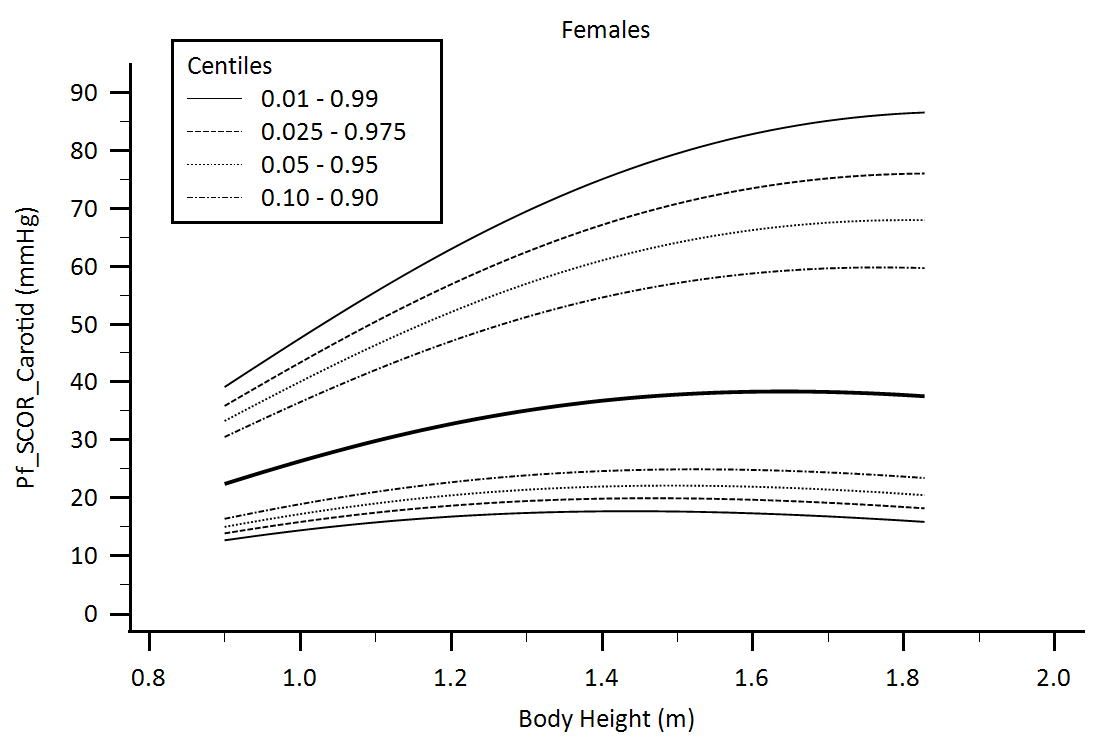

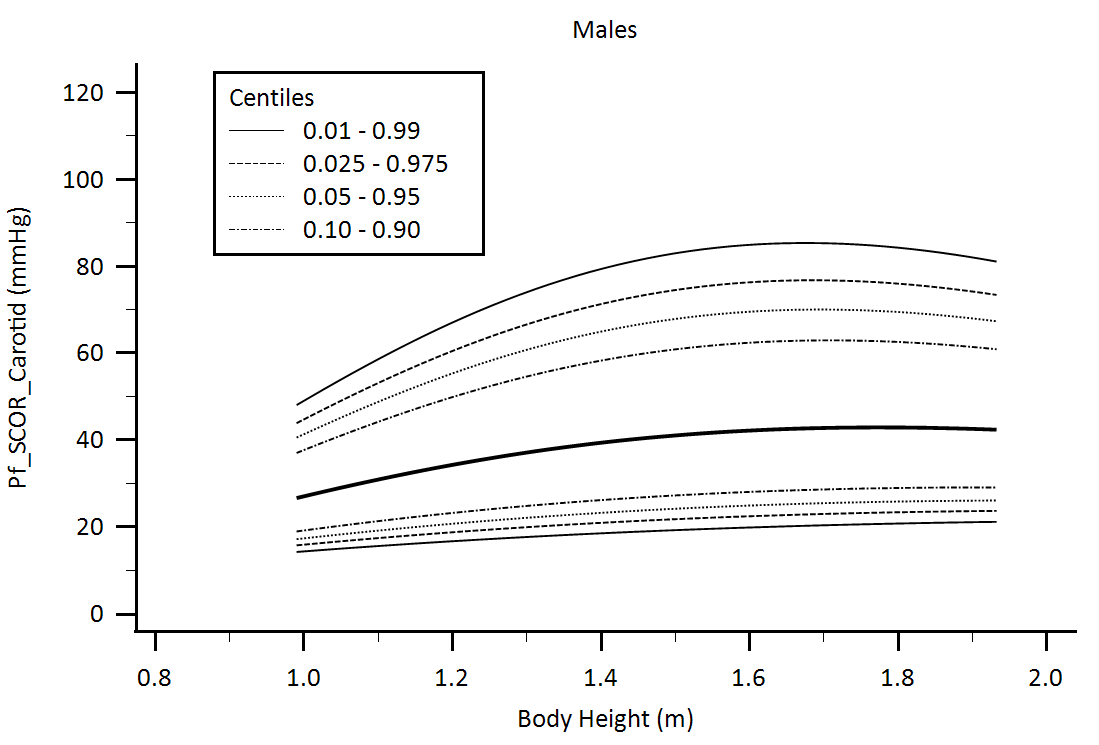


Supplementary Figure 39. Central aortic waveform-derived parameters obtained using carotid artery applanation tonometry (SphygmoCor device, SCOR): Forward Pressure (Pf) body height-related percentiles.


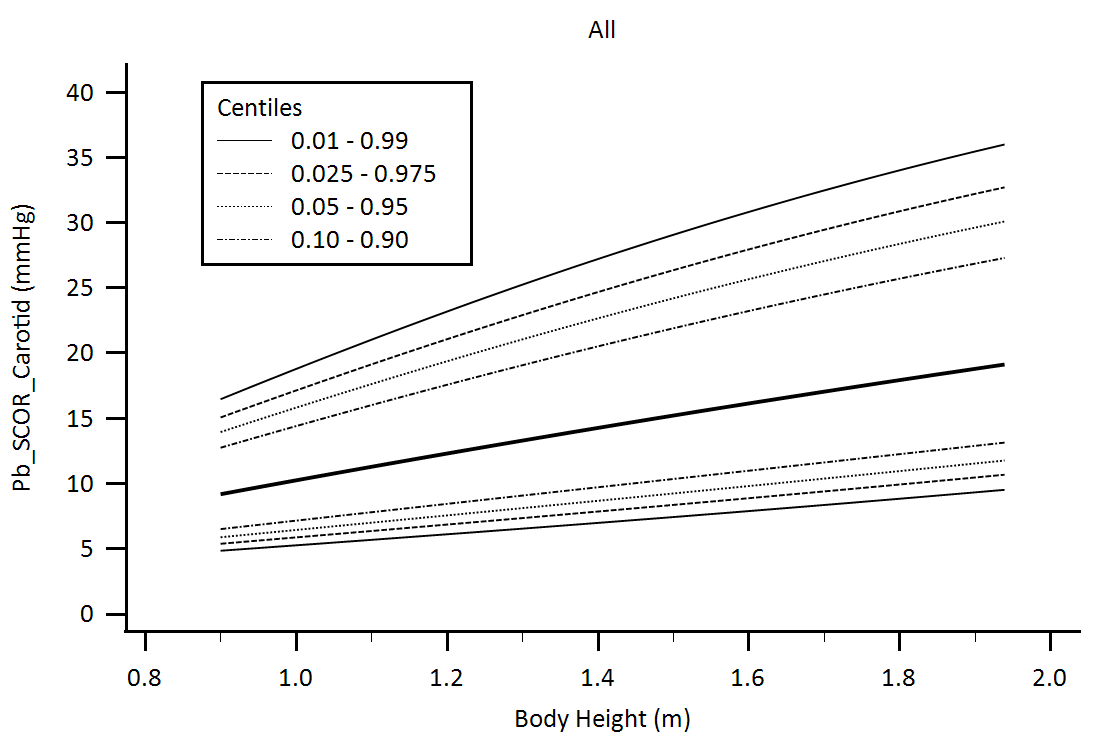

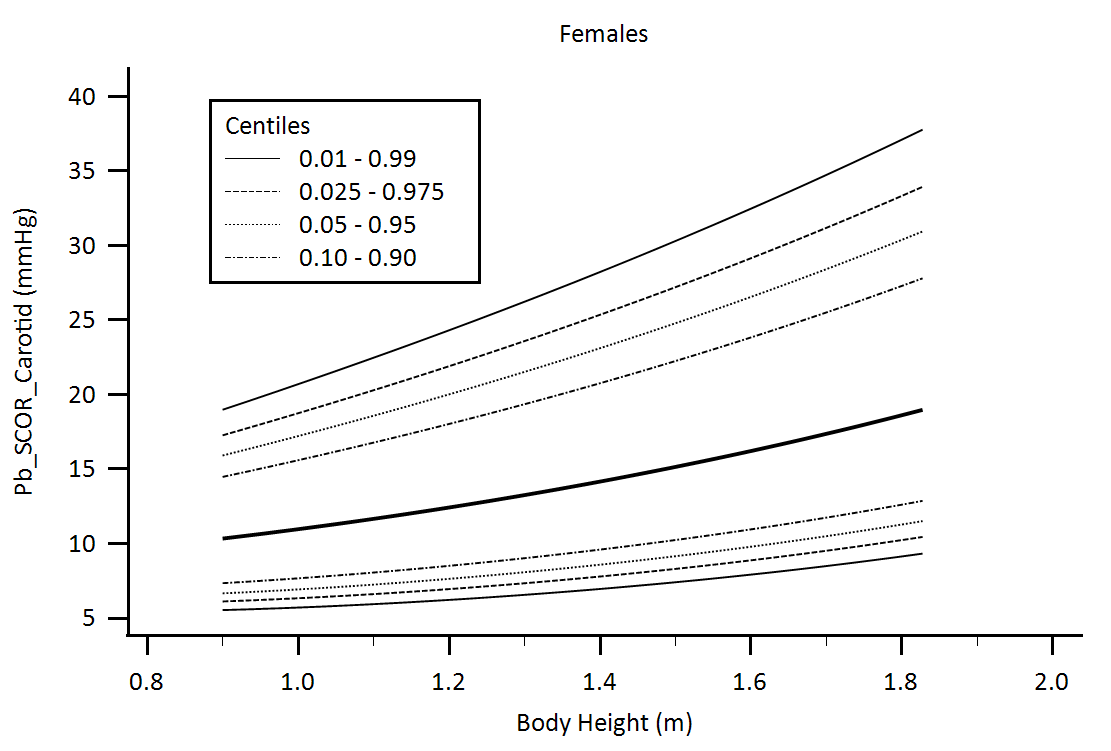

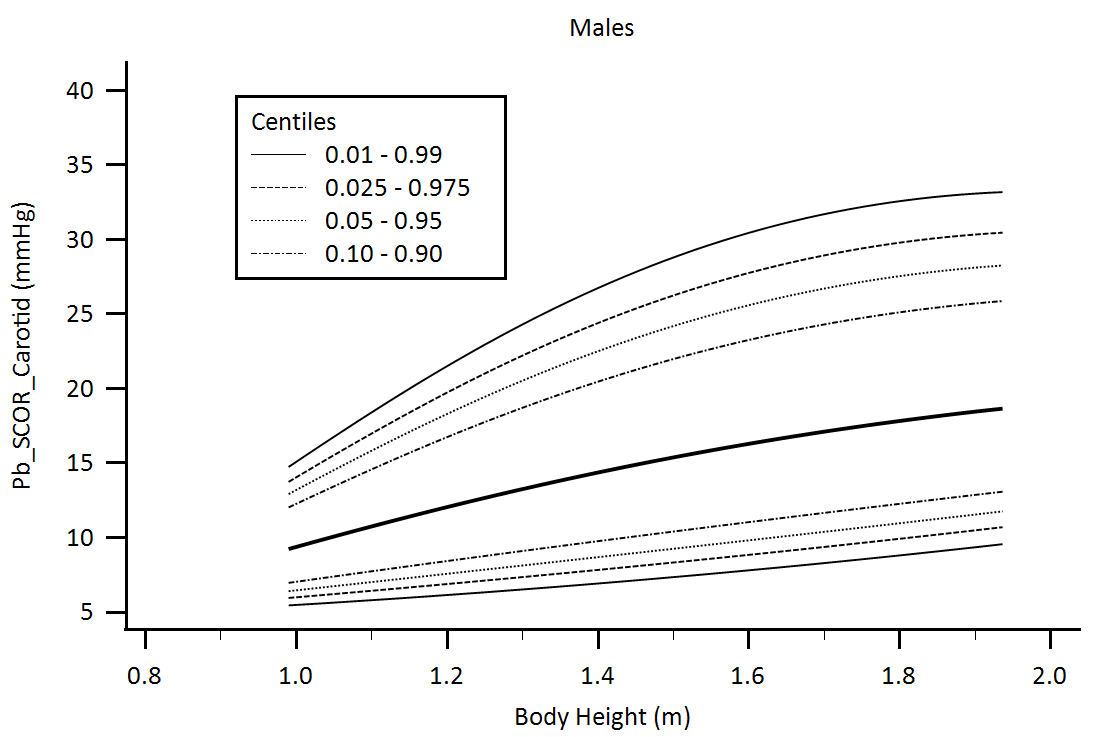


Supplementary Figure 40. Central aortic waveform-derived parameters obtained using carotid artery applanation tonometry (SphygmoCor device, SCOR): Backward Pressure (Pb) body height-related percentiles.


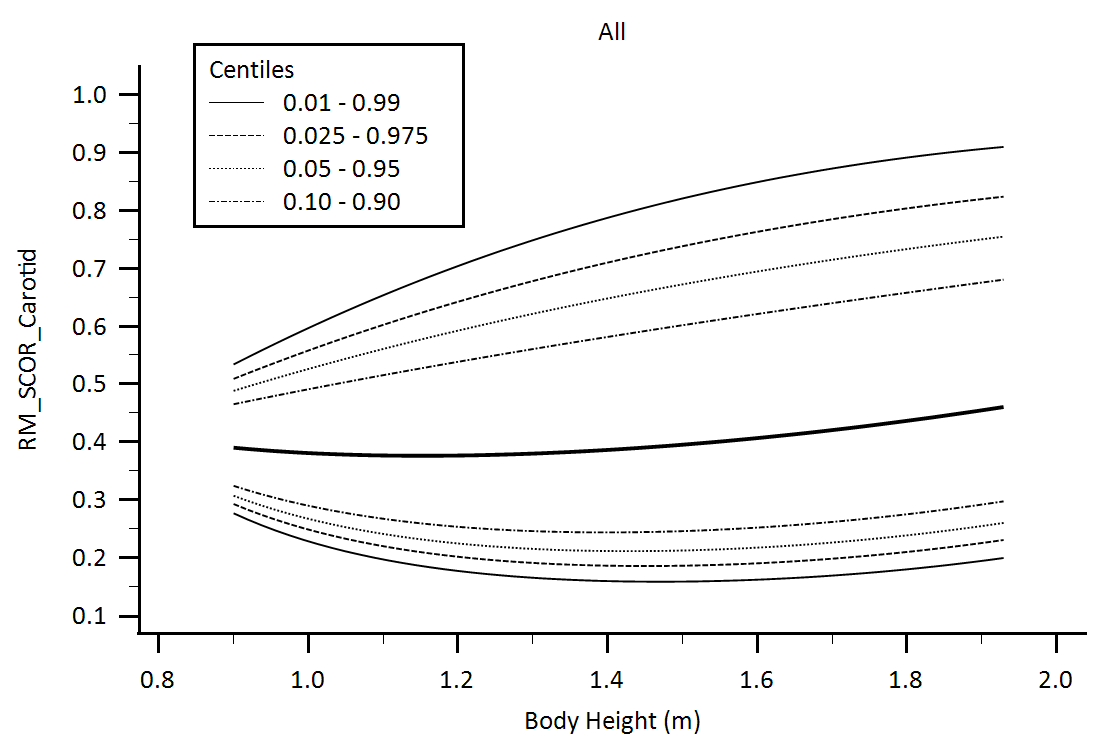

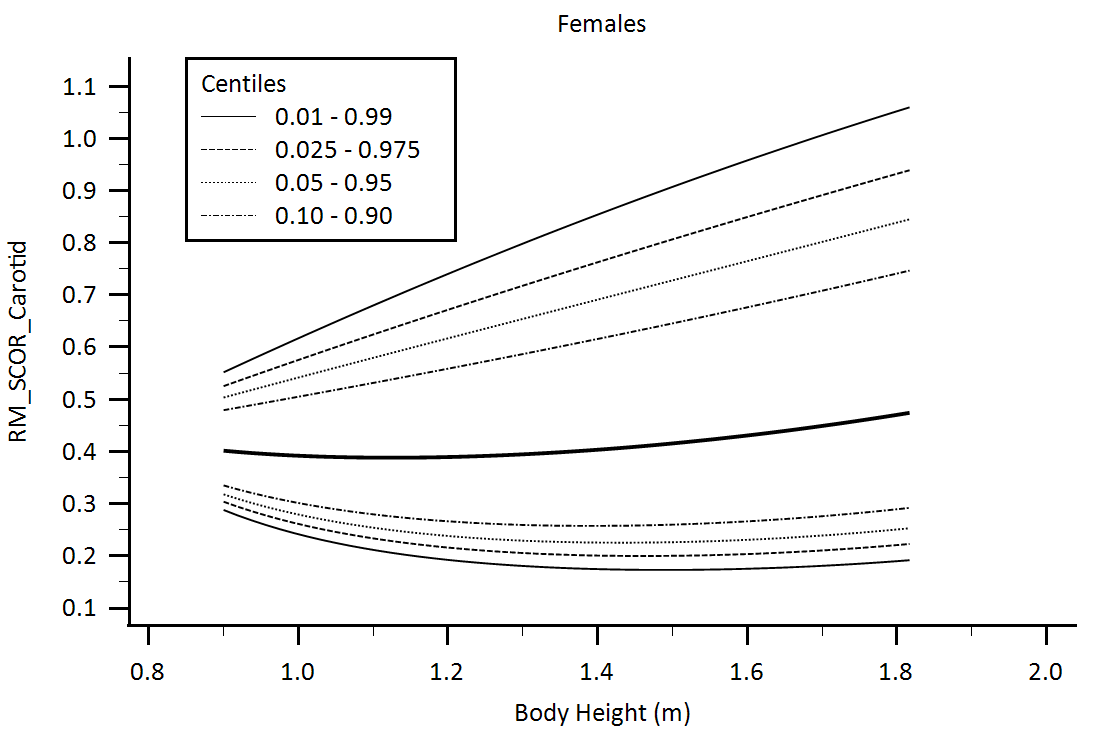

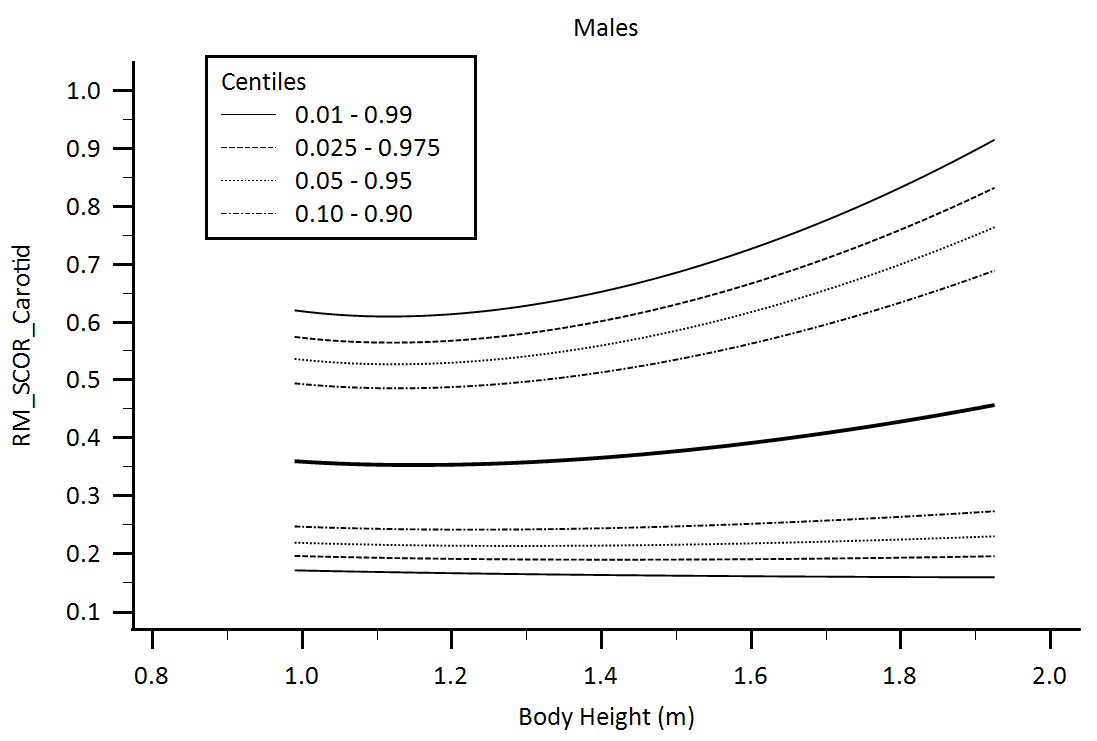


Supplementary Figure 41. Central aortic waveform-derived parameters obtained using carotid artery applanation tonometry (SphygmoCor device, SCOR): Reflection Magnitude (RM) body height-related percentiles.


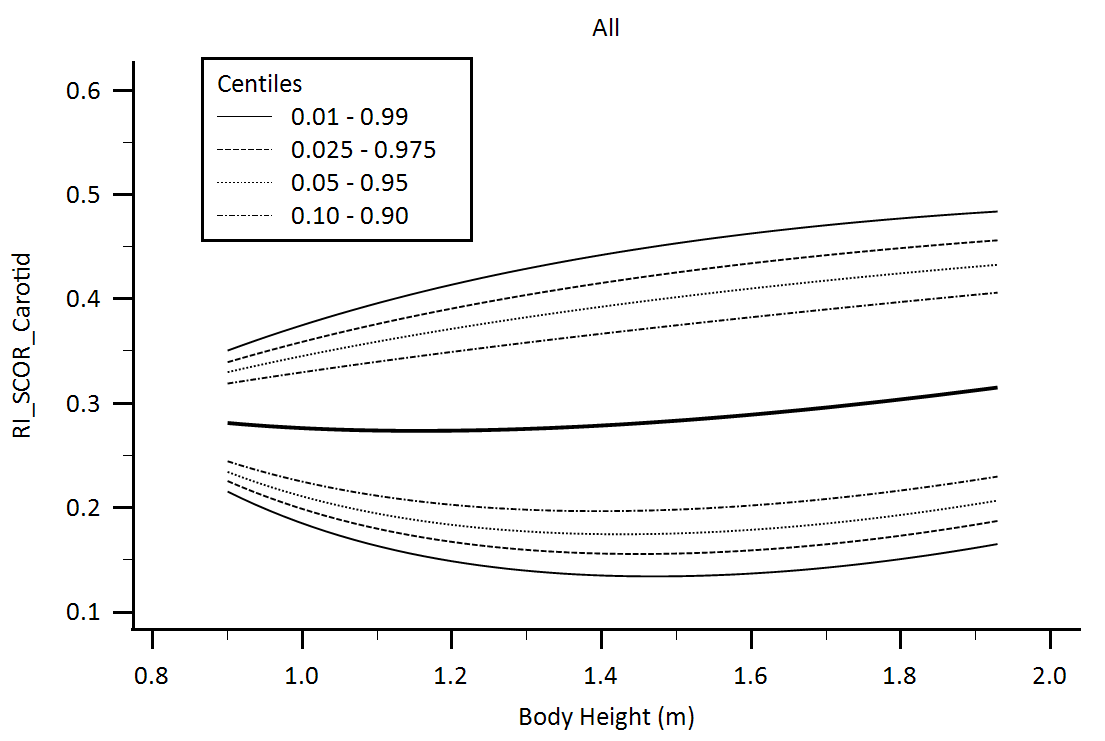

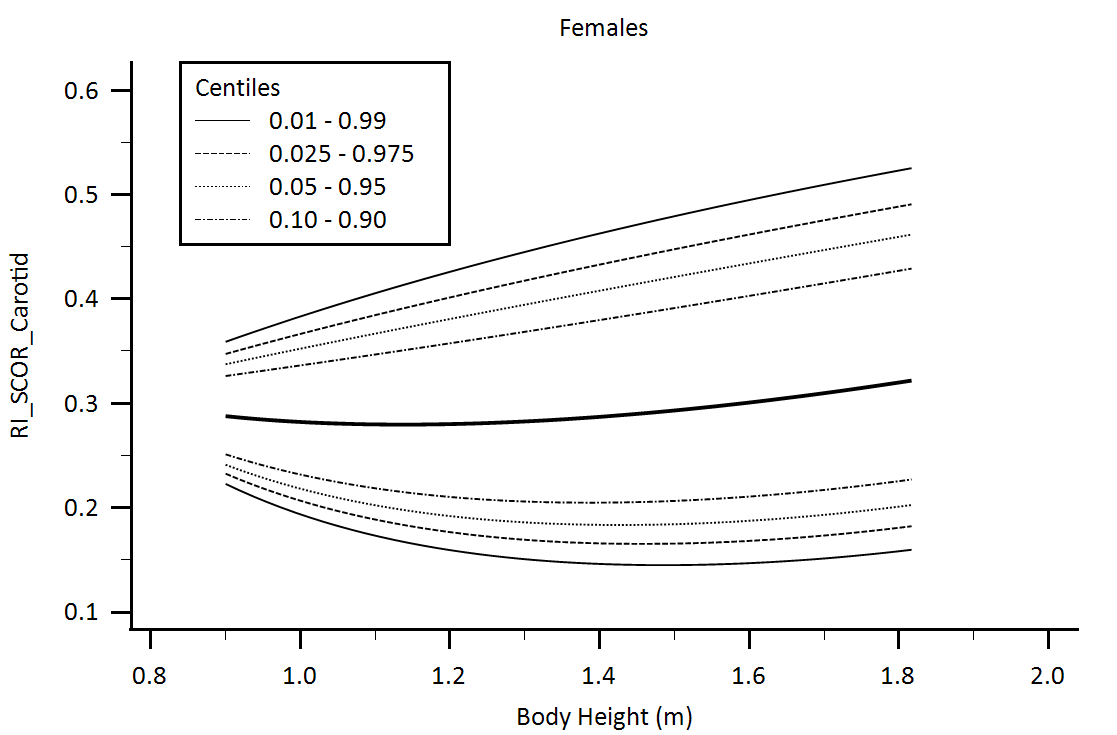

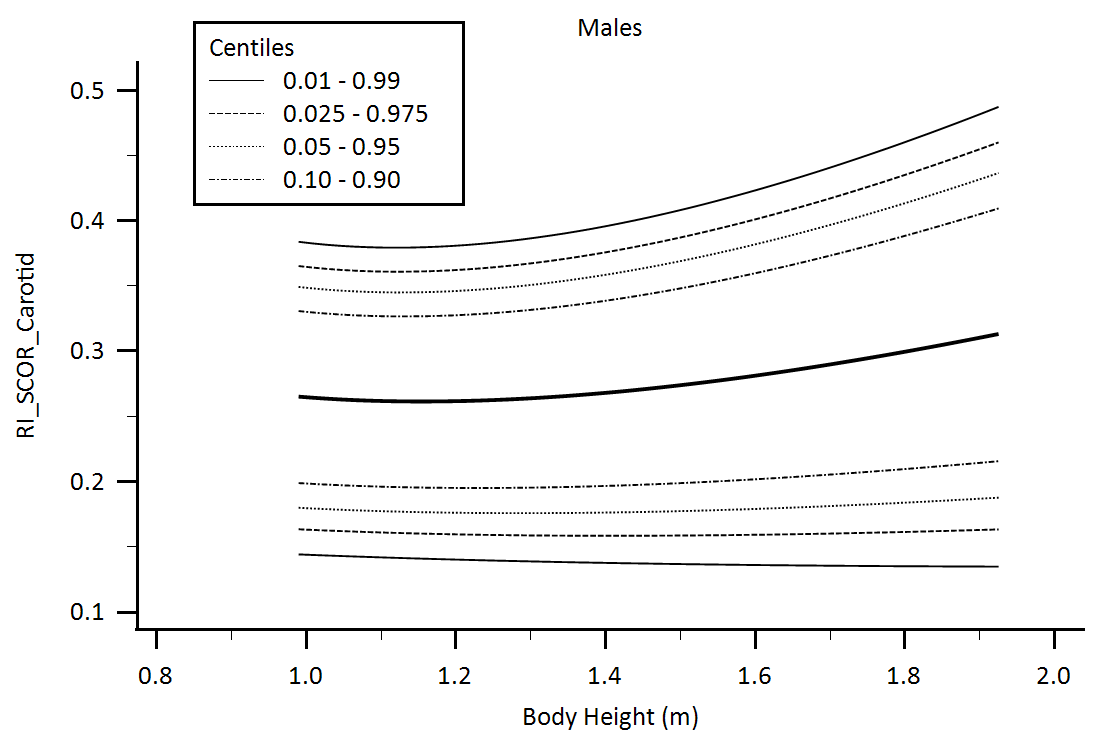


Supplementary Figure 42. Central aortic waveform-derived parameters obtained using carotid artery applanation tonometry (SphygmoCor device, SCOR): Reflection Index (RI) body height-related percentiles.
